# Exome sequencing of the TCL1 mouse model for CLL reveals genetic heterogeneity and dynamics during disease development

**DOI:** 10.1038/s41375-018-0260-4

**Published:** 2018-09-27

**Authors:** Nadja Zaborsky, Franz J. Gassner, Jan P. Höpner, Maria Schubert, Daniel Hebenstreit, Richard Stark, Daniela Asslaber, Markus Steiner, Roland Geisberger, Richard Greil, Alexander Egle

**Affiliations:** 1 Department of Internal Medicine III with Haematology, Medical Oncology, Haemostaseology, Infectiology and Rheumatology, Oncologic Center, Paracelsus Medical University, Salzburg, Austria; 2 Salzburg Cancer Research Institute - Laboratory for Immunological and Molecular Cancer Research (SCRI-LIMCR), Salzburg, Austria; 3 Cancer Cluster Salzburg, Salzburg, Austria; 4 School of Life Sciences, University of Warwick, Coventry, UK

**Keywords:** Cancer genetics, Cancer genetics

## Abstract

The TCL1 mouse model is widely used to study pathophysiology, clonal evolution, and drug sensitivity or resistance of chronic lymphocytic leukemia (CLL). By performing whole exome sequencing, we present the genetic landscape of primary tumors from TCL1 mice and of TCL1 tumors serially transplanted into wild-type recipients to mimic clonal evolution. We show that similar to CLL patients, mutations in mice are frequently subclonal and heterogenous among different primary TCL1 mice. We further describe that this molecular heterogeneity mirrors heterogenous disease characteristics such as organ infiltration or CLL dependent T cell skewing. Similar to human CLL, we further observed the occurrence of novel mutations and structural variations during clonal evolution and found plasticity in the expansion of B cell receptor specific subclones. Thus, our results uncover that the genetic complexity, pathway dependence and clonal dynamics in mouse CLL are in relevant agreement to human CLL, and they are important to consider in future research using the TCL1 mouse for studying CLL.

## Introduction

Recent high throughput sequencing approaches revealed complex genetic landscapes in multiple human cancer entities [1]. In the two largest studies, a panel of 38 genes was found to be recurrently mutated in chronic lymphocytic leukemia (CLL) (most genes at frequencies of < 10%), with *SF3B1, TP53,* and *NOTCH1* being among the predominantly mutated genes [2, 3]. The *Tcl1* transgenic (TCL1) mouse is the most widely used model to study biology, pathophysiology and treatment response of CLL, however, information on mutations in this mouse is still absent [4–7]. Importantly, primary tumors from backcrossed leukemic TCL1 mice can be transplanted onto congenic wild-type recipient mice, allowing the investigation of clonal tumor evolution in a fully immune competent environment [8–10]. Leukemic clones from TCL1 mice showed primarily unmutated IGHV genes (UM), making this mouse an ideal model to study the aggressive form of CLL [5, 11]. In addition, TCL1 mice also exhibit typical T cell skewing associated with CLL development, which is the emergence of TCR-Vβ specific T cell clones and a shift towards effector memory T cells [8, 12–14]. It is currently unknown whether specified IGHV rearrangement on the background of TCL1 overexpression is sufficient for leukemogenesis or if additional mutations are acquired during the preleukemic latency period.

To decipher the genetic landscape in TCL1 mice, we performed whole exome sequencing (WES) of highly purified primary TCL1 tumors from leukemic TCL1 mice and of tumors arising upon transplantation into congenic, immune competent wild-type animals. We found clonal and subclonal mutations, as well as recurrent structural variations (SV) and describe acquisition of mutations/SV and outgrowth of subclones during transplantation of tumors. Similar to human CLL, our results reveal a high genetic complexity of TCL1-driven mouse CLL and uncover high molecular dynamics during clonal evolution.

## Methods

### Mice

Genotyping of TCL1 transgenic mice (C57BL/6J) was performed as previously described [4]. Experiments were performed under approval from the Austrian animal ethics committee (BMWF 66.012/0009-II/3b/2012 and 20901-TGV/52/11-2012). Mice were followed for signs of illness and killed when moribund by CO_2_ suffocation at humane endpoints. Intraperitoneal transfer of murine tumor cells from spleens into two months old congenic immune competent wild-type C57BL/6J recipient mice was performed as previously described [8].

### Source of DNA for sequencing and DNA library preparation

CD5/CD19 positive CLL cells were sorted (FACS Aria III, Becton Dickinson) from splenocytes either of original TCL1 mice or of WT mice intraperitoneally transplanted with CLL cells. Germline DNA was extracted from either tail tips, ear clips, sorted CD5/CD19 negative splenocytes or sorted hepatocytes using DNeasy Blood and Tissue kit including RNaseA digestion (Qiagen, West Sussex, UK). DNA was subjected to whole exome library generation (Agilent SureSelect Mouse All Exon Kit; 49.6 megabases), which were sequenced on the Illumina platform HighSeq2500 using 100 bp paired-end reads with a mean coverage depth of 100x. WES data were deposited in Sequence Read Archive, NCBI, NIH (SRA accession code SRP150049).

## Results

### TCL1 mice have BCR-specific subclones

**Fig. 1:**
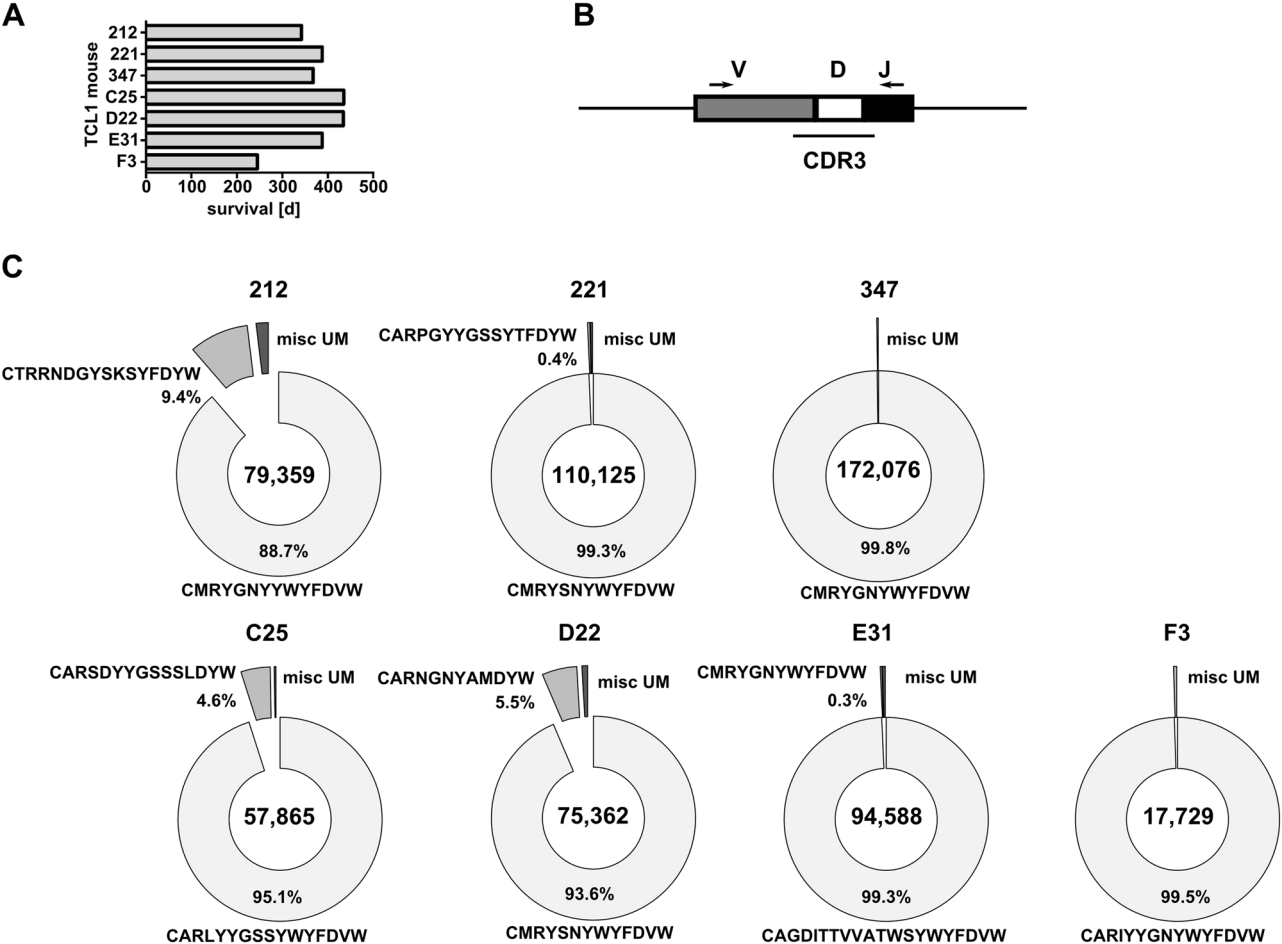
BCR analysis of TCL1 tumors. **a** Overall survival of seven primary TCL1 mice. **b** Schematic representation of the rearranged IgH and primers (arrows) used for PCR amplification and sequencing. **c** Pie charts show the frequency of specific CDR3 VDJ-H usage of seven primary TCL1 tumors. Segment size corresponds to relative occurrence of a particular VDJ-H sequence. The translated CDR3 amino acid sequences of the major and second major clone exceeding 0.3% frequency are depicted next to each segment. The remaining unmutated clones are summarized as miscellaneous unmutated BCRs (misc UM). Numbers in the center of the pie show the total number of sequences analyzed and numbers next to each CDR3 amino acid sequence indicates the percentage of the respective BCR clone

For our analysis, we used a cohort of seven TCL1 mice that had developed high tumor loads of CD5/CD19 double positive CLL cells (mean 86.9% ± 6.1%; Supplement Table 1). The mice were sacrificed at humane endpoints with an overall survival between 245 and 434 days, which is comparable to previously reported data on the TCL1 mouse model on a mixed genetic background [4, 8] (Fig. 1a). We first performed next generation sequencing (NGS) of rearranged IgH-VDJ sequences from genomic DNA of highly purified CLL cells (purity > 98.5%; Fig. 1b and Supplement Table 2) to determine the frequencies of independent B cell clones. In all primary TCL1 tumors analyzed, we observed one predominant unmutated BCR clone with a maximum productive frequency from 88.7 to 99.5%. However, we also observed several minor unmutated clones at frequencies of up to 9.4%, revealing the presence of BCR-specific subclones (Fig. 1c, Supplement Table 3). Interestingly, the TCL1 mice D22 and 221 share an identical predominant BCR CDR3 clone (CMRYSNYWYFDVW; 93.6% and 99.3%, respectively; (Fig. 1c, Supplement Table 3) supporting a concept of BCR stereotypy also in mice as was reported previously [5].

### WES reveals tumor heterogeneity in TCL1 mice

**Fig. 2:**
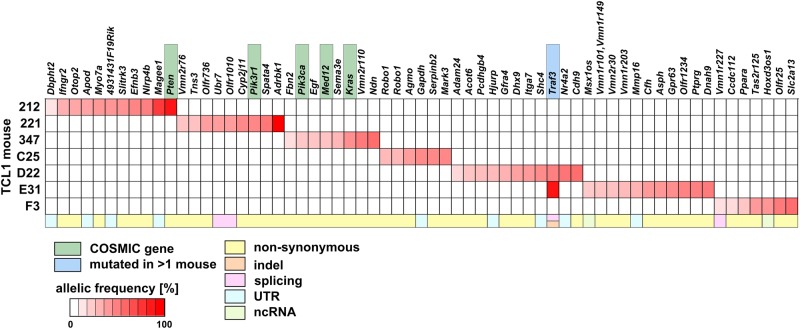
Clonal and subclonal mutations in TCL1 tumors. Heat map of all somatic mutations (non-synonymous SNVs, indels, splicing sites, UTRs, ncRNAs) found by WES. Color spectrum (white to red) corresponds to allelic frequencies of mutations. Green box: mutated COSMIC-CGC genes; blue box: genes that are mutated in more than one primary TCL1 mouse

We performed WES on purified CLL cells as well as on matched germline samples with an average sequencing depth of ~100x. We used the variant caller VarScan2 [15] to detect somatic variations present at allelic frequencies of ≥ 10% in CLL cells. Overall, we identified a total of 76 somatic mutations in seven TCL1 mice. This corresponds to a mutation rate of 0.2 ± 0.05 per megabase, which is similar to the mutation rate reported for human CLL (0.6 ± 0.28 per megabase with a range from 0.03 to 2.3) [2]. From these 76 somatic mutations, 49 (including 1 indel) affected protein coding sequences, nine affected 5′ or 3′ untranslated regions (UTR), four interfered with RNA splicing and two mutations affected exonic non-coding RNA (Fig. 2, Supplement Table 4). We detected only 12 exonic, synonymous mutations, pointing to non-random selection of non-synonymously mutated clones during disease development. Five non-synonymous mutations were located in genes described as tumor drivers in the COSMIC Cancer Gene Census (CGC) database [16], namely *Pten, Pik3r1, Pik3ca, Med12,* and *Kras*. In addition, we found two different *Robo1* mutations in mouse C25 and two different *Traf3* mutations in two different TCL1 mice (Fig. 2), one non-frameshift insertion of a leucine encoding triplet near the C-terminus of TRAF3 (mouse E31) and one affecting splicing of exons 5 and 6 (mouse D22; Supplement Table 4). Overall, the mutations we identified had allelic frequencies between 10.8 and 100%, indicating presence of clonal mutations but also heterogeneous cancer subclones in TCL1 mice. Of note, from our set of 76 somatic mutations, only two genes had previously been described as drivers in CLL (*Med12* and *Kras*). *Traf3* mutations had been discerned in CLL, but had not reached significance in regard to the detection of recurrence in those databases [2]. Mutations in genes most commonly mutated in human CLL samples such as *ATM, TP53,* or *NOTCH1* were not detected in our cohort of seven TCL1 mice.

### TCL1 mice show heterogeneous disease characteristics

**Fig. 3:**
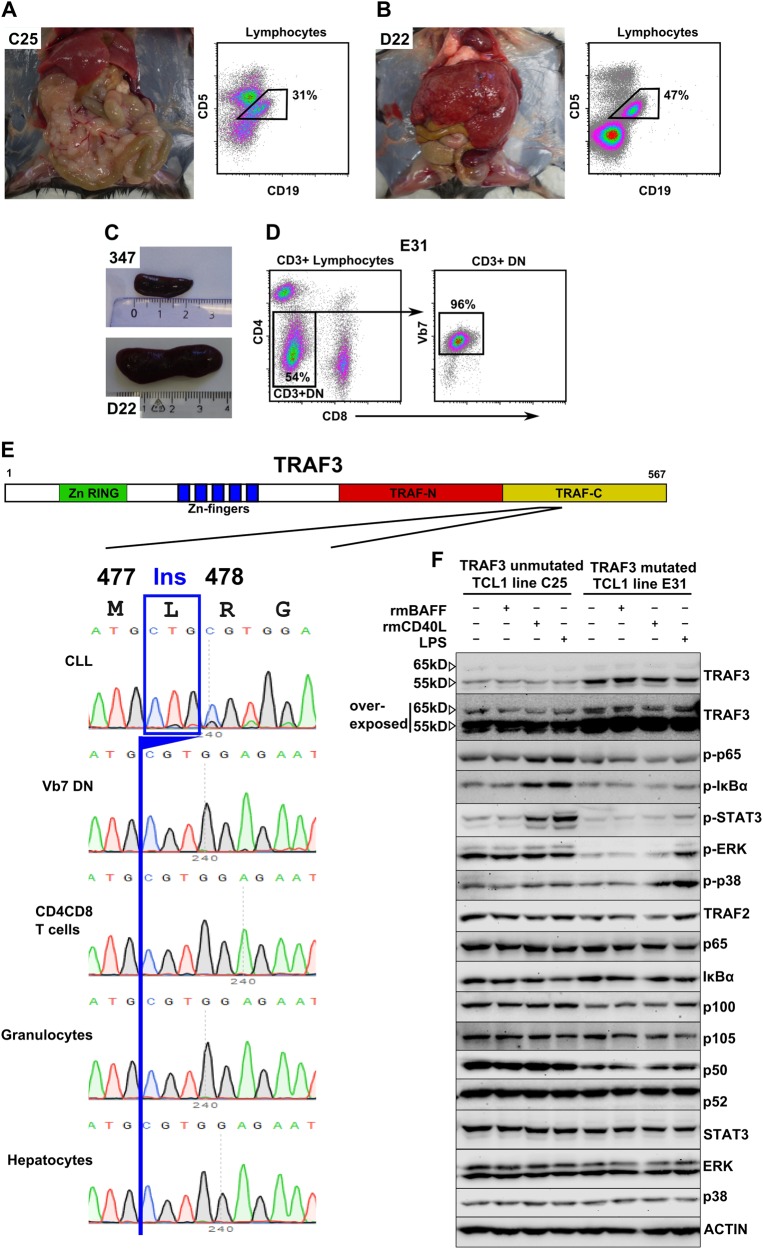
Disease characteristics of TCL1 mice. Infiltration of mesenteric lymph nodes (**a**) and liver (**b**) of TCL1 mice C25 and D22 with CD5/CD19 positive CLL cells. FACS plots show CD5/CD19 stains of cell suspensions of the respective organ pregated for lymphocytes. **c** Spleen size variability shown by two spleens from mouse 347 and D22. **d** Skewing towards Vβ7^+^DN T cells in TCL1 mouse E31. FACS plots show CD4/CD8 distribution in CD3^+^ cells (left) and Vβ7 usage in DN CD3^+^ cells (right). **e** Sanger sequencing of *Traf3* on DNA from the respective sorted splenocyte subset and germline (CD5CD19 negative hepatocytes) control from E31. Insertion (Ins) of leucine (L) at position 477 is indicated in blue. Protein domains of TRAF3 are depicted on top (adapted from Xie [36]. **f** Immunoblots from lysates of leukemic cells from CLL line C25 (*Traf3* unmutated) and line E31 (*Traf3* mutated) upon stimulation with the respective agents. Full length (~62 kD) and a shorter splice variant (~55 kD) are marked with arrows. (rm: recombinant mouse; p- means phosphorylated protein). (See also Supplement Figure S1 for detailed information)

Aside from high molecular heterogeneity, we also observed diverse disease characteristics in our TCL1 mouse cohort, which is in line with previous reports [10]. We noticed exceptionally high infiltration of mesenteric lymph nodes in TCL1 mouse C25 (Fig. 3a) and liver infiltration in TCL1 mouse D22 (Fig. 3b, Supplement Table 5). Spleen weight and size were very variable, ranging from 0.5 g/22 mm (TCL1 347) to 1.9 g/38 mm (TCL1 D22; Fig. 3c), which is comparable to published data on TCL1 mice [12]. Apart from the typical skewing towards memory T cells in TCL1 mice [8] and in line with our previous observation [12] we observed overrepresented CD4/CD8 double negative T cells (DN T cells) in splenocytes from TCL1 mouse E31, with 96% of them expressing the NKT cell specific TCR Vβ7 (DN T cells E31: 54.0%; mean TCL1 mice: 12.7%; SD ± 20.6; mean WT mice: 6.4% SD ± 0.8; Fig. 3d, Supplement Table 6). As TCL1 mouse E31 harbored a clonal *Traf3* mutation (leucine-insertion at aa478) and TRAF3 was recently shown to be important for NKT cell development [17], we suspected that this mutation might also be found in the expanded TCR-Vβ7 DN T cell population, driving its expansion. However, using Sanger sequencing of sorted T cells, we found that the *Traf3* mutation was confined to CLL cells and was not present in TCR-Vβ7 DN T cells or other blood cells sorted from the mouse (Fig. 3e). Nonetheless, we found that the *Traf3* mutation in E31 led to increased protein levels of TRAF3 in the leukemic cells compared to a *Traf3* non-mutated tumor (line C25). Concurrent with TRAF3 described as a signaling inhibitor [18, 19], E31 showed a lower NFkB signaling (phosphorylation of p65 and IkBα) and lower ERK and STAT3 phosphorylation (Fig. 3f). In contrast, leukemic cells from D22 (*Traf3* splicing mutation) revealed decreased full-length isoforms of TRAF3 protein and correspondingly, increased NFkB signaling. However, ERK and STAT3 phosphorylation was also reduced in D22 (Supplement Fig. 1).

### BCR-specific CLL clones can differentially expand and obscure clonal evolution and clonal switching upon serial transplantation

**Fig. 4:**
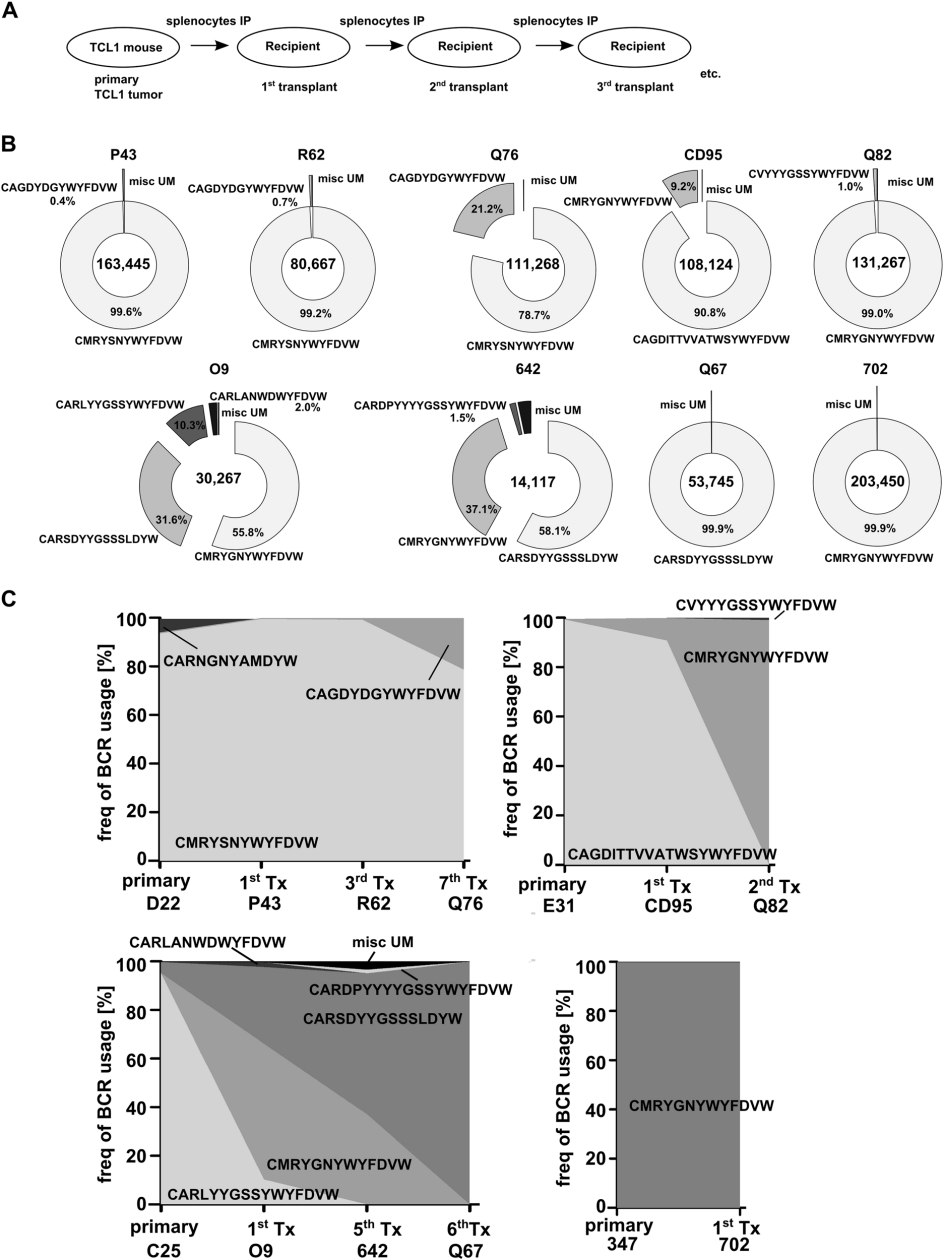
BCR analysis of transplanted TCL1 tumors. **a** Transplantation scheme of TCL1 tumors. **b** Pie charts show the frequency of specific CDR3 VDJ-H usage of nine transplanted TCL1 tumors as in Fig. 1c. **c** Diagrams show the dynamics of BCR-specific clones during transplantations. (Tx: transplant)

In previous experiments it was shown that tumors from TCL1 mice are transplantable into congenic immune competent wild-type recipient mice [8]. This transplantation results in a shortened preleukemic phase and in accelerated tumor development in recipients. As we detected minor BCR-specific subclones in primary TCL1 tumors, we first wanted to analyze clonal dynamics of B cell receptor usage of the individual tumors upon serial transplantation of tumor cells into WT recipients (Fig. 4a). Therefore, we performed IGHV characterization of sorted CLL cells on four randomly selected lines of serial transplants (1st, 3rd, and 7th transplantation from primary tumor D22, 1st, 5th, and 6th transplantation from primary tumor C25, 1st and 2nd transplantation from primary tumor E31 and 1st transplantation from primary tumor 347) isolated from spleens of visibly ill recipients and compared BCR usage with matched original primary tumors. In all nine transplanted tumors analyzed (P43, R62, Q76, O9, 642, Q67, CD95, Q82, 702) we detected one predominant unmutated BCR clone (maximum productive frequencies: from 55.8 to 99.9%) with several minor unmutated clones (highest frequency of 31.6%; Fig. 4b, Supplement Table 3). In transplants from TCL1 mouse D22 and 347, we observed a preserved major BCR clone, with expansion of one minor BCR clone from 0.5% in the primary tumor D22 to 21.3% in the 7th transplant (Fig. 4c, Supplement Table 3). Surprisingly, in transplants from primary tumors C25 and E31, we observed dramatic shifts from one major BCR clone to a completely different clone (Fig. 4d, Supplement Table 3). These clonal dynamics are also recapitulated when tumors are transplanted in parallel into two recipients, leading to outgrowth of different clones in some cases (Supplement Fig. 2). This demonstrates an evolutionary advantage of initially minor clones in the competitive repopulation of the transplant environment. Importantly, we also found subtle differences in organ specific distributions of BCR-specific subclones (Supplement Fig. 3).

### WES shows clonal evolution upon transplantation of tumors

**Fig. 5:**
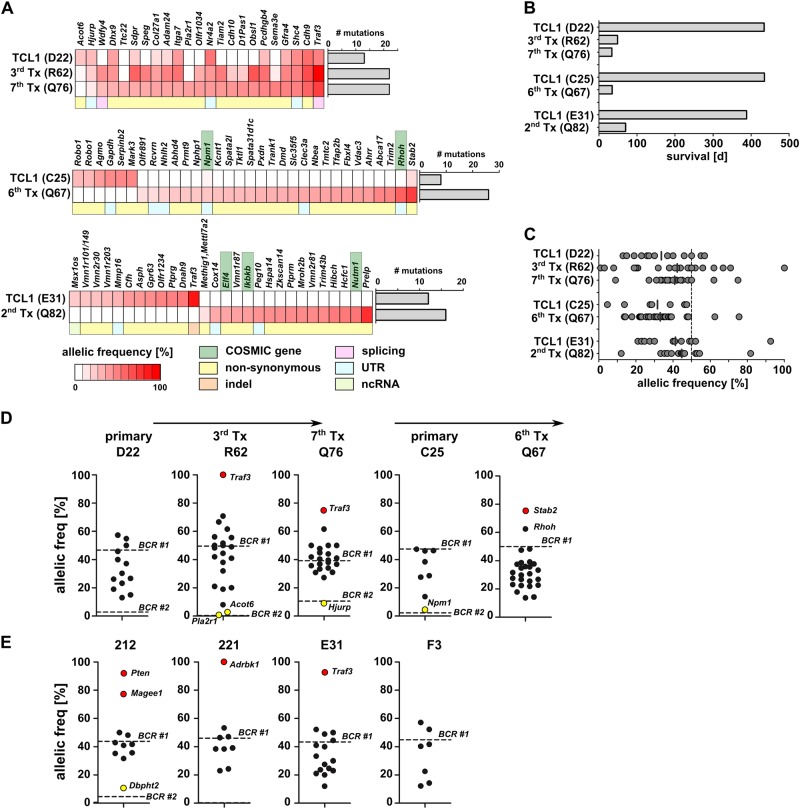
Clonal and subclonal mutations in serially transplanted TCL1 tumors. **a** Heat map of all functional somatic mutations from three tumor lines found by WES. Color spectrum (white to red) corresponds to allelic frequencies of mutations. The total numbers of mutations are depicted as bar graphs next to the heat maps. **b** Graph shows survival of primary TCL1 mice and of tumor-transplant recipients (d: days). **c** Allelic frequencies of all functional mutations are shown as dot plots. **d** Allelic frequencies of all functional somatic mutations in the two analyzed transplant lines (D22 and C25) are shown as dot plots. Within each graph, the horizontal dotted lines indicate expected allelic frequencies of clonal heterozygous somatic mutations within the respective BCR-specific subclones (major clone: BCR #1; most frequent minor clone: BCR #2). Red dots represent mutated genes with allelic frequencies ≥ 75%. Yellow dots represent mutated genes that roughly correspond to clonal heterozygous somatic mutations within BCR-specific subclone #2. **e** Same analysis as (**d**) with primary TCL1 mice

To assess whether the observed BCR dynamics upon tumor transplantation are also associated with clonal evolution of somatic mutations, we performed WES of serially transplanted tumors R62, Q76, Q67, and Q82 and compared mutation profiles with matched original primary tumors. In transplants descending from TCL1 mouse D22, we found the occurrence of additional mutations with increasing transplantation rounds, indicating significant clonal evolution within the original BCR clone during tumor growth in recipients (Fig. 5). By contrast, in the 6th transplant Q67 from TCL1 mouse C25 only two of eight original mutations were discerned, while 24 novel mutations were detected in the transplant (Fig. 5). This finding was not unexpected as this transplant had also shown a severe change in BCR clonality (Fig. 4d), suggesting the observed complete change in clonal architecture was due to the switch of individual clones observed on the level of BCR rearrangement. Similarly, in transplant Q82 (2nd transplant from TCL1 mouse E31), the mutation profile changed completely and none of the mutations from the primary tumor could be detected in the transplant, where only novel gene mutations were discerned (Fig. 5a, Supplement Table 4). Of note, some of the conserved mutations were identified only upon selective manual search for primary tumor- or transplant-specific mutations, leading to annotation of additional mutations with low allelic frequencies or low read counts in primary tumors compared to results from Fig. 2 (Supplement Table 4). Although the primary tumor in TCL1 mouse E31 had a clonal *Traf3* mutation, this mutation was not detectable in the tumor from recipient Q82 (Supplement Table 4). Notably, also the severe T cell skewing described for TCL1 mouse E31 was absent from wild-type recipient Q82 (5.2% DN T cells). In all three transplantation lines, we observed an increase in the total number of mutations upon transplantation (D22-Q76: from 13 to 22 mutations; C25-Q67: 8 to 26 mutations; E31-Q82: 12 to 16 mutations; Fig. 5a). We also noticed a shortened overall survival (Fig. 5b) and a slight increase of mean allelic frequencies upon serial transplantation (Fig. 5c). Analysis of allelic frequencies of mutations in regard to BCR clonality revealed that some somatic mutations are likely confined to distinct BCR-specific subclones (e.g., mutated 5′UTR of *Hjurp* in Q76 or 3′UTR of *Npm1* in C25), while most of the mutations are subclonal within the major BCR-specific CLL clone (Fig. 5d, e). In two transplantation lines (descending from C25 and E31) we observed the occurrence of new mutations in genes described as cancer drivers in COSMIC-CGC database [16], which are *Rhoh, Npm1, Elf4, Ikbkb, and Nutm1* (Fig. 5a).

**Fig. 6:**
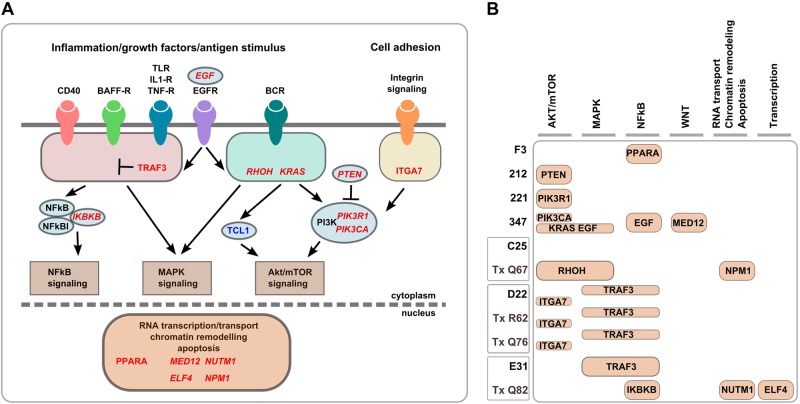
Biological pathways associated with non-synonymously mutated genes in TCL1 mice. **a** Mutated genes are shown in red, TCL1 in blue, COSMIC-CGC genes in italic. **b** Table showing mutated genes from (**a**) affecting different biological pathways in primary and transplanted TCL1 mice. Boxes on the *y*-axis represent sequential transplantation. Mapping of mutated genes to biological pathways was performed using Ingenuity Pathway Analysis tool, IPA (QIAGEN)

In addition to the annotation of single mutations, we mapped non-synonymously mutated genes to biological pathways, revealing BCR signaling, inflammatory stimuli, growth factors and integrin signaling to be implicated in CLL development of TCL1 mice and WT transplanted mice (Fig. 6a). Pathways affected in individual mouse tumors by these mutated genes are shown in Fig. 6b. All mice, with the exception of TCL1 mouse C25 had at least one gene mutated in these pathways.

### Murine CLL shows evolution of recurrent structural chromosomal variations

**Fig. 7:**
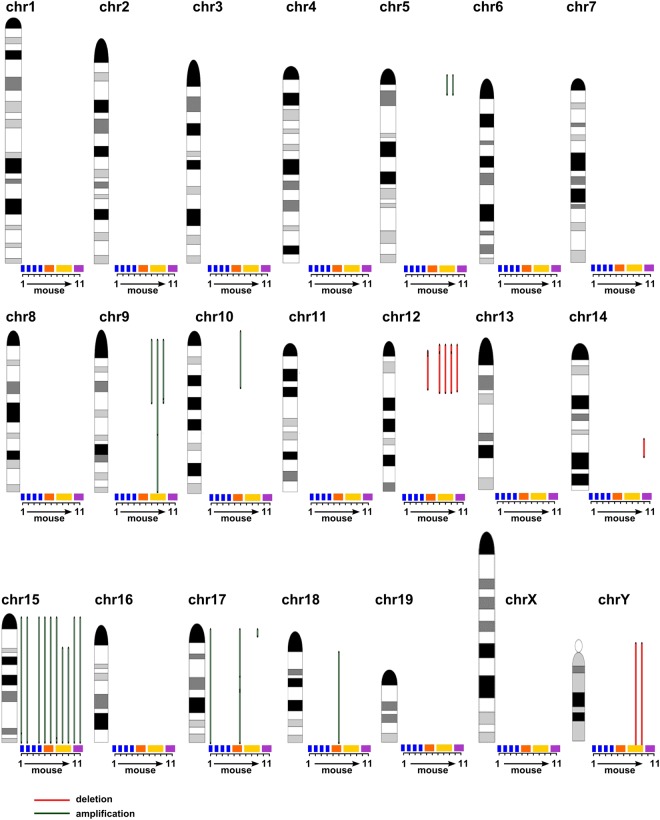
Large structural variations in TCL1 mice. Deletions (red) and amplifications (green) of genomic regions (>5 Mb SV from Supplement Table 7) of the respective chromosomes are indicated as bars for individual mice. Bar length corresponds to length of the SV. Order and sex (m/f) of mouse 1–11 on the *x*-axis: F3 (m), 212 (m), 347 (m), 221 (m), C25 (f), Q67 (f), D22 (m), R62 (m), Q76 (m), E31 (f), and Q82 (f). Colored horizontal bars above the *x*-axis represent individual primary TCL1 mice (blue) and individual transplantation lines (orange (C25, Q67), yellow (D22, R62, Q76), purple (E31, Q82)). Ideograms are adapted from Dr David Adler (University of Washington, Seattle, http://www.pathology.washington.edu/research/cytopages/idiograms/mouse/)

We screened the WES data for large structural chromosomal variations (SV). In all mice analyzed, we could detect several chromosomal deletions and amplifications (Fig. 7, Supplement Table 7 and 8). We found highly recurrent complete or partial trisomy 15 (10 of 11 mice) as well as deletions on chromosome 12q (5 of 11 mice; Fig. 7, Supplement Table 7 and 8). To determine which COSMIC-CGC cancer genes are affected in our mice by the deletions and amplifications of the respective chromosomes (Fig. 7) we used the NCBI homology maps. A list of all COSMIC-CGC cancer genes affected including the *Traf3* gene (which is monoallelically deleted in all *Traf3* mutated cases except for TCL1 mouse D22, whereby only mutated *Traf3* is expressed) on chromosome 12 are shown in Supplement Table 9. Furthermore, we could detect a subclonal translocation t(12:17) in sample Q67 (chr12:69701036; chr17:56593781; Supplement Table 7). Concurrent with our data from IGHV and mutation analyses, we not only observed the acquisition of additional SVs upon tumor transplantation (tumor line from D22: acquisition of SVs on chr5, chr9, chr15, chr17 and chrY), but also complete changes in SV patterns in those tumors in which we also noticed expansion of IGHV specific subclones upon transplantation (tumor line from C25: disappearance of del chr12 and gain of amplifications on chr10, chr17, chr18 and tumor line E31: disappearance of del chr12, del chr14 upon transplantation; Fig. 7, Supplement Table 7 and 8).

## Discussion

In this study we performed WES on primary CLL samples from TCL1 mice and found a striking genetic inter- and intratumoral heterogeneity, which is somewhat similar to human CLL and exceeds that described for other recently sequenced murine leukemia models [20]. This heterogeneity was not necessarily expected as expression of the strong oncogene TCL1 itself could suffice as possible driver for CLL in this mouse model. As may be expected, mutations complementing TCL1, a driver not universally accepted to be important in human CLL, were detected in genes that have not been described in human CLL. However, these mutations interestingly mapped to similar biological pathways, indicating similar dependency on core signals that contribute to CLL growth and survival when comparing human and murine CLL, which is in line with previous reports [7]. In primary TCL1 mice, these pathways primarily comprise BCR, growth factor and inflammatory signaling via MAPK, NFκB, and mTOR. Many mutated genes from transplanted tumors encode proteins important for cell adhesion and transcription, among them the COSMIC-CGC cancer drivers *Npm1*, *Nutm1,* and *Elf4* [21, 22]. Of note, in addition to a mutation in the cancer driver *Kras* [23] we detected two mutations in PI3K subunits *Pik3r1* and *Pik3ca* and a deactivating stop-gain mutation of tumor suppressor *Pten*, which regulates PI3K by dephosphorylating the lipid signaling intermediate PIP3 to PIP2 [24]. We also detected a mutation in *Rhoh* in a transplanted tumor, which was previously reported to be important for CLL development in TCL1 mice [25, 26]. As the only recurrently affected gene in our cohort, two mutations were observed in *Traf3*, which—in accordance with our data—was shown to be a negative regulator of NFκB and MAPK signaling [18, 19]. Interestingly, the *Traf3* mutations we identified had two opposing effects on NFkB and MAPK signaling. However, although *Traf3* has been described as a tumor suppressor and loss of *Traf3* was found in a subset of multiple myeloma patients [27, 28], increased levels of TRAF3 in *Traf3* transgenic mice also promoted autoimmunity, inflammation, and cancer [29].

Aside from somatic mutations, our data revealed many SVs. While human CLL frequently harbors deletion 11q, deletion 13q, deletion 17p and trisomy 12 [30], we recurrently found trisomy 15 and deletion 12q in mouse CLL. In this regard it is interesting to note that comparative genome mapping revealed partially conserved synteny between human chromosome 12 and mouse chromosome 15, suggesting that trisomy 15 in mouse might at least partially correspond to trisomy 12 in CLL patients [31]. Strikingly, although two of three tumor lines analyzed showed complete change in clonality in regard to IGHV and mutations and SVs upon transplantation (tumor lines from original tumors C25 and E31), these tumors shared a common trisomy 15, pointing to trisomy 15 being possibly acquired prior VDJ rearrangement, which could prime for CLL development. Interestingly, among the COSMIC-CGC driver genes, the protooncogene *Myc* is encoded on chr15, which is implicated in many hematological malignancies [32]. Deleted regions on 12q affected mostly genes located proximal to variable elements on the IgH locus (e.g., *Traf3*), suggesting that deletions were likely generated by illegitimate VDJ recombination [33]. This is particularly interesting, as it has been proposed that CLL in TCL1 mice could develop based on failure to successfully edit autoreactive BCR specificity by secondary VDJ recombination [34]. Hence, it is conceivable that collateral damage during primary or secondary VDJ recombination on the inactive allele could cause large deletions on chromosome 12q, which could thus be an initializing event for mouse CLL.

Importantly, our NGS analysis on IGHV expression in CLL tumors revealed a striking plasticity and clonal dynamics. In all cases, independent BCR-specific subclones could be detected, and more strikingly, upon transplantation of tumors, we observed some expansion of initial minor subclones in recipient mice. The existence of multiple productive rearrangements in CLL was recently also noticed in a subset of human CLL and concurring with our mouse data, the expansion of minor clones could also be observed in some of these patients during natural clonal evolution or treatment [35]. Hence, the TCL1 mouse model is ideal for investigating clonal dynamics by analyzing transplanted tumors in recipients with altered microenvironmental backgrounds or with a particular immune-compromisation to draw light on evolutionary pressures exerted by tumor-microenvironment and tumor-immune interactions and immune niches. In this regard, our data are also important to be considered for preclinical treatment studies on TCL1 mice, as clonal evolution, dynamics in subclonal heterogeneity and finally overall treatment responses will certainly depend on the particular mutation profile of the respective mouse tumor, particularly as even transplanted tumors descending from the same primary tumor can exhibit dramatically different genetic profiles and may even represent different individually derived clones.

## Electronic supplementary material

Supplementary methods

Supplement table legends

Supplement Figure Legends

Figure S1

Figure S2

Figure S3

table S1

table S2

table S3

table S4

table S5

table S6

table S7

table S8

table S9

protocol exchange
