# Supplementary material for: Exome sequencing of the TCL1 mouse model for CLL reveals genetic heterogeneity and dynamics during disease development
Source: Leukemia. 2018 Sep 27;33(4):957–68. doi: 10.1038/s41375-018-0260-4 (PMC6477797; doi:10.1038/s41375-018-0260-4)
Supplement: Supplementary file 1 — Supplementary methods [file 41375_2018_260_MOESM1_ESM.docx]

Supplemental methods

Mutation analysis

Sequencing reads were mapped to mouse reference genome (UCSC mm10) using Burrows-Wheeler Aligner with default settings (BWA-MEM v0.7.15).(1) Duplicate removal was performed with default parameters using PicardTools (v2.2.2, http://broadinstitute.github.io/picard/). Alignments were preprocessed including local realignment around indels and base quality recalibration was performed using Genome Analysis Tool Kit (GATKv3.5) (all with default parameters).(2) After mpileup file generation by samtools (v1.3.1) (parameter –B –q 1) (3), VarScan2 (v2.3.7) was used for somatic variant calling (–min-coverage-normal 5 –min-coverage-tumor 5 –min-var-freq 0.05 -somatic-p-value 0.05 –strand-filter 1) and filtering of high confidence calls was performed according to Basic Protocol 2 published by Koboldt et al. (4). Variants were annotated using ANNOVAR (version 2015Dec14) with COSMIC version 79 (cancer.sanger.ac.uk).(5) All programs were executed following the authors’ recommendations. Non-synonymous exonic SNVs, Indels, UTRs, ncRNA and splice-site variants were considered. All remaining mutations were individually checked for accuracy using Integrative Genomics Viewer (IGV).(6, 7) Mapping of mutated genes to biological pathways was performed using Ingenuity Pathway Analysis tool IPA (QIAGEN, IPA fall release September 30, 2017).

Structural Variants Analysis

All germline and tumor reads were trimmed for adapter sequence content using TrimGalore (version 0.4.3; <https://www.bioinformatics.babraham.ac.uk/projects/trim_galore/>) with arguments --paired --gzip. Trimmed reads were aligned to the reference genome mm10 with Hierarchical Indexing for Spliced Alignment of Transcripts (Hisat2, version 2.1.0)(8) using known mouse variant annotations from dbSNP (build 150) with argument --no-spliced-alignment --new-summary --threads 12. The tool Structural variant calling using Structural Variation and Indel Analysis by Assembly (SvABA, version 134; <https://github.com/walaj/svaba>) was used for calling structural variants and InDels on the bam files, with arguments --p 16, comparing each tumor sample to the corresponding germline sample, and default quality filtering arguments of --lod 8 --lod-dbsnp 5 --lod-somatic 2.5 --lod-somatic-dbsnp 4 --scale-errors 1. For Copy Number Variations (CNVs) detection, depth of coverage was calculated for each exome target region using Genome Analysis Toolkit (GATK, version 3.8.0) -T DepthOfCoverage -omitBaseOutput -omitLocusTable. Coverage data was then analysed using the R package ExomeCNV (version 1.4) using the Circular Binary Segmentation method (CBS) to combine genomic bins into regions of equal copy number and visualize the resulting calls in genomic context.

Immunofluorescence staining and flow cytometric analysis

White blood cells were incubated with directly conjugated monoclonal antibodies (mAbs). Fresh blood samples were stained prior to erythrocyte lysis using FACS lysing solution (Becton Dickinson) with the following mAbs: TCR-Vβ7 FITC (TCR-Vβ screening panel, #557004, BD Pharmingen), all Biolegend: CD3 PE, (#100206), CD4 PE-Cy5 (#100514), CD19 PE-Cy7 (#115520), CD8a Pacific Blue (#100725), CD3 AlexaFluor 700 (#100216), CD5 PE-Cy5 (#100610), CD8a PE-Cy7 (#100722), CD19 Pacific Blue (#115523). Data acquisition was performed on GalliosTM Flow Cytometer research system (Beckman Coulter) and data analysis was performed using Kaluza 1.3 Flow Cytometry Analysis Software (Beckman Coulter).

B cell receptor (BCR) sequencing

BCR sequencing from samples F3, 212, 221, C25, D22, E31, R62, Q76, Q67 was performed from the DNA of sorted CLL cells using the survey resolution ImmunoSEQ platform (Adaptive Biotechnologies, Seattle, WA). Bioinformatic and biostatistical analysis of productive clones was performed by immunoSEQ Analyzer 3.0.(9) As the observed low frequency of sequences corresponding to mutated BCRs could be based on PCR errors or deriving from residual healthy B cells, only sequences corresponding to unmutated BCRs were considered for pie chart analysis in Figure 1 and 4. Allele frequencies of BCRs as shown as horizontal dotted lines in Figure 5D are calculated as 50% of clonal BCR frequencies determined by amplicon sequencing. BCR sequencing from samples 347, P42, P43, O9, O11, 642, CD92, CD95, Q82, Q83, 702 and 703 was performed from the DNA of sorted CLL cells upon PCR-amplification of rearranged VDJ genes (IgH locus) using Phusion polymerase (Thermo Fisher Scientific, USA) and a mix of the following degenerated primers, which bind FR1 region of mouse-V elements and J-regions: RG1269 5’-SAGRTBCARCTKMARSAGYCWGGVSCT-3’; RG1270 5’-GARGTGMAGCTKGWDGAGWCTGGDGGA-3’; RG1271 5’-CTGMRGARACDGTGASHVDRGTBCCTK-3’. PCR products were reamplified using tagged primers RG1272 TCGTCGGCAGCGTCAGATGTGTATAAGAGACAG SAGRTBCARCTKMARSAGYCWGGVSCT RG1273 TCGTCGGCAGCGTCAGATGTGTATAAGAGACAG GARGTGMAGCTKGWDGAGWCTGGDGGA RG1274 GTCTCGTGGGCTCGGAGATGTGTATAAGAGACAG CTGMRGARACDGTGASHVDRGTBCCTK. Index PCR was done with Nextera XT index Kit (Illumina) to attach dual indices and Illumina sequencing adapters with the 2x KAPA HiFi Hot Start Polymerase (Roche). After each PCR reaction Agencourt AMPure XP beads (Beckman Coulter Genomics) were used to purify the PCR products. Quantitation was done by Qubit™ dsDNA HS Assay Kit (Thermo Fisher Scientific). Agilent Technologies 4200 TapeStation and the high sensitivity D1000 ScreenTape were used to determine the average fragment sizes. The libraries were diluted to a final concentration of 4 nM and all BCR mouse libraries were pooled. The pooled libraries were then denatured with 0.2 N NaOH and 8 pM were sequenced on Illumina MiSeqDx using reagents kit V3 in 300 bp paired-end run (Illumina). MiXCR software (version 2.1.8) and the reference library repseqio.v1.4 was used for analysis of the B cell receptor repertoire sequencing data (10). BCR sequencing data were deposited in Sequence Read Archive (SRA), NCBI, NIH (SRA accession code SRP150049). We uploaded a detailed protocol on Protocol Exchange (https://www.nature.com/protocolexchange/) with the title “NGS-based analysis of the mouse B-cell receptor repertoire”.

Sanger Sequencing of TRAF3

Exon 12 of mouse *Traf3* was PCR amplified from genomic DNA using primers RG1211 (5'-CTTGCCTATGACAGGCTTGCTGGAG-3') and RG1212 (5'GACCCCTGATCCATCAGCATAAGTGTCAC-3'). PCR products were gel purified (Qiagen, West Sussex, UK) and Sanger sequenced (Eurofins, Germany) using primer RG1215 (5'-GATGTTGAGTGTTCATGACATC-3').

Immunoblotting

Cells were treated for 2h using rmBaff (400 ng/ml, Biotechne), rmCD40L (400 ng/ml, Biotechne) + anti-HA Tag (250 ng, Biotechne) or LPS (10 µg/ml; Sigma) prior lysis in RIPA lysis buffer (0.5% Sodium Deoxycholate, 1 % NP-40, 0.1 % SDS, 50 mM Tris pH 8.0, 150 mM NaCl containing Proteinase Inhibitor Cocktail (Roche, Complete, EDTA-free, 04693132001), 5 mM NaF, 1 mM Na3VO4 for 20 min at 4°C. Lysates were centrifuged at 14,000 rpm for 10 min at 4°C, supernatants were collected and protein concentrations were measured by Bradford assay. 30 µg protein / lane were loaded onto a 9% SDS gel. For protein transfer PVDF membranes (Merck-Millipore, Darmstadt, DE) were used. All antibodies for western blotting were purchased from Cell Signaling Technology. The following antibodies were used: pp65 (clone 93H1), pIKBa (clone 14D4), TRAF2 (clone C192), pERK (#9101) pSTAT3 (#9131), Actin (#K18C11), pp38 (#9211), IKBa (clone L35A5), p65 (clone D14E12), STAT3 (clone 124H6), p38 (#9212), TRAF3 (#4729), p105/p50 (=NFkB1; #12540), p100/p52 (=NFkB2; #4882) and ERK (#9102). Analysis was performed with the LabWorks and the Image Studio Lite Version 5.2 Software.

Statistical analysis and mouse model

No statistical analyses for group comparisons or sample size estimates were used. No blinding or randomization was used. No cell lines or human samples were used for this study. Randomly selected TCL1 mice and TCL1-tumor transplanted recipient mice were selected for analysis. Inclusion criteria: mouse developed and died from CLL (presence of CD5CD19 positive cells in spleens from mice sacrificed at humane endpoints after clear signs of illness). As our study was pioneer work (no data on mutations in TCL1 mice were so far available), we chose a small mouse cohort (7 randomly selected primary TCL1 mice and 4 transplanted tumors) for a pilot study to elucidate non-synonymous mutations by whole exome sequencing, without calculating significance of mutations.

References

1. Li H, Durbin R. Fast and accurate long-read alignment with Burrows-Wheeler transform. Bioinformatics. 2010;26(5):589-95.

2. McKenna A, Hanna M, Banks E, Sivachenko A, Cibulskis K, Kernytsky A, et al. The Genome Analysis Toolkit: a MapReduce framework for analyzing next-generation DNA sequencing data. Genome Res. 2010;20(9):1297-303.

3. Li H, Handsaker B, Wysoker A, Fennell T, Ruan J, Homer N, et al. The Sequence Alignment/Map format and SAMtools. Bioinformatics. 2009;25(16):2078-9.

4. Koboldt DC, Larson DE, Wilson RK. Using VarScan 2 for Germline Variant Calling and Somatic Mutation Detection. CurrProtocBioinformatics. 2013;44:15-7.

5. Wang K, Li M, Hakonarson H. ANNOVAR: functional annotation of genetic variants from high-throughput sequencing data. Nucleic Acids Res. 2010;38(16):e164.

6. Robinson JT, Thorvaldsdottir H, Winckler W, Guttman M, Lander ES, Getz G, et al. Integrative genomics viewer. NatBiotechnol. 2011;29(1):24-6.

7. Thorvaldsdottir H, Robinson JT, Mesirov JP. Integrative Genomics Viewer (IGV): high-performance genomics data visualization and exploration. BriefBioinform. 2013;14(2):178-92.

8. Kim D, Langmead B, Salzberg SL. HISAT: a fast spliced aligner with low memory requirements. Nat Methods. 2015;12(4):357-60.

9. Carlson CS, Emerson RO, Sherwood AM, Desmarais C, Chung MW, Parsons JM, et al. Using synthetic templates to design an unbiased multiplex PCR assay. NatCommun. 2013;4:2680.

10. Bolotin DA, Poslavsky S, Mitrophanov I, Shugay M, Mamedov IZ, Putintseva EV, et al. MiXCR: software for comprehensive adaptive immunity profiling. Nat Methods. 2015;12(5):380-1.
