# Supplementary material for: Exome sequencing of the TCL1 mouse model for CLL reveals genetic heterogeneity and dynamics during disease development
Source: Leukemia. 2018 Sep 27;33(4):957–68. doi: 10.1038/s41375-018-0260-4 (PMC6477797; doi:10.1038/s41375-018-0260-4)
Supplement: Supplementary file 2 — Supplement table legends [file 41375_2018_260_MOESM2_ESM.docx]

Supplementary Information

Supplement Table 1: Tumor load measurements of fresh blood and thawed samples

Supplement Table 2: Purity of sorted CLL cells

Supplement Table 3: BCR receptor analysis of sorted CLL cells

Supplement Table 4: Mutated genes found by WES

Supplement Table 5: Mouse characteristics

Supplement Table 6: T cell subsets

Supplement Table 7: Copy number variations

Supplement Table 8: Structural variations for Fig 7

Supplement Table 9: Synteny of mouse and human chromosomes of Cosmic Cancer genes (incl. Traf3)

Protocol Exchange: Detailed protocol for NGS-based analysis of the mouse B-cell receptor repertoire.
