# Supplementary material for: Exome sequencing of the TCL1 mouse model for CLL reveals genetic heterogeneity and dynamics during disease development
Source: Leukemia. 2018 Sep 27;33(4):957–68. doi: 10.1038/s41375-018-0260-4 (PMC6477797; doi:10.1038/s41375-018-0260-4)
Supplement: Supplementary file 3 — Supplement Figure Legends [file 41375_2018_260_MOESM3_ESM.docx]

Supplement figure legends:

Supplement Figure S1. Signaling in Traf3 mutant D22 cells. (A) Mutation of G>A at splice donor site of intron 5 from mouse D22 is shown. Domains of Traf3 according to [www.uniprot.org](http://www.uniprot.org). Amino acid positions are indicated. (B) Schematic representation of NFκB, STAT3 and MKK signaling. All proteins detected by immunoblotting are schematically depicted. (C) Immunoblots from lysates of leukemic cells descending from CLL lines C25 (Traf3 unmutated) and D22 (Traf3 mutated) upon stimulation with the respective agents. Full length (at around 62 kD) and a shorter splice variant (at around 55 kD) are marked with arrows. ‘p-‘ means phosphorylated protein.

Supplement Figure S2. BCR heterogeneity upon transplantation. Pie charts show the frequency of specific CDR3 VDJ-H usage of four primary TCL1 tumors (D22, C25, 347, E31) serially transplanted in parallel into two WT recipient mice.

Supplement Figure S3. BCR analysis in different organs. The organ specific frequencies of specific CDR3 VDJ-H usage in 3 primary and 4 transplanted tumors are shown. Abbreviations: SPL: spleen; LIV: liver; BM: bone marrow; LNP: peripheral lymph nodes; PC: peritoneal cavity.
