# Supplementary figures and images for: Exome sequencing of the TCL1 mouse model for CLL reveals genetic heterogeneity and dynamics during disease development
Source: Leukemia. 2018 Sep 27;33(4):957–68. doi: 10.1038/s41375-018-0260-4 (PMC6477797; doi:10.1038/s41375-018-0260-4)

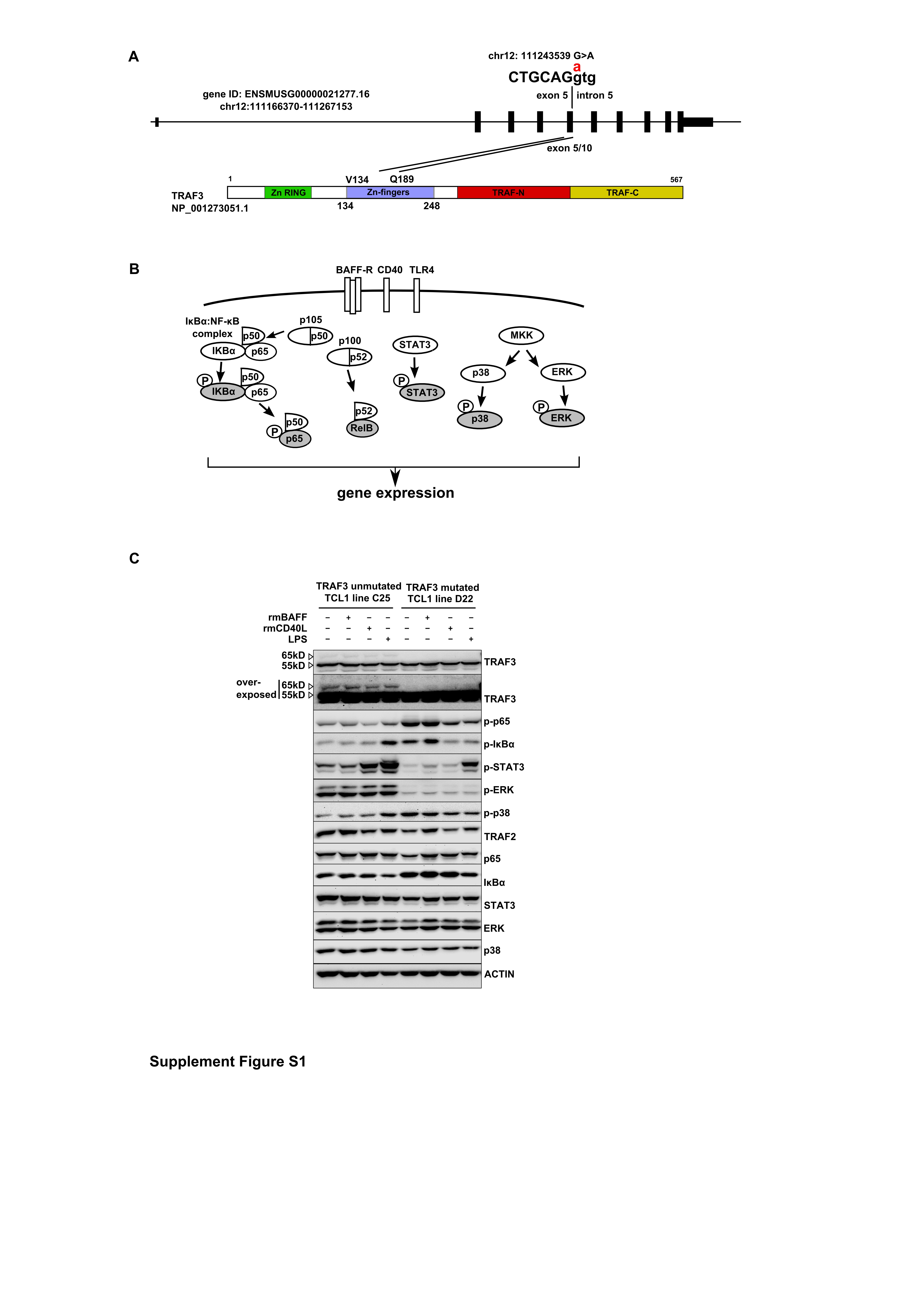

Supplement: Supplementary file 4 — Figure S1 [file 41375_2018_260_MOESM4_ESM.jpg]

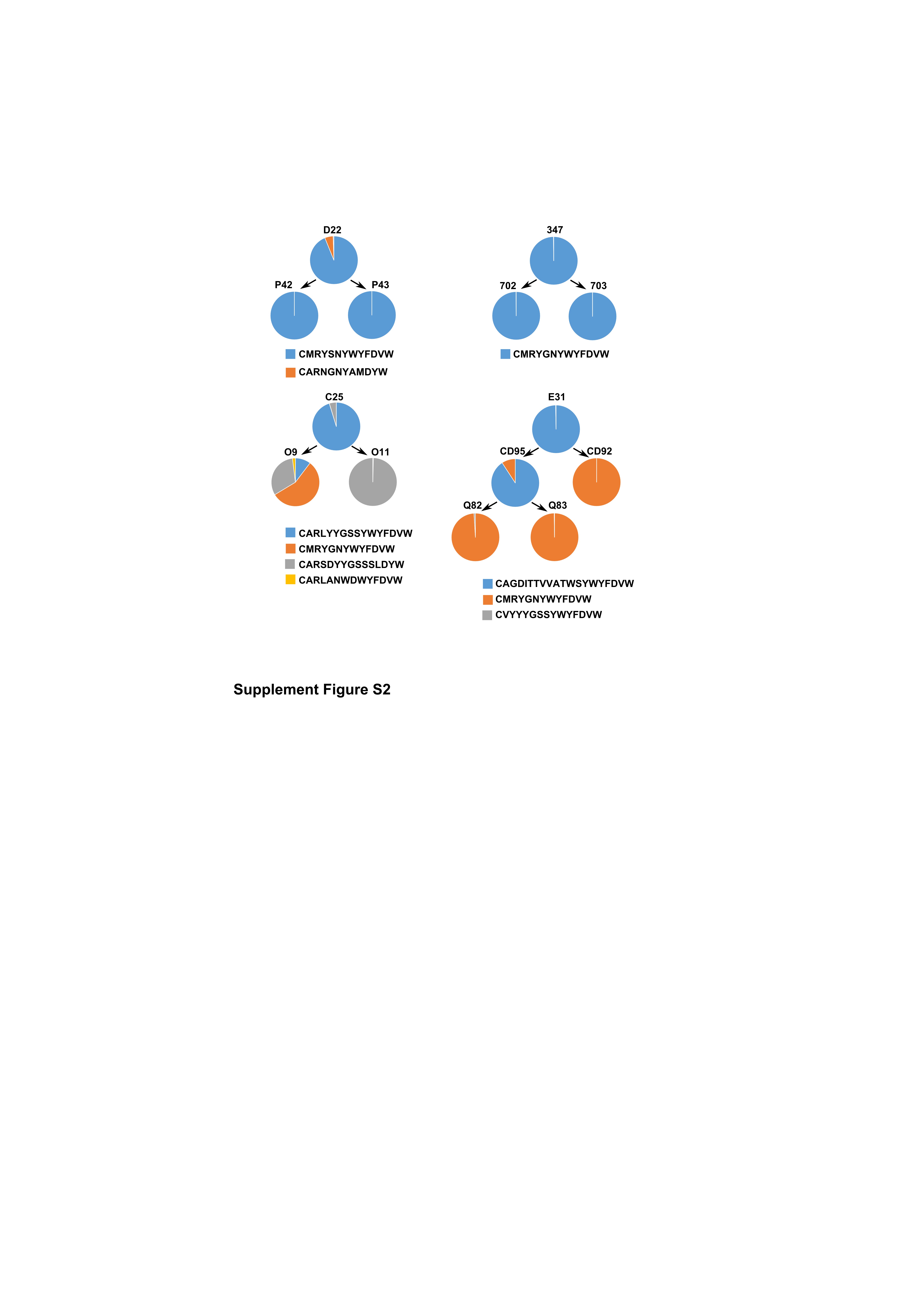

Supplement: Supplementary file 5 — Figure S2 [file 41375_2018_260_MOESM5_ESM.jpg]

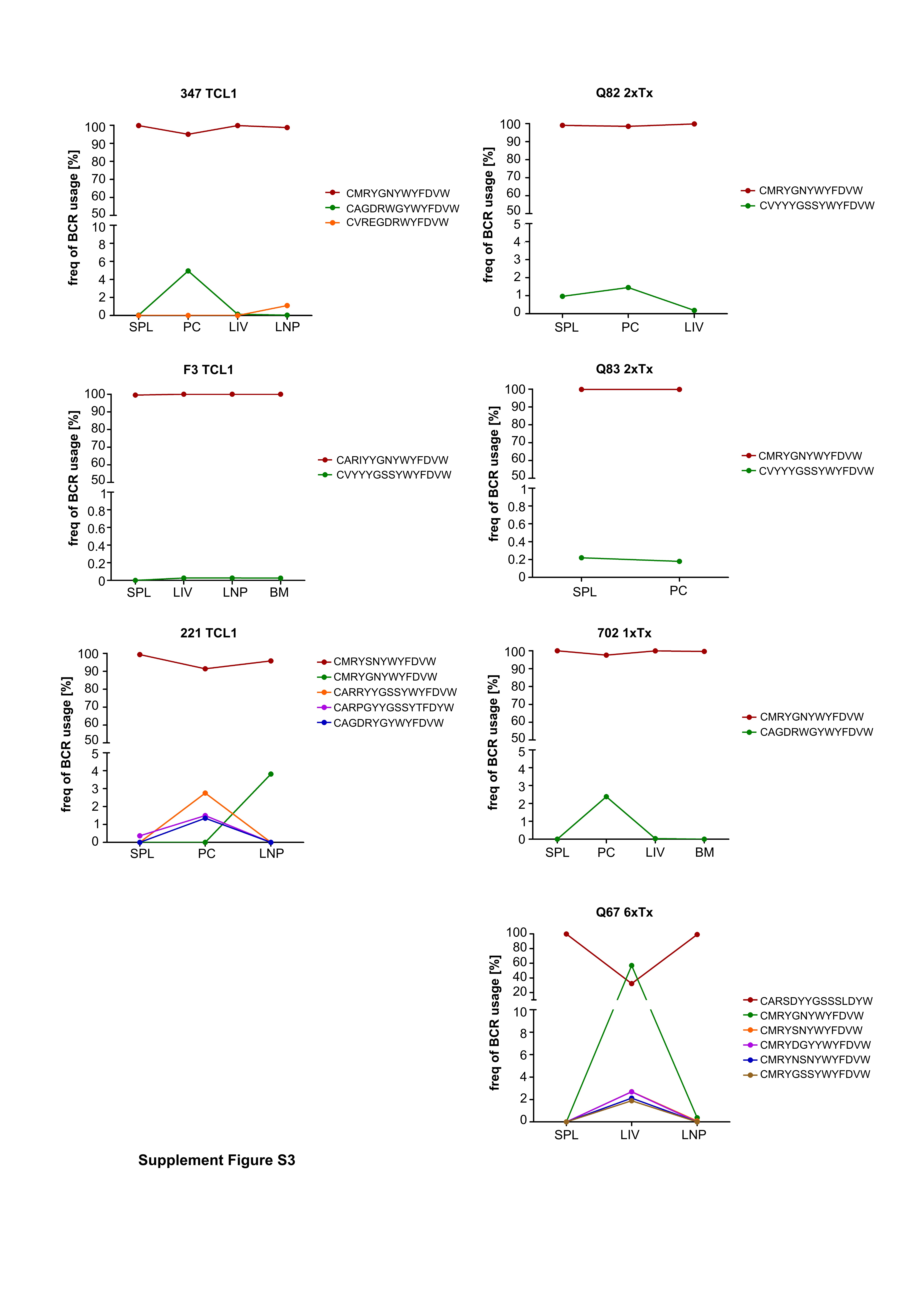

Supplement: Supplementary file 6 — Figure S3 [file 41375_2018_260_MOESM6_ESM.jpg]
