# Supplementary material for: Exome sequencing of the TCL1 mouse model for CLL reveals genetic heterogeneity and dynamics during disease development
Source: Leukemia. 2018 Sep 27;33(4):957–68. doi: 10.1038/s41375-018-0260-4 (PMC6477797; doi:10.1038/s41375-018-0260-4)
Supplement: Supplementary file 9 — table S3 [file 41375_2018_260_MOESM9_ESM.pdf]

sample_name	productive_frequency	templates	amino_acid
TCL_212_1	88.663	70362	CMRYGNYYWYFDVW
TCL_212_1	9.420	7476	CTRRNDGYSKSYFDYW
TCL_212_1	1.072	851	CARRYYGSSYWYFDVW
TCL_212_1	0.287	228	CARSYYDYVYYYAMDYW
TCL_212_1	0.141	112	CMRYSNYWYFDVW
TCL_212_1	0.134	106	CMRYSSYWYFDVW
TCL_212_1	0.073	58	CAGDYDGYWYFDVW
TCL_212_1	0.050	40	CMRYGSSYWYFDVW
TCL_212_1	0.005	4	CMRYGNYYWYFDVW
TCL_212_1	0.005	4	CAGDSDGYWYFDVW
TCL_212_1	0.004	3	CARDDYDGRGFAYW
TCL_212_1	0.004	3	CARLFTTVVDYW
TCL_212_1	0.004	3	CARRYYYGSSYYFDYW
TCL_212_1	0.003	2	CARYYSNYWYFDVW
TCL_212_1	0.003	2	CAGPGLPYYYW
TCL_212_1	0.003	2	CARDDDGYLWYFDVW
TCL_212_1	0.003	2	CSYCEDFAYW
TCL_212_1	0.003	2	CARMRYDYAWFAYW
TCL_212_1	0.003	2	CTGWGFDYW
TCL_212_1	0.003	2	CMRYGNYYWYFDVW
TCL_212_1	0.003	2	CMRYGNYYWYFDVW
TCL_212_1	0.003	2	CMRYGNYYWYFDVW
TCL_212_1	0.003	2	CMRYGNYYWYFDVW
TCL_212_1	0.003	2	CMRYGNYYWYFDVW
TCL_212_1	0.003	2	CMRYGNYYWYFDVW
TCL_212_1	0.003	2	CMRYGNYYWYFDVW
TCL_212_1	0.003	2	CMRYGNYYWYFDVW
TCL_212_1	0.003	2	CMRYGNYYWYFDVW
TCL_212_1	0.003	2	CMRYGNYYWYFDVW
TCL_212_1	0.003	2	CMRYGNYYWYFDVW
TCL_212_1	0.003	2	CMRYGNYYWYFDVW
TCL_212_1	0.003	2	CTRRNDGYSKSYFDYW
TCL_212_1	0.003	2	CMRYGNYYWYFDVW
TCL_212_1	0.003	2	CARPVGNAMDYW
TCL_212_1	0.003	2	CARYVYYGSSSYAMDYW
TCL_212_1	0.001	1	CMANHVTVWYFDVW
TCL_212_1	0.001	1	CARSGYITKGFAYW
TCL_212_1	0.001	1	EDFAYW
TCL_212_1	0.001	1	CARGMVTRAMDYW
TCL_212_1	0.001	1	CHFSPGRLRYFDVW
TCL_212_1	0.001	1	CARFHYGSSWFAYW
TCL_212_1	0.001	1	CARSTYYYGSSSPLWYFDV
TCL_212_1	0.001	1	CARGGKNPNWVDYW
TCL_212_1	0.001	1	CVRENYSNYGGAMDYW
TCL_212_1	0.001	1	CARIAENYDYW
TCL_212_1	0.001	1	CARKLRAFYAMDYW
TCL_212_1	0.001	1	CARKEVGEFITTVVAPMDY
TCL_212_1	0.001	1	CARIFYYDYGFAYW
TCL_212_1	0.001	1	CTRRNDGYSKSYFDYW

TCL_212_1	0.001	1	CAREIVGLRYAMDYW
TCL_212_1	0.001	1	CSVRDLLRLRYFDVW
TCL_212_1	0.001	1	CMRYGNYYWYFDVW
TCL_212_1	0.001	1	CMRYGNYYWYFDVW
TCL_212_1	0.001	1	CARVGAYYYGSSPYYFDYW
TCL_212_1	0.001	1	CARRYYGSSYWYFDVW
TCL_212_1	0.001	1	CAMMTSSLRYFDVW
TCL_212_1	0.001	1	CVTPTVEPYYFDYW
TCL_212_1	0.001	1	CARIIISEAMDYW
TCL_212_1	0.001	1	CMRYGNYYWYFDVW
TCL_212_1	0.001	1	CTRRNDGYSKSYFDYW
TCL_212_1	0.001	1	CMRYCNYFLYFDVW
TCL_212_1	0.001	1	CMRYGNYYYWYFDVW
TCL_212_1	0.001	1	CMRYGNYYWYFDVW
TCL_212_1	0.001	1	CMRYGNYYWYFDVW
TCL_212_1	0.001	1	CMRYGNYYWYFDVW
TCL_212_1	0.001	1	CMRYGNYYWYFDVW
TCL_212_1	0.001	1	CMRYGNYYWYFDVW
TCL_212_1	0.001	1	CMRYGNYYWYFDVW
TCL_212_1	0.001	1	CMRYGNYYWYFDVW
TCL_212_1	0.001	1	CTRRNDGYSKSYFDYW
TCL_212_1	0.001	1	CTRRNDGYSKSYFDYW
TCL_212_1	0.001	1	CTRRNDGYSKSYFDYW
TCL_212_1	0.001	1	CTMGGFAYW
TCL_212_1	0.001	1	CARSGGWLLPMDYW
TCL_212_1	0.001	1	CLTLNASVRYFDVW
TCL_212_1	0.001	1	CVRDYYRYFDVW
TCL_212_1	0.001	1	CARNNGWLLNAMDYW
TCL_212_1	0.001	1	CMRYGNYYWYFDVW
TCL_212_1	0.001	1	CAKRRLYRGDYAMDYW
TCL_212_1	0.001	1	CAGNYVRFRYFDYW
TCL_212_1	0.001	1	CARPRGLRPFAYW
TCL_212_1	0.001	1	CASSWDGAPLDYW
TCL_212_1	0.001	1	CMRYGNYYWYFDVW
TCL_212_1	0.001	1	CVKCAFIGEDFAYW
TCL_212_1	0.001	1	CTRGLLRSSYYYAMDYW
TCL_212_1	0.001	1	CARFGYGSSLGVW
TCL_212_1	0.001	1	CVRRGLYDGYYSFAYW
TCL_212_1	0.001	1	CTRRNDGYSKSYFDYW
TCL_212_1	0.001	1	CARIRYYGSYYFDYW
TCL_212_1	0.001	1	CASSWDGAPLDYW
TCL_212_1	0.001	1	CARRYYGSSYWYFDVW
TCL_212_1	0.001	1	CMRYGNYYWYFDVW
TCL_212_1	0.001	1	CMRYGNYYWYFDVW
TCL_212_1	0.001	1	CMRYGNYYWYFDVW
TCL_212_1	0.001	1	CMRYGNYYWYFDVW
TCL_212_1	0.001	1	CMRHSNYWYFDVW
TCL_212_1	0.001	1	CFVGLCEDFAYW
TCL_212_1	0.001	1	CAGWPTREDFAYW
TCL_212_1	0.001	1	CAKRRLYRGDYAMGYW
TCL_212_1	0.001	1	CTRRNDGYSKSYFDYW
total	100.000	79359	

rearrangement

TCACTATCTTCAGAGACAATGACAAGAGCACCCTGTACCTGCAGATGAGCAATGTGCGATCGGAGGACACAGCCACGTATTTC
TGACTGCAGACAAATCCTCCAGCACAGCCTACATGGAGCTCCGCAGCCTGACATCTGAGGACTCTGCCGTCTATTACTGTACAA
TGACTGTAGACACATCCTCCAGCACAGCCTACATGCAGCTCAGCAGCCTGACATCTGAGGACTCTGCGGTCTATTACTGTGCAA
CTGTAGACAAGTCCTCCAGCACAGCCTACATGGAGCTCCGCAGCCTGACATCTGAGGACTCTGCAGTCTATTACTGTGCAAGA
GATTCACTATCTTCAGAGACAATGACAAGAGCACCCTGTACCTGCAGATGAGCAATGTGCGATCGGAGGACACAGCCACGTAT
GATTCACTATCTTCAGAGACAATGACAAGAGCACCCTGTACCTGCAGATGAGCAATGTGCGATCGGAGGACACAGCCACGTAT
TCTCCATTACTAGAGAAACGTCAAAGAACCAGTTCTTCCTCCAATTGAACTCTGTGACCACAGAGGACACAGCCATGTATTACT
TCACTATCTTCAGAGACAATGACAAGAGCACCCTGTACCTGCAGATGAGCAATGTGCGATCGGAGGACACAGCCACGTATTTC
TTCACTATCTTCAGAGACAATGACAAGAGCACCTGTACCTGCAGATGAGCAATGTGCGATCGGAGGACACAGCCACGTATTTC
TCTCCATTACTAGAGAAACGTCAAAGAACCAGTTCTTCCTCCAATTGAACTCTGTGACCACAGAGGACACAGCCATGTATTACT
CCACATTGACTGTAGACCAATCTTCCAGCACAGCCTACATGCAGCTCAACAGCCTGACATCTGAGGACTCTGCAGTCTATTACT
GACGGTTTGCCTTCTCTTTGGAAACCTCTGCCAGCACTGCCTATTTGCAGATCAACAACCTCAAAAATGAGGACACGGCTACAT
TGACTGTAGACACATCCTCCAGCACAGCCTACATGCAGCTCAGCAGCCTGACATCTGAGGACTCTGCGGTCTATTACTGTGCAA
CCACATTGACTGTAGACAAATCCTCCAGCACAGCCTACATGCAGCTCAGCAGCCTGACATCTGAGGACTCTGCGGTCTATTACT
AGAGCAAGGCCACACTGACTGTAGACAAATCCTCCAGCACAGCCTACATGCAGCTCAGCAGCCTGACATCTGAGGACTCTGCG
CCATCTCCAGAGACAATGCCAAGAACAACCTGTACCTGCAAATGAGCCATCTGAAGTCTGAGGACACAGCCATGTATTACTGT
TGAAAGGCAGATTCGCCATTTCAAGAGATGATTCAAAAAGCAGTGTCTACCTAGAGATGAACAGATTAAGAGAGGAAGACAC
TCACAATCTCCAAGGATACCTCCAAAAACCAGGTATTCCTCAAGATCGCCAATGTGGACACTGCAGATACTGCCACATACTACT
CTGTGAAAGGGAGGTTCACCATCTCAAGAGATGATTCCAAAAGTAGTGTCTACCTGCAAATGAACAACTTAAGGGCTGAAGA
TTCACTATCTTCAGAGACAATGACAAGAGCACCCTGTACCGCAGATGAGCAATGTGCGATCGGAGGACACAGCCACGTATTTC
TTCACTATCTCAGAGACAATGACAAGAGCACCCTGTACCTGCAGATGAGCAATGTGCGATCGGAGGACACAGCCACGTATTTC
TTCACTATCTTCAGAGACAATGACAGAGCACCCTGTACCTGCAGATGAGCAATGTGCGATCGGAGGACACAGCCACGTATTTC
CACTATCTTCAGAGACAATGACAAGAGCACCCCTGTACCTGCAGATGAGCAATGTGCGATCGGAGGACACAGCCACGTATTTC
TTCACTATCTTCAGAGACATGACAAGAGCACCCTGTACCTGCAGATGAGCAATGTGCGATCGGAGGACACAGCCACGTATTTC
TTCACTATCTTCAGAGAAATGACAAGAGCACCCTGTACCTGCAGATGAGCAATGTGCGATCGGAGGACACAGCCACGTATTTC
TTCACTATCTTCAGAGACAATGACAAGAGCACCCGTACCTGCAGATGAGCAATGTGCGATCGGAGGACACAGCCACGTATTTC
TTCACTATCTTCAGAGACAATGACAAGAGACCCTGTACCTGCAGATGAGCAATGTGCGATCGGAGGACACAGCCACGTATTTC
TTCACTATCTTCAGAGACAATGACAAGAGCACCCTGTACCTGAGATGAGCAATGTGCGATCGGAGGACACAGCCACGTATTTC
TTCACTATCTTAGAGACAATGACAAGAGCACCCTGTACCTGCAGATGAGCAATGTGCGATCGGAGGACACAGCCACGTATTTC
ATTCACTATCTTCAGACAATGACAAGAGCACCCTGTACCTGCAGATGAGCAATGTGCGATCGGAGGACACAGCCACGTATTTC
TTCACTATCTTCAGAGACAATGAAAGAGCACCCTGTACCTGCAGATGAGCAATGTGCGATCGGAGGACACAGCCACGTATTTC
CTGACTGCAGACAATCCTCCAGCACAGCCTACATGGAGCTCCGCAGCCTGACATCTGAGGACTCTGCCGTCTATTACTGTACAA
TTCACTATCTTCAGAGACAATGACAAGAGCACCCTGTACTGCAGATGAGCAATGTGCGATCGGAGGACACAGCCACGTATTTC
GCCGATTCACCATCTCCAGAGACAATGCCAAGAACACCCTGTTCCTGCAAATGACCAGTCTGAGGTCTGAGGACACGGCCATG
CTCGAGACACATCCAAGAACCAGTATTACCTGCAGTTGAATTCTGTGACTACTGAGGACACAGCCACATATTACTGTGCAAGAT
CCACACTGACTTCAGACACATCCTCCAGCACAGCCTACATGCAGCTCAGCAGCCTGACATCTGAGGACTCTGCAATCTATTTCT
CAACACTGACTGCAGACAAATCCTCCAGCACTGCCTACATGCAGCTCAGCAGCCTGACATCTGAGGACTCTGCGGTCTATTTCT
ACAATGAGAAGTTCAAGGGCAAGGCCACATTGACTGCAGACAAATCCTCCAGCACAGCCTACATGCAGCTCAACAGCCTGACA
AGGCCACACTGACGGCAGACACATCCTCCAGCACTGCCTACATGCAGCTCAGCAGCCTAACATCTGAGGACTCTGCGGTCTAT
CCACTATAACTGCAGACACATCCTCCAACACAGCCTACCTGCAGCTCAGCAGCCTGACATCTGAGGACACTGCCATCTATTACT
TCACCATCTCCAGAGATAATTCCCAAAGCATCCTCTATCTTCAAATGAATGCCCTGAGAGCTGAGGACAGTGCCACTTATTACT
CATCCAAGAACCAGTATTACCTGCAGTTGAATTCTGTGACTACTGAGGACACAGCCACATATTACTGTGCAAGATCCACCTATT
CCACATTGACTGTAGACCAATCTTCCAGCACAGCCTACATGCAGCTCAACAGCCTGACATCTGAGGACTCTGCAGTCTATTACT
TCTCCAGAGATGATTCACAAAGCATGCTCTATCTGCAAATGAACAACCTGAAAACTGAGGACACAGCCATGTATTACTGTGTG
AGAGTCGGCTCACAATCTCCAAGGATACCTCCAAAAACCAGGTATTCCTCAAGATCGCCAATGTGGACACTGCAGATACTGCC
CCACATTGACTGTAGACAAGTCCTCCAGCACAGCCTACATGGAGCTCCGCAGCCTGACATCTGAGGACTCTGCAGTCTATTACT
CATCCTCCAGCACAGCCTACATGCAGCTCAGCAGCCTGACATCTGAGGACTCTGCGGTCTATTACTGTGCAAGAAAGGAGGTG
TCACAATCTCCAAGGATACCTCCAAAAACCAGGTATTCCTCAAGATCGCCAATGTGGACACTGCAGATACTGCCACATACTACT
CTGACTGCAGACAAATCCTCAGCACAGCCTACATGGAGCTCCGCAGCCTGACATCTGAGGACTCTGCCGTCTATTACTGTACAA

CCATCTCCAGAGACAATGCCAAGAACAACCTGTACCTGCAAATGAGCCATCTGAAGTCTGAGGACACAGCCATGTATTACTGTC
CCATTTCAAGAGATGATTCAAAAAGCAGTGTCTACCTAGAGATGAACAGATTAAGAGAGGAAGACACTGCCACTTATTTTGTA
TTCACTATCTTCAGAGACAATGACAAGAGCACCCTGTACCTCAGATGAGCAATGTGCGATCGGAGGACACAGCCACGTATTTCT
TCTCTATGATCAGAGACAATGACAAGAGCACCCTGTACCTGCAGATGAGCAATGTGCGATCGGAGGACACAGCCACGTATTTC
ACAAGTCCTCCAGCACAGCCTACATGGAGCTCAACAGCCTGACATCTGAGGACTCTGCAGTCTATTACTGTGCAAGAGTGGGG
TGAAGGTAGACACATCCTCCAGCACAGCCTACATGCAGCTCAGCAGCCTGACATCTGAGGACTCTGCGGTCTATTACTGTGCAA
TGAGCATCAGCAAGGACAACTCCAAGAGCCAAATTTTCTTTAAAATGAACAGTCTGCAAGCTGATGACACTGCCATATACTACT
TTGCCTTCTCTTTGGAGACCTCTGCCAGCACTGCCTATTTGCAGATCAACAACCTCAAAAATGAGGACACGGCTACATATTTCTG
GACTGAGCATCAGCAAAGACAACTCCAAGAGTCAAGTTTTCTTAAAAATGAACAGTCTGCAAACTGATGACACAGCCAGGTAC
TTCACATCTTCAGAGACAATGACAAGAGCACCCTGTACCTGCAGATGAGCAATGTGCGATCGGAGGACACAGCCACGTATTTCT
CTGACTGCAGACAAATCCTCCAGCACAGCCTACATGAGCTCCGCAGCCTGACATCTGAGGACTCTGCCGTCTATTACTGTACAA
TCACTATCTTCAGAGACAATGACAAGAGCACCCTGTACCTGCAGATGAGCAATGTGCGATCGGAGGACACAGCCACGTATTTCT
CTATCTTCAGAGACAATGACAAGAGCACCCTGTACCTGCAGATGAGCAATGTGCGATCGGAGGACACAGCCACGTATTTCTGT
TTCACTATCTTCAGAGACAATGACAAGAGCACCCTGTCCTGCAGATGAGCAATGTGCGATCGGAGGACACAGCCACGTATTTCT
TTCACTATCTTCAGAGACAATGACAAGGCACCCTGTACCTGCAGATGAGCAATGTGCGATCGGAGGACACAGCCACGTATTTCT
ATTCACTATCTAGAGACAATGACAAGAGCACCCTGTACCTGCAGATGAGCAATGTGCGATCGGAGGACACAGCCACGTATTTCT
TAACAAACTTCAGAGACAATGACAAGAGCACCCTGTACCTGCAGATGAGCAATGTGCGATCGGAGGACACAGCCACGTATTTC
TTCACTTTCTCAGAGACAATGACAAGAGCACCCTGTACCTGCAGATGAGCAATGTGCGATCGGAGGACACAGCCACGTATTTCT
CACTATCTTCAGAGACAAATGACAAGAGCACCCTGTACCTGCAGATGAGCAATGTGCGATCGGAGGACACAGCCACGTATTTC
TTCACTATCTTCAGAGACAAGACAAGAGCACCCTGTACCTGCAGATGAGCAATGTGCGATCGGAGGACACAGCCACGTATTTCT
CTGACTGCAGACAAATCCTCCAGCACAGCTACATGGAGCTCCGCAGCCTGACATCTGAGGACTCTGCCGTCTATTACTGTACAA
CTGACTGCAGACAAATCTCCAGCACAGCCTACATGGAGCTCCGCAGCCTGACATCTGAGGACTCTGCCGTCTATTACTGTACAA
TGACTGCAGACACATCCTCCAACACAGCCTACCTGCAGCTCAGCAGCCTGACATCTGAGGACACTGCCGTCTATTACTGTACAA
AGTTCAAGGGCAAGGCCATACTGACTGCAGACAAATCCTCCAGCACAGCCTACATGGAGCTCCGCAGCCTGACATCTGAGGAC
CCACTATAACTGCAGACACATCCTCCAACACAGCCTACCTGCAGCTCAGCAGCCTGACATCTGAGGACACTGCCATCTATTACTG
CCACACTTACTGTAGACAAATCCTCCAGCACAGCCTACATGTTGCTCAGCAGCCTGACCTCTGAGGACTCTGCGGTCTATTTCTG
ACAGATTCACCATCTCCAGAGATGATTCACAAAGCATGCTCTATCTGCAAATGAACAACCTGAAAACTGAGGACACAGCCATGT
GCATCAGCAAAGACAACTCCAAGAGTCAAGTTTTCTTAAAAATGAACAGTCTGCAAACTGATGACACAGCCAGGTACTACTGTC
TACATATCTTCAGAGACAATGACAAGAGCACCCTGTACCTGCAGATGAGCAATGTGCGATCGGAGGACACAGCCACGTATTTCT
TCAGCAAAGACAACTCCAAGAGCCAAGTTTTCTTAAAAATGAACAGTCTGCAAACTGATGACACAGCCATGTACTACTGTGCCA
CCACACTGACTGCAGACAAATCCTCCAGCACAGCGTACATGGAGCTCCGCAGCCTGACATCTGAGGACTCTGCGGTCTATTTCT
AATTCATCATCTCCAGAGACAACGCCAAAAATACGCTGTACCTGCAAATGAGCAAAGTGAGATCTGAGGACACAGCCCTTTATT
AGGCCACACTGACTGTAGACAAACCCTCCAGCACAGCCTACATGCAGCTCAGCAGCCTGACATCTGAGGACTCTGCGGTCTATT
TGAATTTCTTCAGAGACAATGACAAGAGCACCCTGTACCTGCAGATGAGCAATGTGCGATCGGAGGACACAGCCACGTATTTCT
TCACCATCTCCAGAGATAATTCCCAAAACATCCTCTATCTTCAAATGAACACCCTGAGGGCTGAGGACAGTGCCACTTATTACTG
CCAGAGACAATGCCAGGAACACCCTGTACCTGCAAATGAGCAGTCTGAAGTCTGAGGACACAGCCATGTATTACTGTACAAGA
GGTTCACCATCTCCAGAGATAATTCCCAAAGCATCCTCTATCTTCAAATGAATGCCCTGAGAGCTGAGGACAGTGCCACTTATTA
TCTCCAGAGATGATTCAGAAAGCATGCTCTATCTGCAAATGAACAACTTGAAAACTGAGGACACAGCCATGTATTACTGTGTGA
GACTGCAGACAAAATCCTCCAGCACAGCCTACATGGAGCTCCGCAGCCTGACATCTGAGGACTCTGCCGTCTATTACTGTACAA
CAATCTCCAAGGATACCTCCAAAAACCAGGTATTCCTCAAGATCGCCAATGTGGACACTGCAGATACTGCCACATACTACTGTG
AGGCCACACTGACTGTAGACAAACCCTCCAGCACAGCCTACATGCAGCTCAGCAGCCTGACATCTGAGGACTCTGCGGTCTATT
CTGACTGTAGACACATCCTCAGCACAGCCTACATGCAGCTCAGCAGCCTGACATCTGAGGACTCTGCGGTCTATTACTGTGCAA
ATTCACTATCTTCAGAGACAATGAAGAGCACCCTGTACCTGCAGATGAGCAATGTGCGATCGGAGGACACAGCCACGTATTTCT
TTGACTATCTTCAGAGAAATGACAAGAGCACCCTGTACCTGCAGATGAGCAATGTGCGATCGGAGGACACAGCCACGTATTTCT
ATTCACTATCTTCAGAGACAATGACAAGAGCACCGTACCTGCAGATGAGCAATGTGCGATCGGAGGACACAGCCACGTATTTCT
ATTCACTATCTTCAGAGACAATGACAAGAGCACCCTGTACCTGCATGAGCAATGTGCGATCGGAGGACACAGCCACGTATTTCT
GTTTCACTATCTTCAGAGACAATGACAAGAGCACCCTGTACCTGCAGATGAGCAATGTGCGATCGGAGGACACAGCCACGTAT
GCAAGGCCACATTGACTGTAGACAAATCCTCTAGCACAGCCCACATGGAGCTCCGGAGCCTGACATCTGAGGACTCTGCAGTCT
GATTCATCGTCTCAAGAGATGATTCCAAAAGCAGTGCATACATGCAGATGAACAGCTTAAGAAAGGAAGACACTGCCGTTTAT
TCAGCAAAGACAACTCCAAGAGCCAAGTTTTCTTAAAAATGAACAGTCTGCAAACTGATGACACAGCCATGTACTACTGTGCCA
CTGACTCAGACAAATCCTCCAGCACAGCCTACATGGAGCTCCGCAGCCTGACATCTGAGGACTCTGCCGTCTATTACTGTACAA

TGTATGAGATATGGTAACTACTACTGGTACTTCGATGTCTGG
GACGGAATGATGGTTACTCCAAGTCCTACTTTGACTACTGG
GGAGGTACTACGGTAGTAGCTACTGGTACTTCGATGTCTGG
AGCTACTATGATTACGTATATTACTATGCTATGGACTACTGG
TTCTGTATGAGATATAGTAACTACTGGTACTTCGATGTCTGG
TTCTGTATGAGATATAGTAGCTACTGGTACTTCGATGTCTGG
GTGCAGGAGACTATGATGGTTACTGGTACTTCGATGTCTGG
TGTATGAGATACGGTAGTAGCTACTGGTACTTCGATGTCTGG
TGTATGAGATATGGTAACTACTACTGGTACTTCGATGTCTGG
GTGCAGGAGACAGTGATGGTTACTGGTACTTCGATGTCTGG
GTGCAAGAGATGATTACGACGGCCGGGGGTTTGCTTACTGG
ATTTCTGTGCAAGATTATTTACTACGGTAGTAGACTACTGG
GAAGGTATTACTACGGTAGTAGCTACTACTTTGACTACTGG
GTGCAAGATACTATAGTAACTACTGGTACTTCGATGTCTGG
GTCTATTATTGTGCAGGCCCTGGACTCCCGTACTATTACTGG
GCAAGAGATGATGATGGTTACCTCTGGTACTTCGATGTCTGG
TGCCACTTATTTTTGTAGTTACTGTGAAGACTTTGCTTACTGG
GTGCTCGAATGAGGTATGATTACGCCTGGTTTGCTTACTGG
ACTGGAATTTATTACTGCACAGGCTGGGGCTTTGACTACTGG
TGTATGAGATATGGTAACTACTACTGGTACTTCGATGTCTGG
TGTATGAGATATGGTAACTACTACTGGTACTTCGATGTCTGG
TGTATGAGATATGGTAACTACTACTGGTACTTCGATGTCTGG
TGTATGAGATATGGTAACTACTACTGGTACTTCGATGTCTGG
TGTATGAGATATGGTAACTACTACTGGTACTTCGATGTCTGG
TGTATGAGATATGGTAACTACTACTGGTACTTCGATGTCTGG
TGTATGAGATATGGTAACTACTACTGGTACTTCGATGTCTGG
TGTATGAGATATGGTAACTACTACTGGTACTTCGATGTCTGG
TGTATGAGATATGGTAACTACTACTGGTACTTCGATGTCTGG
TGTATGAGATATGGTAACTACTACTGGTACTTCGATGTCTGG
TGTATGAGATATGGTAACTACTACTGGTACTTCGATGTCTGG
TGTATGAGATATGGTAACTACTACTGGTACTTCGATGTCTGG
GACGGAATGATGGTTACTCCAAGTCCTACTTTGACTACTGG
TGTATGAGATATGGTAACTACTACTGGTACTTCGATGTCTGG
TATTACTGTGCAAGGCCGGTCGGGAATGCTATGGACTACTGG
ATGTTTACTACGGTAGTAGCTCCTATGCTATGGACTACTGG
TATGGCTAATCATGTCACCGTCTGGTACTTCGATGTCTGG
GTGCAAGATCCGGGTATATTACTAAGGGGTTTGCTTACTGG
TCTGAGGACTCTGCAGTCTTCAGTGAAGACTTTGCTTACTGG
ACTGTGCAAGAGGGATGGTTACGAGGGCTATGGACTACTGG
GTCACTTCTCACCCGGCCGTCTTAGGTACTTCGATGTCTGG
TGCAAGATTCCACTACGGTAGTTCCTGGTTTGCTTACTGG
ACTACGGTAGTAGCAGTCCCCTCTGGTACTTCGATGTCTGG
GTGCAAGAGGGGGAAAAAACCCTAACTGGGTTGACTACTGG
AGAGAAAACTATAGTAACTACGGAGGGGCTATGGACTACTGG
ACATACTACTGTGCTCGAATAGCTGAGAACTATGATTACTGG
GTGCAAGAAAACTACGGGCCTTCTATGCTATGGACTACTGG
GGGGAATTTATTACTACGGTAGTAGCCCCTATGGACTACTGG
GTGCTCGAATCTTCTACTATGATTACGGGTTTGCTTACTGG
GACGGAATGATGGTTACTCCAAGTCCTACTTTGACTACTGG

GCAAGAGAGATTGTGGGATTACGGTATGCTATGGACTACTGG
AGTGTCCGCGACCTTCTCCGTCTTAGGTACTTCGATGTCTGG
TGTATGAGATATGGTAACTACTACTGGTACTTCGATGTCTGG
TGTATGAGATATGGTAACTACTACTGGTACTTCGATGTCTGG
GCCTATTACTACGGTAGTAGCCCGTACTACTTTGACTACTGG
AGGAGGTACTACGGTAGTAGCTACTGGTACTTCGATGTCTGG
TGTGCCATGATGACTTCCAGTCTTAGGTACTTCGATGTCTGG
GTGTAACCCCTACGGTAGAACCTTACTACTTTGACTACTGG
TACTGTGCCAGGATTATTATTTCCGAGGCTATGGACTACTGG
TGTATGAGATATGGTAACTACTACTGGTACTTCGATGTCTGG
GACGGAATGATGGTTACTCCAAGTCCTACTTTGACTACTGG
TGTATGAGATATTGTAACTACTTCTTGTACTTCGATGTCTGG
ATGAGATATGGTAACTACTACTACTGGTACTTCGATGTCTGG
TGTATGAGATATGGTAACTACTACTGGTACTTCGATGTCTGG
TGTATGAGATATGGTAACTACTACTGGTACTTCGATGTCTGG
TGTATGAGATATGGTAACTACTACTGGTACTTCGATGTCTGG
CTGTATGAGATATGGTAACTACTACTGGTACTTCGATGTCTGG
TGTATGAGATATGGTAACTACTACTGGTACTTCGATGTCTGG
TGTATGAGATATGGTAACTACTACTGGTACTTCGATGTCTGG
TGTATGAGATATGGTAACTACTACTGGTACTTCGATGTCTGG
AGACGGAATGATGGTTACTCCAAGTCCTACTTTGACTACTGG
AGACGGAATGATGGTTACTCCAAGTCCTACTTTGACTACTGG
GACGGAATGATGGTTACTCCAAGTCCTACTTTGACTACTGG
CTCTGCCGTCTATTACTGTACAATGGGGGGGTTTGCTTACTGG
GTGCTAGATCGGGGGGATGGTTACTACCTATGGACTACTGG
GTCTAACCCTCAATGCCTCCGTTAGGTACTTCGATGTCTGG
TATTACTGTGTGAGAGACTACTATAGGTACTTCGATGTCTGG
GCCAGAAATAATGGATGGTTACTAAATGCTATGGACTACTGG
TGTATGAGATATGGTAACTACTACTGGTACTTCGATGTCTGG
AAACGCCGCCTCTACCGGGGGGACTATGCTATGGACTACTGG
TGTGCGGGTAACTACGTAAGGTTTAGGTACTTTGACTACTGG
TACTGTGCAAGACCGAGGGGATTACGACCGTTTGCTTACTGG
TATTGTGCATCCTCCTGGGACGGGGCCCCCCTTGACTACTGG
TGTATGAGATATGGTAACTACTACTGGTACTTCGATGTCTGG
GTGTAAAATGTGCATTTATAGGTGAAGACTTTGCTTACTGG
AGGATTACTACGGTCCTCCTATTACTATGCTATGGACTACTGG
ACTGTGCAAGATTCGGCTACGGTAGTTCCCTGGGTGTCTGG
AGACGAGGGCTCTATGATGGTTACTACTCGTTTGCTTACTGG
AGACGGAATGATGGTTACTCCAAGTCCTACTTTGACTACTGG
CTCGAATCAGATACTACGGTTCGTACTACTTTGACTACTGG
TATTGTGCATCCTCCTGGGACGGGGCCCCCCTTGACTATTGG
AGGAGGTACTACGGTAGTAGCTACTGGTACTTCGATGTCTGG
TGTATGAGATATGGTAACTACTACTGGTACTTCGATGTCTGG
TGTATGAGATATGGTAACTACTACTGGTACTTCGATGTCTGG
TGTATGAGATATGGTAACTACTACTGGTACTTCGATGTCTGG
TGTATGAGATATGGTAACTACTACTGGTACTTCGATGTCTGG
TTCTGTATGAGACATAGTAACTACTGGTACTTCGATGTCTGG
TATTATTGTTTTGTGGGGCTGTGTGAAGACTTTGCTTACTGG
TACTGTGCAGGCTGGCCAACACGTGAAGACTTTGCTTACTGG
AAACGCCGCCTCTACCGGGGGGACTATGCTATGGGCTACTGG
GACGGAATGATGGTTACTCCAAGTCCTACTTTGACTACTGG

sample_name	productive_frequency	templates	amino_acid
TCL_221_2	99.307	109362	CMRYSNYWYFDVW
TCL_221_2	0.368	405	CARPGYYGSSYTFDYW
TCL_221_2	0.099	109	CAREGGYSYWYFDVW
TCL_221_2	0.030	33	CMRYGSSYWYFDVW
TCL_221_2	0.008	9	CAGDRSGYTGFAYW
TCL_221_2	0.007	8	CMRYSNYWYFDVW
TCL_221_2	0.007	8	CARFYYYGSSYAMDYW
TCL_221_2	0.004	4	CMRYSNYWYFDVW
TCL_221_2	0.003	3	CARRYYGSSYWYFDVW
TCL_221_2	0.002	2	CARVPYYYGSRTWYFDVW
TCL_221_2	0.002	2	CAGDRYGYWYFDVW
TCL_221_2	0.002	2	CMRYSNYWYFDVW
TCL_221_2	0.002	2	CMRYSNYWYFDVW
TCL_221_2	0.002	2	CMRYSNYWYFDVW
TCL_221_2	0.002	2	CMRYSNYWYFDVW
TCL_221_2	0.002	2	CMRYSNYWYFDVW
TCL_221_2	0.002	2	CMRYSNYWYFDVW
TCL_221_2	0.002	2	CMRYSNYWYFDVW
TCL_221_2	0.002	2	CMRYSNYWYFDVW
TCL_221_2	0.002	2	CMRYSNYWYFDVW
TCL_221_2	0.002	2	CMRYSNYWYFDVW
TCL_221_2	0.002	2	CMRYSNYWYFDVW
TCL_221_2	0.002	2	CMRYSNYWYFDVW
TCL_221_2	0.001	1	CASRWLLRGGAMDYW
TCL_221_2	0.001	1	SVKARLRYFDVW
TCL_221_2	0.001	1	CTLLYYW
TCL_221_2	0.001	1	CMRYSNYWYFDVW
TCL_221_2	0.001	1	CAGHIRSEDFAYW
TCL_221_2	0.001	1	CARTDYYGNYDAMDYW
TCL_221_2	0.001	1	CMRYSNYWYFDVW
TCL_221_2	0.001	1	CMRYSNYWYFDVW
TCL_221_2	0.001	1	CMRYSNYWYFDVW
TCL_221_2	0.001	1	CMRYSNYWYFDVW
TCL_221_2	0.001	1	CMRYSNYWYFDVW
TCL_221_2	0.001	1	CMRYSNYWYFDVW
TCL_221_2	0.001	1	CMRYSNYWYFDVW
TCL_221_2	0.001	1	CARPGYYGSSYTFDYW
TCL_221_2	0.001	1	CMRYSNYWYFDVW
TCL_221_2	0.001	1	CMRYSNYWYFDVW
TCL_221_2	0.001	1	CMRYSNYWYFDVW
TCL_221_2	0.001	1	CMRYSNYWYFDVW
TCL_221_2	0.001	1	CMRYSNYWYFDVW
TCL_221_2	0.001	1	CMRYSNYWYFDVW
TCL_221_2	0.001	1	CMRYSNYWYFDVW
TCL_221_2	0.001	1	CMRYSNYWYFDVW
TCL_221_2	0.001	1	CMRYSNYWYFDVW
TCL_221_2	0.001	1	CMRYSNYWYFDVW
TCL_221_2	0.001	1	CMRYSNYWYFDVW
TCL_221_2	0.001	1	CMRYSNYWYFDVW

TCL_221_2	0.001	1	CMRYSNYWYFDVW
TCL_221_2	0.001	1	CMRYSNYWYFDVW
TCL_221_2	0.001	1	CMRYSNYWYFDVW
TCL_221_2	0.001	1	CMRYSNYWYFDVW
TCL_221_2	0.001	1	CMRYSNYWYFDVW
TCL_221_2	0.001	1	CMRYSNYWYFDVW
TCL_221_2	0.001	1	CMRYSNYWYFDVW
TCL_221_2	0.001	1	CMRYSNYWYFDVW
TCL_221_2	0.001	1	CMRYSNYWYFDVW
TCL_221_2	0.001	1	CTLLYYW
TCL_221_2	0.001	1	CMRYSIYWYFVVW
TCL_221_2	0.001	1	CMRYGSSYWYFDVW
TCL_221_2	0.001	1	CMRYSNYWYFDVW
TCL_221_2	0.001	1	CMRYSNYWYFDVW
TCL_221_2	0.001	1	CMRYSNYWYFDVW
TCL_221_2	0.001	1	CMRYSNYWYFDVW
TCL_221_2	0.001	1	CMRYSNYWYFDVW
TCL_221_2	0.001	1	CMRYSNYWYFDVW
TCL_221_2	0.001	1	CMRYSNYWYFDVW
TCL_221_2	0.001	1	CMRYSNYWYFDVW
TCL_221_2	0.001	1	CMRYSNYWYFDVW
TCL_221_2	0.001	1	CMRYSNYWYFDVW
TCL_221_2	0.001	1	CMRYSNYWYFDVW
TCL_221_2	0.001	1	CMRYSNYWYFDVW
TCL_221_2	0.001	1	CMRYSNYWYFDVW
TCL_221_2	0.001	1	CMRYSNYWYFDVW
TCL_221_2	0.001	1	CMRYSNYWYFDVW
TCL_221_2	0.001	1	CMRYSNYWYFDVW
TCL_221_2	0.001	1	CMRYSNYWYFDVW
TCL_221_2	0.001	1	CMRYSNYWYFDVW
TCL_221_2	0.001	1	CMRYSNYWYFDVW
TCL_221_2	0.001	1	CMRYSNYWYFDVW
TCL_221_2	0.001	1	CMRYSNYWYFDVW
TCL_221_2	0.001	1	CMRYSNYWYFDVW
TCL_221_2	0.001	1	CMRYSNYWYFDVW
TCL_221_2	0.001	1	CMRYSNYWYFDVW
TCL_221_2	0.001	1	CMRYSNYWYFDVW
TCL_221_2	0.001	1	CMRYSNYWYFDVW
TCL_221_2	0.001	1	CMRYSNYWYFDVW
TCL_221_2	0.001	1	CMRYSNYWYFDVW
TCL_221_2	0.001	1	CMRYSNYWYFDVW
TCL_221_2	0.001	1	CMRYSNYWYFDVW
TCL_221_2	0.001	1	CMRYSNYWYFDVW
TCL_221_2	0.001	1	CMRYSNYWYFDVW
TCL_221_2	0.001	1	CMRYSNYWYFDVW
TCL_221_2	0.001	1	CMRYSNYWYFDVW
TCL_221_2	0.001	1	CMRYSNYWYFDVW
TCL_221_2	0.001	1	CMRYSNYWYFDVW
TCL_221_2	0.001	1	CMRYSNYWYFDVW
TCL_221_2	0.001	1	CMRYSNYWYFDVW

TCL_221_2	0.001	1	CMRYSNYWYFDVW
TCL_221_2	0.001	1	CMRYSNYWYFDVW
TCL_221_2	0.001	1	CMRYSNYWYFDVW
TCL_221_2	0.001	1	CMRYSNYWYFDVW
TCL_221_2	0.001	1	CMRYSNYWYFDVW
TCL_221_2	0.001	1	CMRYSNYWYFDVW
TCL_221_2	0.001	1	CMRYSNYWYFDVW
TCL_221_2	0.001	1	CMRYSNYWYFDVW
TCL_221_2	0.001	1	CMRYSNYWYFDVW
TCL_221_2	0.001	1	CMRYSNYWYFDVW
TCL_221_2	0.001	1	CMRYSNYWYFDVW
TCL_221_2	0.001	1	CMRYSNYWYFDVW
TCL_221_2	0.001	1	CMRYSNYWYFDVW
TCL_221_2	0.001	1	CMRYSNYWYFDVW
TCL_221_2	0.001	1	CMRYSNYWYFDVW
TCL_221_2	0.001	1	CMRYSNYWYFDVW
TCL_221_2	0.001	1	CTRDLDGYYWYYYAMDYW
TCL_221_2	0.001	1	CARRGPYYANYFDYW
TCL_221_2	0.001	1	CASGNYAMDYW
TCL_221_2	0.001	1	CTVKGALYYFDYW
TCL_221_2	0.001	1	CMRYSNYWYFDVW
TCL_221_2	0.001	1	CMRYSNYWYFDVW
TCL_221_2	0.001	1	CMRYSNYWYFDVW
TCL_221_2	0.001	1	CAREGGYSYWYFDVW
TCL_221_2	0.001	1	CMRYSNYWYFDVW
TCL_221_2	0.001	1	CMRYSNYWYFDVW
TCL_221_2	0.001	1	CMRYSNYWYFDVW
TCL_221_2	0.001	1	CMRYSNYWYFDVW
TCL_221_2	0.001	1	CMRYSNYWYFDVW
TCL_221_2	0.001	1	CMRYSNYWYFDVW
TCL_221_2	0.001	1	CMRYSNYWYFDVW
TCL_221_2	0.001	1	CMRYSNYWYFDVW
TCL_221_2	0.001	1	CMRYSYFWYFDVW
TCL_221_2	0.001	1	CMRYSNYWYFDVW
TCL_221_2	0.001	1	CMRYSNYWYFDVW
TCL_221_2	0.001	1	CMRYSNYWYFDVW
TCL_221_2	0.001	1	CMRYSNYWYFDVW
TCL_221_2	0.001	1	CMRYSNYWYFDVW
TCL_221_2	0.001	1	CMRYSNYWYFDVW
TCL_221_2	0.001	1	CMRYSNYWYFDVW
TCL_221_2	0.001	1	CMRYSNYWYFDVW
TCL_221_2	0.001	1	CMRYSNYWYFDVW
TCL_221_2	0.001	1	CMRYSNYWYFDVW
TCL_221_2	0.001	1	CMRYSNYWYFDVW
TCL_221_2	0.001	1	CMRYSNYWYFDVW
TCL_221_2	0.001	1	CMRYSNYWYFDVW
TCL_221_2	0.001	1	CMRYSNYWYFDVW
TCL_221_2	0.001	1	CMRYSNYWYFDVW
TCL_221_2	0.001	1	CMRYSNYWYFDVW
TCL_221_2	0.001	1	CMRYSNYWYFDVW

TCL_221_2	0.001	1	CMRYSNYWYFDVW
TCL_221_2	0.001	1	CMRYSNYWYFDVW
TCL_221_2	0.001	1	CMRYSNYWYFDVW
TCL_221_2	0.001	1	CMRYSNYWYFDVW
TCL_221_2	0.001	1	CMRYSNYWYFDVW
TCL_221_2	0.001	1	CMRYSNYWYFDVW
TCL_221_2	0.001	1	CMRYSNYWYFDVW
TCL_221_2	0.001	1	CMRYSNYWYFDVW
TCL_221_2	0.001	1	CMRYSNYWYFDVW
TCL_221_2	0.001	1	CMRYSNYWYFDVW
TCL_221_2	0.001	1	CMRYSNYWYFDVW
TCL_221_2	0.001	1	CMRYSNYWYFDVW
TCL_221_2	0.001	1	CMRYSNYWYFDVW
TCL_221_2	0.001	1	CMRYSNYWYFDVW
TCL_221_2	0.001	1	CMRYSNYWYFDVW
TCL_221_2	0.001	1	CMRYSNYWYFDVW
TCL_221_2	0.001	1	CMRYSNYWYFDVW
TCL_221_2	0.001	1	CMRYSNYWYFDVW
TCL_221_2	0.001	1	CMRYSNYWYFDVW
TCL_221_2	0.001	1	CMRYSNYWYFDVW
TCL_221_2	0.001	1	CMRYSNYWYFDVW
TCL_221_2	0.001	1	CMRYSNYWYFDVW
TCL_221_2	0.001	1	CMRYSNYWYFDVW
TCL_221_2	0.001	1	CMRYSNYWYFDVW
TCL_221_2	0.001	1	CMRYSNYWYFDVW
TCL_221_2	0.001	1	CMRYSNYWYFDVW
TCL_221_2	0.001	1	MRYSNYWYFDVW
TCL_221_2	0.001	1	CSRDDPVREGFAYW
TCL_221_2	0.001	1	CARPGYYGSSYTFDYW
TCL_221_2	0.001	1	CARRGPYYANYFDYW
total	100	110125	

rearrangement

GATTCACTATCTTCAGAGACAATGACAAGAGCACCCTGTACCTGCAGATGAGCAATGTGCGATCGGAGGACACAGCCAC
TGACTGTAGACAAATCCTCCAGCACAGCCTACATGCAGCTCAGCAGCCTGACATCTGAGGACTCTGCGGTCTATTACTGTG
CTATAACTGCAGACACATCCTCCAACACAGCCTACCTGCAGCTCAGCAGCCTGACATCTGAGGACACTGCCATCTATTACT
TCACTATCTTCAGAGACAATGACAAGAGCACCCTGTACCTGCAGATGAGCAATGTGCGATCGGAGGACACAGCCACGTAT
TCTCCATTACTAGAGAAACGTCAAAGAACCAGTTCTTCCTCCAATTGAACTCTGTGACCACAGAGGACACAGCCATGTATT
GATTACATATCTTCAGAGACAATGACAAGAGCACCCTGTACCTGCAGATGAGCAATGTGCGATCGGAGGACACAGCCAC
TGACTGTAGACACATCCTCCAGCACAGCCTACATGCAGCTCAGCAGCCTGACATCTGAGGACTCTGCGGTCTATTACTGTG
CGATTCACTATCTTCAGAGACAATGACAAGAGCACCTGTACCTGCAGATGAGCAATGTGCGATCGGAGGACACAGCCAC
TGACTGCAGACAAATCCTCCAGCACAGCCTACATGCAGTTCAGCAGCCTGACATCTGAGGACTCTGCCATCTATTACTGTG
CAGACAAATCCTCCAGCACAGCGTACATGGAGCTCCGCAGCCTGACATCTGAGGACTCTGCGGTCTATTTCTGTGCAAGA
TCTCCATTACTAGAGAAACGTCAAAGAACCAGTTCTTCCTCCAATTGAACTCTGTGACCACAGAGGACACAGCCATGTATT
CGATTCACTATCTCAGAGACAATGACAAGAGCACCCTGTACCTGCAGATGAGCAATGTGCGATCGGAGGACACAGCCAC
CGATTCACTATCTTCAGAGACAATGACAGAGCACCCTGTACCTGCAGATGAGCAATGTGCGATCGGAGGACACAGCCAC
CGATTCACTATCTTCAGAGACATGACAAGAGCACCCTGTACCTGCAGATGAGCAATGTGCGATCGGAGGACACAGCCAC
CGATTCACTATCTTCAGAGAAATGACAAGAGCACCCTGTACCTGCAGATGAGCAATGTGCGATCGGAGGACACAGCCAC
CGATTCACTATCTTCAGAGACAATGAAAGAGCACCCTGTACCTGCAGATGAGCAATGTGCGATCGGAGGACACAGCCAC
CGATTCACTATCTTCAGAGACAATGACAAGAGCACCCTGTACCTCAGATGAGCAATGTGCGATCGGAGGACACAGCCACG
GATGACATATCTTCAGAGACAATGACAAGAGCACCCTGTACCTGCAGATGAGCAATGTGCGATCGGAGGACACAGCCAC
CGATTCACTATCTTCAGAGACAATGACAAGAGACCCTGTACCTGCAGATGAGCAATGTGCGATCGGAGGACACAGCCAC
CGATTCACTATCTTCAGAGACAATGACAAGAGCACCCGTACCTGCAGATGAGCAATGTGCGATCGGAGGACACAGCCAC
CGATTCACTATCTTCAGAGACAATGACAAGAGCCCCTGTACCTGCAGATGAGCAATGTGCGATCGGAGGACACAGCCAC
GTATACCTATCTTCAGAGACAATGACAAGAGCACCCTGTACCTGCAGATGAGCAATGTGCGATCGGAGGACACAGCCAC
GTATCACATTCTTCAGAGACAATGACAAGAGCACCCTGTACCTGCAGATGAGCAATGTGCGATCGGAGGACACAGCCAC
CACTGACTGTAGACAAATCCTCCAGCACAGCCTACATGCAACTCAGCAGCCTGACATCTGAGGACTCTGCGGTCTATTACT
GCAAGGCCACATTGACTGTCGACAATACCTCCAGCACAGCCTACATGGAGCTCGGCAGCCTGACTTCTGAGGACTCTGCG
CTGAGTCTGTGAAAGGGAGGTTCACCATCTCAAGAGATGATTCCAAAAGTAGTGTCTACCTGCAAATGAACAGCTTAAGA
CGATCACTATCTTCAGAGACAATGACAAGAGCACCCTGTACCTGCAGATGAGCAATGTGCGATCGGAGGACACAGCCAC
GACTGAGCATCAGCAAGGACAACTCCAAGAGCCAAGTTTTCTTAAAAATGAACAGTCTGCAAACTGATGACACAGCCATG
TGACTGTAGACACATCCTCCAGCACAGCCTACATGCAGCTCAGCAGCCTGACATCTGAGGACTCTGCGGTCTATTACTGTG
ATTCACTATCTTCAGAGACAATGACAAGAGCACCCCTGTACCTGCAGATGAGCAATGTGCGATCGGAGGACACAGCCACG
GAATACATATCTTCAGAGACAATGACAAGAGCACCCTGTACCTGCAGATGAGCAATGTGCGATCGGAGGACACAGCCAC
CGATTCACTATCTTCAGAGACAATGACAAGAGCACCCTGTACCGCAGATGAGCAATGTGCGATCGGAGGACACAGCCAC
CGATTCACTATCTTAGAGACAATGACAAGAGCACCCTGTACCTGCAGATGAGCAATGTGCGATCGGAGGACACAGCCAC
TCGATTCACTATCTTCAGACAATGACAAGAGCACCCTGTACCTGCAGATGAGCAATGTGCGATCGGAGGACACAGCCACG
CGATTCACTATCTTCAGAGACAAGACAAGAGCACCCTGTACCTGCAGATGAGCAATGTGCGATCGGAGGACACAGCCAC
TCTTCCATATCTTCAGAGACAATGACAAGAGCACCCTGTACCTGCAGATGAGCAATGTGCGATCGGAGGACACAGCCACG
TTAATGTAGACAAATCCTCCAGCACAGCCTACATGCAGCTCAGCAGCCTGACATCTGAGGACTCTGCGGTCTATTACTGTG
TAATAAATATCTTCAGAGACAATGACAAGAGCACCCTGTACCTGCAGATGAGCAATGTGCGATCGGAGGACACAGCCAC
GAATCACTAAGATCAGAGACAATGACAAGAGCACCCTGTACCTGCAGATGAGCAATGTGCGATCGGAGGACACAGCCAC
CGATTCACTATCTTCAGAGACAATGACAAGAGCACCCTGTACCTGAGATGAGCAATGTGCGATCGGAGGACACAGCCAC
GTTTAAATTTCTTCAGAGACAATGACAAGAGCACCCTGTACCTGCAGATGAGCAATGTGCGATCGGAGGACACAGCCACG
GATTGAGATTCTTCAGAGACAATGACAAGAGCACCCTGTACCTGCAGATGAGCAATGTGCGATCGGAGGACACAGCCAC
GATGAACATTCTTCAGAGACAATGACAAGAGCACCCTGTACCTGCAGATGAGCAATGTGCGATCGGAGGACACAGCCAC
CGATTACTATCTTCAGAGACAATGACAAGAGCACCCTGTACCTGCAGATGAGCAATGTGCGATCGGAGGACACAGCCAC
GATGATATATCTTCAGAGACAATGACAAGAGCACCCTGTACCTGCAGATGAGCAATGTGCGATCGGAGGACACAGCCAC
CGATTCACATCTTCAGAGACAATGACAAGAGCACCCTGTACCTGCAGATGAGCAATGTGCGATCGGAGGACACAGCCAC
GATTCAATAAGATCAGAGACAATGACAAGAGCACCCTGTACCTGCAGATGAGCAATGTGCGATCGGAGGACACAGCCAC
CGATTCACTATCTTCAGAGACAATGACAAGAGCACCCTGTCCTGCAGATGAGCAATGTGCGATCGGAGGACACAGCCAC
TATTCTCATTCTTCAGAGACAATGACAAGAGCACCCTGTACCTGCAGATGAGCAATGTGCGATCGGAGGACACAGCCACG

TATGCCCTCTCTTCAGAGACAATGACAAGAGCACCCTGTACCTGCAGATGAGCAATGTGCGATCGGAGGACACAGCCACG
GCATCCATATCTTCAGAGACAATGACAAGAGCACCCTGTACCTGCAGATGAGCAATGTGCGATCGGAGGACACAGCCAC
GAAGCCATATCTTCAGAGACAATGACAAGAGCACCCTGTACCTGCAGATGAGCAATGTGCGATCGGAGGACACAGCCAC
TCTTCTCAATCTTCAGAGACAATGACAAGAGCACCCTGTACCTGCAGATGAGCAATGTGCGATCGGAGGACACAGCCACG
GAATCCATAGCTTCAGAGACAATGACAAGAGCACCCTGTACCTGCAGATGAGCAATGTGCGATCGGAGGACACAGCCAC
GATGCACTTGATTCAGAGACAATGACAAGAGCACCCTGTACCTGCAGATGAGCAATGTGCGATCGGAGGACACAGCCAC
GATTACCTAGATTCAGAGACAATGACAAGAGCACCCTGTACCTGCAGATGAGCAATGTGCGATCGGAGGACACAGCCAC
GATTCTATATGATCAGAGACAATGACAAGAGCACCCTGTACCTGCAGATGAGCAATGTGCGATCGGAGGACACAGCCAC
TCGATTCACTATCTTCAGAGACAATGACAAGAGCACCCTACCTGCAGATGAGCAATGTGCGATCGGAGGACACAGCCACG
CGGAGTCTGTGAAAGGGAGGTTCACCATCTCAAGAGATGATTCCAAAAGTAGTGTCTACCTGCAAATGAACAGCTTAAG
GAATCACTATCTTCAGAGACAATGACAAGAGCACCCTGTACCTGCAGATGAGCAATGTGCGATCGGAGGACACAGCCAC
TCACATTCTTCAGAGACAATGACAAGAGCACCCTGTACCTGCAGATGAGCAATGTGCGATCGGAGGACACAGCCACGTA
ACGAGATACACTCTTTCCCTACACGACGCTCTTCCGATCTATATCAGATGAGCAATGTGCGATCGGAGGACACAGCCACG
ATCGATTCACTATCTTCAGAGACAATGAGAGCACCCTGTACCTGCAGATGAGCAATGTGCGATCGGAGGACACAGCCACG
TCGATTCACTATCTTCAGAGACAATGAAGAGCACCCTGTACCTGCAGATGAGCAATGTGCGATCGGAGGACACAGCCACG
GATTCACATAGTTCAGAGACAATGACAAGAGCACCCTGTACCTGCAGATGAGCAATGTGCGATCGGAGGACACAGCCAC
GATTAAATAAGTTCAGAGACAATGACAAGAGCACCCTGTACCTGCAGATGAGCAATGTGCGATCGGAGGACACAGCCAC
GAAGCTCTTTCTTCAGAGACAATGACAAGAGCACCCTGTACCTGCAGATGAGCAATGTGCGATCGGAGGACACAGCCAC
TATTACCTTTCTTCAGAGACAATGACAAGAGCACCCTGTACCTGCAGATGAGCAATGTGCGATCGGAGGACACAGCCACG
GAGTCCCATTCTTCAGAGACAATGACAAGAGCACCCTGTACCTGCAGATGAGCAATGTGCGATCGGAGGACACAGCCAC
GTTTGACATTCTTCAGAGACAATGACAAGAGCACCCTGTACCTGCAGATGAGCAATGTGCGATCGGAGGACACAGCCAC
CATGCACATTCTTCAGAGACAATGACAAGAGCACCCTGTACCTGCAGATGAGCAATGTGCGATCGGAGGACACAGCCAC
GAGTAACATTCTTCAGAGACAATGACAAGAGCACCCTGTACCTGCAGATGAGCAATGTGCGATCGGAGGACACAGCCAC
GTGTCTCTCTCTTCAGAGACAATGACAAGAGCACCCTGTACCTGCAGATGAGCAATGTGCGATCGGAGGACACAGCCACG
GTTTACCTCTCTTCAGAGACAATGACAAGAGCACCCTGTACCTGCAGATGAGCAATGTGCGATCGGAGGACACAGCCACG
GCTTACCTCTCTTCAGAGACAATGACAAGAGCACCCTGTACCTGCAGATGAGCAATGTGCGATCGGAGGACACAGCCACG
GATTTCATCTCTTCAGAGACAATGACAAGAGCACCCTGTACCTGCAGATGAGCAATGTGCGATCGGAGGACACAGCCACG
GAATCTCACTCTTCAGAGACAATGACAAGAGCACCCTGTACCTGCAGATGAGCAATGTGCGATCGGAGGACACAGCCAC
GATTACCACTCTTCAGAGACAATGACAAGAGCACCCTGTACCTGCAGATGAGCAATGTGCGATCGGAGGACACAGCCAC
GCTGCACACTCTTCAGAGACAATGACAAGAGCACCCTGTACCTGCAGATGAGCAATGTGCGATCGGAGGACACAGCCAC
CTTTAAGTATCTTCAGAGACAATGACAAGAGCACCCTGTACCTGCAGATGAGCAATGTGCGATCGGAGGACACAGCCAC
TCGATTCTATCTTCAGAGACAATGACAAGAGCACCCTGTACCTGCAGATGAGCAATGTGCGATCGGAGGACACAGCCACG
GTAGATCTATCTTCAGAGACAATGACAAGAGCACCCTGTACCTGCAGATGAGCAATGTGCGATCGGAGGACACAGCCAC
TCTGACCTATCTTCAGAGACAATGACAAGAGCACCCTGTACCTGCAGATGAGCAATGTGCGATCGGAGGACACAGCCAC
GAAGCTATATCTTCAGAGACAATGACAAGAGCACCCTGTACCTGCAGATGAGCAATGTGCGATCGGAGGACACAGCCAC
GAAGCAAGATCTTCAGAGACAATGACAAGAGCACCCTGTACCTGCAGATGAGCAATGTGCGATCGGAGGACACAGCCAC
TATTAATAATCTTCAGAGACAATGACAAGAGCACCCTGTACCTGCAGATGAGCAATGTGCGATCGGAGGACACAGCCAC
GTTTAAGAATCTTCAGAGACAATGACAAGAGCACCCTGTACCTGCAGATGAGCAATGTGCGATCGGAGGACACAGCCAC
TATTACCAATCTTCAGAGACAATGACAAGAGCACCCTGTACCTGCAGATGAGCAATGTGCGATCGGAGGACACAGCCAC
GTTGACCAATCTTCAGAGACAATGACAAGAGCACCCTGTACCTGCAGATGAGCAATGTGCGATCGGAGGACACAGCCAC
GAGAGACAATCTTCAGAGACAATGACAAGAGCACCCTGTACCTGCAGATGAGCAATGTGCGATCGGAGGACACAGCCAC
GAATCTAAATCTTCAGAGACAATGACAAGAGCACCCTGTACCTGCAGATGAGCAATGTGCGATCGGAGGACACAGCCAC
TATTAATTAGCTTCAGAGACAATGACAAGAGCACCCTGTACCTGCAGATGAGCAATGTGCGATCGGAGGACACAGCCAC
GATGCCATAGCTTCAGAGACAATGACAAGAGCACCCTGTACCTGCAGATGAGCAATGTGCGATCGGAGGACACAGCCAC
GTTGCAATAGCTTCAGAGACAATGACAAGAGCACCCTGTACCTGCAGATGAGCAATGTGCGATCGGAGGACACAGCCAC
GAAACACAAGCTTCAGAGACAATGACAAGAGCACCCTGTACCTGCAGATGAGCAATGTGCGATCGGAGGACACAGCCAC
GATTAAAAAGCTTCAGAGACAATGACAAGAGCACCCTGTACCTGCAGATGAGCAATGTGCGATCGGAGGACACAGCCAC
GAGTCACATACTTCAGAGACAATGACAAGAGCACCCTGTACCTGCAGATGAGCAATGTGCGATCGGAGGACACAGCCAC
GATTAAGTTTATTCAGAGACAATGACAAGAGCACCCTGTACCTGCAGATGAGCAATGTGCGATCGGAGGACACAGCCAC
GTTTAACTTTATTCAGAGACAATGACAAGAGCACCCTGTACCTGCAGATGAGCAATGTGCGATCGGAGGACACAGCCACG

GAGTAACTTTATTCAGAGACAATGACAAGAGCACCCTGTACCTGCAGATGAGCAATGTGCGATCGGAGGACACAGCCAC
GATTCTATTTATTCAGAGACAATGACAAGAGCACCCTGTACCTGCAGATGAGCAATGTGCGATCGGAGGACACAGCCACG
GATTCCATCTATTCAGAGACAATGACAAGAGCACCCTGTACCTGCAGATGAGCAATGTGCGATCGGAGGACACAGCCAC
GAATCAGAATATTCAGAGACAATGACAAGAGCACCCTGTACCTGCAGATGAGCAATGTGCGATCGGAGGACACAGCCAC
GTTTCACTTGATTCAGAGACAATGACAAGAGCACCCTGTACCTGCAGATGAGCAATGTGCGATCGGAGGACACAGCCAC
GAAGAACTAGATTCAGAGACAATGACAAGAGCACCCTGTACCTGCAGATGAGCAATGTGCGATCGGAGGACACAGCCAC
GATACTCTATAGTCAGAGACAATGACAAGAGCACCCTGTACCTGCAGATGAGCAATGTGCGATCGGAGGACACAGCCAC
GATGCTCTTTCATCAGAGACAATGACAAGAGCACCCTGTACCTGCAGATGAGCAATGTGCGATCGGAGGACACAGCCAC
GATGCACATTCATCAGAGACAATGACAAGAGCACCCTGTACCTGCAGATGAGCAATGTGCGATCGGAGGACACAGCCAC
GAAGCACATTCATCAGAGACAATGACAAGAGCACCCTGTACCTGCAGATGAGCAATGTGCGATCGGAGGACACAGCCAC
GATTAAATAGCATCAGAGACAATGACAAGAGCACCCTGTACCTGCAGATGAGCAATGTGCGATCGGAGGACACAGCCAC
GAATCACTTACATCAGAGACAATGACAAGAGCACCCTGTACCTGCAGATGAGCAATGTGCGATCGGAGGACACAGCCAC
GATTAACTTGCTGAAGAGACAATGACAAGAGCACCCTGTACCTGCAGATGAGCAATGTGCGATCGGAGGACACAGCCAC
GTTTCAATAGCTGAAGAGACAATGACAAGAGCACCCTGTACCTGCAGATGAGCAATGTGCGATCGGAGGACACAGCCAC
ATTCACTATCTTCAGAGACAAATGACAAGAGCACCCTGTACCTGCAGATGAGCAATGTGCGATCGGAGGACACAGCCAC
CGATTCACTATCTTCAGAGACAATGACAAGAGCACCCTGTACTGCAGATGAGCAATGTGCGATCGGAGGACACAGCCAC
GAGACAATGCCAGGAACACCCTGTACCTGCAAATGAGCAGTCTGAAGTCTGAGGACACAGCCATGTATTACTGTACAAG
CATTGACTGTAGACCAATCTTCCAGCACAGCCTACATGCAGCTCAACAGCCTGACATCTGAGGACTCTGCAGTCTATTACT
AATCCAGACTGAGCATCAGCAAGGACAACTCCAAGAGCCAAGTTTTCTTAAAAATGAACAGTCTGCAAACTGATGACACA
GGTTCACCATCTCAAGAGATGATTCCAAAAGTAGTGTCTACCTGCAAATGAACAACTTAAGGGCTGAAGACACTGGAATT
TAATCTATATCTTCAGAGACAATGACAAGAGCACCCTGTACCTGCAGATGAGCAATGTGCGATCGGAGGACACAGCCACG
CGAATCACTATCTTCAGAGACAATGACAAGAGCACCTGTACCTGCAGATGAGCAATGTGCGATCGGAGGACACAGCCAC
CGATTCACTATCTTCAGAGACAATGACAAGAGCACCCTGACCTGCAGATGAGCAATGTGCGATCGGAGGACACAGCCAC
CAATCACTGCAGACACATCCTCCAACACAGCCTACCTGCAGCTCAGCAGCCTGACATCTGAGGACACTGCCATCTATTACT
GCATCACACTCTTCAGAGACAATGACAAGAGCACCCTGTACCTGCAGATGAGCAATGTGCGATCGGAGGACACAGCCAC
TCTACAATATCTTCAGAGACAATGACAAGAGCACCCTGTACCTGCAGATGAGCAATGTGCGATCGGAGGACACAGCCAC
GTATCTCAATCTTCAGAGACAATGACAAGAGCACCCTGTACCTGCAGATGAGCAATGTGCGATCGGAGGACACAGCCAC
TTTTCTGTATCTTCAGAGACAATGACAAGAGCACCCTGTACCTGCAGATGAGCAATGTGCGATCGGAGGACACAGCCACG
TCTTAAGTATCTTCAGAGACAATGACAAGAGCACCCTGTACCTGCAGATGAGCAATGTGCGATCGGAGGACACAGCCAC
TTTTCCATATCTTCAGAGACAATGACAAGAGCACCCTGTACCTGCAGATGAGCAATGTGCGATCGGAGGACACAGCCACG
GATTCTATAGATTCAGAGACAATGACAAGAGCACCCTGTACCTGCAGATGAGCAATGTGCGATCGGAGGACACAGCCAC
GATTCAATAGAGTCAGAGACAATGACAAGAGCACCCTGTACCTGCAGATGAGCAATGTGCGATCGGAGGACACAGCCAC
GACTCACTATCTTCAGAGACAATGACAAGAGCACCCTGTACCTGCAGATGAGCAATGTGCGATCGGAGGACACAGCCAC
CGATTCACTATCTTCAGAGACACTGACAGAGCACCCTGTACCTGCAGATGAGCAATGTGCGATCGGAGGACACAGCCACG
TCGATTCACAATCTTCAGAGACAATGAAGAGCACCCTGTACCTGCAGATGAGCAATGTGCGATCGGAGGACACAGCCAC
CGATTCACTATCTTCAGAGACAATACAAGAGCACCCTGTACCTGCAGATGAGCAATGTGCGATCGGAGGACACAGCCACG
GAATCACTAGAGTCCGCGACAATGACAAGAGCACCCTGTACCTGCAGATGAGCAATGTGCGATCGGAGGACACAGCCAC
GAGTCACTCTCAGCCGAGACAATGACAAGAGCACCCTGTACCTGCAGATGAGCAATGTGCGATCGGAGGACACAGCCAC
GTTTCACAAATTTCAGAGACAATGACAAGAGCACCCTGTACCTGCAGATGAGCAATGTGCGATCGGAGGACACAGCCAC
GATTCTATTTGTTCAGAGACAATGACAAGAGCACCCTGTACCTGCAGATGAGCAATGTGCGATCGGAGGACACAGCCAC
GATTCAATTAGTTCAGAGACAATGACAAGAGCACCCTGTACCTGCAGATGAGCAATGTGCGATCGGAGGACACAGCCAC
GATTCCCTCAGTTCAGAGACAATGACAAGAGCACCCTGTACCTGCAGATGAGCAATGTGCGATCGGAGGACACAGCCAC
GTTTACCTTTCTTCAGAGACAATGACAAGAGCACCCTGTACCTGCAGATGAGCAATGTGCGATCGGAGGACACAGCCACG
GATTAAAGTTCTTCAGAGACAATGACAAGAGCACCCTGTACCTGCAGATGAGCAATGTGCGATCGGAGGACACAGCCAC
GATGCTCATTCTTCAGAGACAATGACAAGAGCACCCTGTACCTGCAGATGAGCAATGTGCGATCGGAGGACACAGCCAC
GATTATCATTCTTCAGAGACAATGACAAGAGCACCCTGTACCTGCAGATGAGCAATGTGCGATCGGAGGACACAGCCAC
TATTTACATTCTTCAGAGACAATGACAAGAGCACCCTGTACCTGCAGATGAGCAATGTGCGATCGGAGGACACAGCCACG
GTTGCTCTCTCTTCAGAGACAATGACAAGAGCACCCTGTACCTGCAGATGAGCAATGTGCGATCGGAGGACACAGCCACG
GAATATCTCTCTTCAGAGACAATGACAAGAGCACCCTGTACCTGCAGATGAGCAATGTGCGATCGGAGGACACAGCCAC
GAATCTATCTCTTCAGAGACAATGACAAGAGCACCCTGTACCTGCAGATGAGCAATGTGCGATCGGAGGACACAGCCAC

GAGTCAGACTCTTCAGAGACAATGACAAGAGCACCCTGTACCTGCAGATGAGCAATGTGCGATCGGAGGACACAGCCAC
GCATCTATATCTTCAGAGACAATGACAAGAGCACCCTGTACCTGCAGATGAGCAATGTGCGATCGGAGGACACAGCCAC
GAGTCATGATCTTCAGAGACAATGACAAGAGCACCCTGTACCTGCAGATGAGCAATGTGCGATCGGAGGACACAGCCAC
TATTAAGAATCTTCAGAGACAATGACAAGAGCACCCTGTACCTGCAGATGAGCAATGTGCGATCGGAGGACACAGCCAC
GTTAGACAATCTTCAGAGACAATGACAAGAGCACCCTGTACCTGCAGATGAGCAATGTGCGATCGGAGGACACAGCCAC
TAGTAACAATCTTCAGAGACAATGACAAGAGCACCCTGTACCTGCAGATGAGCAATGTGCGATCGGAGGACACAGCCAC
GATACCAAATCTTCAGAGACAATGACAAGAGCACCCTGTACCTGCAGATGAGCAATGTGCGATCGGAGGACACAGCCAC
GTTTCTCTTGCTTCAGAGACAATGACAAGAGCACCCTGTACCTGCAGATGAGCAATGTGCGATCGGAGGACACAGCCAC
GCTTCCCTTGCTTCAGAGACAATGACAAGAGCACCCTGTACCTGCAGATGAGCAATGTGCGATCGGAGGACACAGCCAC
GATGCCCAAGCTTCAGAGACAATGACAAGAGCACCCTGTACCTGCAGATGAGCAATGTGCGATCGGAGGACACAGCCAC
CATGCACAAGCTTCAGAGACAATGACAAGAGCACCCTGTACCTGCAGATGAGCAATGTGCGATCGGAGGACACAGCCAC
GATTATCTTACTTCAGAGACAATGACAAGAGCACCCTGTACCTGCAGATGAGCAATGTGCGATCGGAGGACACAGCCAC
GATGCAGAAACTTCAGAGACAATGACAAGAGCACCCTGTACCTGCAGATGAGCAATGTGCGATCGGAGGACACAGCCA
GTTTCTCTTTATTCAGAGACAATGACAAGAGCACCCTGTACCTGCAGATGAGCAATGTGCGATCGGAGGACACAGCCAC
GATTCTCATTATTCAGAGACAATGACAAGAGCACCCTGTACCTGCAGATGAGCAATGTGCGATCGGAGGACACAGCCAC
GATTCAATTGATTCAGAGACAATGACAAGAGCACCCTGTACCTGCAGATGAGCAATGTGCGATCGGAGGACACAGCCAC
GATTCCATAGATTCAGAGACAATGACAAGAGCACCCTGTACCTGCAGATGAGCAATGTGCGATCGGAGGACACAGCCAC
GAATCTCTATAGTCAGAGACAATGACAAGAGCACCCTGTACCTGCAGATGAGCAATGTGCGATCGGAGGACACAGCCAC
GATTCACAACGATCAGAGACAATGACAAGAGCACCCTGTACCTGCAGATGAGCAATGTGCGATCGGAGGACACAGCCAC
GAGTCACACTCATCAGAGACAATGACAAGAGCACCCTGTACCTGCAGATGAGCAATGTGCGATCGGAGGACACAGCCAC
GATTCCATAGCATCAGAGACAATGACAAGAGCACCCTGTACCTGCAGATGAGCAATGTGCGATCGGAGGACACAGCCAC
GATTCAATCTAATCAGAGACAATGACAAGAGCACCCTGTACCTGCAGATGAGCAATGTGCGATCGGAGGACACAGCCAC
GAATCCATAGCTGCAGAGACAATGACAAGAGCACCCTGTACCTGCAGATGAGCAATGTGCGATCGGAGGACACAGCCA
GATGCAATAGCAGCAGAGACAATGACAAGAGCACCCTGTACCTGCAGATGAGCAATGTGCGATCGGAGGACACAGCCA
GAATAACTAGAGTAAGAGACAATGACAAGAGCACCCTGTACCTGCAGATGAGCAATGTGCGATCGGAGGACACAGCCA
CGATTCACTAGCTTCAGAGAAATGACAAGAGCACCCTGTACCTGCAGATGAGCAATGTGCGATCGGAGGACACAGCCAC
ATCGATTCACTATCTTCAGAGACAATGACAAGAGCACCCTGTACCTGCAGAGCAATGTGCGATCGGAGGACACAGCCAC
GCACCATCTCCAGAGACACATCTCTGAACAAATTCTTTATCCAGCTGAGCTCTCTGACTGATGAGGACACAGTCATGTACT
TGCAGGTAGACAAATCCTCCAGCACAGCCTACATGCAGCTCAGCAGCCTGACATCTGAGGACTCTGCGGTCTATTACTGT
CCTTGACTGTAGACCAATCTTCCAGCACAGCCTACATGCAGCTCAACAGCCTGACATCTGAGGACTCTGCAGTCTATTACT

GTATTTCTGTATGAGATATAGTAACTACTGGTACTTCGATGTCTGG
GCAAGACCGGGGTACTACGGTAGTAGCTACACGTTTGACTACTGG
GTGCTAGAGAAGGTGGTTACAGCTACTGGTACTTCGATGTCTGG
TTTCTGTATGAGATACGGTAGTAGCTACTGGTACTTCGATGTCTGG
ACTGTGCAGGAGACAGATCGGGCTATACGGGGTTTGCTTACTGG
GTATTTCTGTATGAGATATAGTAACTACTGGTACTTCGATGTCTGG
GCAAGATTTTATTACTACGGTAGTAGCTATGCTATGGACTACTGG
GTATTTCTGTATGAGATATAGTAACTACTGGTACTTCGATGTCTGG
GCAAGAAGATACTACGGTAGTAGCTACTGGTACTTCGATGTCTGG
GTCCCCTATTACTACGGTAGTAGGACCTGGTACTTCGATGTCTGG
ACTGTGCAGGAGACAGATATGGTTACTGGTACTTCGATGTCTGG
GTATTTCTGTATGAGATATAGTAACTACTGGTACTTCGATGTCTGG
GTATTTCTGTATGAGATATAGTAACTACTGGTACTTCGATGTCTGG
GTATTTCTGTATGAGATATAGTAACTACTGGTACTTCGATGTCTGG
GTATTTCTGTATGAGATATAGTAACTACTGGTACTTCGATGTCTGG
GTATTTCTGTATGAGATATAGTAACTACTGGTACTTCGATGTCTGG
GTATTTCTGTATGAGATATAGTAACTACTGGTACTTCGATGTCTGG
GTATTTCTGTATGAGATATAGTAACTACTGGTACTTCGATGTCTGG
GTATTTCTGTATGAGATATAGTAACTACTGGTACTTCGATGTCTGG
GTATTTCTGTATGAGATATAGTAACTACTGGTACTTCGATGTCTGG
GTATTTCTGTATGAGATATAGTAACTACTGGTACTTCGATGTCTGG
GTATTTCTGTATGAGATATAGTAACTACTGGTACTTCGATGTCTGG
GTATTTCTGTATGAGATATAGTAACTACTGGTACTTCGATGTCTGG
TGTGCAAGTAGATGGTTACTACGTGGGGGTGCTATGGACTACTGG
GGTCTATTACTCTGTCAAGGCCCGTCTTAGGTACTTCGATGTCTGG
AGCTGAAGACACTGGCATTTATTACTGTACCCTACTATACTACTGG
GTATTTCTGTATGAGATATAGTAACTACTGGTACTTCGATGTCTGG
GTACTACTGTGCCGGTCACATAAGGAGTGAAGACTTTGCTTACTGG
GCAAGAACAGACTACTATGGTAACTACGATGCTATGGACTACTGG
GTATTTCTGTATGAGATATAGTAACTACTGGTACTTCGATGTCTGG
GTATTTCTGTATGAGATATAGTAACTACTGGTACTTCGATGTCTGG
GTATTTCTGTATGAGATATAGTAACTACTGGTACTTCGATGTCTGG
GTATTTCTGTATGAGATATAGTAACTACTGGTACTTCGATGTCTGG
GTATTTCTGTATGAGATATAGTAACTACTGGTACTTCGATGTCTGG
GTATTTCTGTATGAGATATAGTAACTACTGGTACTTCGATGTCTGG
GTATTTCTGTATGAGATATAGTAACTACTGGTACTTCGATGTCTGG
GCAAGACCGGGGTACTACGGTAGTAGCTACACGTTTGACTACTGG
GTATTTCTGTATGAGATATAGTAACTACTGGTACTTCGATGTCTGG
CGTATTTCTGTATGAGATATAGTAACTACTGGTACTTCGATGTCTGG
GTATTTCTGTATGAGATATAGTAACTACTGGTACTTCGATGTCTGG
GTATTTCTGTATGAGATATAGTAACTACTGGTACTTCGATGTCTGG
GTATTTCTGTATGAGATATAGTAACTACTGGTACTTCGATGTCTGG
GTATTTCTGTATGAGATATAGTAACTACTGGTACTTCGATGTCTGG
GTATTTCTGTATGAGATATAGTAACTACTGGTACTTCGATGTCTGG
GTATTTCTGTATGAGATATAGTAACTACTGGTACTTCGATGTCTGG
GTATTTCTGTATGAGATATAGTAACTACTGGTACTTCGATGTCTGG
CGTATTTCTGTATGAGATATAGTAACTACTGGTACTTCGATGTCTGG
GTATTTCTGTATGAGATATAGTAACTACTGGTACTTCGATGTCTGG
GTATTTCTGTATGAGATATAGTAACTACTGGTACTTCGATGTCTGG

GTATTTCTGTATGAGATATAGTAACTACTGGTACTTCGATGTCTGG
GTATTTCTGTATGAGATATAGTAACTACTGGTACTTCGATGTCTGG
GTATTTCTGTATGAGATATAGTAACTACTGGTACTTCGATGTCTGG
GTATTTCTGTATGAGATATAGTAACTACTGGTACTTCGATGTCTGG
GTATTTCTGTATGAGATATAGTAACTACTGGTACTTCGATGTCTGG
GTATTTCTGTATGAGATATAGTAACTACTGGTACTTCGATGTCTGG
GTATTTCTGTATGAGATATAGTAACTACTGGTACTTCGATGTCTGG
GTATTTCTGTATGAGATATAGTAACTACTGGTACTTCGATGTCTGG
GTATTTCTGTATGAGATATAGTAACTACTGGTACTTCGATGTCTGG
AGCTGAAGACACTGGCATTTATTACTGTACCCTACTATACTACTGG
GTATTTCTGTATGAGATATAGTATCTACTGGTACTTCGTTGTCTGG
TTTCTGTATGAGATACGGTAGTAGCTACTGGTACTTCGATGTCTGG
TATTTCTGTATGAGATATAGTAACTACTGGTACTTCGATGTCTGG
GTATTTCTGTATGAGATATAGTAACTACTGGTACTTCGATGTCTGG
GTATTTCTGTATGAGATATAGTAACTACTGGTACTTCGATGTCTGG
GTATTTCTGTATGAGATATAGTAACTACTGGTACTTCGATGTCTGG
CGTATTTCTGTATGAGATATAGTAACTACTGGTACTTCGATGTCTGG
GTATTTCTGTATGAGATATAGTAACTACTGGTACTTCGATGTCTGG
GTATTTCTGTATGAGATATAGTAACTACTGGTACTTCGATGTCTGG
GTATTTCTGTATGAGATATAGTAACTACTGGTACTTCGATGTCTGG
GTATTTCTGTATGAGATATAGTAACTACTGGTACTTCGATGTCTGG
GTATTTCTGTATGAGATATAGTAACTACTGGTACTTCGATGTCTGG
GTATTTCTGTATGAGATATAGTAACTACTGGTACTTCGATGTCTGG
GTATTTCTGTATGAGATATAGTAACTACTGGTACTTCGATGTCTGG
GTATTTCTGTATGAGATATAGTAACTACTGGTACTTCGATGTCTGG
GTATTTCTGTATGAGATATAGTAACTACTGGTACTTCGATGTCTGG
GTATTTCTGTATGAGATATAGTAACTACTGGTACTTCGATGTCTGG
GTATTTCTGTATGAGATATAGTAACTACTGGTACTTCGATGTCTGG
GTATTTCTGTATGAGATATAGTAACTACTGGTACTTCGATGTCTGG
GTATTTCTGTATGAGATATAGTAACTACTGGTACTTCGATGTCTGG
GTATTTCTGTATGAGATATAGTAACTACTGGTACTTCGATGTCTGG
GTATTTCTGTATGAGATATAGTAACTACTGGTACTTCGATGTCTGG
GTATTTCTGTATGAGATATAGTAACTACTGGTACTTCGATGTCTGG
GTATTTCTGTATGAGATATAGTAACTACTGGTACTTCGATGTCTGG
GTATTTCTGTATGAGATATAGTAACTACTGGTACTTCGATGTCTGG
CGTATTTCTGTATGAGATATAGTAACTACTGGTACTTCGATGTCTGG
GTATTTCTGTATGAGATATAGTAACTACTGGTACTTCGATGTCTGG
GTATTTCTGTATGAGATATAGTAACTACTGGTACTTCGATGTCTGG
GTATTTCTGTATGAGATATAGTAACTACTGGTACTTCGATGTCTGG
GTATTTCTGTATGAGATATAGTAACTACTGGTACTTCGATGTCTGG
CGTATTTCTGTATGAGATATAGTAACTACTGGTACTTCGATGTCTGG
GTATTTCTGTATGAGATATAGTAACTACTGGTACTTCGATGTCTGG
GTATTTCTGTATGAGATATAGTAACTACTGGTACTTCGATGTCTGG
CGTATTTCTGTATGAGATATAGTAACTACTGGTACTTCGATGTCTGG
GTATTTCTGTATGAGATATAGTAACTACTGGTACTTCGATGTCTGG
CGTATTTCTGTATGAGATATAGTAACTACTGGTACTTCGATGTCTGG
CGTATTTCTGTATGAGATATAGTAACTACTGGTACTTCGATGTCTGG
GTATTTCTGTATGAGATATAGTAACTACTGGTACTTCGATGTCTGG
GTATTTCTGTATGAGATATAGTAACTACTGGTACTTCGATGTCTGG
GTATTTCTGTATGAGATATAGTAACTACTGGTACTTCGATGTCTGG

GTATTTCTGTATGAGATATAGTAACTACTGGTACTTCGATGTCTGG
TATTTCTGTATGAGATATAGTAACTACTGGTACTTCGATGTCTGG
TATTTCTGTATGAGATATAGTAACTACTGGTACTTCGATGTCTGG
GTATTTCTGTATGAGATATAGTAACTACTGGTACTTCGATGTCTGG
TATTTCTGTATGAGATATAGTAACTACTGGTACTTCGATGTCTGG
GTATTTCTGTATGAGATATAGTAACTACTGGTACTTCGATGTCTGG
GTATTTCTGTATGAGATATAGTAACTACTGGTACTTCGATGTCTGG
TATTTCTGTATGAGATATAGTAACTACTGGTACTTCGATGTCTGG
GTATTTCTGTATGAGATATAGTAACTACTGGTACTTCGATGTCTGG
GTATTTCTGTATGAGATATAGTAACTACTGGTACTTCGATGTCTGG
GTATTTCTGTATGAGATATAGTAACTACTGGTACTTCGATGTCTGG
GTATTTCTGTATGAGATATAGTAACTACTGGTACTTCGATGTCTGG
GTATTTCTGTATGAGATATAGTAACTACTGGTACTTCGATGTCTGG
GTATTTCTGTATGAGATATAGTAACTACTGGTACTTCGATGTCTGG
TATTTCTGTATGAGATATAGTAACTACTGGTACTTCGATGTCTGG
TATTTCTGTATGAGATATAGTAACTACTGGTACTTCGATGTCTGG
GACCTTGATGGTTACTACTGGTATTACTATGCTATGGACTACTGG
GTGCAAGAAGGGGTCCTTACTACGCCAACTACTTTGACTACTGG
GCCATGTACTACTGTGCCAGTGGTAACTATGCTATGGACTACTGG
TATTACTGCACAGTGAAGGGTGCCCTGTACTACTTTGACTACTGG
TATTTCTGTATGAGATATAGTAACTACTGGTACTTCGATGTCTGG
GTATTTCTGTATGAGATATAGTAACTACTGGTACTTCGATGTCTGG
TATTTCTGTATGAGATATAGTAACTACTGGTACTTCGATGTCTGG
GTGCTAGAGAAGGTGGTTACAGCTACTGGTACTTCGATGTCTGG
TATTTCTGTATGAGATATAGTAACTACTGGTACTTCGATGTCTGG
TATTTCTGTATGAGATATAGTAACTACTGGTACTTCGATGTCTGG
TATTTCTGTATGAGATATAGTAACTACTGGTACTTCGATGTCTGG
TATTTCTGTATGAGATATAGTAACTACTGGTACTTCGATGTCTGG
TATTTCTGTATGAGATATAGTAACTACTGGTACTTCGATGTCTGG
TATTTCTGTATGAGATATAGTAACTACTGGTACTTCGATGTCTGG
GTATTTCTGTATGAGATATAGTAACTACTGGTACTTCGATGTCTGG
GTATTTCTGTATGAGATATAGTAACTACTGGTACTTCGATGTCTGG
TATTTCTGTATGAGATATAGTTACTTCTGGTACTTCGATGTCTGG
TATTTCTGTATGAGATATAGTAACTACTGGTACTTCGATGTCTGG
TATTTCTGTATGAGATATAGTAACTACTGGTACTTCGATGTCTGG
TATTTCTGTATGAGATATAGTAACTACTGGTACTTCGATGTCTGG
GTATTTCTGTATGAGATATAGTAACTACTGGTACTTCGATGTCTGG
GTATTTCTGTATGAGATATAGTAACTACTGGTACTTCGATGTCTGG
TATTTCTGTATGAGATATAGTAACTACTGGTACTTCGATGTCTGG
TATTTCTGTATGAGATATAGTAACTACTGGTACTTCGATGTCTGG
GTATTTCTGTATGAGATATAGTAACTACTGGTACTTCGATGTCTGG
TATTTCTGTATGAGATATAGTAACTACTGGTACTTCGATGTCTGG
TATTTCTGTATGAGATATAGTAACTACTGGTACTTCGATGTCTGG
GTATTTCTGTATGAGATATAGTAACTACTGGTACTTCGATGTCTGG
TATTTCTGTATGAGATATAGTAACTACTGGTACTTCGATGTCTGG
TATTTCTGTATGAGATATAGTAACTACTGGTACTTCGATGTCTGG
TATTTCTGTATGAGATATAGTAACTACTGGTACTTCGATGTCTGG
TATTTCTGTATGAGATATAGTAACTACTGGTACTTCGATGTCTGG
TATTTCTGTATGAGATATAGTAACTACTGGTACTTCGATGTCTGG
TATTTCTGTATGAGATATAGTAACTACTGGTACTTCGATGTCTGG

GTATTTCTGTATGAGATATAGTAACTACTGGTACTTCGATGTCTGG
GTATTTCTGTATGAGATATAGTAACTACTGGTACTTCGATGTCTGG
GTATTTCTGTATGAGATATAGTAACTACTGGTACTTCGATGTCTGG
GTATTTCTGTATGAGATATAGTAACTACTGGTACTTCGATGTCTGG
GTATTTCTGTATGAGATATAGTAACTACTGGTACTTCGATGTCTGG
GTATTTCTGTATGAGATATAGTAACTACTGGTACTTCGATGTCTGG
GTATTTCTGTATGAGATATAGTAACTACTGGTACTTCGATGTCTGG
GTATTTCTGTATGAGATATAGTAACTACTGGTACTTCGATGTCTGG
GTATTTCTGTATGAGATATAGTAACTACTGGTACTTCGATGTCTGG
GTATTTCTGTATGAGATATAGTAACTACTGGTACTTCGATGTCTGG
GTATTTCTGTATGAGATATAGTAACTACTGGTACTTCGATGTCTGG
GTATTTCTGTATGAGATATAGTAACTACTGGTACTTCGATGTCTGG
CGTATTTCTGTATGAGATATAGTAACTACTGGTACTTCGATGTCTGG
GTATTTCTGTATGAGATATAGTAACTACTGGTACTTCGATGTCTGG
GTATTTCTGTATGAGATATAGTAACTACTGGTACTTCGATGTCTGG
GTATTTCTGTATGAGATATAGTAACTACTGGTACTTCGATGTCTGG
GTATTTCTGTATGAGATATAGTAACTACTGGTACTTCGATGTCTGG
GTATTTCTGTATGAGATATAGTAACTACTGGTACTTCGATGTCTGG
GTATTTCTGTATGAGATATAGTAACTACTGGTACTTCGATGTCTGG
GTATTTCTGTATGAGATATAGTAACTACTGGTACTTCGATGTCTGG
GTATTTCTGTATGAGATATAGTAACTACTGGTACTTCGATGTCTGG
GTATTTCTGTATGAGATATAGTAACTACTGGTACTTCGATGTCTGG
GTATTTCTGTATGAGATATAGTAACTACTGGTACTTCGATGTCTGG
CGTATTTCTGTATGAGATATAGTAACTACTGGTACTTCGATGTCTGG
CGTATTTCTGTATGAGATATAGTAACTACTGGTACTTCGATGTCTGG
GTATTTCTGTATGAGATATAGTAACTACTGGTACTTCGATGTCTGG
GTATTTCTGTATGAGATATAGTAACTACTGGTACTTCGATGTCTGG
ACTGTTCTAGGGACGATCCTGTGCGTGAAGGCTTTGCTTACTGG
GCAAGACCGGGGTACTACGGTAGTAGCTACACGTTTGACTACTGG
GTGCAAGAAGGGGTCCTTACTACGCCAACTACTTTGACTACTGG

sample_name	templates	productive_frequency	aaSeqCDR3
TCL1_347	171688	99.775	CMRYGNYWYFDVW
TCL1_347	197	0.114	CARLLRYYWYFDVW
TCL1_347	81	0.047	CAGDRWGYWYFDVW
TCL1_347	49	0.028	CMRNGYYYWYFDVW
TCL1_347	29	0.017	CARLGRGWYFDVW
TCL1_347	5	0.003	CAGDITTVVATWSYWYFDVW
TCL1_347	4	0.002	CARDYDGYYAMDYW
TCL1_347	4	0.002	CARTYEGNYFDYW
TCL1_347	2	0.001	CVRDTIYYYNSSYKDYAMDYW
TCL1_347	1	0.001	CARHYYGSSYVDWYFDVW
TCL1_347	1	0.001	CARIYYYGSSPYYFDYW
TCL1_347	1	0.001	CASYYGSSNWYFDVW
TCL1_347	1	0.001	CARYYGSSSWYFDVW
TCL1_347	1	0.001	CARGRGLGTWYFDVW
TCL1_347	1	0.001	CARDSGSYNWYFDVW
TCL1_347	1	0.001	CARSISWGTYFDYW
TCL1_347	1	0.001	CARRYDSYWYFDVW
TCL1_347	1	0.001	CAREHGTYWYFDVW
TCL1_347	1	0.001	CMRYGNYYWYFDVW
TCL1_347	1	0.001	CARLLRYYWYFEVW
TCL1_347	1	0.001	CARQSRPGYFDYW
TCL1_347	1	0.001	CASGHPRFAYW
TCL1_347	1	0.001	CAREGSSFDYW
TCL1_347	1	0.001	CARLNYFDYW
TCL1_347	1	0.001	CVYWYFDVW
TCL1_347	1	0.001	CVRHYYIW
total	172076	100	

clonalSequence
TGTATGAGATATGGTAACTACTGGTACTTCGATGTCTGG
TGTGCAAGATTACTACGGTACTACTGGTACTTCGATGTCTGG
TGTGCAGGAGACAGATGGGGCTACTGGTACTTCGATGTCTGG
TGTATGAGAAATGGTTACTACTACTGGTACTTCGATGTCTGG
TGTGCAAGACTGGGACGTGGGTGGTACTTCGATGTCTGG
TGTGCAGGAGACATTACTACGGTAGTAGCTACGTGGAGCTACTGGTACTTCGATGTCTGG
TGTGCAAGAGACTATGATGGTTACTATGCTATGGACTACTGG
TGTGCAAGAACCTATGAGGGGAACTACTTTGACTACTGG
TGTGTGAGAGATACAATTTATTACTACAATAGTAGCTACAAAGACTATGCTATGGACTACTGG
TGTGCAAGACATTACTACGGTAGTAGCTACGTGGACTGGTACTTCGATGTCTGG
TGTGCAAGAATTTATTACTACGGTAGTAGCCCGTACTACTTTGACTACTGG
TGTGCAAGTTACTACGGTAGTAGCAACTGGTACTTCGATGTCTGG
TGTGCAAGATACTACGGTAGTAGCTCCTGGTACTTCGATGTCTGG
TGTGCAAGAGGAAGAGGCTTGGGAACCTGGTACTTCGATGTCTGG
TGTGCAAGAGATAGTGGTAGCTACAACTGGTACTTCGATGTCTGG
TGTGCAAGATCCATTTCCTGGGGGACGTACTTTGACTACTGG
TGTGCAAGACGGTATGATTCTTACTGGTACTTCGATGTCTGG
TGTGCAAGAGAACACGGTACCTACTGGTACTTCGATGTCTGG
TGTATGAGATATGGTAACTACTACTGGTACTTCGATGTCTGG
TGCGCAAGATTACTACGGTACTACTGGTACTTCGAAGTCTGG
TGTGCAAGACAAAGCCGCCCCGGCTACTTTGACTACTGG
TGTGCCAGTGGGCACCCTCGGTTTGCTTACTGG
TGTGCAAGAGAGGGTAGTAGCTTTGACTACTGG
TGTGCAAGACTTAACTACTTTGACTACTGG
TGTGTTTACTGGTACTTCGATGTCTGG
TGTGTGAGACACTACTATATCTGG

sample_name	productive_frequency	templates	amino_acid
TCL_D22_3	93.576	70521	CMRYSNYWYFDVW
TCL_D22_3	5.525	4164	CARNGNYAMDYW
TCL_D22_3	0.519	391	CAGDYDGYWYFDVW
TCL_D22_3	0.200	151	CALLRYW
TCL_D22_3	0.085	64	CANWDWYFDVW
TCL_D22_3	0.005	4	CMRYSNYWYFDVW
TCL_D22_3	0.003	2	CAPIYYGWAWFAYW
TCL_D22_3	0.003	2	CATLLICRLRYFDVW
TCL_D22_3	0.003	2	CMRYSNYWYFDVW
TCL_D22_3	0.003	2	CMRYSNYWYFDVW
TCL_D22_3	0.003	2	CMRYSNYWYFDVW
TCL_D22_3	0.003	2	CMRYSNYWYFDVW
TCL_D22_3	0.003	2	CMRYSNYWYFDVW
TCL_D22_3	0.003	2	CMRYSNYWYFDVW
TCL_D22_3	0.003	2	CMRYSNYWYFDVW
TCL_D22_3	0.003	2	CMRYSNYWYFDVW
TCL_D22_3	0.003	2	CMRYSNYWYFDVW
TCL_D22_3	0.003	2	CMRYSNYWYFDVW
TCL_D22_3	0.001	1	EDFAYW
TCL_D22_3	0.001	1	CPQSPLRLRYFDVW
TCL_D22_3	0.001	1	FPRLRYFDVW
TCL_D22_3	0.001	1	VMIHHLPLRYFDVW
TCL_D22_3	0.001	1	CAGEDFAYW
TCL_D22_3	0.001	1	CARNGNYAMDYW
TCL_D22_3	0.001	1	CARNGNYAMDYW
TCL_D22_3	0.001	1	CARNGNYAMDYW
TCL_D22_3	0.001	1	CARNGNYAMDYW
TCL_D22_3	0.001	1	CARKVGSSLCGFAYW
TCL_D22_3	0.001	1	CARNGNYAMDYW
TCL_D22_3	0.001	1	CARNGNYAMDYW
TCL_D22_3	0.001	1	CARNGNYAMDYW
TCL_D22_3	0.001	1	CARNGNYAMDYW
TCL_D22_3	0.001	1	CAYDRSTRLRYFDVW
TCL_D22_3	0.001	1	CAKLGCYW
TCL_D22_3	0.001	1	CMRYSNYWYFDVW
TCL_D22_3	0.001	1	CMRYSNYWYFDVW
TCL_D22_3	0.001	1	CMRYSNYWYFDVW
TCL_D22_3	0.001	1	CMRYSNYWYFDVW
TCL_D22_3	0.001	1	CMRYSNYWYFDVW
TCL_D22_3	0.001	1	CMRYSNYWYFDVW
TCL_D22_3	0.001	1	CMRYSNYWYFDVW
TCL_D22_3	0.001	1	CMRYSNYWYFDVW
TCL_D22_3	0.001	1	CMRYSNYWYFDVW
TCL_D22_3	0.001	1	CMRYSNYWYFDVW
TCL_D22_3	0.001	1	CMRYSNYWYFDVW
TCL_D22_3	0.001	1	CMRYSNYWYFDVW
TCL_D22_3	0.001	1	CMRYSNYWYFDVW
TCL_D22_3	0.001	1	CMRYSNYWYFDVW
TCL_D22_3	0.001	1	CMRYSNYWYFDVW

TCL_D22_3	0.001	1	CMRYSNYWYFDVW
TCL_D22_3	0.001	1	CMRYSNYWYFDVW
TCL_D22_3	0.001	1	CMRYSNYWYFDVW
TCL_D22_3	0.001	1	CMRYSNYWYFDVW
TCL_D22_3	0.001	1	CMRYSNYWYFDVW
TCL_D22_3	0.001	1	CMRYSNYWYFDVW
TCL_D22_3	0.001	1	CMRYSNYWYFDVW
TCL_D22_3	0.001	1	CAGQQREDFAYW
TCL_D22_3	0.001	1	CAGRLIMQPLRYFDVW
TCL_D22_3	0.001	1	CAGDYDGYWYFDVW
TCL_D22_3	0.001	1	CSETRLYSMYFDVW
TCL_D22_3	0.001	1	CSETRLYSRYFDVW
total	100.000	75362	

rearrangement

GATTCACTATCTTCAGAGACAATGACAAGAGCACCCTGTACCTGCAGATGAGCAATGTGCGATCGGAGGACACAGCCAC
CCAGACTGAGCATCAGCAAGGACAATTCCAAGAGCCAAGTTTTCTTTAAAATGAACAGTCTGCAAGCTGATGACACAGCC
TCTCCATTACTAGAGAAACGTCAAAGAACCAGTTCTTCCTCCAATTGAACTCTGTGACCACAGAGGACACAGCCATGTATT
ATGAGAAGTTCAAGAGCAAGGCCACACTGACTGTAGACACATCCTCCAGCACAGCCTACATGCAGCTCAGCAGCCTGACA
AGGACAAGGCCACATTGACTGCAGACAAATCCTCCAGCACAGCCTACATGCAACTGAGCAGCCTGACATCTGAGGACTCT
CGATTCACTATCTTCAGAGACAATGACAAGAGCACCTGTACCTGCAGATGAGCAATGTGCGATCGGAGGACACAGCCAC
CCACACTGACTGCAGACAAATCCTCCAGCACAGCGTACATGGAGCTCCGCAGCCTGACATCTGAGGACTCTGCGGTCTAT
CCATCACTCATGACACATCTAAGAACCATTTCTTCCTGAAGTTGAATTCTGTGACTACTGAGGACACAGCCACATATTACT
CGATTCACTATCTTCAGAGACATGACAAGAGCACCCTGTACCTGCAGATGAGCAATGTGCGATCGGAGGACACAGCCAC
CGATTCACTATCTCAGAGACAATGACAAGAGCACCCTGTACCTGCAGATGAGCAATGTGCGATCGGAGGACACAGCCAC
CGATCACTATCTTCAGAGACAATGACAAGAGCACCCTGTACCTGCAGATGAGCAATGTGCGATCGGAGGACACAGCCAC
CGATTCACTATCTTCAGAGAAATGACAAGAGCACCCTGTACCTGCAGATGAGCAATGTGCGATCGGAGGACACAGCCAC
CGATTCACTATCTTCAGAGACAATGACAGAGCACCCTGTACCTGCAGATGAGCAATGTGCGATCGGAGGACACAGCCAC
CGATTCACTATCTTCAGAGACAATGACAAGAGACCCTGTACCTGCAGATGAGCAATGTGCGATCGGAGGACACAGCCAC
CGATTCACTATCTTCAGAGACAATGACAAGAGCACCCTGTACCGCAGATGAGCAATGTGCGATCGGAGGACACAGCCAC
CGATTCACTATCTTCAGAGACAATGACAAGAGCACCCTGTACTGCAGATGAGCAATGTGCGATCGGAGGACACAGCCAC
ATTCACTATCTTCAGAGACAATGACAAGAGCACCCCTGTACCTGCAGATGAGCAATGTGCGATCGGAGGACACAGCCAC
CGATTCACTATCTTCAGAGACAATGACAAGAGCACCCGTACCTGCAGATGAGCAATGTGCGATCGGAGGACACAGCCAC
ACAACCAGAAGTTCAAGGGCAAGGCCACATTGACTGTAGACAAGTCCTCCAGCACAGCCTACATGGAGCTCCGCAGCCT
CCACACTGACTGCAGACACATCCTCCAGCACAGCCTACATGCAGCTCAGCAGCCTGACATCTGAGGACTCTGCAGTCTATT
TCAAAGGCAAGGCCACACTAACAGAACACAAATCCTCCAGCACAGCCTACATAGAGCTTAGCAACCTGACCTCTGAGGAC
CCACACTGACTGCAGACAAATCCTCCAGCACAGCCTACATGCAGCTCAGCAGCCTGACATCTGAGGACTCTGCAGTCTAT
AGTTCAAGGGCAAGGCCACACTGACTGCAGACAAATCCTCCAGCACAGCCTACATGCAGCTCAGCAGCCTGACATCTGA
TCCAGACTGAGCATCAGCAAGGACAATTCCAAGAGCCAAGTTTTCTTTAAATGAACAGTCTGCAAGCTGATGACACAGCC
ACATAATGAGCATCAGCAAGGACAATTCCAAGAGCCAAGTTTTCTTTAAAATGAACAGTCTGCAAGCTGATGACACAGCC
CCATAATTAGCATCAGCAAGGACAATTCCAAGAGCCAAGTTTTCTTTAAAATGAACAGTCTGCAAGCTGATGACACAGCC
TCCAGACTGAGCATCAGCAAGACAATTCCAAGAGCCAAGTTTTCTTTAAAATGAACAGTCTGCAAGCTGATGACACAGCC
GCATCAGCAAGGACAATTCCAAGAGCCAAGTTTTCTTTAAAATGAACAGTCTGCAAGCTGATGACACAGCCATATATTACT
TCCAGACTGAGCATCAGCAAGGACAATTCCAAGAGCCAAGTTTTCTTAAAATGAACAGTCTGCAAGCTGATGACACAGCC
CAGACTGAGCATCAGCAAGGACAATTCCAAGAGCCAAGTTTTCTTTAAAAATGAACAGTCTGCAAGCTGATGACACAGCC
TCCAGACTGAGCATCAGCAAGGACAATTCAAGAGCCAAGTTTTCTTTAAAATGAACAGTCTGCAAGCTGATGACACAGCC
TCCAGACTGAGCATCAGCAAGGACAATTCCAAGAGCCAAGTTTCTTTAAAATGAACAGTCTGCAAGCTGATGACACAGCC
GCATCAGCAAGGACAACTCCAAGAGCCAAATTTTCTTTAAAATGAACAGTCTGCAAGCTGATGACACTGCCATATACTACT
CAGCTCTCATGTCCAGACTGAGCATCAGCAAAGACAACTCCAAGAGCCAAGTTTTCTTAAAAATGAACAGTCTGCAAACT
TCGATTCACTATCTTCAGACAATGACAAGAGCACCCTGTACCTGCAGATGAGCAATGTGCGATCGGAGGACACAGCCAC
GATTCCATTTCTTCAGAGACAATGACAAGAGCACCCTGTACCTGCAGATGAGCAATGTGCGATCGGAGGACACAGCCAC
GATTCAGATTCTTCAGAGACAATGACAAGAGCACCCTGTACCTGCAGATGAGCAATGTGCGATCGGAGGACACAGCCAC
CGATTACTATCTTCAGAGACAATGACAAGAGCACCCTGTACCTGCAGATGAGCAATGTGCGATCGGAGGACACAGCCAC
CGATTCACTATCTTCAGAGACAATGACAAGAGCACCCTGACCTGCAGATGAGCAATGTGCGATCGGAGGACACAGCCAC
CGATTCACTATCTTCAGAGACAATGACAAGAGCACCCTGTACCTGAGATGAGCAATGTGCGATCGGAGGACACAGCCAC
CGATTCACTATCTTCAGAGACAATGACAAGAGCACCCTGTACCTCAGATGAGCAATGTGCGATCGGAGGACACAGCCAC
CGATTCACTATCTTCAGAGACAATGACAAGAGCACCCTGTCCTGCAGATGAGCAATGTGCGATCGGAGGACACAGCCAC
GATTCCAAATCTTCAGAGACAATGACAAGAGCACCCTGTACCTGCAGATGAGCAATGTGCGATCGGAGGACACAGCCAC
CGATTCACTATCTTCAGAGACAAGACAAGAGCACCCTGTACCTGCAGATGAGCAATGTGCGATCGGAGGACACAGCCAC
CGATTCACTATCTTCAGAGACAATGAAAGAGCACCCTGTACCTGCAGATGAGCAATGTGCGATCGGAGGACACAGCCAC
ATCGATTCACTATCTTCAGAGACAATGAGAGCACCCTGTACCTGCAGATGAGCAATGTGCGATCGGAGGACACAGCCAC
CGATTCACTATCTTCGAGACAATGACAAGAGCACCCTGTACCTGCAGATGAGCAATGTGCGATCGGAGGACACAGCCAC
CGATTCACTATCTTAGAGACAATGACAAGAGCACCCTGTACCTGCAGATGAGCAATGTGCGATCGGAGGACACAGCCAC
TAGTCCCTATCTTCAGAGACAATGACAAGAGCACCCTGTACCTGCAGATGAGCAATGTGCGATCGGAGGACACAGCCAC

CGATAACTATCTTCAGAGACAATGACAAGAGCACCCTGTACCTGCAGATGAGCAATGTGCGATCGGAGGACACAGCCAC
CGATTCACATCTTCAGAGACAATGACAAGAGCACCCTGTACCTGCAGATGAGCAATGTGCGATCGGAGGACACAGCCAC
GATGAAATAGCTTCAGAGACAATGACAAGAGCACCCTGTACCTGCAGATGAGCAATGTGCGATCGGAGGACACAGCCAC
TATTAACTTACTTCAGAGACAATGACAAGAGCACCCTGTACCTGCAGATGAGCAATGTGCGATCGGAGGACACAGCCACG
TCGATTCACTATCTTCAGAGACAATGACAAGCACCCTGTACCTGCAGATGAGCAATGTGCGATCGGAGGACACAGCCACG
TCGATTCACTATCTTCAGAGACAATGAAGAGCACCCTGTACCTGCAGATGAGCAATGTGCGATCGGAGGACACAGCCACG
CGATTCACTATCTTCAGAGACAATACAAGAGCACCCTGTACCTGCAGATGAGCAATGTGCGATCGGAGGACACAGCCACG
GCCCCATCTCCATTACTAGAGAAACGTCAAAGAACCAGTTCTTCCTCCAATTGAACTCTGTGACCACAGAGGACACAGCCA
TTACTAGAGAAACGTCAAAGAACCAGTTCTTCCTCCAATTGAACTCTGTGACCACAGAGGACACAGCCATGTATTACTGTG
ATCTCCATACTAGAGAAACGTCAAAGAACCAGTTCTTCCTCCAATTGAACTCTGTGACCACAGAGGACACAGCCATGTATT
TCGCCATTTCAAGAGATGATTCAAAAAGCAGTGTCTACCTAGAGATGAACAGATTAAGAGAGGAAGACACTGCCACTTAT
TCGCCATTTCAAGAGATGATTCAAAAAGCAGTGTCTACCTAGAGATGAACAGATTAAGAGAGGAAGACACTGCCACTTAT

TATTTCTGTATGAGATATAGTAACTACTGGTACTTCGATGTCTGG
ATATATTACTGTGCCAGAAATGGTAACTATGCTATGGACTACTGG
ACTGTGCAGGAGACTATGATGGTTACTGGTACTTCGATGTCTGG
TCTGAGGACTCTGCGGTCTATTACTGTGCATTACTACGGTACTGG
GCAGTCTATTACTGTGCAAACTGGGACTGGTACTTCGATGTCTGG
TATTTCTGTATGAGATATAGTAACTACTGGTACTTCGATGTCTGG
TTCTGTGCACCCATCTACTATGGTTGGGCCTGGTTTGCTTACTGG
TGCAACGTTATTGATTTGCCGTCTTAGGTACTTCGATGTCTGG
TATTTCTGTATGAGATATAGTAACTACTGGTACTTCGATGTCTGG
TATTTCTGTATGAGATATAGTAACTACTGGTACTTCGATGTCTGG
TATTTCTGTATGAGATATAGTAACTACTGGTACTTCGATGTCTGG
TATTTCTGTATGAGATATAGTAACTACTGGTACTTCGATGTCTGG
TATTTCTGTATGAGATATAGTAACTACTGGTACTTCGATGTCTGG
TATTTCTGTATGAGATATAGTAACTACTGGTACTTCGATGTCTGG
TATTTCTGTATGAGATATAGTAACTACTGGTACTTCGATGTCTGG
TATTTCTGTATGAGATATAGTAACTACTGGTACTTCGATGTCTGG
TATTTCTGTATGAGATATAGTAACTACTGGTACTTCGATGTCTGG
TATTTCTGTATGAGATATAGTAACTACTGGTACTTCGATGTCTGG
ACATCTGAGGACTCTGCAGTCTATTACGAAGACTTTGCTTACTGG
TCTGTCCACAATCGCCACTCCGCCTTAGGTACTTCGATGTCTGG
TCTGCTGTCTATTACTTTCCCCGTCTTAGGTACTTCGATGTCTGG
CTGTGATGATACATCACCTGCCGCTTAGGTACTTCGATGTCTGG
GACTCTGCAGTCTATTTCTGTGCTGGTGAAGACTTTGCTTACTGG
ATATATTACTGTGCCAGAAATGGTAACTATGCTATGGACTACTGG
ATATATTACTGTGCCAGAAATGGTAACTATGCTATGGACTACTGG
TATATTACTGTGCCAGAAATGGTAACTATGCTATGGACTACTGG
ATATATTACTGTGCCAGAAATGGTAACTATGCTATGGACTACTGG
GTGCCAGAAAGGTCGGTAGTAGCCTCTGCGGGTTTGCTTACTGG
ATATATTACTGTGCCAGAAATGGTAACTATGCTATGGACTACTGG
ATATATTACTGTGCCAGAAATGGTAACTATGCTATGGACTACTGG
ATATATTACTGTGCCAGAAATGGTAACTATGCTATGGACTACTGG
ATATATTACTGTGCCAGAAATGGTAACTATGCTATGGACTACTGG
GTGCCTATGATCGTTCCACCCGTCTTAGGTACTTCGATGTCTGG
ATGACACAGCCATGTACTACTGTGCCAAACTGGGTTGCTACTGG
TATTTCTGTATGAGATATAGTAACTACTGGTACTTCGATGTCTGG
TATTTCTGTATGAGATATAGTAACTACTGGTACTTCGATGTCTGG
TATTTCTGTATGAGATATAGTAACTACTGGTACTTCGATGTCTGG
TATTTCTGTATGAGATATAGTAACTACTGGTACTTCGATGTCTGG
TATTTCTGTATGAGATATAGTAACTACTGGTACTTCGATGTCTGG
TATTTCTGTATGAGATATAGTAACTACTGGTACTTCGATGTCTGG
TATTTCTGTATGAGATATAGTAACTACTGGTACTTCGATGTCTGG
TATTTCTGTATGAGATATAGTAACTACTGGTACTTCGATGTCTGG
TATTTCTGTATGAGATATAGTAACTACTGGTACTTCGATGTCTGG
TATTTCTGTATGAGATATAGTAACTACTGGTACTTCGATGTCTGG
TATTTCTGTATGAGATATAGTAACTACTGGTACTTCGATGTCTGG
TATTTCTGTATGAGATATAGTAACTACTGGTACTTCGATGTCTGG
TATTTCTGTATGAGATATAGTAACTACTGGTACTTCGATGTCTGG
TATTTCTGTATGAGATATAGTAACTACTGGTACTTCGATGTCTGG
TATTTCTGTATGAGATATAGTAACTACTGGTACTTCGATGTCTGG

GTATTTCTGTATGAGATATAGTAACTACTGGTACTTCGATGTCTGG
GTATTTCTGTATGAGATATAGTAACTACTGGTACTTCGATGTCTGG
CGTATTTCTGTATGAGATATAGTAACTACTGGTACTTCGATGTCTGG
GTATTTCTGTATGAGATATAGTAACTACTGGTACTTCGATGTCTGG
GTATTTCTGTATGAGATATAGTAACTACTGGTACTTCGATGTCTGG
GTATTTCTGTATGAGATATAGTAACTACTGGTACTTCGATGTCTGG
GTATTTCTGTATGAGATATAGTAACTACTGGTACTTCGATGTCTGG
ATGTATTACTGTGCAGGACAGCAACGTGAAGACTTTGCTTACTGG
GCAGGACGATTGATTATGCAACCCCTTAGGTACTTCGATGTCTGG
TACTGTGCAGGAGACTATGATGGTTACTGGTACTTCGATGTCTGG
TTTTTGTAGTGAGACTCGCCTTTACTCTATGTACTTCGATGTCTGG
TTTTTGTAGTGAGACTCGCCTTTACTCTAGGTACTTCGATGTCTGG

sample_name	productive_frequency	templates	amino_acid
TCL_E31_2	99.286	93913	CAGDITTVVATWSYWYFD\
TCL_E31_2	0.260	246	CMRYGNYWYFDVW
TCL_E31_2	0.040	38	CAGDITTVVATWSYWYFD\
TCL_E31_2	0.026	25	CAKIYYYGSSYAMDYW
TCL_E31_2	0.014	13	CARIYYGNYWYFDVW
TCL_E31_2	0.007	7	CAGDITTVVATWSYWYFD\
TCL_E31_2	0.003	3	CARSRGSGYSPFAYW
TCL_E31_2	0.003	3	CAGDITTVVATWSYWYFD\
TCL_E31_2	0.003	3	CAGDITTVVATWSYWYFD\
TCL_E31_2	0.002	2	CHFQAFRLRYFDVW
TCL_E31_2	0.002	2	CAGDITTVVATWSYWYFD\
TCL_E31_2	0.002	2	CAGDITTVVATWSYWYFD\
TCL_E31_2	0.002	2	CAGDITTVVATWSYWYFD\
TCL_E31_2	0.002	2	CAGDITTVVATWSYWYFD\
TCL_E31_2	0.002	2	CAGDITTVVATWSYWYFD\
TCL_E31_2	0.002	2	CAGDITTVVATWSYWYFD\
TCL_E31_2	0.002	2	CAGDITTVVATWSYWYFD\
TCL_E31_2	0.002	2	CAGDITTVVATWSYWYFD\
TCL_E31_2	0.002	2	CAGDITTVVATWSYWYFD\
TCL_E31_2	0.001	1	CARAASEDFAYW
TCL_E31_2	0.001	1	CVTTGDRLRYFDVW
TCL_E31_2	0.001	1	CVLNTITLRYFDVW
TCL_E31_2	0.001	1	CVVLCHPSVRYFDVW
TCL_E31_2	0.001	1	CDFSSHRLRYFDVW
TCL_E31_2	0.001	1	CACVRGSEDFAYW
TCL_E31_2	0.001	1	CAGDITTVVATWSYWYFD\
TCL_E31_2	0.001	1	CAGDITTVVATWSYWYFD\
TCL_E31_2	0.001	1	CAGDITTVVATWSYWYFD\
TCL_E31_2	0.001	1	CAQRSIGEDFAYW
TCL_E31_2	0.001	1	CAGDITTVVATWSYWYFD\
TCL_E31_2	0.001	1	CAGDITTVVATWSYWYFD\
TCL_E31_2	0.001	1	CAGDITTVVATWSYWYFD\
TCL_E31_2	0.001	1	CAGDITTVVATWSYWYFD\
TCL_E31_2	0.001	1	CAGDITTVVATWSYWYFD\
TCL_E31_2	0.001	1	CAGDITTVVATWSYWYFD\
TCL_E31_2	0.001	1	CMRYGNYWYFDVW
TCL_E31_2	0.001	1	CAGDITTVVATWSYWYFD\
TCL_E31_2	0.001	1	CAGDITTVVATWSYWYFD\
TCL_E31_2	0.001	1	CAGDITTVVATWSYWYFD\
TCL_E31_2	0.001	1	CAGDITTVVATWSYWYFD\
TCL_E31_2	0.001	1	CAGDITTVVATWSYWYFD\
TCL_E31_2	0.001	1	CAGDITTVVATWSYWYFD\
TCL_E31_2	0.001	1	CAGDITTVVATWSYWYFD\
TCL_E31_2	0.001	1	CAGDITTVVATWSYWYFD\
TCL_E31_2	0.001	1	CAGDITTVVATWSYWYFD\
TCL_E31_2	0.001	1	CAGDITTVVATWSYWYFD\
TCL_E31_2	0.001	1	CAGDITTVVATWSYWYFD\
TCL_E31_2	0.001	1	CAGDITTVVATWSYWYFD\
TCL_E31_2	0.001	1	CAGDITTVVATWSYWYFD\

TCL_E31_2	0.001	1	CAGDITTVVATWSYWYFD\
TCL_E31_2	0.001	1	CAGDITTVVATWSYWYFD\
TCL_E31_2	0.001	1	CAGDITTVVATWSYWYFD\
TCL_E31_2	0.001	1	CAGDITTVVATWSYWYFD\
TCL_E31_2	0.001	1	CAGDITTVVATWSYWYFD\
TCL_E31_2	0.001	1	CAGDITTVVATWSYWYFD\
TCL_E31_2	0.001	1	CAGDITTVVATWSYWYFD\
TCL_E31_2	0.001	1	CAGDITTVVATWSYWYFD\
TCL_E31_2	0.001	1	CAGDITTVVATWSYWYFD\
TCL_E31_2	0.001	1	CAGDITTVVATWSYWYFD\
TCL_E31_2	0.001	1	CAGDITTVVATWSYWYFD\
TCL_E31_2	0.001	1	CAGDITTVVATWSYWYFD\
TCL_E31_2	0.001	1	CAGDITTVVATWSYWYFD\
TCL_E31_2	0.001	1	CAGDITTVVATWSYWYFD\
TCL_E31_2	0.001	1	CAGDITTVVATWSYWYFD\
TCL_E31_2	0.001	1	CAGDITTVVATWSYWYFD\
TCL_E31_2	0.001	1	CAGDITTVVATWSYWYFD\
TCL_E31_2	0.001	1	CAGDITTVVATWSYWYFD\
TCL_E31_2	0.001	1	CAGDITTVVATWSYWYFD\
TCL_E31_2	0.001	1	CAGDITTVVATWSYWYFD\
TCL_E31_2	0.001	1	CAGDITTVVATWSYWYFD\
TCL_E31_2	0.001	1	CAGDITTVVATWSYWYFD\
TCL_E31_2	0.001	1	CAGDITTVVATWSYWYFD\
TCL_E31_2	0.001	1	CAGDITTVVATWSYWYFD\
TCL_E31_2	0.001	1	CAGDITTVVATWSYWYFD\
TCL_E31_2	0.001	1	CAGDITTVVATWSYWYFD\
TCL_E31_2	0.001	1	CAGDITTVVATWSYWYFD\
TCL_E31_2	0.001	1	CAGDITTVVATWSYWYFD\
TCL_E31_2	0.001	1	CAGDITTVVATWSYWYFD\
TCL_E31_2	0.001	1	CAGDITTVVATWSYWYFD\
TCL_E31_2	0.001	1	CAGDITTVVATWSYWYFD\
TCL_E31_2	0.001	1	CAGDITTVATWSYWYFDV\
TCL_E31_2	0.001	1	CAGDITTVVATWSYWYFD\
TCL_E31_2	0.001	1	CAGDITSVVATWSYWYFD\
TCL_E31_2	0.001	1	CAGDITTVVATWSYWYFD\
TCL_E31_2	0.001	1	CAGDITTVVATWSYWYFD\
TCL_E31_2	0.001	1	CAGDITTVVATWSYWYFD\
TCL_E31_2	0.001	1	CAGDITTVVATWSYWYFD\
TCL_E31_2	0.001	1	CAGDITTVVATWSYWYFD\
TCL_E31_2	0.001	1	CAGDITTVVATWSYWYFD\
TCL_E31_2	0.001	1	CAGDITTVVATWSYWYFD\
TCL_E31_2	0.001	1	CAGDITTVVATWSYWYFD\
TCL_E31_2	0.001	1	CAGDITTVVATWSYWYFD\
TCL_E31_2	0.001	1	CAGDITTVVATWSYWYFD\
TCL_E31_2	0.001	1	CAGDITTVVATWSYWYFD\
TCL_E31_2	0.001	1	CAGDITTVVATWSYWYFD\
TCL_E31_2	0.001	1	CAGDITTVVATWSYWYFD\
TCL_E31_2	0.001	1	CAGDITTVVATWSYWYFD\
TCL_E31_2	0.001	1	CAGDITTVVATWSYWYFD\
TCL_E31_2	0.001	1	CAGDITTVVATWSYWYFD\

TCL_E31_2	0.001	1	CAGDITTVVATWSYWYFD\
TCL_E31_2	0.001	1	CAGDITTVVATWSYWYFD\
TCL_E31_2	0.001	1	CAGDITTVVATWSYWYFD\
TCL_E31_2	0.001	1	CAGDITTVVATWSYWYFD\
TCL_E31_2	0.001	1	CAGDITTVVATWSYWYFD\
TCL_E31_2	0.001	1	CAGDITTVVATWSYWYFD\
TCL_E31_2	0.001	1	CAGDITTVVATWSYWYFD\
TCL_E31_2	0.001	1	CAGDITTVVATWSYWYFD\
TCL_E31_2	0.001	1	CAGDITTVVATWSYWYFD\
TCL_E31_2	0.001	1	CAGDITTVVATWSYWYFD\
TCL_E31_2	0.001	1	CAGDITTVVATWSYWYFD\
TCL_E31_2	0.001	1	CAGDITTVVATWSYWYFD\
TCL_E31_2	0.001	1	CAGDITTVVATWSYWYFD\
TCL_E31_2	0.001	1	CAGDITTVVATWSYWYFD\
TCL_E31_2	0.001	1	CAGDITTVVATWSYWYFD\
TCL_E31_2	0.001	1	CAGDITTVVATWSYWYFD\
TCL_E31_2	0.001	1	CAGDITTVVATWSYWYFD\
TCL_E31_2	0.001	1	CAGDITTVVATWSYWYFD\
TCL_E31_2	0.001	1	CAGDITTVVATWSYWYFD\
TCL_E31_2	0.001	1	CAGDITTVVATWSYWYFD\
TCL_E31_2	0.001	1	CAGDITTVVATWSYWYFD\
TCL_E31_2	0.001	1	CAGDITTVVATWSYWYFD\
TCL_E31_2	0.001	1	CAGDITTVVATWSYWYFD\
TCL_E31_2	0.001	1	CAGDITTVVATWSYWYFD\
TCL_E31_2	0.001	1	CAGDITTVVATWSYWYFD\
TCL_E31_2	0.001	1	CAGDITTVVATWSYWYFD\
TCL_E31_2	0.001	1	CAGDITTVVATWSYWYFD\
TCL_E31_2	0.001	1	CAGDITTVVATWSYWYFD\
TCL_E31_2	0.001	1	CAGDITTVVATWSYWYFD\
TCL_E31_2	0.001	1	CAGDITTVVATWSYWYFD\
TCL_E31_2	0.001	1	CAGDITTVVATWSYWYFD\
TCL_E31_2	0.001	1	CAGDITTVVATWSYWYFD\
TCL_E31_2	0.001	1	CAGDITTVVATWSYWYFD\
TCL_E31_2	0.001	1	CAGDITTVVATWSYWYFD\
TCL_E31_2	0.001	1	CAGDITTVVATWSYWYFD\
TCL_E31_2	0.001	1	CAGDITTVVATWSYWYFD\
TCL_E31_2	0.001	1	CAGDITTVVATWSYWYFD\
TCL_E31_2	0.001	1	CAGDITTVVATWSYWYFD\
TCL_E31_2	0.001	1	CAGDITTVVATWSYWYFD\
TCL_E31_2	0.001	1	CAGDITTVVATWSYWYFD\
TCL_E31_2	0.001	1	CAGDITTVVATWSYWYFD\
TCL_E31_2	0.001	1	CAGDITTVVATWSYWYFD\
TCL_E31_2	0.001	1	CAGDITTVVATWSYWYFD\
TCL_E31_2	0.001	1	CAGDITTVVATWSYWYFD\
TCL_E31_2	0.001	1	CAGDITTVVATWSYWYFD\
TCL_E31_2	0.001	1	CAGDITTVVATWSYWYFD\
TCL_E31_2	0.001	1	CAGDITTVVATWSYWYFD\
TCL_E31_2	0.001	1	CAGDITTVVATWSYWYFD\
TCL_E31_2	0.001	1	CAGDITTVVATWSYWYFD\
TCL_E31_2	0.001	1	CAGDITTVVATWSYWYFD\

TCL_E31_2	0.001	1	CAGDITTVVATWSYWYFD\
TCL_E31_2	0.001	1	CAGDITTVVATWSYWYFD\
TCL_E31_2	0.001	1	CAGDITTVVATWSYWYFD\
TCL_E31_2	0.001	1	CAGDITTVVATWSYWYFD\
TCL_E31_2	0.001	1	CAGDITTVVATWSYWYFD\
TCL_E31_2	0.001	1	CAGDITTVVATWSYWYFD\
TCL_E31_2	0.001	1	CAGDITTVVATWSYWYFD\
TCL_E31_2	0.001	1	CAGDITTVVATWSYWYFD\
TCL_E31_2	0.001	1	CAGDITTVVATWSYWYFD\
TCL_E31_2	0.001	1	CAGDITTVVATWSYWYFD\
TCL_E31_2	0.001	1	CAGDITTVVATWSYWYFD\
TCL_E31_2	0.001	1	CAGDITTVVATWSYWYFD\
TCL_E31_2	0.001	1	CAGDITTVVATWSYWYFD\
TCL_E31_2	0.001	1	CAGDITTVVATWSYWYFD\
TCL_E31_2	0.001	1	CAGDITTVVATWSYWYFD\
TCL_E31_2	0.001	1	CAGDITTVVATWSYWYFD\
TCL_E31_2	0.001	1	CAGDITTVVATWSYWYFD\
TCL_E31_2	0.001	1	CAGDITTVVATWSYWYFD\
TCL_E31_2	0.001	1	CAGDITTVVATWSYWYFD\
TCL_E31_2	0.001	1	CAGDITTVVATWSYWYFD\
TCL_E31_2	0.001	1	CAGDITTVVATWSYWYFD\
TCL_E31_2	0.001	1	CAGDITTVVATWSYWYFD\
TCL_E31_2	0.001	1	CAGDITTVVATWSYWYFD\
TCL_E31_2	0.001	1	CAGDITTVVATWSYWYFD\
TCL_E31_2	0.001	1	CAGDITTVVATWSYWYFD\
TCL_E31_2	0.001	1	CAGDITTVVATWSYWYFD\
TCL_E31_2	0.001	1	CAKIYYYGSSYAMDYW
TCL_E31_2	0.001	1	CITREDFAYW
TCL_E31_2	0.001	1	CSRYSSEDFAYW
TCL_E31_2	0.001	1	CTSCRLRYFDVW
TCL_E31_2	0.001	1	CYQRVSRLRYFDVW
TCL_E31_2	0.001	1	CAGDITTVVATWSYWYFD\
TCL_E31_2	0.001	1	CAGDITTVVATWSYWYFD\
TCL_E31_2	0.001	1	CAGDITTVVATWSYWYFD\
TCL_E31_2	0.001	1	CAGDITTVVATWSYWYFD\
TCL_E31_2	0.001	1	CAGDITTVVATWSYWYFD\
TCL_E31_2	0.001	1	CAGDITTVVATWSYWYFD\
TCL_E31_2	0.001	1	CMRYGNYWYFDVW
TCL_E31_2	0.001	1	CAGDITTVVATWSYWYFD\
TCL_E31_2	0.001	1	CAGDITTVVATWSYWYFD\
TCL_E31_2	0.001	1	CAGDITTVVATWSYWYFD\
TCL_E31_2	0.001	1	CAGDITTVVATWSYWYFD\
TCL_E31_2	0.001	1	CAGDITTVVATWSYWYFD\
TCL_E31_2	0.001	1	CAGDITTVVATWSYWYFD\
TCL_E31_2	0.001	1	CAGDITTVVATWSYWYFD\
TCL_E31_2	0.001	1	CAGDITTVVATWSYWYFD\
TCL_E31_2	0.001	1	CAGDITTVVATWSYWYFD\
TCL_E31_2	0.001	1	CAGDITTVVATWSYWYFD\
TCL_E31_2	0.001	1	CAGDITTVVATWSYWYFD\
TCL_E31_2	0.001	1	CAGDITTVVATWSYWYFD\

TCL_E31_2	0.001	1	CAGDITTVVATWSYWYFD\
TCL_E31_2	0.001	1	CAGDITTVVATWSYWYFD\
TCL_E31_2	0.001	1	CAGDITTVVATWSYWYFD\
TCL_E31_2	0.001	1	CAGDITTVVATWSYWYFD\
TCL_E31_2	0.001	1	CAGDITTVVATWSYWYFD\
TCL_E31_2	0.001	1	CAGDITTVVATWSYWYFD\
TCL_E31_2	0.001	1	CAGDITTVVATWSYWYFD\
TCL_E31_2	0.001	1	CAGDITTVVATWSYWYFD\
TCL_E31_2	0.001	1	CAGDITTVVATWSYWYFD\
TCL_E31_2	0.001	1	CAGDITTVVATWSYWYFD\
TCL_E31_2	0.001	1	CAGDITTVVATWSYWYFD\
TCL_E31_2	0.001	1	CAGDITTVVATWSYWYFD\
TCL_E31_2	0.001	1	CAGDITTVVATWSYWYFD\
TCL_E31_2	0.001	1	CAGDITTVVATWSYWYFD\
TCL_E31_2	0.001	1	CAGDITTVVATWSYWYFD\
TCL_E31_2	0.001	1	CAGDITTVVATWSYWYFD\
TCL_E31_2	0.001	1	CAGDITTVVATWSYWYFD\
TCL_E31_2	0.001	1	CAGDITTVVATWSYWYFD\
TCL_E31_2	0.001	1	CAGDITTVVATWSYWYFD\
TCL_E31_2	0.001	1	CAGDITTVVATWSYWYFD\
TCL_E31_2	0.001	1	CAGDITTVVATWSYWYFD\
TCL_E31_2	0.001	1	CAGDITTVVATWSYWYFD\
TCL_E31_2	0.001	1	CAGDITTVVATWSYWYFD\
TCL_E31_2	0.001	1	CAGDITTVVATWSYWYFD\
TCL_E31_2	0.001	1	CAGDITTVVATWSYWYFD\
TCL_E31_2	0.001	1	CAGDITTVVATWSYWYFD\
TCL_E31_2	0.001	1	CAGDITTVVATWSYWYFD\
TCL_E31_2	0.001	1	CAGDITTVVATWSYWYFD\
TCL_E31_2	0.001	1	CAGDITTVVATWSYWYFD\
TCL_E31_2	0.001	1	CAGDITTVVATWSYWYFD\
TCL_E31_2	0.001	1	CAGDITTVVATWSYWYFD\
TCL_E31_2	0.001	1	CAGDITTVVATWSYWYFD\
TCL_E31_2	0.001	1	CAGDITTVVATWSYWYFD\
TCL_E31_2	0.001	1	CAGDITTVVATWSYWYFD\
TCL_E31_2	0.001	1	CAGDITTVVATWSYWYFD\
TCL_E31_2	0.001	1	CAGDITTVVATWSYWYFD\
TCL_E31_2	0.001	1	CAGDITTVVATWSYWYFD\
TCL_E31_2	0.001	1	CAGDITTVVATWSYWYFD\
TCL_E31_2	0.001	1	CAGDITTVVATWSYWYFD\
TCL_E31_2	0.001	1	CAGDITTVVATWSYWYFD\
TCL_E31_2	0.001	1	CAGDITTVVATWSYWYFD\
TCL_E31_2	0.001	1	CAGDITTVVATWSYWYFD\
TCL_E31_2	0.001	1	CAGDITTVVATWSYWYFD\
TCL_E31_2	0.001	1	CAGDITTVVATWSYWYFD\
TCL_E31_2	0.001	1	CAGDITTVVATWSYWYFD\
TCL_E31_2	0.001	1	CAGDITTVVATWSYWYFD\
TCL_E31_2	0.001	1	CAGDITTVVATWSYWYFD\
TCL_E31_2	0.001	1	CAGDITTVVATWSYWYFD\
TCL_E31_2	0.001	1	CAGDITTVVATWSYWYFD\
TCL_E31_2	0.001	1	CAGDITTVVATWSYWYFD\

TCL_E31_2	0.001	1 CAGDITTVVATWSYWYFD\
TCL_E31_2	0.001	1 CAGDITTVVATWSYWYFD\
TCL_E31_2	0.001	1 CAGDITTVVATWSYWYFD\
TCL_E31_2	0.001	1 CAGDITTVVATWSYWYFD\
TCL_E31_2	0.001	1 CAGDITTVVATWSYWYFD\
TCL_E31_2	0.001	1 CAGDITTVVATWSYWYFD\
TCL_E31_2	0.001	1 CAGDITTVVATWSYWYFD\
TCL_E31_2	0.001	1 CAGDITTVVATWSYWYFD\
TCL_E31_2	0.001	1 CAGDITTVVATWSYWYFD\
TCL_E31_2	0.001	1 CAGDITTVVATWSYWYFD\
TCL_E31_2	0.001	1 CAGDITTVVATWSYWYFD\
TCL_E31_2	0.001	1 CAGDITTVVATWSYWYFD\
TCL_E31_2	0.001	1 CAGDITTVVATWSYWYFD\
TCL_E31_2	0.001	1 CAGDITTVVATWSYWYFD\
TCL_E31_2	0.001	1 CAGDITTVVATWSYWYFD\
TCL_E31_2	0.001	1 CAGDITTVVATWSYWYFD\
TCL_E31_2	0.001	1 CAGDITTVVATWSYWYFD\
TCL_E31_2	0.001	1 CAGDITTVVATWSYWYFD\
TCL_E31_2	0.001	1 CAGDITTVVATWSYWYFD\
TCL_E31_2	0.001	1 CAGDITTVVATWSYWYFD\
TCL_E31_2	0.001	1 CAGDITTVVATWSYWYFD\
TCL_E31_2	0.001	1 CAGDITTVVATWSYWYFD\
TCL_E31_2	0.001	1 CAGDITTVVATWSYWYFD\
TCL_E31_2	0.001	1 CAGDITTVVATWSYWYFD\
TCL_E31_2	0.001	1 CAGDITTVVATWSYWYFD\
TCL_E31_2	0.001	1 CAGDITTVVATWSYWYFD\
TCL_E31_2	0.001	1 CAGDITTVVATWSYWYFD\
TCL_E31_2	0.001	1 CAGDITTVVATWSYWYFD\
TCL_E31_2	0.001	1 CAGDITTVVATWSYWYFD\
TCL_E31_2	0.001	1 CAGDITTVVATWSYWYFD\
TCL_E31_2	0.001	1 CAGDITTVVATWSYWYFD\
TCL_E31_2	0.001	1 CAGDITTVVATWSYWYFD\
TCL_E31_2	0.001	1 CAGDITTVVATWSYWYFD\
TCL_E31_2	0.001	1 CAGDITTVVATWSYWYFD\
TCL_E31_2	0.001	1 CAGDITTVVATWSYWYFD\
TCL_E31_2	0.001	1 CAGDITTVVATWSYWYFD\
TCL_E31_2	0.001	1 CAGDITTVVATWSYWYFD\
TCL_E31_2	0.001	1 CAGDITTVVATWSYWYFD\
TCL_E31_2	0.001	1 CAGDITTVVATWSYWYFD\
TCL_E31_2	0.001	1 CAGDITTVVATWSYWYFD\
TCL_E31_2	0.001	1 CAGDITTVVATWSYWYFD\
TCL_E31_2	0.001	1 CAGDITTVVATWSYWYFD\
TCL_E31_2	0.001	1 CAGDITTVVATWSYWYFD\
TCL_E31_2	0.001	1 CAGDITTVVATWSYWYFD\
TCL_E31_2	0.001	1 CAGDITTVVATWSYWYFD\
TCL_E31_2	0.001	1 CAGDITTVVATWSYWYFD\
TCL_E31_2	0.001	1 CAGDITTVVATWSYWYFD\
TCL_E31_2	0.001	1 CAGDITTVVATWSYWYFD\
TCL_E31_2	0.001	1 CAGDITTVVATWSYWYFD\
TCL_E31_2	0.001	1 CAGDITTVVATWSYWYFD\

TCL_E31_2	0.001	1	CAGDITTVVATWSYWYFD\
TCL_E31_2	0.001	1	CAGDITTVVATWSYWYFD\
TCL_E31_2	0.001	1	CAGDITTVVATWSYWYFD\
TCL_E31_2	0.001	1	CAGDITTVVATWSYWYFD\
TCL_E31_2	0.001	1	CAGDITTVVATWSYWYFD\
TCL_E31_2	0.001	1	CAGDITTVVATWSYWYFD\
TCL_E31_2	0.001	1	CAGDITTVVATWSYWYFD\
TCL_E31_2	0.001	1	CAGDITTVVATWSYWYFD\
TCL_E31_2	0.001	1	CAGDITTVVATWSYWYFD\
TCL_E31_2	0.001	1	CAGDITTVVATWSYWYFD\
TCL_E31_2	0.001	1	CAGDITTVVATWSYWYFD\
TCL_E31_2	0.001	1	CAGDITTVVATWSYWYFD\
TCL_E31_2	0.001	1	CAGDITTVVATWSYWYFD\
TCL_E31_2	0.001	1	CAGDITTVVATWSYWYFD\
TCL_E31_2	0.001	1	CAGDITTVVATWSYWYFD\
TCL_E31_2	0.001	1	CAGDITTVVATWSYWYFD\
TCL_E31_2	0.001	1	CAGDITTVVATWSYWYFD\
TCL_E31_2	0.001	1	CAGDITTVVATWSYWYFD\
TCL_E31_2	0.001	1	CAGDITTVVATWSYWYFD\
TCL_E31_2	0.001	1	CAGDITTVVATWSYWYFD\
TCL_E31_2	0.001	1	CAGDITTVVATWSYWYFD\
TCL_E31_2	0.001	1	CAGDITTVVATWSYWYFD\
TCL_E31_2	0.001	1	CAGDITTVVATWSYWYFD\
TCL_E31_2	0.001	1	CAGDITTVVATWSYWYFD\
TCL_E31_2	0.001	1	CAGDITTVVATWSYWYFD\
TCL_E31_2	0.001	1	CAGDITTVVATWSYWYFD\
TCL_E31_2	0.001	1	CAGDITTVVATWSYWYFD\
TCL_E31_2	0.001	1	CAGDITTVVATWSYWYFD\
TCL_E31_2	0.001	1	CAGDITTVVATWSYWYFD\
TCL_E31_2	0.001	1	CAGDITTVVATWSYWYFD\
TCL_E31_2	0.001	1	CAGDITTVVATWSYWYFD\
TCL_E31_2	0.001	1	CAGDITTVVATWSYWYFD\
TCL_E31_2	0.001	1	CAGDITTVVATWSYWYFD\
TCL_E31_2	0.001	1	CAGDITTVVATWSYWYFD\
TCL_E31_2	0.001	1	CAGDITTVVATWSYWYFD\
TCL_E31_2	0.001	1	CADITTVVATWSYWYFDV\
TCL_E31_2	0.001	1	CAGDITTVVATGSYWYFDV
TCL_E31_2	0.001	1	CAIPRLRYFDVW
total	100	94588	

rearrangement

CGTCAAAGAACCAGTTCTTCCTCCAATTGAACTCTGTGACCACAGAGGACACAGCCATGTATTACTGTGCAGGAGACATT
GATTCACTATCTTCAGAGACAATGACAAGAGCACCCTGTACCTGCAGATGAGCAATGTGCGATCGGAGGACACAGCCAC
CTGAAAAGAACCAGTTCTTCCTCCAATTGAACTCTGTGACCACAGAGGACACAGCCATGTATTACTGTGCAGGAGACATT
TGACTGTAGACACATCCTCCAGCACAGCCTACATGCAGCTCAGCAGCCTGACATCTGAGGACTCTGCGGTCTATTACTGT
CACTGACTGTAGACACATCCTCCAGCACAGCCTACATGCAGCTCAGCAGCCTGACATCTGAGGACTCTGCGGTCTATTAC
ACGTCAAGAACCAGTTCTTCCTCCAATTGAACTCTGTGACCACAGAGGACACAGCCATGTATTACTGTGCAGGAGACATT
CATTGACTGTAGACAAATCCTCCAGCACAGCCTACATGCAGCTCAGCAGCCTGACATCTGAGGACTCTGCGGTCTATTAC
ACGTCAAAGAACAGTTCTTCCTCCAATTGAACTCTGTGACCACAGAGGACACAGCCATGTATTACTGTGCAGGAGACATT
ACGTCAAAGAACCAGTTCTTCCTCAATTGAACTCTGTGACCACAGAGGACACAGCCATGTATTACTGTGCAGGAGACATT
CCACATTGACTGTAGACACATCCTCCAGCACAGCCTACATGCAGCTCAGCAGCCTGACATCTGAGGACTCTGCGGTCTAT
CGGCAACTCACCAGTTCTTCCTCCAATTGAACTCTGTGACCACAGAGGACACAGCCATGTATTACTGTGCAGGAGACATT
ACGTCAAAGAACCAGTTCTCCTCCAATTGAACTCTGTGACCACAGAGGACACAGCCATGTATTACTGTGCAGGAGACATT
CTGCAACTAACCAGTTCTTCCTCCAATTGAACTCTGTGACCACAGAGGACACAGCCATGTATTACTGTGCAGGAGACATTA
ACGTCAAAGACCAGTTCTTCCTCCAATTGAACTCTGTGACCACAGAGGACACAGCCATGTATTACTGTGCAGGAGACATT
ACGTCAAAGAACCAGTTCTTCCCCAATTGAACTCTGTGACCACAGAGGACACAGCCATGTATTACTGTGCAGGAGACATT
CTGCAAATCACCAGTTCTTCCTCCAATTGAACTCTGTGACCACAGAGGACACAGCCATGTATTACTGTGCAGGAGACATTA
ACGTAAAGAACCAGTTCTTCCTCCAATTGAACTCTGTGACCACAGAGGACACAGCCATGTATTACTGTGCAGGAGACATT
CGTCAACTACACAGTTCTTCCTCCAATTGAACTCTGTGACCACAGAGGACACAGCCATGTATTACTGTGCAGGAGACATTA
ACGTCAAAGAACCAGTTCTTCCTCCATTGAACTCTGTGACCACAGAGGACACAGCCATGTATTACTGTGCAGGAGACATT
GGCGATTCACCATCTCCAGAGACAATGCCAAGAACAACCTGTACCTGCAAATGAGCAGTCTGAGGTCTGAGGACACGGC
CCAAACTGACTGCAGTCACATCCGCCAGCACTGCCTACATGGAGCTCAGCAGCCTGACAAATGAGGACTCTGCGGTCTAT
CCACATTCTCTGTAGACCGGTCCTCCAGCACAGTGTACATGGTGTTGAACAGCCTGACATCTGAGGACCCTGCTGTCTAT
GCATCAGCAAGGACAACTCCAAGAGCCAAGTTTTCTTAAAAATGAACAGTCTGCAAACTGATGACACAGCCATATATTAC
CAACATTGACTGTAGACATATCCTCTAGCACAGCCTATATGGAGCTTAGCAGATTGACATCTGAAGACTCAGAGGTATAT
GAATCTCCATAACTCGAGACACATCCAAGAACCAGTATTACCTGCAGTTGAATTCTGTGACTACTGAGGACACAGCCACA
CGGAAACTAACCAGTTCTTCCTCCAATTGAACTCTGTGACCACAGAGGACACAGCCATGTATTACTGTGCAGGAGACATT
ACGTCAAAGAACCAGTTCTTCCTCCAATTAACTCTGTGACCACAGAGGACACAGCCATGTATTACTGTGCAGGAGACATT
CCGTCAAGAACCAGTTCTTCCTCCAATTGAACTCTGTGACCACAGAGGACACAGCCATGTATTACTGTGCAGGAGACATT
GAATCTCCATCACTCATGACACATCTAAGAACCATTTCTTCCTGAAGTTGAATTCTGTGACTACTGAGGACACAGCCACAT
CGTACAAGACACAGTTCTTCCTCCAATTGAACTCTGTGACCACAGAGGACACAGCCATGTATTACTGTGCAGGAGACATT
ACGTCAAAGAACCAGTTCTTCCTCCAATGAACTCTGTGACCACAGAGGACACAGCCATGTATTACTGTGCAGGAGACATT
ATTCAGAAAGCATGCTCTATCTGCAAATGAACAACTTGAAAACTGAGGACACAGCCATGTATTACTGTGCAGGAGACATT
ACGTCAAAGAACCAGTTCTTCTCCAATTGAACTCTGTGACCACAGAGGACACAGCCATGTATTACTGTGCAGGAGACATT
CGGCATAGTCCCAGTTCTTCCTCCAATTGAACTCTGTGACCACAGAGGACACAGCCATGTATTACTGTGCAGGAGACATT
CGTACAATCACCAGTTCTTCCTCCAATTGAACTCTGTGACCACAGAGGACACAGCCATGTATTACTGTGCAGGAGACATTA
TCTTCACTATCTTCAGAGACAATGACAAGAGCACCCTGTACCTGCAGATGAGCAATGTGCGATCGGAGGACACAGCCAC
AAACGTCAAAGAACCAGTTCCTCCAATTGAACTCTGTGACCACAGAGGACACAGCCATGTATTACTGTGCAGGAGACATT
CGGAAAAGACACTGTTCTTCCTCCAATTGAACTCTGTGACCACAGAGGACACAGCCATGTATTACTGTGCAGGAGACATT
CGTCCAAGACAGAGTTCTTCCTCCAATTGAACTCTGTGACCACAGAGGACACAGCCATGTATTACTGTGCAGGAGACATT
CGTACAATAAGCAGTTCTTCCTCCAATTGAACTCTGTGACCACAGAGGACACAGCCATGTATTACTGTGCAGGAGACATT
AGGCAAACTACCAGTTCTTCCTCCAATTGAACTCTGTGACCACAGAGGACACAGCCATGTATTACTGTGCAGGAGACATT
AGTCATATCACCAGTTCTTCCTCCAATTGAACTCTGTGACCACAGAGGACACAGCCATGTATTACTGTGCAGGAGACATTA
GTTAAATGAACCAGTTCTTCCTCCAATTGAACTCTGTGACCACAGAGGACACAGCCATGTATTACTGTGCAGGAGACATT
ACGGAAAGAACCAGTTCTTCCTCCAATTGAACTCTGTGACCACAGAGGACACAGCCATGTATTACTGTGCAGGAGACATT
CGTCATACACACAGTTCTTCCTCCAATTGAACTCTGTGACCACAGAGGACACAGCCATGTATTACTGTGCAGGAGACATTA
CGTCAATCTAACAGTTCTTCCTCCAATTGAACTCTGTGACCACAGAGGACACAGCCATGTATTACTGTGCAGGAGACATTA
ACGTCAAAGAACCAGTTCTTCCTCCAATTGACTCTGTGACCACAGAGGACACAGCCATGTATTACTGTGCAGGAGACATT
CGGCAAAGACACCTTTCTTCCTCCAATTGAACTCTGTGACCACAGAGGACACAGCCATGTATTACTGTGCAGGAGACATT
CGTCTATGATCGAGTTCTTCCTCCAATTGAACTCTGTGACCACAGAGGACACAGCCATGTATTACTGTGCAGGAGACATTA

CGGCATAGACCGAGTTCTTCCTCCAATTGAACTCTGTGACCACAGAGGACACAGCCATGTATTACTGTGCAGGAGACATTA
CGTCCAATCACGAGTTCTTCCTCCAATTGAACTCTGTGACCACAGAGGACACAGCCATGTATTACTGTGCAGGAGACATTA
CGTAAAATAAAGAGTTCTTCCTCCAATTGAACTCTGTGACCACAGAGGACACAGCCATGTATTACTGTGCAGGAGACATTA
CTTCATAGTAGCAGTTCTTCCTCCAATTGAACTCTGTGACCACAGAGGACACAGCCATGTATTACTGTGCAGGAGACATTA
CTTCAATTATCCAGTTCTTCCTCCAATTGAACTCTGTGACCACAGAGGACACAGCCATGTATTACTGTGCAGGAGACATTA
CGTAATACATCCAGTTCTTCCTCCAATTGAACTCTGTGACCACAGAGGACACAGCCATGTATTACTGTGCAGGAGACATTA
CGTCACACTCCCAGTTCTTCCTCCAATTGAACTCTGTGACCACAGAGGACACAGCCATGTATTACTGTGCAGGAGACATTA
CTTAAACGACCCAGTTCTTCCTCCAATTGAACTCTGTGACCACAGAGGACACAGCCATGTATTACTGTGCAGGAGACATTA
AGTAAACGACCCAGTTCTTCCTCCAATTGAACTCTGTGACCACAGAGGACACAGCCATGTATTACTGTGCAGGAGACATTA
CCTCAACCACCCAGTTCTTCCTCCAATTGAACTCTGTGACCACAGAGGACACAGCCATGTATTACTGTGCAGGAGACATTA
CCTAAAACACCCAGTTCTTCCTCCAATTGAACTCTGTGACCACAGAGGACACAGCCATGTATTACTGTGCAGGAGACATTA
CTTCAATTTACCAGTTCTTCCTCCAATTGAACTCTGTGACCACAGAGGACACAGCCATGTATTACTGTGCAGGAGACATTA
AGTCATATTACCAGTTCTTCCTCCAATTGAACTCTGTGACCACAGAGGACACAGCCATGTATTACTGTGCAGGAGACATTA
CGTCTATCTACCAGTTCTTCCTCCAATTGAACTCTGTGACCACAGAGGACACAGCCATGTATTACTGTGCAGGAGACATTA
CTTCAATCTACCAGTTCTTCCTCCAATTGAACTCTGTGACCACAGAGGACACAGCCATGTATTACTGTGCAGGAGACATTA
GGTCAATCTACCAGTTCTTCCTCCAATTGAACTCTGTGACCACAGAGGACACAGCCATGTATTACTGTGCAGGAGACATTA
CGTCTCACTACCAGTTCTTCCTCCAATTGAACTCTGTGACCACAGAGGACACAGCCATGTATTACTGTGCAGGAGACATTA
ACGGCAAAGACCAGTTCTTCCTCCAATTGAACTCTGTGACCACAGAGGACACAGCCATGTATTACTGTGCAGGAGACATTA
ACGTCAACCACCAGTTCTTCCTCCAATTGAACTCTGTGACCACAGAGGACACAGCCATGTATTACTGTGCAGGAGACATTA
AGGAAATTAACCAGTTCTTCCTCCAATTGAACTCTGTGACCACAGAGGACACAGCCATGTATTACTGTGCAGGAGACATTA
AGTAAATCAACCAGTTCTTCCTCCAATTGAACTCTGTGACCACAGAGGACACAGCCATGTATTACTGTGCAGGAGACATTA
CGTCTAATATACAGTTCTTCCTCCAATTGAACTCTGTGACCACAGAGGACACAGCCATGTATTACTGTGCAGGAGACATTA
CGGCATAGATACAGTTCTTCCTCCAATTGAACTCTGTGACCACAGAGGACACAGCCATGTATTACTGTGCAGGAGACATTA
CGGCCAATAAACAGTTCTTCCTCCAATTGAACTCTGTGACCACAGAGGACACAGCCATGTATTACTGTGCAGGAGACATTA
CGTAAAACTACAAGTTCTTCCTCCAATTGAACTCTGTGACCACAGAGGACACAGCCATGTATTACTGTGCAGGAGACATTA
CGTCAACGACCCCTGTCTTCCTCCAATTGAACTCTGTGACCACAGAGGACACAGCCATGTATTACTGTGCAGGAGACATTA
CGTCACAGCACCCTGTCTTCCTCCAATTGAACTCTGTGACCACAGAGGACACAGCCATGTATTACTGTGCAGGAGACATTA
CGGCACAGAACCCTGTCTTCCTCCAATTGAACTCTGTGACCACAGAGGACACAGCCATGTATTACTGTGCAGGAGACATTA
ACGTCAAAGAACCAGTCTTCCTCCAATTGAACTCTGTGACCACAGAGGACACAGCCATGTATTACTGTGCAGGAGACATTA
ACGTCAAAGAACCAGTGCTCCTCCAATTGAACTCTGTGACCACAGAGGACACAGCCATGTATTACTGTGCAGGAGACATT
ACGTCAAAGAACCAGTGCTTCCCCAATTGAACTCTGTGACCACAGAGGACACAGCCATGTATTACTGTGCAGGAGACATT
AAACGTCAAAGAACCAGTTCTTCCTCCAATTGAACTCTGTGACCACAGAGGACACAGCCATGTATTACTGTGCAGGAGAC
CCTAACAGAACCAGTTCTTCCTCCAATTGAACTCTGTGACCACAGAGGACACAGCCATGTATTACTGTGCAGGAGACATTA
CGTTATAGAACCAGTTCTTCCTCCAATTGAACTCTGTGACCACAGAGGACACAGCCATGTATTACTGTGCAGGAGACATTA
CGTCAATTATCCTTTTCTTCCTCCAATTGAACTCTGTGACCACAGAGGACACAGCCATGTATTACTGTGCAGGAGACATTA
CGGCAAATAACACTTTCTTCCTCCAATTGAACTCTGTGACCACAGAGGACACAGCCATGTATTACTGTGCAGGAGACATTA
CGGCACAGAACACTTTCTTCCTCCAATTGAACTCTGTGACCACAGAGGACACAGCCATGTATTACTGTGCAGGAGACATTA
CGTCAACCAACACTTTCTTCCTCCAATTGAACTCTGTGACCACAGAGGACACAGCCATGTATTACTGTGCAGGAGACATTA
CTTCTAAGTACGATTTCTTCCTCCAATTGAACTCTGTGACCACAGAGGACACAGCCATGTATTACTGTGCAGGAGACATTA
CGTCTAATACACATTTCTTCCTCCAATTGAACTCTGTGACCACAGAGGACACAGCCATGTATTACTGTGCAGGAGACATTA
CGTAAAATTAACATTTCTTCCTCCAATTGAACTCTGTGACCACAGAGGACACAGCCATGTATTACTGTGCAGGAGACATTA
CGTCAACTACACTGTTCTTCCTCCAATTGAACTCTGTGACCACAGAGGACACAGCCATGTATTACTGTGCAGGAGACATTA
CGGAAAAGACCACGTTCTTCCTCCAATTGAACTCTGTGACCACAGAGGACACAGCCATGTATTACTGTGCAGGAGACATTA
CGTCATAGTTCGAGTTCTTCCTCCAATTGAACTCTGTGACCACAGAGGACACAGCCATGTATTACTGTGCAGGAGACATTA
CGTGAAATATCGAGTTCTTCCTCCAATTGAACTCTGTGACCACAGAGGACACAGCCATGTATTACTGTGCAGGAGACATTA
CGTGAATGATCGAGTTCTTCCTCCAATTGAACTCTGTGACCACAGAGGACACAGCCATGTATTACTGTGCAGGAGACATTA
AGTGAATGTACGAGTTCTTCCTCCAATTGAACTCTGTGACCACAGAGGACACAGCCATGTATTACTGTGCAGGAGACATTA
CGTGATAGTACGAGTTCTTCCTCCAATTGAACTCTGTGACCACAGAGGACACAGCCATGTATTACTGTGCAGGAGACATTA
CGTCATACTACGAGTTCTTCCTCCAATTGAACTCTGTGACCACAGAGGACACAGCCATGTATTACTGTGCAGGAGACATTA
CGTCAATTCACGAGTTCTTCCTCCAATTGAACTCTGTGACCACAGAGGACACAGCCATGTATTACTGTGCAGGAGACATTA

CTGCAACGCACGAGTTCTTCCTCCAATTGAACTCTGTGACCACAGAGGACACAGCCATGTATTACTGTGCAGGAGACATTA
CTGAAAAGACAGAGTTCTTCCTCCAATTGAACTCTGTGACCACAGAGGACACAGCCATGTATTACTGTGCAGGAGACATTA
CGGCAAAGCAAGAGTTCTTCCTCCAATTGAACTCTGTGACCACAGAGGACACAGCCATGTATTACTGTGCAGGAGACATTA
CGTCTATGAAAGAGTTCTTCCTCCAATTGAACTCTGTGACCACAGAGGACACAGCCATGTATTACTGTGCAGGAGACATTA
AGTCTATGAAAGAGTTCTTCCTCCAATTGAACTCTGTGACCACAGAGGACACAGCCATGTATTACTGTGCAGGAGACATTA
CGTCTATGATGCAGTTCTTCCTCCAATTGAACTCTGTGACCACAGAGGACACAGCCATGTATTACTGTGCAGGAGACATTA
CTTCATAGACGCAGTTCTTCCTCCAATTGAACTCTGTGACCACAGAGGACACAGCCATGTATTACTGTGCAGGAGACATTA
CGGCTAAGTAGCAGTTCTTCCTCCAATTGAACTCTGTGACCACAGAGGACACAGCCATGTATTACTGTGCAGGAGACATTA
CGTGAAACTAGCAGTTCTTCCTCCAATTGAACTCTGTGACCACAGAGGACACAGCCATGTATTACTGTGCAGGAGACATTA
CTTCAAACTAGCAGTTCTTCCTCCAATTGAACTCTGTGACCACAGAGGACACAGCCATGTATTACTGTGCAGGAGACATTA
CCTCAAATCTCCAGTTCTTCCTCCAATTGAACTCTGTGACCACAGAGGACACAGCCATGTATTACTGTGCAGGAGACATTA
CTTATATGATCCAGTTCTTCCTCCAATTGAACTCTGTGACCACAGAGGACACAGCCATGTATTACTGTGCAGGAGACATTA
AGTCATCGATCCAGTTCTTCCTCCAATTGAACTCTGTGACCACAGAGGACACAGCCATGTATTACTGTGCAGGAGACATTA
AGTCTACGATCCAGTTCTTCCTCCAATTGAACTCTGTGACCACAGAGGACACAGCCATGTATTACTGTGCAGGAGACATTA
CGTGAATCATCCAGTTCTTCCTCCAATTGAACTCTGTGACCACAGAGGACACAGCCATGTATTACTGTGCAGGAGACATTA
GGTCATACATCCAGTTCTTCCTCCAATTGAACTCTGTGACCACAGAGGACACAGCCATGTATTACTGTGCAGGAGACATTA
AGTCATACATCCAGTTCTTCCTCCAATTGAACTCTGTGACCACAGAGGACACAGCCATGTATTACTGTGCAGGAGACATTA
AGTCAATGTCCCAGTTCTTCCTCCAATTGAACTCTGTGACCACAGAGGACACAGCCATGTATTACTGTGCAGGAGACATTA
AGGCAAATCCCCAGTTCTTCCTCCAATTGAACTCTGTGACCACAGAGGACACAGCCATGTATTACTGTGCAGGAGACATTA
CGACAAATCCCCAGTTCTTCCTCCAATTGAACTCTGTGACCACAGAGGACACAGCCATGTATTACTGTGCAGGAGACATTA
CGGCACCGCCCCAGTTCTTCCTCCAATTGAACTCTGTGACCACAGAGGACACAGCCATGTATTACTGTGCAGGAGACATTA
CGGCTACTACCCAGTTCTTCCTCCAATTGAACTCTGTGACCACAGAGGACACAGCCATGTATTACTGTGCAGGAGACATTA
CTTCACATACCCAGTTCTTCCTCCAATTGAACTCTGTGACCACAGAGGACACAGCCATGTATTACTGTGCAGGAGACATTA
CGGCTATGACCCAGTTCTTCCTCCAATTGAACTCTGTGACCACAGAGGACACAGCCATGTATTACTGTGCAGGAGACATTA
CGAGAAATTACCAGTTCTTCCTCCAATTGAACTCTGTGACCACAGAGGACACAGCCATGTATTACTGTGCAGGAGACATTA
CGTATATGTACCAGTTCTTCCTCCAATTGAACTCTGTGACCACAGAGGACACAGCCATGTATTACTGTGCAGGAGACATTA
AGGCATAGTACCAGTTCTTCCTCCAATTGAACTCTGTGACCACAGAGGACACAGCCATGTATTACTGTGCAGGAGACATTA
CCTCACACTACCAGTTCTTCCTCCAATTGAACTCTGTGACCACAGAGGACACAGCCATGTATTACTGTGCAGGAGACATTA
CTTGAAACTACCAGTTCTTCCTCCAATTGAACTCTGTGACCACAGAGGACACAGCCATGTATTACTGTGCAGGAGACATTA
CCTCCAATCACCAGTTCTTCCTCCAATTGAACTCTGTGACCACAGAGGACACAGCCATGTATTACTGTGCAGGAGACATTA
CTGAAAATCACCAGTTCTTCCTCCAATTGAACTCTGTGACCACAGAGGACACAGCCATGTATTACTGTGCAGGAGACATTA
AGTCTATGCACCAGTTCTTCCTCCAATTGAACTCTGTGACCACAGAGGACACAGCCATGTATTACTGTGCAGGAGACATTA
CGGACACGCACCAGTTCTTCCTCCAATTGAACTCTGTGACCACAGAGGACACAGCCATGTATTACTGTGCAGGAGACATTA
CTACAACGCACCAGTTCTTCCTCCAATTGAACTCTGTGACCACAGAGGACACAGCCATGTATTACTGTGCAGGAGACATTA
AGTGATAGCACCAGTTCTTCCTCCAATTGAACTCTGTGACCACAGAGGACACAGCCATGTATTACTGTGCAGGAGACATTA
CGGACCAGCACCAGTTCTTCCTCCAATTGAACTCTGTGACCACAGAGGACACAGCCATGTATTACTGTGCAGGAGACATTA
CTGCTACTAACCAGTTCTTCCTCCAATTGAACTCTGTGACCACAGAGGACACAGCCATGTATTACTGTGCAGGAGACATTA
CGAACACTAACCAGTTCTTCCTCCAATTGAACTCTGTGACCACAGAGGACACAGCCATGTATTACTGTGCAGGAGACATTA
CGAGAACTAACCAGTTCTTCCTCCAATTGAACTCTGTGACCACAGAGGACACAGCCATGTATTACTGTGCAGGAGACATTA
CTGAAACTAACCAGTTCTTCCTCCAATTGAACTCTGTGACCACAGAGGACACAGCCATGTATTACTGTGCAGGAGACATTA
GGTGATATAACCAGTTCTTCCTCCAATTGAACTCTGTGACCACAGAGGACACAGCCATGTATTACTGTGCAGGAGACATTA
CTTGCAATAACCAGTTCTTCCTCCAATTGAACTCTGTGACCACAGAGGACACAGCCATGTATTACTGTGCAGGAGACATTA
AGTATATGAACCAGTTCTTCCTCCAATTGAACTCTGTGACCACAGAGGACACAGCCATGTATTACTGTGCAGGAGACATTA
ACTGAATGAACCAGTTCTTCCTCCAATTGAACTCTGTGACCACAGAGGACACAGCCATGTATTACTGTGCAGGAGACATTA
AACGTCAGAACCAGTTCTTCCTCCAATTGAACTCTGTGACCACAGAGGACACAGCCATGTATTACTGTGCAGGAGACATTA
CGGCCATCAACCAGTTCTTCCTCCAATTGAACTCTGTGACCACAGAGGACACAGCCATGTATTACTGTGCAGGAGACATTA
GCTCAATCAACCAGTTCTTCCTCCAATTGAACTCTGTGACCACAGAGGACACAGCCATGTATTACTGTGCAGGAGACATTA
AGGCCAACAACCAGTTCTTCCTCCAATTGAACTCTGTGACCACAGAGGACACAGCCATGTATTACTGTGCAGGAGACATTA
CGTCAAATCTACAGTTCTTCCTCCAATTGAACTCTGTGACCACAGAGGACACAGCCATGTATTACTGTGCAGGAGACATTA
CGTCATAGCTACAGTTCTTCCTCCAATTGAACTCTGTGACCACAGAGGACACAGCCATGTATTACTGTGCAGGAGACATTA

CGTCTACGATACAGTTCTTCCTCCAATTGAACTCTGTGACCACAGAGGACACAGCCATGTATTACTGTGCAGGAGACATTA
CGGCTAAGATACAGTTCTTCCTCCAATTGAACTCTGTGACCACAGAGGACACAGCCATGTATTACTGTGCAGGAGACATTA
CGGAACAGACACAGTTCTTCCTCCAATTGAACTCTGTGACCACAGAGGACACAGCCATGTATTACTGTGCAGGAGACATTA
CGGACAAGACACAGTTCTTCCTCCAATTGAACTCTGTGACCACAGAGGACACAGCCATGTATTACTGTGCAGGAGACATTA
CCTCAAATTAACAGTTCTTCCTCCAATTGAACTCTGTGACCACAGAGGACACAGCCATGTATTACTGTGCAGGAGACATTA
CGTCTACGTAACAGTTCTTCCTCCAATTGAACTCTGTGACCACAGAGGACACAGCCATGTATTACTGTGCAGGAGACATTA
CCGCAACTAAACAGTTCTTCCTCCAATTGAACTCTGTGACCACAGAGGACACAGCCATGTATTACTGTGCAGGAGACATTA
CGGCAAAGACGAAGTTCTTCCTCCAATTGAACTCTGTGACCACAGAGGACACAGCCATGTATTACTGTGCAGGAGACATTA
GGTATAAGTAGAAGTTCTTCCTCCAATTGAACTCTGTGACCACAGAGGACACAGCCATGTATTACTGTGCAGGAGACATTA
CGTCTAATATCAAGTTCTTCCTCCAATTGAACTCTGTGACCACAGAGGACACAGCCATGTATTACTGTGCAGGAGACATTA
CGTGAATGACCAAGTTCTTCCTCCAATTGAACTCTGTGACCACAGAGGACACAGCCATGTATTACTGTGCAGGAGACATTA
CGTAATAGACCAAGTTCTTCCTCCAATTGAACTCTGTGACCACAGAGGACACAGCCATGTATTACTGTGCAGGAGACATTA
CGGCTAAGACCCTCTTCTTCCTCCAATTGAACTCTGTGACCACAGAGGACACAGCCATGTATTACTGTGCAGGAGACATTA
CGTCACAGATCCCTGTCTTCCTCCAATTGAACTCTGTGACCACAGAGGACACAGCCATGTATTACTGTGCAGGAGACATTA
CGGCAAAGACCCCTGTCTTCCTCCAATTGAACTCTGTGACCACAGAGGACACAGCCATGTATTACTGTGCAGGAGACATTA
CGGCAAAGCACCCTGTCTTCCTCCAATTGAACTCTGTGACCACAGAGGACACAGCCATGTATTACTGTGCAGGAGACATTA
CGTCAACTGACCATGTCTTCCTCCAATTGAACTCTGTGACCACAGAGGACACAGCCATGTATTACTGTGCAGGAGACATTA
CGACAACGACCCTGGTCTTCCTCCAATTGAACTCTGTGACCACAGAGGACACAGCCATGTATTACTGTGCAGGAGACATTA
CGTCCACGACCCCGGTCTTCCTCCAATTGAACTCTGTGACCACAGAGGACACAGCCATGTATTACTGTGCAGGAGACATTA
CGGCAACGACCCCGGTCTTCCTCCAATTGAACTCTGTGACCACAGAGGACACAGCCATGTATTACTGTGCAGGAGACATTA
CGGCAAACACCCCGGTCTTCCTCCAATTGAACTCTGTGACCACAGAGGACACAGCCATGTATTACTGTGCAGGAGACATTA
CGTCACAGAAGACGGTCTTCCTCCAATTGAACTCTGTGACCACAGAGGACACAGCCATGTATTACTGTGCAGGAGACATTA
CGGCAACGACCCACGTCTTCCTCCAATTGAACTCTGTGACCACAGAGGACACAGCCATGTATTACTGTGCAGGAGACATTA
CGTCAACGACCCCCAGCTTCCTCCAATTGAACTCTGTGACCACAGAGGACACAGCCATGTATTACTGTGCAGGAGACATTA
ACGTCAACGAACCAGTTCTTCCCCAATTGAACTCTGTGACCACAGAGGACACAGCCATGTATTACTGTGCAGGAGACATTA
GTCAAAGAACCAGTTCTTCCTCCAAATTGAACTCTGTGACCACAGAGGACACAGCCATGTATTACTGTGCAGGAGACATTA
TTAATGTAGACACATCCTCCAGCACAGCCTACATGCAGCTCAGCAGCCTGACATCTGAGGACTCTGCGGTCTATTACTGTG
TCCAGGGCAAGGCCACTATGACTGCAGACACATCCTCCAACACAGCCTACCTGCAGCTCAGCAGCCTGACATCTGAGGAC
GCAAGGCCACATTCACTGCAGATACATCCTCCAACACAGCCTACATGCAACTCAGCAGCCTGACAACTGAGGACTCTGCCA
GCAAGGCCACATTGACTGTAGACAATTCCTCCAGCACAGCCTACATGGAGCTCAGCAGCCTGACATCTGAGGACTCTGCA
CCACACTGACGGCAGACACATCCTCCAGCACAGCCTACATGCAGCTCAGCAGCCTGACATCTGAGGACTCTGCGGTCCAT
CGGAAAATCACCAGTTCTTCCTCCAATTGAACTCTGTGACCACAGAGGACACAGCCATGTATTACTGTGCAGGAGACATTA
CGGCAACGACACAGTTCTTCCTCCAATTGAACTCTGTGACCACAGAGGACACAGCCATGTATTACTGTGCAGGAGACATTA
CGTAAACTACCCAGTTCTTCCTCCAATTGAACTCTGTGACCACAGAGGACACAGCCATGTATTACTGTGCAGGAGACATTA
CGGAAAACTACCAGTTCTTCCTCCAATTGAACTCTGTGACCACAGAGGACACAGCCATGTATTACTGTGCAGGAGACATTA
CGGCAACCCACCAGTTCTTCCTCCAATTGAACTCTGTGACCACAGAGGACACAGCCATGTATTACTGTGCAGGAGACATTA
CGGAAATCAACCAGTTCTTCCTCCAATTGAACTCTGTGACCACAGAGGACACAGCCATGTATTACTGTGCAGGAGACATTA
GATGAACTATCTTCAGAGACAATGACAAGAGCACCCTGTACCTGCAGATGAGCAATGTGCGATCGGAGGACACAGCCAC
ACGTCAAAGAACCAGTTTTCCTCCAATTGAACTCTGTGACCACAGAGGACACAGCCATGTATTACTGTGCAGGAGACATTA
CGTACACGACCCAGTTCTTCCTCCAATTGAACTCTGTGACCACAGAGGACACAGCCATGTATTACTGTGCAGGAGACATTA
CGTAAATCTACCAGTTCTTCCTCCAATTGAACTCTGTGACCACAGAGGACACAGCCATGTATTACTGTGCAGGAGACATTA
CTTCAAATATACAGTTCTTCCTCCAATTGAACTCTGTGACCACAGAGGACACAGCCATGTATTACTGTGCAGGAGACATTA
CGTCACACACACAGTTCTTCCTCCAATTGAACTCTGTGACCACAGAGGACACAGCCATGTATTACTGTGCAGGAGACATTA
ACGTCAAAGAACCGTTCTTCCTCCAATTGAACTCTGTGACCACAGAGGACACAGCCATGTATTACTGTGCAGGAGACATTA
CGGCACACTACCAGTTCTTCCTCCAATTGAACTCTGTGACCACAGAGGACACAGCCATGTATTACTGTGCAGGAGACATTA
AGTCTAATCACCAGTTCTTCCTCCAATTGAACTCTGTGACCACAGAGGACACAGCCATGTATTACTGTGCAGGAGACATTA
CCGCAAACCACCAGTTCTTCCTCCAATTGAACTCTGTGACCACAGAGGACACAGCCATGTATTACTGTGCAGGAGACATTA
CTGACAATAACCAGTTCTTCCTCCAATTGAACTCTGTGACCACAGAGGACACAGCCATGTATTACTGTGCAGGAGACATTA
GCTAATAGAACCAGTTCTTCCTCCAATTGAACTCTGTGACCACAGAGGACACAGCCATGTATTACTGTGCAGGAGACATTA
CGTCACATCACAAGTTCTTCCTCCAATTGAACTCTGTGACCACAGAGGACACAGCCATGTATTACTGTGCAGGAGACATTA

CGGCACAGACACCGTTCTTCCTCCAATTGAACTCTGTGACCACAGAGGACACAGCCATGTATTACTGTGCAGGAGACATTA
CGTCATATATCGAGTTCTTCCTCCAATTGAACTCTGTGACCACAGAGGACACAGCCATGTATTACTGTGCAGGAGACATTA
CGGCAATGACCGAGTTCTTCCTCCAATTGAACTCTGTGACCACAGAGGACACAGCCATGTATTACTGTGCAGGAGACATTA
CGTAAATGAAAGAGTTCTTCCTCCAATTGAACTCTGTGACCACAGAGGACACAGCCATGTATTACTGTGCAGGAGACATTA
CGTCAATCTAGCAGTTCTTCCTCCAATTGAACTCTGTGACCACAGAGGACACAGCCATGTATTACTGTGCAGGAGACATTA
AGTAAAACATCCAGTTCTTCCTCCAATTGAACTCTGTGACCACAGAGGACACAGCCATGTATTACTGTGCAGGAGACATTA
CTTCAATCACCCAGTTCTTCCTCCAATTGAACTCTGTGACCACAGAGGACACAGCCATGTATTACTGTGCAGGAGACATTA
CTTAAATGTACCAGTTCTTCCTCCAATTGAACTCTGTGACCACAGAGGACACAGCCATGTATTACTGTGCAGGAGACATTA
CTTAAAACTACCAGTTCTTCCTCCAATTGAACTCTGTGACCACAGAGGACACAGCCATGTATTACTGTGCAGGAGACATTA
AGTAAAACTACCAGTTCTTCCTCCAATTGAACTCTGTGACCACAGAGGACACAGCCATGTATTACTGTGCAGGAGACATTA
CTGCCACGCACCAGTTCTTCCTCCAATTGAACTCTGTGACCACAGAGGACACAGCCATGTATTACTGTGCAGGAGACATTA
CTGACACGAACCAGTTCTTCCTCCAATTGAACTCTGTGACCACAGAGGACACAGCCATGTATTACTGTGCAGGAGACATTA
ACGCACAGAACCAGTTCTTCCTCCAATTGAACTCTGTGACCACAGAGGACACAGCCATGTATTACTGTGCAGGAGACATTA
CGTACATCAACCAGTTCTTCCTCCAATTGAACTCTGTGACCACAGAGGACACAGCCATGTATTACTGTGCAGGAGACATTA
CGTCTATGATACAGTTCTTCCTCCAATTGAACTCTGTGACCACAGAGGACACAGCCATGTATTACTGTGCAGGAGACATTA
CGTATAAGATACAGTTCTTCCTCCAATTGAACTCTGTGACCACAGAGGACACAGCCATGTATTACTGTGCAGGAGACATTA
CGTCAAACTCACAGTTCTTCCTCCAATTGAACTCTGTGACCACAGAGGACACAGCCATGTATTACTGTGCAGGAGACATTA
CTTCTAAGTAACAGTTCTTCCTCCAATTGAACTCTGTGACCACAGAGGACACAGCCATGTATTACTGTGCAGGAGACATTA
ACGTAAAAGAACAGTTCTTCCTCCAATTGAACTCTGTGACCACAGAGGACACAGCCATGTATTACTGTGCAGGAGACATTA
ATTCACAAAGCATGCTCTATCTGCAAATGAACAACTTGAAAACTGAGGACACAGCCATGTATTACTGTGCAGGAGACATTA
CTTCACAGTACCTTTTCTTCCTCCAATTGAACTCTGTGACCACAGAGGACACAGCCATGTATTACTGTGCAGGAGACATTA
CGGCAACGCACCCTTTCTTCCTCCAATTGAACTCTGTGACCACAGAGGACACAGCCATGTATTACTGTGCAGGAGACATTA
CGGCAATTAAGCATTTCTTCCTCCAATTGAACTCTGTGACCACAGAGGACACAGCCATGTATTACTGTGCAGGAGACATTA
CGTCTATGTAACTGTTCTTCCTCCAATTGAACTCTGTGACCACAGAGGACACAGCCATGTATTACTGTGCAGGAGACATTA
CGTCTTAGAAGATGTTCTTCCTCCAATTGAACTCTGTGACCACAGAGGACACAGCCATGTATTACTGTGCAGGAGACATTA
CGGCTAAGATCATGTTCTTCCTCCAATTGAACTCTGTGACCACAGAGGACACAGCCATGTATTACTGTGCAGGAGACATTA
CGGCACCGACCCCGTTCTTCCTCCAATTGAACTCTGTGACCACAGAGGACACAGCCATGTATTACTGTGCAGGAGACATTA
AGTCCACGACCCCGTTCTTCCTCCAATTGAACTCTGTGACCACAGAGGACACAGCCATGTATTACTGTGCAGGAGACATTA
CGGCCACGACCCCGTTCTTCCTCCAATTGAACTCTGTGACCACAGAGGACACAGCCATGTATTACTGTGCAGGAGACATTA
CGGAAAACACCCCGTTCTTCCTCCAATTGAACTCTGTGACCACAGAGGACACAGCCATGTATTACTGTGCAGGAGACATTA
CGTACAAGGCACCGTTCTTCCTCCAATTGAACTCTGTGACCACAGAGGACACAGCCATGTATTACTGTGCAGGAGACATTA
CTTAAAAGACCACGTTCTTCCTCCAATTGAACTCTGTGACCACAGAGGACACAGCCATGTATTACTGTGCAGGAGACATTA
CGGCACAGCACACGTTCTTCCTCCAATTGAACTCTGTGACCACAGAGGACACAGCCATGTATTACTGTGCAGGAGACATTA
CGTTAAATATCGAGTTCTTCCTCCAATTGAACTCTGTGACCACAGAGGACACAGCCATGTATTACTGTGCAGGAGACATTA
CTGCAAATATCGAGTTCTTCCTCCAATTGAACTCTGTGACCACAGAGGACACAGCCATGTATTACTGTGCAGGAGACATTA
CGGAAATGATCGAGTTCTTCCTCCAATTGAACTCTGTGACCACAGAGGACACAGCCATGTATTACTGTGCAGGAGACATTA
CGTCATTGTACGAGTTCTTCCTCCAATTGAACTCTGTGACCACAGAGGACACAGCCATGTATTACTGTGCAGGAGACATTA
CGTCTATGTACGAGTTCTTCCTCCAATTGAACTCTGTGACCACAGAGGACACAGCCATGTATTACTGTGCAGGAGACATTA
CGGCAACTCACGAGTTCTTCCTCCAATTGAACTCTGTGACCACAGAGGACACAGCCATGTATTACTGTGCAGGAGACATTA
CTGCAAAGACAGAGTTCTTCCTCCAATTGAACTCTGTGACCACAGAGGACACAGCCATGTATTACTGTGCAGGAGACATTA
CGTCATAGTAAGAGTTCTTCCTCCAATTGAACTCTGTGACCACAGAGGACACAGCCATGTATTACTGTGCAGGAGACATTA
CGTTAAAGTAAGAGTTCTTCCTCCAATTGAACTCTGTGACCACAGAGGACACAGCCATGTATTACTGTGCAGGAGACATTA
CGTCTAATAAAGAGTTCTTCCTCCAATTGAACTCTGTGACCACAGAGGACACAGCCATGTATTACTGTGCAGGAGACATTA
CGGCTAAGACGCAGTTCTTCCTCCAATTGAACTCTGTGACCACAGAGGACACAGCCATGTATTACTGTGCAGGAGACATTA
CTTCAATGTAGCAGTTCTTCCTCCAATTGAACTCTGTGACCACAGAGGACACAGCCATGTATTACTGTGCAGGAGACATTA
CGTCATACTAGCAGTTCTTCCTCCAATTGAACTCTGTGACCACAGAGGACACAGCCATGTATTACTGTGCAGGAGACATTA
CGTAAAACTAGCAGTTCTTCCTCCAATTGAACTCTGTGACCACAGAGGACACAGCCATGTATTACTGTGCAGGAGACATTA
CGTCCACGCAGCAGTTCTTCCTCCAATTGAACTCTGTGACCACAGAGGACACAGCCATGTATTACTGTGCAGGAGACATTA
CGGCATAGCAGCAGTTCTTCCTCCAATTGAACTCTGTGACCACAGAGGACACAGCCATGTATTACTGTGCAGGAGACATTA
CTTCATATAAGCAGTTCTTCCTCCAATTGAACTCTGTGACCACAGAGGACACAGCCATGTATTACTGTGCAGGAGACATTA

CGTCATACTTCCAGTTCTTCCTCCAATTGAACTCTGTGACCACAGAGGACACAGCCATGTATTACTGTGCAGGAGACATTA
CGTAAACTCTCCAGTTCTTCCTCCAATTGAACTCTGTGACCACAGAGGACACAGCCATGTATTACTGTGCAGGAGACATTA
CGTTCAATATCCAGTTCTTCCTCCAATTGAACTCTGTGACCACAGAGGACACAGCCATGTATTACTGTGCAGGAGACATTA
GGTCACTGATCCAGTTCTTCCTCCAATTGAACTCTGTGACCACAGAGGACACAGCCATGTATTACTGTGCAGGAGACATTA
CGGCACTGATCCAGTTCTTCCTCCAATTGAACTCTGTGACCACAGAGGACACAGCCATGTATTACTGTGCAGGAGACATTA
CGGCTATGATCCAGTTCTTCCTCCAATTGAACTCTGTGACCACAGAGGACACAGCCATGTATTACTGTGCAGGAGACATTA
CGTCTATCATCCAGTTCTTCCTCCAATTGAACTCTGTGACCACAGAGGACACAGCCATGTATTACTGTGCAGGAGACATTA
CGGCAATGTCCCAGTTCTTCCTCCAATTGAACTCTGTGACCACAGAGGACACAGCCATGTATTACTGTGCAGGAGACATTA
CGTCTAATCCCCAGTTCTTCCTCCAATTGAACTCTGTGACCACAGAGGACACAGCCATGTATTACTGTGCAGGAGACATTA
CGTCACTTACCCAGTTCTTCCTCCAATTGAACTCTGTGACCACAGAGGACACAGCCATGTATTACTGTGCAGGAGACATTA
CTGCAACTACCCAGTTCTTCCTCCAATTGAACTCTGTGACCACAGAGGACACAGCCATGTATTACTGTGCAGGAGACATTA
CGTATACGACCCAGTTCTTCCTCCAATTGAACTCTGTGACCACAGAGGACACAGCCATGTATTACTGTGCAGGAGACATTA
ACGTCACGACCCAGTTCTTCCTCCAATTGAACTCTGTGACCACAGAGGACACAGCCATGTATTACTGTGCAGGAGACATTA
CGGACACGACCCAGTTCTTCCTCCAATTGAACTCTGTGACCACAGAGGACACAGCCATGTATTACTGTGCAGGAGACATTA
CGGAACACACCCAGTTCTTCCTCCAATTGAACTCTGTGACCACAGAGGACACAGCCATGTATTACTGTGCAGGAGACATTA
CTGCAACTTACCAGTTCTTCCTCCAATTGAACTCTGTGACCACAGAGGACACAGCCATGTATTACTGTGCAGGAGACATTA
CTTCACATTACCAGTTCTTCCTCCAATTGAACTCTGTGACCACAGAGGACACAGCCATGTATTACTGTGCAGGAGACATTA
CTTCTATGTACCAGTTCTTCCTCCAATTGAACTCTGTGACCACAGAGGACACAGCCATGTATTACTGTGCAGGAGACATTA
GGTAATAGTACCAGTTCTTCCTCCAATTGAACTCTGTGACCACAGAGGACACAGCCATGTATTACTGTGCAGGAGACATTA
CTGCAATCTACCAGTTCTTCCTCCAATTGAACTCTGTGACCACAGAGGACACAGCCATGTATTACTGTGCAGGAGACATTA
CTTCAACCTACCAGTTCTTCCTCCAATTGAACTCTGTGACCACAGAGGACACAGCCATGTATTACTGTGCAGGAGACATTA
CGACATACTACCAGTTCTTCCTCCAATTGAACTCTGTGACCACAGAGGACACAGCCATGTATTACTGTGCAGGAGACATTA
CGTACAACTACCAGTTCTTCCTCCAATTGAACTCTGTGACCACAGAGGACACAGCCATGTATTACTGTGCAGGAGACATTA
AGTCTACTCACCAGTTCTTCCTCCAATTGAACTCTGTGACCACAGAGGACACAGCCATGTATTACTGTGCAGGAGACATTA
AGTAAACTCACCAGTTCTTCCTCCAATTGAACTCTGTGACCACAGAGGACACAGCCATGTATTACTGTGCAGGAGACATTA
CTTAAATGCACCAGTTCTTCCTCCAATTGAACTCTGTGACCACAGAGGACACAGCCATGTATTACTGTGCAGGAGACATTA
CTTCCACGCACCAGTTCTTCCTCCAATTGAACTCTGTGACCACAGAGGACACAGCCATGTATTACTGTGCAGGAGACATTA
CCGCAACCCACCAGTTCTTCCTCCAATTGAACTCTGTGACCACAGAGGACACAGCCATGTATTACTGTGCAGGAGACATTA
CTACAAACCACCAGTTCTTCCTCCAATTGAACTCTGTGACCACAGAGGACACAGCCATGTATTACTGTGCAGGAGACATTA
CCTAAAACCACCAGTTCTTCCTCCAATTGAACTCTGTGACCACAGAGGACACAGCCATGTATTACTGTGCAGGAGACATTA
GGTAAATTAACCAGTTCTTCCTCCAATTGAACTCTGTGACCACAGAGGACACAGCCATGTATTACTGTGCAGGAGACATTA
CGTCTCCTAACCAGTTCTTCCTCCAATTGAACTCTGTGACCACAGAGGACACAGCCATGTATTACTGTGCAGGAGACATTA
ATTCATATAACCAGTTCTTCCTCCAATTGAACTCTGTGACCACAGAGGACACAGCCATGTATTACTGTGCAGGAGACATTA
AGACATATAACCAGTTCTTCCTCCAATTGAACTCTGTGACCACAGAGGACACAGCCATGTATTACTGTGCAGGAGACATTA
GTTCTAATAACCAGTTCTTCCTCCAATTGAACTCTGTGACCACAGAGGACACAGCCATGTATTACTGTGCAGGAGACATTA
CGAATATGAACCAGTTCTTCCTCCAATTGAACTCTGTGACCACAGAGGACACAGCCATGTATTACTGTGCAGGAGACATTA
CCTACATGAACCAGTTCTTCCTCCAATTGAACTCTGTGACCACAGAGGACACAGCCATGTATTACTGTGCAGGAGACATTA
ATTAAATGAACCAGTTCTTCCTCCAATTGAACTCTGTGACCACAGAGGACACAGCCATGTATTACTGTGCAGGAGACATTA
CTCAAACGAACCAGTTCTTCCTCCAATTGAACTCTGTGACCACAGAGGACACAGCCATGTATTACTGTGCAGGAGACATTA
TCTATAAGAACCAGTTCTTCCTCCAATTGAACTCTGTGACCACAGAGGACACAGCCATGTATTACTGTGCAGGAGACATTA
AGGGCAAGAACCAGTTCTTCCTCCAATTGAACTCTGTGACCACAGAGGACACAGCCATGTATTACTGTGCAGGAGACATT
GGTAAATCAACCAGTTCTTCCTCCAATTGAACTCTGTGACCACAGAGGACACAGCCATGTATTACTGTGCAGGAGACATTA
CGCAAATCAACCAGTTCTTCCTCCAATTGAACTCTGTGACCACAGAGGACACAGCCATGTATTACTGTGCAGGAGACATTA
AGGCATACAACCAGTTCTTCCTCCAATTGAACTCTGTGACCACAGAGGACACAGCCATGTATTACTGTGCAGGAGACATTA
CGCAATACAACCAGTTCTTCCTCCAATTGAACTCTGTGACCACAGAGGACACAGCCATGTATTACTGTGCAGGAGACATTA
GGTATAACAACCAGTTCTTCCTCCAATTGAACTCTGTGACCACAGAGGACACAGCCATGTATTACTGTGCAGGAGACATTA
CGTCAATTATACAGTTCTTCCTCCAATTGAACTCTGTGACCACAGAGGACACAGCCATGTATTACTGTGCAGGAGACATTA
CGTTAAATATACAGTTCTTCCTCCAATTGAACTCTGTGACCACAGAGGACACAGCCATGTATTACTGTGCAGGAGACATTA
CGGAAACGATACAGTTCTTCCTCCAATTGAACTCTGTGACCACAGAGGACACAGCCATGTATTACTGTGCAGGAGACATTA
CGTCAATCATACAGTTCTTCCTCCAATTGAACTCTGTGACCACAGAGGACACAGCCATGTATTACTGTGCAGGAGACATTA

CATCAAAGTCACAGTTCTTCCTCCAATTGAACTCTGTGACCACAGAGGACACAGCCATGTATTACTGTGCAGGAGACATTA
CGGCAATTACACAGTTCTTCCTCCAATTGAACTCTGTGACCACAGAGGACACAGCCATGTATTACTGTGCAGGAGACATTA
AGTCAACTACACAGTTCTTCCTCCAATTGAACTCTGTGACCACAGAGGACACAGCCATGTATTACTGTGCAGGAGACATTA
CGGAAATGACACAGTTCTTCCTCCAATTGAACTCTGTGACCACAGAGGACACAGCCATGTATTACTGTGCAGGAGACATTA
CGTAAATCACACAGTTCTTCCTCCAATTGAACTCTGTGACCACAGAGGACACAGCCATGTATTACTGTGCAGGAGACATTA
CTTCATAGTAACAGTTCTTCCTCCAATTGAACTCTGTGACCACAGAGGACACAGCCATGTATTACTGTGCAGGAGACATTA
CTTCACAGTAACAGTTCTTCCTCCAATTGAACTCTGTGACCACAGAGGACACAGCCATGTATTACTGTGCAGGAGACATTA
CTTCAAACTAACAGTTCTTCCTCCAATTGAACTCTGTGACCACAGAGGACACAGCCATGTATTACTGTGCAGGAGACATTA
CGTCAAATTATAAGTTCTTCCTCCAATTGAACTCTGTGACCACAGAGGACACAGCCATGTATTACTGTGCAGGAGACATTA
CGTCTAATAAGAAGTTCTTCCTCCAATTGAACTCTGTGACCACAGAGGACACAGCCATGTATTACTGTGCAGGAGACATTA
CGTCAATCAAGAAGTTCTTCCTCCAATTGAACTCTGTGACCACAGAGGACACAGCCATGTATTACTGTGCAGGAGACATTA
CGTCAATTATCAAGTTCTTCCTCCAATTGAACTCTGTGACCACAGAGGACACAGCCATGTATTACTGTGCAGGAGACATTA
CGACAAATATCAAGTTCTTCCTCCAATTGAACTCTGTGACCACAGAGGACACAGCCATGTATTACTGTGCAGGAGACATTA
CGGCAATGATCAAGTTCTTCCTCCAATTGAACTCTGTGACCACAGAGGACACAGCCATGTATTACTGTGCAGGAGACATTA
CGTAATAGATCAAGTTCTTCCTCCAATTGAACTCTGTGACCACAGAGGACACAGCCATGTATTACTGTGCAGGAGACATTA
CGTCAAATTCCAAGTTCTTCCTCCAATTGAACTCTGTGACCACAGAGGACACAGCCATGTATTACTGTGCAGGAGACATTA
CGTAAAAGATAAAGTTCTTCCTCCAATTGAACTCTGTGACCACAGAGGACACAGCCATGTATTACTGTGCAGGAGACATTA
CTTAAAAGACAAAGTTCTTCCTCCAATTGAACTCTGTGACCACAGAGGACACAGCCATGTATTACTGTGCAGGAGACATTA
AGTAAAATAAAAAGTTCTTCCTCCAATTGAACTCTGTGACCACAGAGGACACAGCCATGTATTACTGTGCAGGAGACATTA
ACGTCAAAGAACCATTCTTCCTCCAATTGAACTCTGTGACCACAGAGGACACAGCCATGTATTACTGTGCAGGAGACATTA
CGGCAAAGCACCTTGTCTTCCTCCAATTGAACTCTGTGACCACAGAGGACACAGCCATGTATTACTGTGCAGGAGACATTA
CGTCATAGAAACTTGTCTTCCTCCAATTGAACTCTGTGACCACAGAGGACACAGCCATGTATTACTGTGCAGGAGACATTA
CGTCATATAACCCTGTCTTCCTCCAATTGAACTCTGTGACCACAGAGGACACAGCCATGTATTACTGTGCAGGAGACATTA
CGGCAACGAACCCTGTCTTCCTCCAATTGAACTCTGTGACCACAGAGGACACAGCCATGTATTACTGTGCAGGAGACATTA
CGTCCAACAACCCTGTCTTCCTCCAATTGAACTCTGTGACCACAGAGGACACAGCCATGTATTACTGTGCAGGAGACATTA
CGTCATATAACGATGTCTTCCTCCAATTGAACTCTGTGACCACAGAGGACACAGCCATGTATTACTGTGCAGGAGACATTA
CGGAAAAGACACATGTCTTCCTCCAATTGAACTCTGTGACCACAGAGGACACAGCCATGTATTACTGTGCAGGAGACATTA
CGGCAACGATCCCGGTCTTCCTCCAATTGAACTCTGTGACCACAGAGGACACAGCCATGTATTACTGTGCAGGAGACATTA
CGGCAAACATCAAGGTCTTCCTCCAATTGAACTCTGTGACCACAGAGGACACAGCCATGTATTACTGTGCAGGAGACATTA
CGTCACAGCACCCCGTCTTCCTCCAATTGAACTCTGTGACCACAGAGGACACAGCCATGTATTACTGTGCAGGAGACATTA
CGGCACAGACCCACGTCTTCCTCCAATTGAACTCTGTGACCACAGAGGACACAGCCATGTATTACTGTGCAGGAGACATTA
CGGCAACGCACCATCTCTTCCTCCAATTGAACTCTGTGACCACAGAGGACACAGCCATGTATTACTGTGCAGGAGACATTA
ACTTCAAAGAACCAGTTCTCCTCCAATTGAACTCTGTGACCACAGAGGACACAGCCATGTATTACTGTGCAGGAGACATTA
CCGTCAAAGAACCAGTTCTTCCCCAATTGAACTCTGTGACCACAGAGGACACAGCCATGTATTACTGTGCAGGAGACATTA
AAACGTCAAAGAACCAGTTCTTCCTCCAATTGAACTCTGTGACCACAGAGGACACAGCCATGTATTACTGTGCAGACATTA
CGCGAAAGAACCAGTTCTTCCTCCAATTGAACTCTGTGACCACAGAGGACACAGCCATGTATTACTGTGCAGGAGACATTA
GTCGATTCACCATCTCCAGAGACAATGCCAAGAACAACCTGTACCTGCAAATGAGCAGTCTGAGGTCTGAGGACACGGC

ACTACGGTAGTAGCTACGTGGAGCTACTGGTACTTCGATGTCTGG
GTATTTCTGTATGAGATATGGTAACTACTGGTACTTCGATGTCTGG
ACTACGGTAGTAGCTACGTGGAGCTACTGGTACTTCGATGTCTGG
GCAAAAATTTATTACTACGGTAGTAGCTATGCTATGGACTACTGG
TGTGCAAGAATCTACTATGGTAACTACTGGTACTTCGATGTCTGG
ACTACGGTAGTAGCTACGTGGAGCTACTGGTACTTCGATGTCTGG
TGTGCAAGATCGAGAGGCTCAGGCTACTCCCCATTTGCTTACTGG
ACTACGGTAGTAGCTACGTGGAGCTACTGGTACTTCGATGTCTGG
ACTACGGTAGTAGCTACGTGGAGCTACTGGTACTTCGATGTCTGG
TACTGTCATTTTCAGGCATTCCGTCTTAGGTACTTCGATGTCTGG
ACTACGGTAGTAGCTACGTGGAGCTACTGGTACTTCGATGTCTGG
ACTACGGTAGTAGCTACGTGGAGCTACTGGTACTTCGATGTCTGG
ACTACGGTAGTAGCTACGTGGAGCTACTGGTACTTCGATGTCTGG
ACTACGGTAGTAGCTACGTGGAGCTACTGGTACTTCGATGTCTGG
ACTACGGTAGTAGCTACGTGGAGCTACTGGTACTTCGATGTCTGG
ACTACGGTAGTAGCTACGTGGAGCTACTGGTACTTCGATGTCTGG
ACTACGGTAGTAGCTACGTGGAGCTACTGGTACTTCGATGTCTGG
ACTACGGTAGTAGCTACGTGGAGCTACTGGTACTTCGATGTCTGG
ACTACGGTAGTAGCTACGTGGAGCTACTGGTACTTCGATGTCTGG
CTTGTATTACTGTGCACGGGCTGCGAGTGAAGACTTTGCTTACTGG
TACTGTGTTACGACCGGAGACCGTCTTAGGTACTTCGATGTCTGG
TACTGTGTTTTGAACACCATCACTCTTAGGTACTTCGATGTCTGG
TGTGTAGTTTTATGCCATCCGTCCGTTAGGTACTTCGATGTCTGG
TACTGTGACTTCAGTAGCCACCGTCTTAGGTACTTCGATGTCTGG
TATTACTGTGCATGCGTGCGCGGTAGTGAAGACTTTGCTTACTGG
ACTACGGTAGTAGCTACGTGGAGCTACTGGTACTTCGATGTCTGG
ACTACGGTAGTAGCTACGTGGAGCTACTGGTACTTCGATGTCTGG
ACTACGGTAGTAGCTACGTGGAGCTACTGGTACTTCGATGTCTGG
ATTACTGTGCACAGCGCAGCATTGGTGAAGACTTTGCTTACTGG
ACTACGGTAGTAGCTACGTGGAGCTACTGGTACTTCGATGTCTGG
ACTACGGTAGTAGCTACGTGGAGCTACTGGTACTTCGATGTCTGG
ACTACGGTAGTAGCTACGTGGAGCTACTGGTACTTCGATGTCTGG
ACTACGGTAGTAGCTACGTGGAGCTACTGGTACTTCGATGTCTGG
ACTACGGTAGTAGCTACGTGGAGCTACTGGTACTTCGATGTCTGG
ACTACGGTAGTAGCTACGTGGAGCTACTGGTACTTCGATGTCTGG
GTATTTCTGTATGAGATATGGTAACTACTGGTACTTCGATGTCTGG
ACTACGGTAGTAGCTACGTGGAGCTACTGGTACTTCGATGTCTGG
ACTACGGTAGTAGCTACGTGGAGCTACTGGTACTTCGATGTCTGG
ACTACGGTAGTAGCTACGTGGAGCTACTGGTACTTCGATGTCTGG
ACTACGGTAGTAGCTACGTGGAGCTACTGGTACTTCGATGTCTGG
ACTACGGTAGTAGCTACGTGGAGCTACTGGTACTTCGATGTCTGG
ACTACGGTAGTAGCTACGTGGAGCTACTGGTACTTCGATGTCTGG
ACTACGGTAGTAGCTACGTGGAGCTACTGGTACTTCGATGTCTGG
ACTACGGTAGTAGCTACGTGGAGCTACTGGTACTTCGATGTCTGG
ACTACGGTAGTAGCTACGTGGAGCTACTGGTACTTCGATGTCTGG
ACTACGGTAGTAGCTACGTGGAGCTACTGGTACTTCGATGTCTGG
ACTACGGTAGTAGCTACGTGGAGCTACTGGTACTTCGATGTCTGG
ACTACGGTAGTAGCTACGTGGAGCTACTGGTACTTCGATGTCTGG
ACTACGGTAGTAGCTACGTGGAGCTACTGGTACTTCGATGTCTGG

ACTACGGTAGTAGCTACGTGGAGCTACTGGTACTTCGATGTCTGG
ACTACGGTAGTAGCTACGTGGAGCTACTGGTACTTCGATGTCTGG
ACTACGGTAGTAGCTACGTGGAGCTACTGGTACTTCGATGTCTGG
CTACGGTAGTAGCTACGTGGAGCTACTGGTACTTCGATGTCTGG
CTACGGTAGTAGCTACGTGGAGCTACTGGTACTTCGATGTCTGG
CTACGGTAGTAGCTACGTGGAGCTACTGGTACTTCGATGTCTGG
CTACGGTAGTAGCTACGTGGAGCTACTGGTACTTCGATGTCTGG
ACTACGGTAGTAGCTACGTGGAGCTACTGGTACTTCGATGTCTGG
ACTACGGTAGTAGCTACGTGGAGCTACTGGTACTTCGATGTCTGG
CTACGGTAGTAGCTACGTGGAGCTACTGGTACTTCGATGTCTGG
ACTACGGTAGTAGCTACGTGGAGCTACTGGTACTTCGATGTCTGG
CTACGGTAGTAGCTACGTGGAGCTACTGGTACTTCGATGTCTGG
CTACGGTAGTAGCTACGTGGAGCTACTGGTACTTCGATGTCTGG
CTACGGTAGTAGCTACGTGGAGCTACTGGTACTTCGATGTCTGG
CTACGGTAGTAGCTACGTGGAGCTACTGGTACTTCGATGTCTGG
ACTACGGTAGTAGCTACGTGGAGCTACTGGTACTTCGATGTCTGG
CTACGGTAGTAGCTACGTGGAGCTACTGGTACTTCGATGTCTGG
ACTACGGTAGTAGCTACGTGGAGCTACTGGTACTTCGATGTCTGG
ACTACGGTAGTAGCTACGTGGAGCTACTGGTACTTCGATGTCTGG
ACTACGGTAGTAGCTACGTGGAGCTACTGGTACTTCGATGTCTGG
ACTACGGTAGTAGCTACGTGGAGCTACTGGTACTTCGATGTCTGG
CTACGGTAGTAGCTACGTGGAGCTACTGGTACTTCGATGTCTGG
ACTACGGTAGTAGCTACGTGGAGCTACTGGTACTTCGATGTCTGG
ACTACGGTAGTAGCTACGTGGAGCTACTGGTACTTCGATGTCTGG
ACTACGGTAGTAGCTACGTGGAGCTACTGGTACTTCGATGTCTGG
ACTACGGTAGTAGCTACGTGGAGCTACTGGTACTTCGATGTCTGG
ACTACGGTAGTAGCTACGTGGAGCTACTGGTACTTCGATGTCTGG
ACTACGGTAGTAGCTACGTGGAGCTACTGGTACTTCGATGTCTGG
ACTACGGTAGTAGCTACGTGGAGCTACTGGTACTTCGATGTCTGG
ACTACGGTAGTAGCTACGTGGAGCTACTGGTACTTCGATGTCTGG
ACTACGGTAGTAGCTACGTGGAGCTACTGGTACTTCGATGTCTGG
ATTACTACGGTAGCTACGTGGAGCTACTGGTACTTCGATGTCTGG
ACTACTGTAGTAGCTACGTGGAGCTACTGGTACTTCGATGTCTGG
ACTTCGGTAGTAGCTACGTGGAGCTACTGGTACTTCGATGTCTGG
CTACGGTAGTAGCTACGTGGAGCTACTGGTACTTCGATGTCTGG
ACTACGGTAGTAGCTACGTGGAGCTACTGGTACTTCGATGTCTGG
ACTACGGTAGTAGCTACGTGGAGCTACTGGTACTTCGATGTCTGG
CTACGGTAGTAGCTACGTGGAGCTACTGGTACTTCGATGTCTGG
CTACGGTAGTAGCTACGTGGAGCTACTGGTACTTCGATGTCTGG
CTACGGTAGTAGCTACGTGGAGCTACTGGTACTTCGATGTCTGG
CTACGGTAGTAGCTACGTGGAGCTACTGGTACTTCGATGTCTGG
CTACGGTAGTAGCTACGTGGAGCTACTGGTACTTCGATGTCTGG
ACTACGGTAGTAGCTACGTGGAGCTACTGGTACTTCGATGTCTGG
ACTACGGTAGTAGCTACGTGGAGCTACTGGTACTTCGATGTCTGG
ACTACGGTAGTAGCTACGTGGAGCTACTGGTACTTCGATGTCTGG
ACTACGGTAGTAGCTACGTGGAGCTACTGGTACTTCGATGTCTGG
ACTACGGTAGTAGCTACGTGGAGCTACTGGTACTTCGATGTCTGG
ACTACGGTAGTAGCTACGTGGAGCTACTGGTACTTCGATGTCTGG
ACTACGGTAGTAGCTACGTGGAGCTACTGGTACTTCGATGTCTGG
ACTACGGTAGTAGCTACGTGGAGCTACTGGTACTTCGATGTCTGG

ACTACGGTAGTAGCTACGTGGAGCTACTGGTACTTCGATGTCTGG
ACTACGGTAGTAGCTACGTGGAGCTACTGGTACTTCGATGTCTGG
ACTACGGTAGTAGCTACGTGGAGCTACTGGTACTTCGATGTCTGG
ACTACGGTAGTAGCTACGTGGAGCTACTGGTACTTCGATGTCTGG
ACTACGGTAGTAGCTACGTGGAGCTACTGGTACTTCGATGTCTGG
ACTACGGTAGTAGCTACGTGGAGCTACTGGTACTTCGATGTCTGG
ACTACGGTAGTAGCTACGTGGAGCTACTGGTACTTCGATGTCTGG
ACTACGGTAGTAGCTACGTGGAGCTACTGGTACTTCGATGTCTGG
ACTACGGTAGTAGCTACGTGGAGCTACTGGTACTTCGATGTCTGG
ACTACGGTAGTAGCTACGTGGAGCTACTGGTACTTCGATGTCTGG
CTACGGTAGTAGCTACGTGGAGCTACTGGTACTTCGATGTCTGG
CTACGGTAGTAGCTACGTGGAGCTACTGGTACTTCGATGTCTGG
ACTACGGTAGTAGCTACGTGGAGCTACTGGTACTTCGATGTCTGG
ACTACGGTAGTAGCTACGTGGAGCTACTGGTACTTCGATGTCTGG
ACTACGGTAGTAGCTACGTGGAGCTACTGGTACTTCGATGTCTGG
ACTACGGTAGTAGCTACGTGGAGCTACTGGTACTTCGATGTCTGG
ACTACGGTAGTAGCTACGTGGAGCTACTGGTACTTCGATGTCTGG
ACTACGGTAGTAGCTACGTGGAGCTACTGGTACTTCGATGTCTGG
ACTACGGTAGTAGCTACGTGGAGCTACTGGTACTTCGATGTCTGG
ACTACGGTAGTAGCTACGTGGAGCTACTGGTACTTCGATGTCTGG
ACTACGGTAGTAGCTACGTGGAGCTACTGGTACTTCGATGTCTGG
ACTACGGTAGTAGCTACGTGGAGCTACTGGTACTTCGATGTCTGG
CTACGGTAGTAGCTACGTGGAGCTACTGGTACTTCGATGTCTGG
ACTACGGTAGTAGCTACGTGGAGCTACTGGTACTTCGATGTCTGG
ACTACGGTAGTAGCTACGTGGAGCTACTGGTACTTCGATGTCTGG
ACTACGGTAGTAGCTACGTGGAGCTACTGGTACTTCGATGTCTGG
ACTACGGTAGTAGCTACGTGGAGCTACTGGTACTTCGATGTCTGG
CTACGGTAGTAGCTACGTGGAGCTACTGGTACTTCGATGTCTGG
ACTACGGTAGTAGCTACGTGGAGCTACTGGTACTTCGATGTCTGG
CTACGGTAGTAGCTACGTGGAGCTACTGGTACTTCGATGTCTGG
ACTACGGTAGTAGCTACGTGGAGCTACTGGTACTTCGATGTCTGG
ACTACGGTAGTAGCTACGTGGAGCTACTGGTACTTCGATGTCTGG
ACTACGGTAGTAGCTACGTGGAGCTACTGGTACTTCGATGTCTGG
ACTACGGTAGTAGCTACGTGGAGCTACTGGTACTTCGATGTCTGG
ACTACGGTAGTAGCTACGTGGAGCTACTGGTACTTCGATGTCTGG
ACTACGGTAGTAGCTACGTGGAGCTACTGGTACTTCGATGTCTGG
ACTACGGTAGTAGCTACGTGGAGCTACTGGTACTTCGATGTCTGG
ACTACGGTAGTAGCTACGTGGAGCTACTGGTACTTCGATGTCTGG
ACTACGGTAGTAGCTACGTGGAGCTACTGGTACTTCGATGTCTGG
ACTACGGTAGTAGCTACGTGGAGCTACTGGTACTTCGATGTCTGG
ACTACGGTAGTAGCTACGTGGAGCTACTGGTACTTCGATGTCTGG
ACTACGGTAGTAGCTACGTGGAGCTACTGGTACTTCGATGTCTGG
ACTACGGTAGTAGCTACGTGGAGCTACTGGTACTTCGATGTCTGG
ACTACGGTAGTAGCTACGTGGAGCTACTGGTACTTCGATGTCTGG
ACTACGGTAGTAGCTACGTGGAGCTACTGGTACTTCGATGTCTGG
ACTACGGTAGTAGCTACGTGGAGCTACTGGTACTTCGATGTCTGG
ACTACGGTAGTAGCTACGTGGAGCTACTGGTACTTCGATGTCTGG
ACTACGGTAGTAGCTACGTGGAGCTACTGGTACTTCGATGTCTGG
ACTACGGTAGTAGCTACGTGGAGCTACTGGTACTTCGATGTCTGG
ACTACGGTAGTAGCTACGTGGAGCTACTGGTACTTCGATGTCTGG

ACTACGGTAGTAGCTACGTGGAGCTACTGGTACTTCGATGTCTGG
ACTACGGTAGTAGCTACGTGGAGCTACTGGTACTTCGATGTCTGG
ACTACGGTAGTAGCTACGTGGAGCTACTGGTACTTCGATGTCTGG
ACTACGGTAGTAGCTACGTGGAGCTACTGGTACTTCGATGTCTGG
CTACGGTAGTAGCTACGTGGAGCTACTGGTACTTCGATGTCTGG
ACTACGGTAGTAGCTACGTGGAGCTACTGGTACTTCGATGTCTGG
ACTACGGTAGTAGCTACGTGGAGCTACTGGTACTTCGATGTCTGG
ACTACGGTAGTAGCTACGTGGAGCTACTGGTACTTCGATGTCTGG
ACTACGGTAGTAGCTACGTGGAGCTACTGGTACTTCGATGTCTGG
CTACGGTAGTAGCTACGTGGAGCTACTGGTACTTCGATGTCTGG
ACTACGGTAGTAGCTACGTGGAGCTACTGGTACTTCGATGTCTGG
ACTACGGTAGTAGCTACGTGGAGCTACTGGTACTTCGATGTCTGG
ACTACGGTAGTAGCTACGTGGAGCTACTGGTACTTCGATGTCTGG
ACTACGGTAGTAGCTACGTGGAGCTACTGGTACTTCGATGTCTGG
ACTACGGTAGTAGCTACGTGGAGCTACTGGTACTTCGATGTCTGG
ACTACGGTAGTAGCTACGTGGAGCTACTGGTACTTCGATGTCTGG
ACTACGGTAGTAGCTACGTGGAGCTACTGGTACTTCGATGTCTGG
ACTACGGTAGTAGCTACGTGGAGCTACTGGTACTTCGATGTCTGG
ACTACGGTAGTAGCTACGTGGAGCTACTGGTACTTCGATGTCTGG
ACTACGGTAGTAGCTACGTGGAGCTACTGGTACTTCGATGTCTGG
ACTACGGTAGTAGCTACGTGGAGCTACTGGTACTTCGATGTCTGG
ACTACGGTAGTAGCTACGTGGAGCTACTGGTACTTCGATGTCTGG
ACTACGGTAGTAGCTACGTGGAGCTACTGGTACTTCGATGTCTGG
ACTACGGTAGTAGCTACGTGGAGCTACTGGTACTTCGATGTCTGG
ACTACGGTAGTAGCTACGTGGAGCTACTGGTACTTCGATGTCTGG
ACTACGGTAGTAGCTACGTGGAGCTACTGGTACTTCGATGTCTGG
CAAAAATTTATTACTACGGTAGTAGCTATGCTATGGACTACTGG
ACTGCCGTCTATTACTGTATAACGCGTGAAGACTTTGCTTACTGG
ATCTATTACTGTAGTCGGTACAGTAGTGAAGACTTTGCTTACTGG
GTCTATTACTGTACCTCGTGCCGTCTTAGGTACTTCGATGTCTGG
TTCTGTTACCAACGTGTAAGCCGTCTTAGGTACTTCGATGTCTGG
ACTACGGTAGTAGCTACGTGGAGCTACTGGTACTTCGATGTCTGG
ACTACGGTAGTAGCTACGTGGAGCTACTGGTACTTCGATGTCTGG
ACTACGGTAGTAGCTACGTGGAGCTACTGGTACTTCGATGTCTGG
ACTACGGTAGTAGCTACGTGGAGCTACTGGTACTTCGATGTCTGG
ACTACGGTAGTAGCTACGTGGAGCTACTGGTACTTCGATGTCTGG
ACTACGGTAGTAGCTACGTGGAGCTACTGGTACTTCGATGTCTGG
GTATTTCTGTATGAGATATGGTAACTACTGGTACTTCGATGTCTGG
ACTACGGTAGTAGCTACGTGGAGCTACTGGTACTTCGATGTCTGG
ACTACGGTAGTAGCTACGTGGAGCTACTGGTACTTCGATGTCTGG
ACTACGGTAGTAGCTACGTGGAGCTACTGGTACTTCGATGTCTGG
CTACGGTAGTAGCTACGTGGAGCTACTGGTACTTCGATGTCTGG
ACTACGGTAGTAGCTACGTGGAGCTACTGGTACTTCGATGTCTGG
ACTACGGTAGTAGCTACGTGGAGCTACTGGTACTTCGATGTCTGG
ACTACGGTAGTAGCTACGTGGAGCTACTGGTACTTCGATGTCTGG
ACTACGGTAGTAGCTACGTGGAGCTACTGGTACTTCGATGTCTGG
ACTACGGTAGTAGCTACGTGGAGCTACTGGTACTTCGATGTCTGG
ACTACGGTAGTAGCTACGTGGAGCTACTGGTACTTCGATGTCTGG
ACTACGGTAGTAGCTACGTGGAGCTACTGGTACTTCGATGTCTGG
ACTACGGTAGTAGCTACGTGGAGCTACTGGTACTTCGATGTCTGG

ACTACGGTAGTAGCTACGTGGAGCTACTGGTACTTCGATGTCTGG
CTACGGTAGTAGCTACGTGGAGCTACTGGTACTTCGATGTCTGG
ACTACGGTAGTAGCTACGTGGAGCTACTGGTACTTCGATGTCTGG
ACTACGGTAGTAGCTACGTGGAGCTACTGGTACTTCGATGTCTGG
ACTACGGTAGTAGCTACGTGGAGCTACTGGTACTTCGATGTCTGG
ACTACGGTAGTAGCTACGTGGAGCTACTGGTACTTCGATGTCTGG
CTACGGTAGTAGCTACGTGGAGCTACTGGTACTTCGATGTCTGG
CTACGGTAGTAGCTACGTGGAGCTACTGGTACTTCGATGTCTGG
CTACGGTAGTAGCTACGTGGAGCTACTGGTACTTCGATGTCTGG
ACTACGGTAGTAGCTACGTGGAGCTACTGGTACTTCGATGTCTGG
ACTACGGTAGTAGCTACGTGGAGCTACTGGTACTTCGATGTCTGG
ACTACGGTAGTAGCTACGTGGAGCTACTGGTACTTCGATGTCTGG
ACTACGGTAGTAGCTACGTGGAGCTACTGGTACTTCGATGTCTGG
ACTACGGTAGTAGCTACGTGGAGCTACTGGTACTTCGATGTCTGG
CTACGGTAGTAGCTACGTGGAGCTACTGGTACTTCGATGTCTGG
ACTACGGTAGTAGCTACGTGGAGCTACTGGTACTTCGATGTCTGG
ACTACGGTAGTAGCTACGTGGAGCTACTGGTACTTCGATGTCTGG
CTACGGTAGTAGCTACGTGGAGCTACTGGTACTTCGATGTCTGG
ACTACGGTAGTAGCTACGTGGAGCTACTGGTACTTCGATGTCTGG
ACTACGGTAGTAGCTACGTGGAGCTACTGGTACTTCGATGTCTGG
CTACGGTAGTAGCTACGTGGAGCTACTGGTACTTCGATGTCTGG
ACTACGGTAGTAGCTACGTGGAGCTACTGGTACTTCGATGTCTGG
ACTACGGTAGTAGCTACGTGGAGCTACTGGTACTTCGATGTCTGG
CTACGGTAGTAGCTACGTGGAGCTACTGGTACTTCGATGTCTGG
ACTACGGTAGTAGCTACGTGGAGCTACTGGTACTTCGATGTCTGG
ACTACGGTAGTAGCTACGTGGAGCTACTGGTACTTCGATGTCTGG
ACTACGGTAGTAGCTACGTGGAGCTACTGGTACTTCGATGTCTGG
ACTACGGTAGTAGCTACGTGGAGCTACTGGTACTTCGATGTCTGG
ACTACGGTAGTAGCTACGTGGAGCTACTGGTACTTCGATGTCTGG
ACTACGGTAGTAGCTACGTGGAGCTACTGGTACTTCGATGTCTGG
ACTACGGTAGTAGCTACGTGGAGCTACTGGTACTTCGATGTCTGG
ACTACGGTAGTAGCTACGTGGAGCTACTGGTACTTCGATGTCTGG
ACTACGGTAGTAGCTACGTGGAGCTACTGGTACTTCGATGTCTGG
ACTACGGTAGTAGCTACGTGGAGCTACTGGTACTTCGATGTCTGG
ACTACGGTAGTAGCTACGTGGAGCTACTGGTACTTCGATGTCTGG
ACTACGGTAGTAGCTACGTGGAGCTACTGGTACTTCGATGTCTGG
ACTACGGTAGTAGCTACGTGGAGCTACTGGTACTTCGATGTCTGG
ACTACGGTAGTAGCTACGTGGAGCTACTGGTACTTCGATGTCTGG
ACTACGGTAGTAGCTACGTGGAGCTACTGGTACTTCGATGTCTGG
ACTACGGTAGTAGCTACGTGGAGCTACTGGTACTTCGATGTCTGG
ACTACGGTAGTAGCTACGTGGAGCTACTGGTACTTCGATGTCTGG
ACTACGGTAGTAGCTACGTGGAGCTACTGGTACTTCGATGTCTGG
ACTACGGTAGTAGCTACGTGGAGCTACTGGTACTTCGATGTCTGG
ACTACGGTAGTAGCTACGTGGAGCTACTGGTACTTCGATGTCTGG
CTACGGTAGTAGCTACGTGGAGCTACTGGTACTTCGATGTCTGG
ACTACGGTAGTAGCTACGTGGAGCTACTGGTACTTCGATGTCTGG
ACTACGGTAGTAGCTACGTGGAGCTACTGGTACTTCGATGTCTGG
ACTACGGTAGTAGCTACGTGGAGCTACTGGTACTTCGATGTCTGG
ACTACGGTAGTAGCTACGTGGAGCTACTGGTACTTCGATGTCTGG
CTACGGTAGTAGCTACGTGGAGCTACTGGTACTTCGATGTCTGG

CTACGGTAGTAGCTACGTGGAGCTACTGGTACTTCGATGTCTGG
CTACGGTAGTAGCTACGTGGAGCTACTGGTACTTCGATGTCTGG
CTACGGTAGTAGCTACGTGGAGCTACTGGTACTTCGATGTCTGG
ACTACGGTAGTAGCTACGTGGAGCTACTGGTACTTCGATGTCTGG
ACTACGGTAGTAGCTACGTGGAGCTACTGGTACTTCGATGTCTGG
ACTACGGTAGTAGCTACGTGGAGCTACTGGTACTTCGATGTCTGG
CTACGGTAGTAGCTACGTGGAGCTACTGGTACTTCGATGTCTGG
ACTACGGTAGTAGCTACGTGGAGCTACTGGTACTTCGATGTCTGG
CTACGGTAGTAGCTACGTGGAGCTACTGGTACTTCGATGTCTGG
CTACGGTAGTAGCTACGTGGAGCTACTGGTACTTCGATGTCTGG
CTACGGTAGTAGCTACGTGGAGCTACTGGTACTTCGATGTCTGG
ACTACGGTAGTAGCTACGTGGAGCTACTGGTACTTCGATGTCTGG
ACTACGGTAGTAGCTACGTGGAGCTACTGGTACTTCGATGTCTGG
ACTACGGTAGTAGCTACGTGGAGCTACTGGTACTTCGATGTCTGG
ACTACGGTAGTAGCTACGTGGAGCTACTGGTACTTCGATGTCTGG
CTACGGTAGTAGCTACGTGGAGCTACTGGTACTTCGATGTCTGG
CTACGGTAGTAGCTACGTGGAGCTACTGGTACTTCGATGTCTGG
CTACGGTAGTAGCTACGTGGAGCTACTGGTACTTCGATGTCTGG
ACTACGGTAGTAGCTACGTGGAGCTACTGGTACTTCGATGTCTGG
CTACGGTAGTAGCTACGTGGAGCTACTGGTACTTCGATGTCTGG
CTACGGTAGTAGCTACGTGGAGCTACTGGTACTTCGATGTCTGG
ACTACGGTAGTAGCTACGTGGAGCTACTGGTACTTCGATGTCTGG
ACTACGGTAGTAGCTACGTGGAGCTACTGGTACTTCGATGTCTGG
CTACGGTAGTAGCTACGTGGAGCTACTGGTACTTCGATGTCTGG
ACTACGGTAGTAGCTACGTGGAGCTACTGGTACTTCGATGTCTGG
CTACGGTAGTAGCTACGTGGAGCTACTGGTACTTCGATGTCTGG
CTACGGTAGTAGCTACGTGGAGCTACTGGTACTTCGATGTCTGG
ACTACGGTAGTAGCTACGTGGAGCTACTGGTACTTCGATGTCTGG
ACTACGGTAGTAGCTACGTGGAGCTACTGGTACTTCGATGTCTGG
ACTACGGTAGTAGCTACGTGGAGCTACTGGTACTTCGATGTCTGG
ACTACGGTAGTAGCTACGTGGAGCTACTGGTACTTCGATGTCTGG
CTACGGTAGTAGCTACGTGGAGCTACTGGTACTTCGATGTCTGG
CTACGGTAGTAGCTACGTGGAGCTACTGGTACTTCGATGTCTGG
ACTACGGTAGTAGCTACGTGGAGCTACTGGTACTTCGATGTCTGG
CTACGGTAGTAGCTACGTGGAGCTACTGGTACTTCGATGTCTGG
ACTACGGTAGTAGCTACGTGGAGCTACTGGTACTTCGATGTCTGG
ACTACGGTAGTAGCTACGTGGAGCTACTGGTACTTCGATGTCTGG
ACTACGGTAGTAGCTACGTGGAGCTACTGGTACTTCGATGTCTGG
ACTACGGTAGTAGCTACGTGGAGCTACTGGTACTTCGATGTCTGG
ACTACGGTAGTAGCTACGTGGAGCTACTGGTACTTCGATGTCTGG
ACTACGGTAGTAGCTACGTGGAGCTACTGGTACTTCGATGTCTGG
ACTACGGTAGTAGCTACGTGGAGCTACTGGTACTTCGATGTCTGG
ACTACGGTAGTAGCTACGTGGAGCTACTGGTACTTCGATGTCTGG
ACTACGGTAGTAGCTACGTGGAGCTACTGGTACTTCGATGTCTGG
ACTACGGTAGTAGCTACGTGGAGCTACTGGTACTTCGATGTCTGG
ACTACGGTAGTAGCTACGTGGAGCTACTGGTACTTCGATGTCTGG
CTACGGTAGTAGCTACGTGGAGCTACTGGTACTTCGATGTCTGG
CTACGGTAGTAGCTACGTGGAGCTACTGGTACTTCGATGTCTGG
ACTACGGTAGTAGCTACGTGGAGCTACTGGTACTTCGATGTCTGG
CTACGGTAGTAGCTACGTGGAGCTACTGGTACTTCGATGTCTGG

CTACGGTAGTAGCTACGTGGAGCTACTGGTACTTCGATGTCTGG
CTACGGTAGTAGCTACGTGGAGCTACTGGTACTTCGATGTCTGG
CTACGGTAGTAGCTACGTGGAGCTACTGGTACTTCGATGTCTGG
ACTACGGTAGTAGCTACGTGGAGCTACTGGTACTTCGATGTCTGG
CTACGGTAGTAGCTACGTGGAGCTACTGGTACTTCGATGTCTGG
CTACGGTAGTAGCTACGTGGAGCTACTGGTACTTCGATGTCTGG
CTACGGTAGTAGCTACGTGGAGCTACTGGTACTTCGATGTCTGG
CTACGGTAGTAGCTACGTGGAGCTACTGGTACTTCGATGTCTGG
CTACGGTAGTAGCTACGTGGAGCTACTGGTACTTCGATGTCTGG
CTACGGTAGTAGCTACGTGGAGCTACTGGTACTTCGATGTCTGG
CTACGGTAGTAGCTACGTGGAGCTACTGGTACTTCGATGTCTGG
CTACGGTAGTAGCTACGTGGAGCTACTGGTACTTCGATGTCTGG
CTACGGTAGTAGCTACGTGGAGCTACTGGTACTTCGATGTCTGG
CTACGGTAGTAGCTACGTGGAGCTACTGGTACTTCGATGTCTGG
CTACGGTAGTAGCTACGTGGAGCTACTGGTACTTCGATGTCTGG
CTACGGTAGTAGCTACGTGGAGCTACTGGTACTTCGATGTCTGG
ACTACGGTAGTAGCTACGTGGAGCTACTGGTACTTCGATGTCTGG
CTACGGTAGTAGCTACGTGGAGCTACTGGTACTTCGATGTCTGG
ACTACGGTAGTAGCTACGTGGAGCTACTGGTACTTCGATGTCTGG
CTACGGTAGTAGCTACGTGGAGCTACTGGTACTTCGATGTCTGG
ACTACGGTAGTAGCTACGTGGAGCTACTGGTACTTCGATGTCTGG
CTACGGTAGTAGCTACGTGGAGCTACTGGTACTTCGATGTCTGG
CTACGGTAGTAGCTACGTGGAGCTACTGGTACTTCGATGTCTGG
ACTACGGTAGTAGCTACGTGGAGCTACTGGTACTTCGATGTCTGG
CTACGGTAGTAGCTACGTGGAGCTACTGGTACTTCGATGTCTGG
CTACGGTAGTAGCTACGTGGAGCTACTGGTACTTCGATGTCTGG
ACTACGGTAGTAGCTACGTGGAGCTACTGGTACTTCGATGTCTGG
ACTACGGTAGTAGCTACGTGGAGCTACTGGTACTTCGATGTCTGG
ACTACGGTAGTAGCTACGTGGAGCTACTGGTACTTCGATGTCTGG
CTACGGTAGTAGCTACGTGGAGCTACTGGTACTTCGATGTCTGG
ACTACGGTAGTAGCTACGTGGAGCTACTGGTACTTCGATGTCTGG
CTACGGTAGTAGCTACGTGGAGCTACTGGTACTTCGATGTCTGG
CTACGGTAGTAGCTACGTGGAGCTACTGGTACTTCGATGTCTGG
ACTACGGTAGTAGCTACGTGGAGCTACTGGTACTTCGATGTCTGG
CTACGGTAGTAGCTACGTGGAGCTACTGGTACTTCGATGTCTGG
ACTACGGTAGTAGCTACGGGGAGCTACTGGTACTTCGATGTCTGG
TTGTATTACTGTGCAATACCCCGTCTTAGGTACTTCGATGTCTGG

sample_name	productive_frequency	templates	amino_acid
TCL_F3_2	99.504	17641	CARIYYGNYWYFDVW
TCL_F3_2	0.011	2	CMRYGNYWYFDVW
TCL_F3_2	0.006	1	CARIYYGNYWYFDVW
TCL_F3_2	0.006	1	CARIYYGNYWYFDVW
TCL_F3_2	0.006	1	CANGRLRYFDVW
TCL_F3_2	0.006	1	CRTGEDFAYW
TCL_F3_2	0.006	1	CARIYYGNYWYFDVW
TCL_F3_2	0.006	1	CARIYYGNYWYFDVW
TCL_F3_2	0.006	1	CARIYYGNYWYFDVW
TCL_F3_2	0.006	1	CARIYYGNYWYFDVW
TCL_F3_2	0.006	1	CARIYYGNYWYFDVW
TCL_F3_2	0.006	1	ARIYYGNYWYFDVW
TCL_F3_2	0.006	1	CARIYYGNYWYFDVW
TCL_F3_2	0.006	1	CARIYYGNYWYFDVW
TCL_F3_2	0.006	1	CASGELDRLRYFDVW
TCL_F3_2	0.006	1	CARIYYGNYWYFDVW
TCL_F3_2	0.006	1	CARIYYGNYWYFDVW
TCL_F3_2	0.006	1	CARIYYGNYWYFDVW
TCL_F3_2	0.006	1	CAIILVCRLRYFDVW
TCL_F3_2	0.006	1	CARIYYGNYWYFDVW
TCL_F3_2	0.006	1	CARIYYGNYWYFDVW
TCL_F3_2	0.006	1	CARIYYGNYWYFDVW
TCL_F3_2	0.006	1	CARIYYGNYWYFDVW
TCL_F3_2	0.006	1	CARIYYGNYWYFDVW
TCL_F3_2	0.006	1	CARIYYGNYWYFDVW
TCL_F3_2	0.006	1	CARIYYGNYWYFDVW
TCL_F3_2	0.006	1	CARIYYGNYWYFDVW
TCL_F3_2	0.006	1	CARIYYGNYWYFDVW
TCL_F3_2	0.006	1	CARIYYGNYWYFDVW
TCL_F3_2	0.006	1	CARIYYGNYWYFDVW
TCL_F3_2	0.006	1	CARIYYGNYWYFDVW
TCL_F3_2	0.006	1	CARIYYGNYWYFDVW
TCL_F3_2	0.006	1	CARIYYGNYWYFDVW
TCL_F3_2	0.006	1	CARIYYGNYWYFDVW
TCL_F3_2	0.006	1	CARIYYGNYWYFDVW
TCL_F3_2	0.006	1	CANGRLRYFDVW
TCL_F3_2	0.006	1	CRTGEDFAYW
TCL_F3_2	0.006	1	CARIYYCNYWYFDVW
TCL_F3_2	0.006	1	CARIYGNYWYFDVW
TCL_F3_2	0.006	1	CARIYYGNYWYFDVW
TCL_F3_2	0.006	1	CARIYYGNYWYFDVW
TCL_F3_2	0.006	1	CARIYYGNYWYFDVW
TCL_F3_2	0.006	1	CARIYYGNYWYFDVW
TCL_F3_2	0.006	1	CARIYYGNYWYFDVW
TCL_F3_2	0.006	1	CARIYYGNYWYFDVW
TCL_F3_2	0.006	1	CARIYYGNYWYFDVW
TCL_F3_2	0.006	1	CARIYYGNYWYFDVW
TCL_F3_2	0.006	1	CARIYYGNYWYFDVW
TCL_F3_2	0.006	1	CARIYYGNYWYFDVW
TCL_F3_2	0.006	1	CARIYYGNYWYFDVW
TCL_F3_2	0.006	1	CARIYYGNYWYFDVW
TCL_F3_2	0.006	1	CARIYYGNYWYFDVW
TCL_F3_2	0.006	1	CARIYYGNYWYFDVW
TCL_F3_2	0.006	1	CARIYYGNYWYFDVW
TCL_F3_2	0.006	1	CARIYYGNYWYFDVW
TCL_F3_2	0.006	1	CARIYYGNYWYFDVW
TCL_F3_2	0.006	1	CARIYYGNYWYFDVW
TCL_F3_2	0.006	1	CARIYYGNYWYFDVW
TCL_F3_2	0.006	1	CARIYYGNYWYFDVW
TCL_F3_2	0.006	1	CARIYYGNYWYFDVW
TCL_F3_2	0.006	1	CARIYYGNYWYFDVW
TCL_F3_2	0.006	1	CARIYYGNYWYFDVW
TCL_F3_2	0.006	1	CARIYYGNYWYFDVW
TCL_F3_2	0.006	1	CARIYYGNYWYFDVW
TCL_F3_2	0.006	1	CARIYYGNYWYFDVW
TCL_F3_2	0.006	1	CARIYYGNYWYFDVW
TCL_F3_2	0.006	1	CARIYYGNYWYFDVW
TCL_F3_2	0.006	1	CARIYYGNYWYFDVW
TCL_F3_2	0.006	1	CARIYYGNYWYFDVW
TCL_F3_2	0.006	1	CARIYYGNYWYFDVW
TCL_F3_2	0.006	1	CARIYYGNYWYFDVW
TCL_F3_2	0.006	1	CARIYYGNYWYFDVW
TCL_F3_2	0.006	1	CARIYYGNYWYFDVW
TCL_F3_2	0.006	1	CARIYYGNYWYFDVW
TCL_F3_2	0.006	1	CARIYYGNYWYFDVW
TCL_F3_2	0.006	1	CARIYYGNYWYFDVW
TCL_F3_2	0.006	1	CARIYYGNYWYFDVW
TCL_F3_2	0.006	1	CARIYYGNYWYFDVW
TCL_F3_2	0.006	1	CARIYYGNYWYFDVW
TCL_F3_2	0.006	1	CARIYYGNYWYFDVW
TCL_F3_2	0.006	1	CARIYYGNYWYFDVW
TCL_F3_2	0.006	1	CARIYYGNYWYFDVW
TCL_F3_2	0.006	1	CARIYYGNYWYFDVW
TCL_F3_2	0.006	1	CARIYYGNYWYFDVW
TCL_F3_2	0.006	1	CARIYYGNYWYFDVW
TCL_F3_2	0.006	1	CARIYYGNYWYFDVW
TCL_F3_2	0.006	1	CARIYYGNYWYFDVW
TCL_F3_2	0.006	1	CARIYYGNYWYFDGW
total	100.000	17729	

rearrangement

CACTGACTGTAGACACATCCTCCAGCACAGCCTACATGCAGCTCAGCAGCCTGACATCTGAGGACTCTGCGGTCTATTACTGTGCAAGAATCTACTATGGTAACTACTGGTACTTCGATGTCTGG
GATTCACTATCTTCAGAGACAATGACAAGAGCACCCTGTACCTGCAGATGAGCAATGTGCGATCGGAGGACACAGCCACGTATTTCTGTATGAGATATGGTAACTACTGGTACTTCGATGTCTGG
CCAGGACTGTAGACACATCCTCCAGCACAGCCTACATGCAGCTCAGCAGCCTGACATCTGAGGACTCTGCGGTCTATTACTGTGCAAGAATCTACTATGGTAACTACTGGTACTTCGATGTCTGG
ACACTGACTGTAGACACATCCTCAGCACAGCCTACATGCAGCTCAGCAGCCTGACATCTGAGGACTCTGCGGTCTATTACTGTGCAAGAATCTACTATGGTAACTACTGGTACTTCGATGTCTGG
GTCGGTTCACCATCTCCAGAGATAATTCTCAAAACATCCTCTATCTTCAAATGAACACCCTGAGAGCTGAGGCCAGTGCCACTTATTACTGTGCAAACGGCCGTCTTAGGTACTTCGATGTCTGG
TGAATGGGAGGTTCACCATCTCAAGAGATGATTCCAAAAGTAGTGTCTACCTGCAAATGAACAGCTTAAGAGCTGAAGATACTGGCATTTATTACTGTCGAACGGGTGAAGACTTTGCTTACTGG
ACACTGACTGTAGACACATCCCCAGCACAGCCTACATGCAGCTCAGCAGCCTGACATCTGAGGACTCTGCGGTCTATTACTGTGCAAGAATCTACTATGGTAACTACTGGTACTTCGATGTCTGG
CACTGACTTCAGACAAATCCTCCAGCACAGCCTACATGGAGCTCAGCAGCCTGACCTCTGAGGACTCTGCGGTCTATTACTGTGCAAGAATCTACTATGGTAACTACTGGTACTTCGATGTCTGG
ACACTGACTGTAGACACATCCTCCAGACAGCCTACATGCAGCTCAGCAGCCTGACATCTGAGGACTCTGCGGTCTATTACTGTGCAAGAATCTACTATGGTAACTACTGGTACTTCGATGTCTGG
ACACTGACTGTAGACACATCTCCAGCACAGCCTACATGCAGCTCAGCAGCCTGACATCTGAGGACTCTGCGGTCTATTACTGTGCAAGAATCTACTATGGTAACTACTGGTACTTCGATGTCTGG
CATTGACTGCAAACACATCCTCGAGCACAGCCTACATGGAACTCAGCAGCCTCACATCTGAGGACTCTGCGGTCTATTACTGTGCAAGAATCTACTATGGTAACTACTGGTACTTCGATGTCTGG
CCACACTGACTGTAGACACATCCTCCAGCACAGCCTACATGCAGCTCAGCCTGACATCTGAGGACTCTGCGGTCTATTACTGTGCAAGAATCTACTATGGTAACTACTGGTACTTCGATGTCTGG
ACACTGACTGTGACACATCCTCCAGCACAGCCTACATGCAGCTCAGCAGCCTGACATCTGAGGACTCTGCGGTCTATTACTGTGCAAGAATCTACTATGGTAACTACTGGTACTTCGATGTCTGG
ACACTGACTGTAGACACATCCTCCAGCACAGCCACATGCAGCTCAGCAGCCTGACATCTGAGGACTCTGCGGTCTATTACTGTGCAAGAATCTACTATGGTAACTACTGGTACTTCGATGTCTGG
CCATCACTCGAGACACTTCCAAGAACCAGTTCTACCTGCAGTTGAATTCTGTGACTACTGAGGACACAGCCATATATTACTGTGCAAGTGGCGAGCTGGACCGTCTTAGGTACTTCGATGTCTGG
CCACACTGACTGTAGACACATCCAGCACAGCCTACATGCAGCTCAGCAGCCTGACATCTGAGGACTCTGCGGTCTATTACTGTGCAAGAATCTACTATGGTAACTACTGGTACTTCGATGTCTGG
CATTGACTGCAGACAAATCCTCCAGCACAGCCTACATGCAACTGAGCAGCCTGACATCTGAGGACTCTGCAGTCTATTACTGTGCAAGAATCTACTATGGTAACTACTGGTACTTCGATGTCTGG
CATTGACTGTAGACAAATCCTCCAGCACAGCCTACATGCAGCTCAACAGCCTGACATCTGAGGACTCTGCAGTCTATTACTGTGCAAGAATCTACTATGGTAACTACTGGTACTTCGATGTCTGG
GCATCAGCAAGGACAACTCCAAGAGCCAAGTTTTCTTAAAAATGAACAGTCTGCAAACTGATGACACAGCCAGGTACTACTGTGCCATAATATTAGTGTGCCGTCTTAGGTACTTCGATGTCTGG
CACGGCAGGTAGACACATCCTCCAGCACAGCCTACATGCAGCTCAGCAGCCTGACATCTGAGGACTCTGCGGTCTATTACTGTGCAAGAATCTACTATGGTAACTACTGGTACTTCGATGTCTGG
ACACTGACTGAGACACATCCTCCAGCACAGCCTACATGCAGCTCAGCAGCCTGACATCTGAGGACTCTGCGGTCTATTACTGTGCAAGAATCTACTATGGTAACTACTGGTACTTCGATGTCTGG
ACACTGACTGTAGACACATCCTCCAGCACAGCCTCATGCAGCTCAGCAGCCTGACATCTGAGGACTCTGCGGTCTATTACTGTGCAAGAATCTACTATGGTAACTACTGGTACTTCGATGTCTGG
ACACTGACTGTAGACACATCCTCCAGCACAGCTACATGCAGCTCAGCAGCCTGACATCTGAGGACTCTGCGGTCTATTACTGTGCAAGAATCTACTATGGTAACTACTGGTACTTCGATGTCTGG
CACACTGACTGTAGACACATCCTCCAGCAGCCTACATGCAGCTCAGCAGCCTGACATCTGAGGACTCTGCGGTCTATTACTGTGCAAGAATCTACTATGGTAACTACTGGTACTTCGATGTCTGG
CCCTGACGTTCGACACATCCTCCAGCACAGCCTACATGCAGCTCAGCAGCCTGACATCTGAGGACTCTGCGGTCTATTACTGTGCAAGAATCTACTATGGTAACTACTGGTACTTCGATGTCTGG
AACTTAATTTAGACACATCCTCCAGCACAGCCTACATGCAGCTCAGCAGCCTGACATCTGAGGACTCTGCGGTCTATTACTGTGCAAGAATCTACTATGGTAACTACTGGTACTTCGATGTCTGG
ACCTTCCTGTAGACACATCCTCCAGCACAGCCTACATGCAGCTCAGCAGCCTGACATCTGAGGACTCTGCGGTCTATTACTGTGCAAGAATCTACTATGGTAACTACTGGTACTTCGATGTCTGG
CTAGTACTGTAGACACATCCTCCAGCACAGCCTACATGCAGCTCAGCAGCCTGACATCTGAGGACTCTGCGGTCTATTACTGTGCAAGAATCTACTATGGTAACTACTGGTACTTCGATGTCTGG
CCCGGCCGGTAGACACATCCTCCAGCACAGCCTACATGCAGCTCAGCAGCCTGACATCTGAGGACTCTGCGGTCTATTACTGTGCAAGAATCTACTATGGTAACTACTGGTACTTCGATGTCTGG
CACAGAAGCTAGACACATCCTCCAGCACAGCCTACATGCAGCTCAGCAGCCTGACATCTGAGGACTCTGCGGTCTATTACTGTGCAAGAATCTACTATGGTAACTACTGGTACTTCGATGTCTGG
CTCTGTCTTGAGACACATCCTCCAGCACAGCCTACATGCAGCTCAGCAGCCTGACATCTGAGGACTCTGCGGTCTATTACTGTGCAAGAATCTACTATGGTAACTACTGGTACTTCGATGTCTGG
CAGTGCCGGGAGACACATCCTCCAGCACAGCCTACATGCAGCTCAGCAGCCTGACATCTGAGGACTCTGCGGTCTATTACTGTGCAAGAATCTACTATGGTAACTACTGGTACTTCGATGTCTGG
AACTGCATGAAGACACATCCTCCAGCACAGCCTACATGCAGCTCAGCAGCCTGACATCTGAGGACTCTGCGGTCTATTACTGTGCAAGAATCTACTATGGTAACTACTGGTACTTCGATGTCTGG
CCCTGCCAGAAGACACATCCTCCAGCACAGCCTACATGCAGCTCAGCAGCCTGACATCTGAGGACTCTGCGGTCTATTACTGTGCAAGAATCTACTATGGTAACTACTGGTACTTCGATGTCTGG
CTATGACTGCAGACACATCCTCCAACACAGCCTACCTGCAGCTCAGCAGCCTGACATCTGAGGACACTGCCGTCTATTACTGTGCAAGAATCTACTATGGTAACTACTGGTACTTCGATGTCTGG
GGCGGTTCACCATCTCCAGAGATAATTCTCAAAACATCCTCTATCTTCAAATGAACACCCTGAGAGCTGAGGCCAGTGCCACTTATTACTGTGCAAACGGCCGTCTTAGGTACTTCGATGTCTGG
TTAATGGGAGGTTCACCATCTCAAGAGATGATTCCAAAAGTAGTGTCTACCTGCAAATGAACAGCTTAAGAGCTGAAGATACTGGCATTTATTACTGTCGAACGGGTGAAGACTTTGCTTACTGG
CAATGTATGTAGACACATCCTCCAGCACAGCCTACATGCAGCTCAGCAGCCTGACATCTGAGGACTCTGCGGTCTATTACTGTGCAAGAATCTACTATTGTAACTACTGGTACTTCGATGTCTGG
CCACACTGACTGTAGACACATCCTCCAGCACAGCCTACATGCAGCTCAGCAGCCTGACATCTGAGGACTCTGCGGTCTATTACTGTGCAAGAATCTATGGTAACTACTGGTACTTCGATGTCTGG
CACTGACTGTAGACAAATCCTCCAGCACAGCCTACATGCAGCTCAGCAGCCTGACATCTGAAGACTCTGCGGTCTATTTCTGTGCAAGAATCTACTATGGTAACTACTGGTACTTCGATGTCTGG
ACACTGACTGTAGACACATCCTCCAGCACAGCCTACATGCAGCTAGCAGCCTGACATCTGAGGACTCTGCGGTCTATTACTGTGCAAGAATCTACTATGGTAACTACTGGTACTTCGATGTCTGG
CACACTGACTGTAGACACATCCTCCAGCACAGCCTATGCAGCTCAGCAGCCTGACATCTGAGGACTCTGCGGTCTATTACTGTGCAAGAATCTACTATGGTAACTACTGGTACTTCGATGTCTGG
CACACTGACTGTAGACACATCCTAGCACAGCCTACATGCAGCTCAGCAGCCTGACATCTGAGGACTCTGCGGTCTATTACTGTGCAAGAATCTACTATGGTAACTACTGGTACTTCGATGTCTGG
CAAGGACTTTATCCACATCCTCCAGCACAGCCTACATGCAGCTCAGCAGCCTGACATCTGAGGACTCTGCGGTCTATTACTGTGCAAGAATCTACTATGGTAACTACTGGTACTTCGATGTCTGG
CACTGAATTTCTACACATCCTCCAGCACAGCCTACATGCAGCTCAGCAGCCTGACATCTGAGGACTCTGCGGTCTATTACTGTGCAAGAATCTACTATGGTAACTACTGGTACTTCGATGTCTGG
CACTTAATTTATACACATCCTCCAGCACAGCCTACATGCAGCTCAGCAGCCTGACATCTGAGGACTCTGCGGTCTATTACTGTGCAAGAATCTACTATGGTAACTACTGGTACTTCGATGTCTGG
CACAGAATTTATACACATCCTCCAGCACAGCCTACATGCAGCTCAGCAGCCTGACATCTGAGGACTCTGCGGTCTATTACTGTGCAAGAATCTACTATGGTAACTACTGGTACTTCGATGTCTGG
CACGGACGTTATACACATCCTCCAGCACAGCCTACATGCAGCTCAGCAGCCTGACATCTGAGGACTCTGCGGTCTATTACTGTGCAAGAATCTACTATGGTAACTACTGGTACTTCGATGTCTGG
CACTTAGTGGATACACATCCTCCAGCACAGCCTACATGCAGCTCAGCAGCCTGACATCTGAGGACTCTGCGGTCTATTACTGTGCAAGAATCTACTATGGTAACTACTGGTACTTCGATGTCTGG
CCCTGACGTTTGACACATCCTCCAGCACAGCCTACATGCAGCTCAGCAGCCTGACATCTGAGGACTCTGCGGTCTATTACTGTGCAAGAATCTACTATGGTAACTACTGGTACTTCGATGTCTGG
CACGGCCGGTCGACACATCCTCCAGCACAGCCTACATGCAGCTCAGCAGCCTGACATCTGAGGACTCTGCGGTCTATTACTGTGCAAGAATCTACTATGGTAACTACTGGTACTTCGATGTCTGG
CACAGCAGGTCGACACATCCTCCAGCACAGCCTACATGCAGCTCAGCAGCCTGACATCTGAGGACTCTGCGGTCTATTACTGTGCAAGAATCTACTATGGTAACTACTGGTACTTCGATGTCTGG
GAGTGTCTTTAGACACATCCTCCAGCACAGCCTACATGCAGCTCAGCAGCCTGACATCTGAGGACTCTGCGGTCTATTACTGTGCAAGAATCTACTATGGTAACTACTGGTACTTCGATGTCTGG
CACTGCTGTTAGACACATCCTCCAGCACAGCCTACATGCAGCTCAGCAGCCTGACATCTGAGGACTCTGCGGTCTATTACTGTGCAAGAATCTACTATGGTAACTACTGGTACTTCGATGTCTGG
AACTGTCGTTAGACACATCCTCCAGCACAGCCTACATGCAGCTCAGCAGCCTGACATCTGAGGACTCTGCGGTCTATTACTGTGCAAGAATCTACTATGGTAACTACTGGTACTTCGATGTCTGG
CAATTACGTTAGACACATCCTCCAGCACAGCCTACATGCAGCTCAGCAGCCTGACATCTGAGGACTCTGCGGTCTATTACTGTGCAAGAATCTACTATGGTAACTACTGGTACTTCGATGTCTGG
CAAGGACGTTAGACACATCCTCCAGCACAGCCTACATGCAGCTCAGCAGCCTGACATCTGAGGACTCTGCGGTCTATTACTGTGCAAGAATCTACTATGGTAACTACTGGTACTTCGATGTCTGG
CTAGGTCTGTAGACACATCCTCCAGCACAGCCTACATGCAGCTCAGCAGCCTGACATCTGAGGACTCTGCGGTCTATTACTGTGCAAGAATCTACTATGGTAACTACTGGTACTTCGATGTCTGG
CCATTCCTGTAGACACATCCTCCAGCACAGCCTACATGCAGCTCAGCAGCCTGACATCTGAGGACTCTGCGGTCTATTACTGTGCAAGAATCTACTATGGTAACTACTGGTACTTCGATGTCTGG
ACCGTCCTGTAGACACATCCTCCAGCACAGCCTACATGCAGCTCAGCAGCCTGACATCTGAGGACTCTGCGGTCTATTACTGTGCAAGAATCTACTATGGTAACTACTGGTACTTCGATGTCTGG
ACGAGACTGTAGACACATCCTCCAGCACAGCCTACATGCAGCTCAGCAGCCTGACATCTGAGGACTCTGCGGTCTATTACTGTGCAAGAATCTACTATGGTAACTACTGGTACTTCGATGTCTGG
CAAGTCATGTAGACACATCCTCCAGCACAGCCTACATGCAGCTCAGCAGCCTGACATCTGAGGACTCTGCGGTCTATTACTGTGCAAGAATCTACTATGGTAACTACTGGTACTTCGATGTCTGG
ACCTGCATGTAGACACATCCTCCAGCACAGCCTACATGCAGCTCAGCAGCCTGACATCTGAGGACTCTGCGGTCTATTACTGTGCAAGAATCTACTATGGTAACTACTGGTACTTCGATGTCTGG
CCATTAATGTAGACACATCCTCCAGCACAGCCTACATGCAGCTCAGCAGCCTGACATCTGAGGACTCTGCGGTCTATTACTGTGCAAGAATCTACTATGGTAACTACTGGTACTTCGATGTCTGG
ACATTAATGTAGACACATCCTCCAGCACAGCCTACATGCAGCTCAGCAGCCTGACATCTGAGGACTCTGCGGTCTATTACTGTGCAAGAATCTACTATGGTAACTACTGGTACTTCGATGTCTGG
AAATTAATGTAGACACATCCTCCAGCACAGCCTACATGCAGCTCAGCAGCCTGACATCTGAGGACTCTGCGGTCTATTACTGTGCAAGAATCTACTATGGTAACTACTGGTACTTCGATGTCTGG
CCGTGCCGGTAGACACATCCTCCAGCACAGCCTACATGCAGCTCAGCAGCCTGACATCTGAGGACTCTGCGGTCTATTACTGTGCAAGAATCTACTATGGTAACTACTGGTACTTCGATGTCTGG
CCAGGCCGGTAGACACATCCTCCAGCACAGCCTACATGCAGCTCAGCAGCCTGACATCTGAGGACTCTGCGGTCTATTACTGTGCAAGAATCTACTATGGTAACTACTGGTACTTCGATGTCTGG
CCCGTACGGTAGACACATCCTCCAGCACAGCCTACATGCAGCTCAGCAGCCTGACATCTGAGGACTCTGCGGTCTATTACTGTGCAAGAATCTACTATGGTAACTACTGGTACTTCGATGTCTGG
CCATGTCCGTAGACACATCCTCCAGCACAGCCTACATGCAGCTCAGCAGCCTGACATCTGAGGACTCTGCGGTCTATTACTGTGCAAGAATCTACTATGGTAACTACTGGTACTTCGATGTCTGG
CCATGACGCTAGACACATCCTCCAGCACAGCCTACATGCAGCTCAGCAGCCTGACATCTGAGGACTCTGCGGTCTATTACTGTGCAAGAATCTACTATGGTAACTACTGGTACTTCGATGTCTGG
CACTTAAGCTAGACACATCCTCCAGCACAGCCTACATGCAGCTCAGCAGCCTGACATCTGAGGACTCTGCGGTCTATTACTGTGCAAGAATCTACTATGGTAACTACTGGTACTTCGATGTCTGG
CACGTACTTGAGACACATCCTCCAGCACAGCCTACATGCAGCTCAGCAGCCTGACATCTGAGGACTCTGCGGTCTATTACTGTGCAAGAATCTACTATGGTAACTACTGGTACTTCGATGTCTGG
CACTGAGATGAGACACATCCTCCAGCACAGCCTACATGCAGCTCAGCAGCCTGACATCTGAGGACTCTGCGGTCTATTACTGTGCAAGAATCTACTATGGTAACTACTGGTACTTCGATGTCTGG
CACAGTCGGGAGACACATCCTCCAGCACAGCCTACATGCAGCTCAGCAGCCTGACATCTGAGGACTCTGCGGTCTATTACTGTGCAAGAATCTACTATGGTAACTACTGGTACTTCGATGTCTGG
CCCTGAAGGAAGACACATCCTCCAGCACAGCCTACATGCAGCTCAGCAGCCTGACATCTGAGGACTCTGCGGTCTATTACTGTGCAAGAATCTACTATGGTAACTACTGGTACTTCGATGTCTGG
CACTGACGGACCACACATCCTCCAGCACAGCCTACATGCAGCTCAGCAGCCTGACATCTGAGGACTCTGCGGTCTATTACTGTGCAAGAATCTACTATGGTAACTACTGGTACTTCGATGTCTGG
CACGGACTTGACACACATCCTCCAGCACAGCCTACATGCAGCTCAGCAGCCTGACATCTGAGGACTCTGCGGTCTATTACTGTGCAAGAATCTACTATGGTAACTACTGGTACTTCGATGTCTGG
CACAGACAGAACACACATCCTCCAGCACAGCCTACATGCAGCTCAGCAGCCTGACATCTGAGGACTCTGCGGTCTATTACTGTGCAAGAATCTACTATGGTAACTACTGGTACTTCGATGTCTGG
CACTTCCTTTAGACAAATCCTCCAGCACAGCCTACATGCAGCTCAGCAGCCTGACATCTGAGGACTCTGCGGTCTATTACTGTGCAAGAATCTACTATGGTAACTACTGGTACTTCGATGTCTGG
AACTGAATTTAGACAAATCCTCCAGCACAGCCTACATGCAGCTCAGCAGCCTGACATCTGAGGACTCTGCGGTCTATTACTGTGCAAGAATCTACTATGGTAACTACTGGTACTTCGATGTCTGG
CACTTTATGTAGACAAATCCTCCAGCACAGCCTACATGCAGCTCAGCAGCCTGACATCTGAGGACTCTGCGGTCTATTACTGTGCAAGAATCTACTATGGTAACTACTGGTACTTCGATGTCTGG
CAATGTATGTAGACAAATCCTCCAGCACAGCCTACATGCAGCTCAGCAGCCTGACATCTGAGGACTCTGCGGTCTATTACTGTGCAAGAATCTACTATGGTAACTACTGGTACTTCGATGTCTGG
CACTAACAGAACACAAATCCTCCAGCACAGCCTACATGCAGCTCAGCAGCCTGACATCTGAGGACTCTGCGGTCTATTACTGTGCAAGAATCTACTATGGTAACTACTGGTACTTCGATGTCTGG
ACACTGACTGTAGACACACCTCCAGCACAGCCTACATGCAGCTCAGCAGCCTGACATCTGAGGACTCTGCGGTCTATTACTGTGCAAGAATCTACTATGGTAACTACTGGTACTTCGATGTCTGG
CCACACTGACTGTAGACACATCCTCCACAGCCTACATGCAGCTCAGCAGCCTGACATCTGAGGACTCTGCGGTCTATTACTGTGCAAGAATCTACTATGGTAACTACTGGTACTTCGATGTCTGG
CATTGACTGTAGACAAGTCCTCCAGCACAGCCTACATGGAGCTCCGCAGCCTGACATCTGAGGACTCTGCAGTCTATTACTGTGCAAGAATCTACTATGGTAACTACTGGTACTTCGATGTCTGG
CAAGGCCTGTAGACACATCCTCCAGCACAGCCTACATGCAGCTCAGCAGCCTGACATCTGAGGACTCTGCGGTCTATTACTGTGCAAGAATCTACTATGGTAACTACTGGTACTTCGATGGCTGG

sample_name	productive_frequency	templates	amino_acid
TCL_C25_1	95.075	55015	CARLYYGSSYWYFDVW
TCL_C25_1	4.647	2689	CARSDYYGSSSLDYW
TCL_C25_1	0.079	46	CMRYGNYWYFDVW
TCL_C25_1	0.033	19	CARRYYGSSYWYFDVW
TCL_C25_1	0.007	4	CARLYYGSSYWYFDVW
TCL_C25_1	0.007	4	CARSDYYGSSSLDYW
TCL_C25_1	0.007	4	LCKTAQAAYW
TCL_C25_1	0.005	3	CARNDGYYWYFDVW
TCL_C25_1	0.003	2	CARHGSSYYFDYW
TCL_C25_1	0.003	2	CARLYYGSSYWYFDVW
TCL_C25_1	0.003	2	CARLYYGSSYWYFDVW
TCL_C25_1	0.003	2	LCKTAQAAYW
TCL_C25_1	0.003	2	CARLYYGSSYWYFDVW
TCL_C25_1	0.003	2	CARLYYGSSYWYFDVW
TCL_C25_1	0.003	2	CARLYYGSSYWYFDVW
TCL_C25_1	0.003	2	ARLYYGSSYWYFDVW
TCL_C25_1	0.003	2	CARLYYGSSYWYFDVW
TCL_C25_1	0.003	2	CARSDYYGSSYWYFDVW
TCL_C25_1	0.003	2	CKTAQAAYW
TCL_C25_1	0.003	2	CARLYYGSSYWYFDVW
TCL_C25_1	0.003	2	CARLYYGSSSLDYW
TCL_C25_1	0.003	2	CASFPESEDFAYW
TCL_C25_1	0.002	1	CARSDYGSSYYFDYW
TCL_C25_1	0.002	1	CARLYYGSSYWYFDVW
TCL_C25_1	0.002	1	CARLYYGSSYWYFDVW
TCL_C25_1	0.002	1	CARLYYGSSYWYFDVW
TCL_C25_1	0.002	1	CARLYYGSSYWYFDVW
TCL_C25_1	0.002	1	CARLYYGSSYWYFDVW
TCL_C25_1	0.002	1	CARLYYGSSYWYFDVW
TCL_C25_1	0.002	1	CAWPRLRYFDVW
TCL_C25_1	0.002	1	CARLYYGSSYWYFDVW
TCL_C25_1	0.002	1	CARLYYGSSYWYFDVW
TCL_C25_1	0.002	1	CARLYYGSSYWYFDVW
TCL_C25_1	0.002	1	CARLYYGSSYWYFDVW
TCL_C25_1	0.002	1	CARLYYGSSYWYFDVW
TCL_C25_1	0.002	1	CARLYYGSSYWYFDVW
TCL_C25_1	0.002	1	CARLYYGSSYWYFDVW
TCL_C25_1	0.002	1	CARLYYGSSYWYFDVW
TCL_C25_1	0.002	1	CARLYYGSSYWYFDVW
TCL_C25_1	0.002	1	CARLYYGSSYWYFDVW
TCL_C25_1	0.002	1	CARLYYGSSYWYFDVW
TCL_C25_1	0.002	1	CARLYYGSSYWYFDVW
TCL_C25_1	0.002	1	CARLYYGSSYWYFDVW
TCL_C25_1	0.002	1	CARLYYGSSYWYFDVW
TCL_C25_1	0.002	1	CARLYYGSSYWYFDVW
TCL_C25_1	0.002	1	CARLYYGSSYWYFDVW
TCL_C25_1	0.002	1	CARLYYGSSYWYFDVW
TCL_C25_1	0.002	1	CARPYYGSSYWYFDVW
TCL_C25_1	0.002	1	CKTAQAAYW
TCL_C25_1	0.002	1	CKTAQAAYW
TCL_C25_1	0.002	1	CKTAQAAYW
TCL_C25_1	0.002	1	CARQQAAYW
TCL_C25_1	0.002	1	CARHGSSYYFDYW
TCL_C25_1	0.002	1	CARFYYGSSYAMDYW
TCL_C25_1	0.002	1	CDVNGSRLRYFDVW
TCL_C25_1	0.002	1	CARLYYGSSYWYFDVW
TCL_C25_1	0.002	1	CARDYYGSRRDYW
TCL_C25_1	0.002	1	RCEDFAYW
TCL_C25_1	0.002	1	CARLYYGSSYWYFDVW
TCL_C25_1	0.002	1	CARLYYGSSYWYFDVW
TCL_C25_1	0.002	1	CARSDYYGSSSLDYW
TCL_C25_1	0.002	1	CARLYYGSSYWYFDVW
TCL_C25_1	0.002	1	CARLYYGSSYWYFDVW
TCL_C25_1	0.002	1	CARLYYGSSYWYFDVW
TCL_C25_1	0.002	1	CARLYYGSSYWYFDVW
TCL_C25_1	0.002	1	CARLYYGSSLWYFVVW
TCL_C25_1	0.002	1	CARLYYGSSYWYFDVW
TCL_C25_1	0.002	1	CARLYYGSSYWYFDVW
TCL_C25_1	0.002	1	CARLYYGSSYWYFDVW
TCL_C25_1	0.002	1	CARLYYGSSYWYFDVW
TCL_C25_1	0.002	1	CARLYYGSSYWYFDVW
TCL_C25_1	0.002	1	CARLYYGSSYWYFDVW
TCL_C25_1	0.002	1	CKTAQAAYW
TCL_C25_1	0.002	1	CKTAQAAYW
TCL_C25_1	0.002	1	CARSDYYGSSSLDYW
total	100.000	57865	

rearrangement
TGACTGTAGACACATCCTCCAGCACAGCCTACATGCAGCTCAGCAGCCTGACATCTGAGGACTCTGCGGTCTATTACTGTGCCCGCCTTTACTACGGTAGTAGCTACTGGTACTTCGATGTCTGG
CATTGACTGTAGACAAGTCCTCCAGCACAGCCTACATGGAGCTCCGCAGCCTGACATCTGAGGACTCTGCAGTCTATTACTGTGCAAGATCAGATTACTACGGTAGTAGCTCCCTTGACTACTGG
GATTCACTATCTTCAGAGACAATGACAAGAGCACCCTGTACCTGCAGATGAGCAATGTGCGATCGGAGGACACAGCCACGTATTTCTGTATGAGATATGGTAACTACTGGTACTTCGATGTCTGG
TGACTGTAGACACATCCTCCAGCACAGCCTACATGCAGCTCAGCAGCCTGACATCTGAGGACTCTGCGGTCTATTACTGTGCAAGAAGGTACTACGGTAGTAGCTACTGGTACTTCGATGTCTGG
CTGACTGTAGACACATCCTCAGCACAGCCTACATGCAGCTCAGCAGCCTGACATCTGAGGACTCTGCGGTCTATTACTGTGCCCGCCTTTACTACGGTAGTAGCTACTGGTACTTCGATGTCTGG
CACTGACTGTAGACACATCCTCCAGCACAGCCTACATGCAGCTCAGCAGCCTGACATCTGAGGACTCTGCGGTCTATTACTGTGCAAGATCAGATTACTACGGTAGTAGCTCCCTTGACTACTGG
TGGAGAGACGATTCATCATCTCCAGAGACAATACCAAGAAGACCCTGTACCTGCAAATGAGCAGTCTTGAGGTCTGAGGACACAGCCTTGTATTACTGTGCAAGACAGCTCAGGCTGCTTACTGG
CCACATTGACTGTAGACAAATCCTCCAGCACAGCCTACATGCAGCTCAGCAGCCTGACATCTGAGGACTCTGCGGTCTATTACTGTGCAAGAAATGATGGTTACTACTGGTACTTCGATGTCTGG
GATTCACCATCTCCAGAGACAATGCCAAGAACACCCTGTACCTGCAAATGAGCCGTCTGAAGTCTGAGGACACAGCCATGTATTACTGTGCAAGACACGGTAGTAGCTACTACTTTGACTACTGG
TGACTGTAGACAAGTCCTCCAGCACAGCCTACATGGAGCTCCGCAGCCTGACATCTGAGGACTCTGCAGTCTATTACTGTGCCCGCCTTTACTACGGTAGTAGCTACTGGTACTTCGATGTCTGG
CTGACTGTAGACACATCCCCAGCACAGCCTACATGCAGCTCAGCAGCCTGACATCTGAGGACTCTGCGGTCTATTACTGTGCCCGCCTTTACTACGGTAGTAGCTACTGGTACTTCGATGTCTGG
TGGAGAGACGATTCATCATCTCCAGAGACAATACCAAGAAGACCCTGTACCTGCAAAATGAGCAGTCTGAGGTCTGAGGACACAGCCTTGTATTACTGTGCAAGACAGCTCAGGCTGCTTACTGG
CTGACTGTGACACATCCTCCAGCACAGCCTACATGCAGCTCAGCAGCCTGACATCTGAGGACTCTGCGGTCTATTACTGTGCCCGCCTTTACTACGGTAGTAGCTACTGGTACTTCGATGTCTGG
CTGACTGTAGACACATCTCCAGCACAGCCTACATGCAGCTCAGCAGCCTGACATCTGAGGACTCTGCGGTCTATTACTGTGCCCGCCTTTACTACGGTAGTAGCTACTGGTACTTCGATGTCTGG
TGACTGCAGTCACATCCGCCAGCACTGCCTACATGGAGCTCAGCAGCCTGACAAATGAGGACTCTGCGGTCTATTACTGTGCCCGCCTTTACTACGGTAGTAGCTACTGGTACTTCGATGTCTGG
CACTGACTGTAGACACATCCTCCAGCACAGCCTACATGCAGCTCAGCCTGACATCTGAGGACTCTGCGGTCTATTACTGTGCCCGCCTTTACTACGGTAGTAGCTACTGGTACTTCGATGTCTGG
TGACTGCAAACACATCCTCGAGCACAGCCTACATGGAACTCAGCAGCCTCACATCTGAGGACTCTGCGGTCTATTACTGTGCCCGCCTTTACTACGGTAGTAGCTACTGGTACTTCGATGTCTGG
CTGTAGACAAGTCCTCCAGCACAGCCTACATGGAGCTCCGCAGCCTGACATCTGAGGACTCTGCAGTCTATTACTGTGCAAGATCAGATTACTACGGTAGTAGCTACTGGTACTTCGATGTCTGG
CCATGGAGAGACGATTCATCATCTCCAGAGACAATACCAAGAAGACCCTGTACCTGCAAATGAGCAGTCTGAGGTCTGGACACAGCCTTGTATTACTGTGCAAGACAGCTCAGGCTGCTTACTGG
TGACTTCAGACAAATCCTCCAGCACAGCCTACATGGAGCTCAGCAGCCTGACCTCTGAGGACTCTGCGGTCTATTACTGTGCCCGCCTTTACTACGGTAGTAGCTACTGGTACTTCGATGTCTGG
CCACACTGACTGTAGACACATCCTCCAGCACAGCCTACATGCAGCTCAGCAGCCTGACATCTGAGGACTCTGCGGTCTATTACTGTGCCCGCCTTTACTACGGTAGTAGCTCCCTTGACTACTGG
GACTGAGCATCAGCAAGGACAACTCCAAGAGCCAAGTTTTCTTAAAAATGAACAGTCTGCAAACTGATGACACAGCCATGTACTACTGTGCCAGCTTTCCTGAAAGTGAAGACTTTGCTTACTGG
CATTGACTGTAGACAAATCCTCCAGCACAGCCTACATGCAGCTCAGCAGCCTGACATCTGAGGACTCTGCGGTCTATTACTGTGCAAGATCTGACTACGGTAGTAGCTACTACTTTGACTACTGG
GTCCTGTAGACACATCCTCCAGCACAGCCTACATGCAGCTCAGCAGCCTGACATCTGAGGACTCTGCGGTCTATTACTGTGCCCGCCTTTACTACGGTAGTAGCTACTGGTACTTCGATGTCTGG
TAACTGTAGACAAGTCATCCAGCACAGCCTACATGCAGCTCAAGAGCCTGACATCTGAGGACTCTGCTGTCTATTACTGTGCCCGCCTTTACTACGGTAGTAGCTACTGGTACTTCGATGTCTGG
CTGACTGTAGACACATCCTCCAGCACAGCTACATGCAGCTCAGCAGCCTGACATCTGAGGACTCTGCGGTCTATTACTGTGCCCGCCTTTACTACGGTAGTAGCTACTGGTACTTCGATGTCTGG
TGTCTTGAGACACATCCTCCAGCACAGCCTACATGCAGCTCAGCAGCCTGACATCTGAGGACTCTGCGGTCTATTACTGTGCCCGCCTTTACTACGGTAGTAGCTACTGGTACTTCGATGTCTGG
TGACTGCAGACAAATCCTCCAGCACAGCCTACATGCAACTGAGCAGCCTGACATCTGAGGACTCTGCAGTCTATTACTGTGCCCGCCTTTACTACGGTAGTAGCTACTGGTACTTCGATGTCTGG
CTGACTGAGACACATCCTCCAGCACAGCCTACATGCAGCTCAGCAGCCTGACATCTGAGGACTCTGCGGTCTATTACTGTGCCCGCCTTTACTACGGTAGTAGCTACTGGTACTTCGATGTCTGG
GCAGGCTCGCAGTCTCCAAAGACACCTCCAACAACCAAGCATTCCTGAATATCATCACTGTGGAAACTGCAGATACTGCCATATACTACTGTGCTTGGCCCCGTCTTAGGTACTTCGATGTCTGG
CTGACTGTAGACACATCCTCCAGCACAGCCTACATGAGCTCAGCAGCCTGACATCTGAGGACTCTGCGGTCTATTACTGTGCCCGCCTTTACTACGGTAGTAGCTACTGGTACTTCGATGTCTGG
CTGACTGTAGACACATCCTCCAGACAGCCTACATGCAGCTCAGCAGCCTGACATCTGAGGACTCTGCGGTCTATTACTGTGCCCGCCTTTACTACGGTAGTAGCTACTGGTACTTCGATGTCTGG
TGTCTTTATACACATCCTCCAGCACAGCCTACATGCAGCTCAGCAGCCTGACATCTGAGGACTCTGCGGTCTATTACTGTGCCCGCCTTTACTACGGTAGTAGCTACTGGTACTTCGATGTCTGG
TGAGATTAGACACATCCTCCAGCACAGCCTACATGCAGCTCAGCAGCCTGACATCTGAGGACTCTGCGGTCTATTACTGTGCCCGCCTTTACTACGGTAGTAGCTACTGGTACTTCGATGTCTGG
TGACAGAACACACATCCTCCAGCACAGCCTACATGCAGCTCAGCAGCCTGACATCTGAGGACTCTGCGGTCTATTACTGTGCCCGCCTTTACTACGGTAGTAGCTACTGGTACTTCGATGTCTGG
CTGACTGTAGACACATCCTCCAGCACAGCCACATGCAGCTCAGCAGCCTGACATCTGAGGACTCTGCGGTCTATTACTGTGCCCGCCTTTACTACGGTAGTAGCTACTGGTACTTCGATGTCTGG
TGACTGTAGACAAGTCCTCTAGCACAGCCTACATGGAGCTCCGCAGCCTGACATCTGAGGACTCTGCAGTCTATTACTGTGCCCGCCTTTACTACGGTAGTAGCTACTGGTACTTCGATGTCTGG
TGACTGTAGACAAATCCTCCAGCACAGCCTACATGCAGCTCAACAGCCTGACATCTGAGGACTCTGCAGTCTATTACTGTGCCCGCCTTTACTACGGTAGTAGCTACTGGTACTTCGATGTCTGG
TGACTGTAGACAAGTCCTCCAGCACAGCCTACATGGAGCTCCGCAGCCTGACATCTGAGGACACTGCAGTCTATTACTGTGCCCGCCTTTACTACGGTAGTAGCTACTGGTACTTCGATGTCTGG
TGACTGTAGACAAATCCTCCAGCACAGCCTACATGCAGCTCAGCAGCCTGACATCTGAAGACTCTGCGGTCTATTTCTGTGCCCGCCTTTACTACGGTAGTAGCTACTGGTACTTCGATGTCTGG
CTGACTGTAGACACATCCTCCAGCACAGCCTACTGCAGCTCAGCAGCCTGACATCTGAGGACTCTGCGGTCTATTACTGTGCCCGCCTTTACTACGGTAGTAGCTACTGGTACTTCGATGTCTGG
CTGACTGTAGACACATCCTCCAGCACAGCCTCATGCAGCTCAGCAGCCTGACATCTGAGGACTCTGCGGTCTATTACTGTGCCCGCCTTTACTACGGTAGTAGCTACTGGTACTTCGATGTCTGG
ACTGACTGTACACATCCTCCAGCACAGCCTACATGCAGCTCAGCAGCCTGACATCTGAGGACTCTGCGGTCTATTACTGTGCCCGCCTTTACTACGGTAGTAGCTACTGGTACTTCGATGTCTGG
AGCCTGACGACACATCCTCCAGCACAGCCTACATGCAGCTCAGCAGCCTGACATCTGAGGACTCTGCGGTCTATTACTGTGCCCGCCTTTACTACGGTAGTAGCTACTGGTACTTCGATGTCTGG
TAACAGAACACAAATCCTCCAGCACAGCCTACATGCAGCTCAGCAGCCTGACATCTGAGGACTCTGCGGTCTATTACTGTGCCCGCCTTTACTACGGTAGTAGCTACTGGTACTTCGATGTCTGG
CACTGACTGTAGACACATCCAGCACAGCCTACATGCAGCTCAGCAGCCTGACATCTGAGGACTCTGCGGTCTATTACTGTGCCCGCCTTTACTACGGTAGTAGCTACTGGTACTTCGATGTCTGG
TGACTGCAGACAAATCCTCCAGCACAGCCTACATGGAGCTCCGCAGCCTGACATCTGAGGACTCTGCCGTCTATTACTGTGCCCGCCTTTACTACGGTAGTAGCTACTGGTACTTCGATGTCTGG
CTGACTGTAGACACATCCCCAGCACAGCCTACATGCAGCTCAGCAGCCTGACATCTGAGGACTCTGCGGTCTATTACTGTGCCCGCCCTTACTACGGTAGTAGCTACTGGTACTTCGATGTCTGG
CCATGGAGAGACGATTCATCATCTCCAGAGACAATACCAAGAAGACCCTGTACCTGCAAATGAGCAGTCTGAGGTCTGAGGACACAGCCTTATTACTGTGCAAGACAGCTCAGGCTGCTTACTGG
CCATGGAGAGACGATTCATCATCTCCAGAGACAATACCAAGAAGACCCTGTACCTGCAAATGAGCAGTCTGAGGTCTGAGGACAGCCTTGTATTACTGTGCAAGACAGCTCAGGCTGCTTACTGG
CCATGGAGAGACGATTCATCATCTCCAGAGACAATACCAAGAAGACCCTGTACCTGCAAATGAGCAGTCTGAGGTCTGAGGACACAGCCTTGTATTACTGCAAGACAGCTCAGGCTGCTTACTGG
CCATGGAGAGACGATTCATCATCTCCAGAGACAATACCAAGAAGACCCTGTACCTGCAAATGAGCAGTCTGAGGTCTGAGGACACAGCCTTGTATTACTGTGCAAGACAGCAGGCTGCTTACTGG
GAATCACCATCTCCAGAGACAATGCCAAGAACACCCTGTACCTGCAAATGAGCCGTCTGAAGTCTGAGGACACAGCCATGTATTACTGTGCAAGACACGGTAGTAGCTACTACTTTGACTACTGG
CACTGACTGTAGACACATCCTCCAGCACAGCCTACATGCAGCTCAGCAGCCTGACATCTGAGGACTCTGCGGTCTATTACTGTGCAAGATTTACTACGGTAGTAGCTACGCTATGGACTACTGG
CCACATTGACTGTAGACAAATCCTCCAGCACAGCCTACATGCAGCTCAACAGCCTGACATCTGAGGACTCTGCAGTCTATTACTGTGATGTCAATGGCTCCCGTCTTAGGTACTTCGATGTCTGG
TGACGTGAGACACATCCTCCAGCACAGCCTACATGCAGCTCAGCAGCCTGACATCTGAGGACTCTGCGGTCTATTACTGTGCCCGCCTTTACTACGGTAGTAGCTACTGGTACTTCGATGTCTGG
GAATCTCCATCACTCGTGACACATCTAAGAACCAGTTTTTCCTGAAGTTGAATTCTGTGACTACTGAGGACACAGCCACATATTACTGTGCAAGAGATTACTACGGTAGTCGGAGAGACTACTGG
AAAACTTCAAGGGCAAGGCCACATTGACTGCAAACACATCCTCGAGCACAGCCTACATGGAACTCAGCAGCCTCACATCTGAGGACTCTGCGGTCTATTACAGGTGTGAAGACTTTGCTTACTGG
TGACTGTAGACAATTCCTCCAGCACAGCCTACATGGAGCTCAGCAGCCTGACATCTGAGGACTCTGCAGTCTATTACTGTGCCCGCCTTTACTACGGTAGTAGCTACTGGTACTTCGATGTCTGG
ACTGACTGGACACATCCTCCAGCACAGCCTACATGCAGCTCAGCAGCCTGACATCTGAGGACTCTGCGGTCTATTACTGTGCCCGCCTTTACTACGGTAGTAGCTACTGGTACTTCGATGTCTGG
ACATTGACTGTAGACAAGTCCTCAGCACAGCCTACATGGAGCTCCGCAGCCTGACATCTGAGGACTCTGCAGTCTATTACTGTGCAAGATCAGATTACTACGGTAGTAGCTCCCTTGACTACTGG
ACTGACTGTAGACACATCCTCCAGCAGCCTACATGCAGCTCAGCAGCCTGACATCTGAGGACTCTGCGGTCTATTACTGTGCCCGCCTTTACTACGGTAGTAGCTACTGGTACTTCGATGTCTGG
ACTGACTGTAGACATCCTCCAGCACAGCCTACATGCAGCTCAGCAGCCTGACATCTGAGGACTCTGCGGTCTATTACTGTGCCCGCCTTTACTACGGTAGTAGCTACTGGTACTTCGATGTCTGG
CTGACTGTAGACACATCCTCCAGCACAGCCTACAGCAGCTCAGCAGCCTGACATCTGAGGACTCTGCGGTCTATTACTGTGCCCGCCTTTACTACGGTAGTAGCTACTGGTACTTCGATGTCTGG
TGACTGCAGACACATCCTCCAACACAGCCTACCTGCAGCTCAGCAGCCTGACATCTGAGGACACTGCCGTCTATTACTGTGCCCGCCTTTACTACGGTAGTAGCTACTGGTACTTCGATGTCTGG
TGACTGTAGACACATCCTCCAGCACAGCCTACATGCAGCTCAGCAGCCTGACATCTGAGGACTCTGCGGTCTATTACTGTGCCCGCCTTTACTACGGTAGTAGCTTGTGGTACTTCGTTGTCTGG
TGACTGCAGACACATCCTCCAGCACAGCCTACATGCAGCTCAGCAGCCTGACATCTGAGGACTCTGCAGTCTATTTCTGTGCCCGCCTTTACTACGGTAGTAGCTACTGGTACTTCGATGTCTGG
ACTGACTGTAGACACATCCTCCAGCACAGCCTATGCAGCTCAGCAGCCTGACATCTGAGGACTCTGCGGTCTATTACTGTGCCCGCCTTTACTACGGTAGTAGCTACTGGTACTTCGATGTCTGG
ACTGACTGTAGACACATCCTAGCACAGCCTACATGCAGCTCAGCAGCCTGACATCTGAGGACTCTGCGGTCTATTACTGTGCCCGCCTTTACTACGGTAGTAGCTACTGGTACTTCGATGTCTGG
GGCCGGTCGACACATCCTCCAGCACAGCCTACATGCAGCTCAGCAGCCTGACATCTGAGGACTCTGCGGTCTATTACTGTGCCCGCCTTTACTACGGTAGTAGCTACTGGTACTTCGATGTCTGG
CTGCTGTAGACACATCCTCCAGCACAGCCTACATGCAGCTCAGCAGCCTGACATCTGAGGACTCTGCGGTCTATTACTGTGCCCGCCTTTACTACGGTAGTAGCTACTGGTACTTCGATGTCTGG
TCACAGAAGACACATCCTCCAGCACAGCCTACATGCAGCTCAGCAGCCTGACATCTGAGGACTCTGCGGTCTATTACTGTGCCCGCCTTTACTACGGTAGTAGCTACTGGTACTTCGATGTCTGG
CCATGGAGAGACGATTCATCATCTCCAGAGACAATACCAAGAAGACCCTGTACCTGCAAATGAGCAGTGAGGTCTGAGGACACAGCCTTGTATTACTGTGCAAGACAGCTCAGGCTGCTTACTGG
CCATGGAGAGACGATTCATCATCTCCAGAGACAATACCAAGAAGACCCTGTACCTGCAAATGAGCAGTCAGGTCTGAGGACACAGCCTTGTATTACTGTGCAAGACAGCTCAGGCTGCTTACTGG
CATTGACTGCAGACAAATCCTCCAGCACAGCCTACATGCAACTGAGCAGCCTGACATCTGAGGACTCTGCAGTCTATTACTGTGCAAGATCAGATTACTACGGTAGTAGCTCCCTTGACTACTGG

sample_name	templates	productive_frequency	aaSeqCDR3	clonalSequence
TCL1_347_PC	103590	94.957	CMRYGNYWYFDVW	TGTATGAGATATGGTAACTACTGGTACTTCGATGTCTGG
TCL1_347_PC	5392	4.943	CAGDRWGYWYFDVW	TGTGCAGGAGACAGATGGGGCTACTGGTACTTCGATGTCTGG
TCL1_347_PC	108	0.099	CARLLRYYWYFDVW	TGTGCAAGATTACTACGGTACTACTGGTACTTCGATGTCTGG
TCL1_347_PC	2	0.002	CMRYDSNYWYFDVW	TGTATGAGATATGATAGTAACTACTGGTACTTCGATGTCTGG
total	109092	100		

sample_name	templates	productive_frequency	aaSeqCDR3	clonalSequence
TCL1_347_LIVER	134453	99.817	CMRYGNYWYFDVW	TGTATGAGATATGGTAACTACTGGTACTTCGATGTCTGG
TCL1_347_LIVER	189	0.140	CAGDRWGYWYFDVW	TGTGCAGGAGACAGATGGGGCTACTGGTACTTCGATGTCTGG
TCL1_347_LIVER	58	0.043	CARLLRYYWYFDVW	TGTGCAAGATTACTACGGTACTACTGGTACTTCGATGTCTGG
total	134700	100		

sample_name	templates	productive_frequency	aaSeqCDR3	clonalSequence
TCL1_347_LNP	117243	98.711	CMRYGNYWYFDVW	TGTATGAGATATGGTAACTACTGGTACTTCGATGTCTGG
TCL1_347_LNP	1313	1.105	CVREGDRWYFDVW	TGTGTGAGAGAAGGCGACCGGTGGTACTTCGATGTCTGG
TCL1_347_LNP	48	0.040	CAGDRWGYWYFDVW	TGTGCAGGAGACAGATGGGGCTACTGGTACTTCGATGTCTGG
TCL1_347_LNP	40	0.034	CARDYYGNYGYFDVW	TGTGCAAGAGATTACTATGGTAACTACGGGTACTTCGATGTCTGG
TCL1_347_LNP	32	0.027	CARLYYGSSYWYFDVW	TGTGCAAGACTTTACTACGGTAGTAGCTACTGGTACTTCGATGTCTGG
TCL1_347_LNP	20	0.017	CASHYYYGSSWYFDVW	TGTGCTAGCCATTATTACTACGGTAGTAGCTGGTACTTCGATGTCTGG
TCL1_347_LNP	18	0.015	CARERDDYDGTRYFDVW	TGTGCAAGAGAGAGGGATGATTACGACGGGACAAGGTACTTCGATGTCTGG
TCL1_347_LNP	18	0.015	CMRNGYYYWYFDVW	TGTATGAGAAATGGTTACTACTACTGGTACTTCGATGTCTGG
TCL1_347_LNP	13	0.011	CARLLRYYWYFDVW	TGTGCAAGATTACTACGGTACTACTGGTACTTCGATGTCTGG
TCL1_347_LNP	8	0.007	CARLSYDYDGYYAMDYW	TGTGCAAGATTGTCCTATGATTACGACGGCTACTATGCTATGGACTACTGG
TCL1_347_LNP	7	0.006	CTRNGYDGFDYW	TGTACCAGGAATGGTTACGACGGCTTTGACTACTGG
TCL1_347_LNP	3	0.003	CVYYYGSSYWYFDVW	TGTGTATATTACTACGGTAGTAGCTACTGGTACTTCGATGTCTGG
TCL1_347_LNP	3	0.003	CMRYGSSYWYFDVW	TGTATGAGATATGGTAGTAGCTACTGGTACTTCGATGTCTGG
TCL1_347_LNP	2	0.002	CARLLRYYWYFDVW	TGTGCGAGATTACTACGGTACTACTGGTACTTCGATGTCTGG
TCL1_347_LNP	2	0.002	CATGTETWFAYW	TGTGCAACTGGGACAGAGACCTGGTTTGCTTACTGG
TCL1_347_LNP	1	0.001	CARERDDYDRTRYFDVW	TGTGCAAGAGAGAGGGATGATTACGACAGGACAAGGTACTTCGATGTCTGG
TCL1_347_LNP	1	0.001	CARYGAYDGYYYFDYW	TGTGCAAGATACGGGGCTTATGATGGTTACTACTACTTTGACTACTGG
TCL1_347_LNP	1	0.001	CARSDYYGSSSLDYW	TGTGCAAGATCAGATTACTACGGTAGTAGCTCCCTTGACTACTGG
TCL1_347_LNP	1	0.001	CARPDYPAWFAYW	TGTGCAAGGCCCGATTACCCCGCCTGGTTTGCTTACTGG
total	118774	100		

sample_name	templates	productive_frequency	aaSeqCDR3	clonalSequence
TCL1_F3_LIVER	191330	99.959	CARIYYGNYWYFDVW	TGTGCAAGAATCTACTATGGTAACTACTGGTACTTCGATGTCTGG
TCL1_F3_LIVER	53	0.028	CVYYYGSSYWYFDVW	TGTGTATATTACTACGGTAGTAGCTACTGGTACTTCGATGTCTGG
TCL1_F3_LIVER	8	0.004	CMRYGNYWYFDVW	TGTATGAGATATGGTAACTACTGGTACTTCGATGTCTGG
TCL1_F3_LIVER	6	0.003	CMRYSNYWYFDVW	TGTATGAGATATAGTAACTACTGGTACTTCGATGTCTGG
TCL1_F3_LIVER	4	0.002	CMRYGSSYWYFDVW	TGTATGAGATACGGTAGTAGCTACTGGTACTTCGATGTCTGG
TCL1_F3_LIVER	3	0.002	CVYYYGISYWYFDVW	TGTGTATATTACTACGGTATTAGCTACTGGTACTTCGATGTCTGG
TCL1_F3_LIVER	1	0.001	CARSAIYYDYDYWYFDVW	TGTGCAAGATCGGCCATCTACTATGATTATGACTACTGGTACTTCGATGTCTGG
TCL1_F3_LIVER	1	0.001	CARRYYGSSYWYFDVW	TGTGCAAGAAGATACTACGGTAGTAGCTACTGGTACTTCGATGTCTGG
TCL1_F3_LIVER	1	0.001	CARIYGNYWYFDVW	TGTGCAAGAATCTACGGTAACTACTGGTACTTCGATGTCTGG
TCL1_F3_LIVER	1	0.001	CTKGYDWYFDVW	TGCACAAAGGGTTACGACTGGTACTTCGATGTCTGG
TCL1_F3_LIVER	1	0.001	CAREGETVDYW	TGTGCAAGAGAGGGAGAAACGGTTGACTACTGG
total	191409	100		

sample_name	templates	productive_frequency	aaSeqCDR3	clonalSequence
TCL1_F3_LNP	171007	99.953	CARIYYGNYWYFDVW	TGTGCAAGAATCTACTATGGTAACTACTGGTACTTCGATGTCTGG
TCL1_F3_LNP	46	0.027	CVYYYGSSYWYFDVW	TGTGTATATTACTACGGTAGTAGCTACTGGTACTTCGATGTCTGG
TCL1_F3_LNP	14	0.008	CARDYYGSNWYFDVW	TGTGCAAGAGATTACTACGGTAGTAACTGGTACTTCGATGTCTGG
TCL1_F3_LNP	9	0.005	CMRYGNYWYFDVW	TGTATGAGATATGGTAACTACTGGTACTTCGATGTCTGG
TCL1_F3_LNP	4	0.002	CVYYYGISYWYFDVW	TGTGTATATTACTACGGTATTAGCTACTGGTACTTCGATGTCTGG
TCL1_F3_LNP	3	0.002	CARHGGYWYFDVW	TGTGCAAGACACGGGGGCTACTGGTACTTCGATGTCTGG
TCL1_F3_LNP	2	0.001	CARDYDYKVDYW	TGTGCAAGGGACTATGATTACAAGGTTGACTACTGG
TCL1_F3_LNP	1	0.001	CARRVVARYWYFDVW	TGTGCAAGACGGGTAGTAGCTCGCTACTGGTACTTCGATGTCTGG
TCL1_F3_LNP	1	0.001	CARYDYAFDYW	TGTGCAAGATATGATTACGCCTTTGACTACTGG
total	171087			

sample_name	templates	productive_frequency	aaSeqCDR3	clonalSequence
TCL1_F3_BM	207301	99.961	CARIYYGNYWYFDVW	TGTGCAAGAATCTACTATGGTAACTACTGGTACTTCGATGTCTGG
TCL1_F3_BM	54	0.026	CVYYYGSSYWYFDVW	TGTGTATATTACTACGGTAGTAGCTACTGGTACTTCGATGTCTGG
TCL1_F3_BM	9	0.004	CMRYGNYWYFDVW	TGTATGAGATATGGTAACTACTGGTACTTCGATGTCTGG
TCL1_F3_BM	5	0.002	CARDYYGSNWYFDVW	TGTGCAAGAGATTACTACGGTAGTAACTGGTACTTCGATGTCTGG
TCL1_F3_BM	4	0.002	CATAYW	TGTGCAACTGCTTACTGG
TCL1_F3_BM	3	0.001	CVYYYGISYWYFDVW	TGTGTATATTACTACGGTATTAGCTACTGGTACTTCGATGTCTGG
TCL1_F3_BM	2	0.001	YCARIYYGNYWYFDVW	TACTGTGCAAGAATCTACTATGGTAACTACTGGTACTTCGATGTCTGG
TCL1_F3_BM	1	0.000	CARSGVYGSSLYWYFDVW	TGTGCAAGATCGGGGGTCTACGGTAGTAGCCTTTACTGGTACTTCGATGTCTGG
TCL1_F3_BM	1	0.000	CASHDYYGSSWYFDVW	TGTGCAAGCCACGATTACTACGGTAGTAGCTGGTACTTCGATGTCTGG
TCL1_F3_BM	1	0.000	CARYGYGSSYGYFDVW	TGTGCAAGATACGGTTACGGTAGTAGCTACGGGTACTTCGATGTCTGG
TCL1_F3_BM	1	0.000	CARGGFSNYVRYFDVW	TGTGCAAGAGGGGGATTTAGTAACTACGTGAGGTACTTCGATGTCTGG
total	207382	100		

sample_name	templates	productive_frequency	aaSeqCDR3
TCL1_221_PC	135870	91.369	CMRYSNYWYFDVW
TCL1_221_PC	4092	2.752	CARRYYGSSYWYFDVW
TCL1_221_PC	2223	1.495	CARPGYYGSSYTFDYW
TCL1_221_PC	2005	1.348	CAGDRYGYWYFDVW
TCL1_221_PC	1219	0.820	CARFYYYGSSYAMDYW
TCL1_221_PC	1170	0.787	CMRYGSSYWYFDVW
TCL1_221_PC	976	0.656	CAREGGYSYWYFDVW
TCL1_221_PC	367	0.247	CAGDYDGYWYFDVW
TCL1_221_PC	244	0.164	CARYYGSSYWFAYW
TCL1_221_PC	221	0.149	CARDYSNYWYFDVW
TCL1_221_PC	55	0.037	CTTMVNW
TCL1_221_PC	43	0.029	CARPGYYGSSYWYFDVW
TCL1_221_PC	43	0.029	CARRYYGSSYTFDYW
TCL1_221_PC	28	0.019	CAGDRSGYTGFAYW
TCL1_221_PC	21	0.014	CAGDRLGYWYFDVW
TCL1_221_PC	18	0.012	CARRYYGSSYAMDYW
TCL1_221_PC	17	0.011	CARYYVSSYWFAYW
TCL1_221_PC	12	0.008	CARWGTGHYFDYW
TCL1_221_PC	11	0.007	CARFYYYGSSYWYFDVW
TCL1_221_PC	9	0.006	CARPGYYGSSYAMDYW
TCL1_221_PC	9	0.006	CARYYGSSYWYFDVW
TCL1_221_PC	9	0.006	CARYKRTGTKDYW
TCL1_221_PC	6	0.004	CARFYYYGSSYTFDYW
TCL1_221_PC	5	0.003	CARRYYGSSYWFAYW
TCL1_221_PC	5	0.003	CARRYYGSSFAYW
TCL1_221_PC	4	0.003	CTRDLDGYYWYYYAMDYW
TCL1_221_PC	4	0.003	CARPGYYGSSYWFAYW
TCL1_221_PC	4	0.003	CARFYYYGSSFAYW
TCL1_221_PC	3	0.002	CAGDPYGYWYFDVW
TCL1_221_PC	2	0.001	LTSEDSAVYYCARPGYYGSSYTFDYW
TCL1_221_PC	2	0.001	CARVGLYDYDEGYFDYW
TCL1_221_PC	2	0.001	CARRYYGSSYTFDYR
TCL1_221_PC	2	0.001	CAGDRSGYTGFAYW
TCL1_221_PC	1	0.001	CARGTLITTVVDWYFDVW
TCL1_221_PC	1	0.001	CAGDRYSNYWYFDVW
TCL1_221_PC	1	0.001	CARYYGSSFAYW
TCL1_221_PC	1	0.001	SARCLWYFDYW
total	148705	100	

clonalSequence
TGTATGAGATATAGTAACTACTGGTACTTCGATGTCTGG
TGTGCAAGAAGATACTACGGTAGTAGCTACTGGTACTTCGATGTCTGG
TGTGCAAGACCGGGGTACTACGGTAGTAGCTACACGTTTGACTACTGG
TGTGCAGGAGACAGATATGGTTACTGGTACTTCGATGTCTGG
TGTGCAAGATTTTATTACTACGGTAGTAGCTATGCTATGGACTACTGG
TGTATGAGATACGGTAGTAGCTACTGGTACTTCGATGTCTGG
TGTGCTAGAGAAGGTGGTTACAGCTACTGGTACTTCGATGTCTGG
TGTGCAGGAGACTATGATGGTTACTGGTACTTCGATGTCTGG
TGTGCAAGATACTACGGTAGTAGCTACTGGTTTGCTTACTGG
TGTGCCAGAGACTATAGTAACTACTGGTACTTCGATGTCTGG
TGTACCACTATGGTTAACTGG
TGTGCAAGACCGGGGTACTACGGTAGTAGCTACTGGTACTTCGATGTCTGG
TGTGCAAGAAGATACTACGGTAGTAGCTACACGTTTGACTACTGG
TGTGCAGGAGACAGATCGGGCTATACGGGGTTTGCTTACTGG
TGTGCAGGAGACAGACTGGGCTACTGGTACTTCGATGTCTGG
TGTGCAAGAAGATACTACGGTAGTAGCTATGCTATGGACTACTGG
TGTGCAAGATACTACGTTAGTAGCTACTGGTTTGCTTACTGG
TGTGCAAGATGGGGGACGGGACACTACTTTGACTACTGG
TGTGCAAGATTTTATTACTACGGTAGTAGCTACTGGTACTTCGATGTCTGG
TGTGCAAGACCGGGGTACTACGGTAGTAGCTATGCTATGGACTACTGG
TGTGCAAGATACTACGGTAGTAGCTACTGGTACTTCGATGTCTGG
TGTGCAAGATATAAGCGAACTGGGACGAAGGACTACTGG
TGTGCAAGATTTTATTACTACGGTAGTAGCTACACGTTTGACTACTGG
TGTGCAAGAAGATACTACGGTAGTAGCTACTGGTTTGCTTACTGG
TGTGCAAGAAGATACTACGGTAGTAGCTTTGCTTACTGG
TGTACAAGAGACCTTGATGGTTACTACTGGTATTACTATGCTATGGACTACTGG
TGTGCAAGACCGGGGTACTACGGTAGTAGCTACTGGTTTGCTTACTGG
TGTGCAAGATTTTATTACTACGGTAGTAGCTTTGCTTACTGG
TGTGCAGGAGACCCTTATGGTTACTGGTACTTCGATGTCTGG
CTGACATCTGAGGACTCTGCGGTCTATTACTGTGCAAGACCGGGGTACTACGGTAGTAGCTACACGTTTGACTACTGG
TGTGCAAGAGTAGGGCTCTATGATTACGACGAGGGTTACTTTGACTACTGG
TGTGCAAGAAGATACTACGGTAGTAGCTACACGTTTGACTACAGG
TGTGCTGGAGACAGATCGGGCTATACGGGGTTTGCTTACTGG
TGTGCAAGAGGAACCCTAATTACTACGGTAGTAGACTGGTACTTCGATGTCTGG
TGTGCAGGAGACAGATATAGTAACTACTGGTACTTCGATGTCTGG
TGTGCAAGATACTACGGTAGTAGCTTTGCTTACTGG
TCTGCAAGATGTCTATGGTACTTTGACTACTGG

sample_name	templates	productive_frequency	aaSeqCDR3	clonalSequence
TCL1_221_LNP	113932	95.748	CMRYSNYWYFDVW	TGTATGAGATATAGTAACTACTGGTACTTCGATGTCTGG
TCL1_221_LNP	4540	3.815	CMRYGNYWYFDVW	TGTATGAGATATGGTAACTACTGGTACTTCGATGTCTGG
TCL1_221_LNP	517	0.434	CMRYGSSYWYFDVW	TGTATGAGATATGGTAGTAGCTACTGGTACTTCGATGTCTGG
TCL1_221_LNP	2	0.002	CVRHTVWNLQCEPRHKPP	TGTGTGAGACACACAGTGTGGAATCTTCAGTGTGAGCCTAGACACAAACCTCCA
total	118991	100		

sample_name	templates	productive_frequency	aaSeqCDR3	clonalSequence
TX_Q82_PC	118494	98.428	CMRYGNYWYFDVW	TGTATGAGATATGGTAACTACTGGTACTTCGATGTCTGG
TX_Q82_PC	1754	1.457	CVYYYGSSYWYFDVW	TGTGTATATTACTACGGTAGTAGCTACTGGTACTTCGATGTCTGG
TX_Q82_PC	117	0.097	CARKGNYWYFDVW	TGTGCAAGGAAGGGTAACTACTGGTACTTCGATGTCTGG
TX_Q82_PC	4	0.003	CAGDYDGYWYFDVW	TGTGCAGGAGACTATGATGGTTACTGGTACTTCGATGTCTGG
TX_Q82_PC	4	0.003	CARRHYGSSPLAYW	TGTGCAAGACGGCACTACGGTAGTAGCCCCCTTGCTTACTGG
TX_Q82_PC	3	0.002	CAKIYYYGSSYAMDYW	TGTGCAAAAATTTATTACTACGGTAGTAGCTATGCTATGGACTACTGG
TX_Q82_PC	3	0.002	CARYYYGSSYAMDYW	TGTGCAAGATATTACTACGGTAGTAGCTATGCTATGGACTACTGG
TX_Q82_PC	3	0.002	CARYGLLWYWYFDVW	TGTGCAAGATATGGACTACTATGGTACTGGTACTTCGATGTCTGG
TX_Q82_PC	3	0.002	CAREDGYYFAYW	TGTGCAAGGGAGGATGGTTACTACTTTGCTTACTGG
TX_Q82_PC	2	0.002	CAKRAQATGSFAYW	TGTGCCAAACGGGCTCAGGCTACGGGGTCGTTTGCTTACTGG
total	120387	100		

sample_name	templates	productive_frequency	aaSeqCDR3	clonalSequence
TX_Q82_LIVER	55669	99.778	CMRYGNYWYFDVW	TGTATGAGATATGGTAACTACTGGTACTTCGATGTCTGG
TX_Q82_LIVER	105	0.188	CVYYYGSSYWYFDVW	TGTGTATATTACTACGGTAGTAGCTACTGGTACTTCGATGTCTGG
TX_Q82_LIVER	10	0.018	CVYYYGSSYWYFDVW	TGTGTATATTACTACGGTAGTAGTTACTGGTACTTCGATGTCTGG
TX_Q82_LIVER	9	0.016	CVYYYGISYWYFDVW	TGTGTATATTACTACGGTATTAGCTACTGGTACTTCGATGTCTGG
total	55793	100		

sample_name	templates	productive_frequency	aaSeqCDR3	clonalSequence
TX_Q83_PC	105153	99.816	CMRYGNYWYFDVW	TGTATGAGATATGGTAACTACTGGTACTTCGATGTCTGG
TX_Q83_PC	190	0.180	CVYYYGSSYWYFDVW	TGTGTATATTACTACGGTAGTAGCTACTGGTACTTCGATGTCTGG
TX_Q83_PC	3	0.003	CARGMITRYAMDYW	TGTGCAAGGGGGATGATTACACGGTATGCTATGGACTACTGG
TX_Q83_PC	1	0.001	CAREGGYSYWYFDVW	TGTGCTAGAGAAGGTGGTTACAGCTACTGGTACTTCGATGTCTGG
total	105347	100		

sample_name	templates	productive_frequency	aaSeqCDR3	clonalSequence
TX_702_PC	181594	97.539	CMRYGNYWYFDVW	TGTATGAGATATGGTAACTACTGGTACTTCGATGTCTGG
TX_702_PC	4431	2.380	CAGDRWGYWYFDVW	TGTGCAGGAGACAGATGGGGCTACTGGTACTTCGATGTCTGG
TX_702_PC	58	0.031	CMRYNSNYWYFDVW	TGTATGAGATATAATAGTAACTACTGGTACTTCGATGTCTGG
TX_702_PC	50	0.027	CARRHYYGSYYAMDYW	TGTGCAAGAAGGCATTACTACGGTAGTTACTATGCTATGGACTACTGG
TX_702_PC	23	0.012	CARRDYGSSYWYFDVW	TGTGCAAGAAGGGACTACGGTAGTAGCTACTGGTACTTCGATGTCTGG
TX_702_PC	7	0.004	CARDDGYYDYW	TGTGCAAGAGATGATGGTTACTACGACTACTGG
TX_702_PC	4	0.002	CAREEITTVVDWYFDVW	TGTGCAAGAGAGGAGATTACTACGGTAGTGGACTGGTACTTCGATGTCTGG
TX_702_PC	3	0.002	CAGDITTVVATWSYWYFDVW	TGTGCAGGAGACATTACTACGGTAGTAGCTACGTGGAGCTACTGGTACTTCGATGTCTGG
TX_702_PC	3	0.002	CARLAYGSSQGYAMDYW	TGTGCAAGATTAGCCTACGGTAGTAGCCAGGGGTATGCTATGGACTACTGG
tX_702_PC	2	0.001	CARLYSNYW	TGTGCAAGACTCTATAGTAACTACTGG
TX_702_PC	1	0.001	CTRSGYYVGAWFAYW	TGTACCAGGAGTGGTTACTACGTTGGGGCCTGGTTTGCTTACTGG
total	186176	100		

sample_name	templates	productive_frequency	aaSeqCDR3	clonalSequence
TX_702_LIVER	123451	99.901	CMRYGNYWYFDVW	TGTATGAGATATGGTAACTACTGGTACTTCGATGTCTGG
TX_702_LIVER	49	0.040	CARLYYGSSYWYFDVW	TGTGCCCGCCTTTACTACGGTAGTAGCTACTGGTACTTCGATGTCTGG
TX_702_LIVER	40	0.032	CAGDRWGYWYFDVW	TGTGCAGGAGACAGATGGGGCTACTGGTACTTCGATGTCTGG
TX_702_LIVER	17	0.014	CAREYGSSYDWYFDVW	TGTGCAAGAGAGTACGGTAGTAGCTACGACTGGTACTTCGATGTCTGG
TX_702_LIVER	6	0.005	CTPYYYGSSYIAYW	TGTACCCCATATTACTACGGTAGTAGCTACATTGCTTACTGG
TX_702_LIVER	5	0.004	CVYYYGSSYWYFDVW	TGTGTATATTACTACGGTAGTAGCTACTGGTACTTCGATGTCTGG
TX_702_LIVER	5	0.004	CAREVTGAYYFDYW	TGTGCAAGAGAGGTAACTGGGGCTTACTACTTTGACTACTGG
total	123573	100		

sample_name	templates	productive_frequency	aaSeqCDR3	clonalSequence
TX_702_BM	180472	99.605	CMRYGNYWYFDVW	TGTATGAGATATGGTAACTACTGGTACTTCGATGTCTGG
TX_702_BM	219	0.121	CVRGGGSSLYWYFDVW	TGTGTGAGAGGTGGCGGTAGTAGCCTCTACTGGTACTTCGATGTCTGG
TX_702_BM	163	0.090	CTRGYYYIWYFDVW	TGTACAAGAGGGTATTACTACATCTGGTACTTCGATGTCTGG
TX_702_BM	75	0.041	CTTGRYGIDWYFDVW	TGTACTACAGGGCGGTATGGTATCGACTGGTACTTCGATGTCTGG
TX_702_BM	66	0.036	CARRRAYGSSSRFDYW	TGTGCAAGAAGAAGGGCGTACGGTAGTAGCTCACGCTTTGACTACTGG
TX_702_BM	39	0.022	CAKHGYYWYFDVW	TGTGCCAAACATGGTTACTACTGGTACTTCGATGTCTGG
TX_702_BM	39	0.022	CAGFDVW	TGTGCGGGGTTCGATGTCTGG
TX_702_BM	30	0.017	CARNYYGSSYFDYW	TGTGCAAGAAATTACTACGGTAGTAGCTACTTTGACTACTGG
TX_702_BM	22	0.012	CARLLRYYWYFDVW	TGTGCAAGATTACTACGGTACTACTGGTACTTCGATGTCTGG
TX_702_BM	13	0.007	CARTPRRRQLLRIYAMDYW	TGTGCAAGAACCCCCCGCAGGAGACAATTACTACGGATTTATGCTATGGACTACTGG
TX_702_BM	10	0.006	CAGDYDGYWYFDVW	TGTGCAGGAGACTATGATGGTTACTGGTACTTCGATGTCTGG
TX_702_BM	6	0.003	CARNKDDYYAMDYW	TGTGCCAGAAATAAGGATGATTACTATGCTATGGACTACTGG
TX_702_BM	5	0.003	CARTMGDYDEGDYW	TGTGCCAGAACGATGGGGGATTACGACGAAGGGGACTACTGG
TX_702_BM	4	0.002	CASEDYYGSSYFSYYAMDYW	TGTGCAAGCGAGGATTACTACGGTAGTAGCTACTTTTCTTACTATGCTATGGACTACTGG
TX_702_BM	4	0.002	CARFPITTVVANWYFDVW	TGTGCAAGATTCCCGATTACTACGGTAGTAGCTAACTGGTACTTCGATGTCTGG
TX_702_BM	4	0.002	CARRVGTSPYFDYW	TGTGCAAGACGGGTTGGGACGTCCCCCTACTTTGACTACTGG
TX_702_BM	4	0.002	CATTTVVSFDYW	TGTGCAACTACTACGGTAGTTTCCTTTGACTACTGG
TX_702_BM	3	0.002	CARWGGGNFWYFDVW	TGTGCAAGATGGGGTGGGGGTAACTTCTGGTACTTCGATGTCTGG
TX_702_BM	2	0.001	CARWAIYYGNYDWYFDVW	TGTGCAAGATGGGCGATCTACTATGGTAACTACGACTGGTACTTCGATGTCTGG
TX_702_BM	2	0.001	CARSPPYYSNPHAMDYW	TGTGCAAGATCTCCTCCCTACTATAGTAACCCCCATGCTATGGACTACTGG
TX_702_BM	1	0.001	CARTPRRRQLLRMYAMDYW	TGTGCAAGAACCCCCCGCAGGAGACAATTACTACGGATGTATGCTATGGACTACTGG
TX_702_BM	1	0.001	CARGGYYYGPYAMDYW	TGTGCAAGAGGGGGGTATTACTACGGTCCTTATGCTATGGACTACTGG
TX_702_BM	1	0.001	CARLLRYYWCFDVW	TGTGCAAGATTACTACGGTACTACTGGTGCTTCGATGTCTGG
TX_702_BM	1	0.001	CARGGFAYW	TGTGCAAGGGGGGGGTTTGCTTACTGG
TX_702_BM	1	0.001	CVGFDVW	TGTGTGGGGTTCGATGTCTGG
total	181187	100		

sample_name	templates	productive_frequency	aaSeqCDR3	clonalSequence
TX_Q67_LIVER	21719	57.008	CMRYGNYWYFDVW	TGTATGAGATATGGTAACTACTGGTACTTCGATGTCTGG
TX_Q67_LIVER	12267	32.199	CARSDYYGSSSLDYW	TGTGCAAGATCAGATTACTACGGTAGTAGCTCCCTTGACTACTGG
TX_Q67_LIVER	1026	2.693	CMRYSNYWYFDVW	TGTATGAGATATAGTAACTACTGGTACTTCGATGTCTGG
TX_Q67_LIVER	1023	2.685	CMRYDGYYWYFDVW	TGTATGAGATATGATGGTTACTACTGGTACTTCGATGTCTGG
TX_Q67_LIVER	808	2.121	CMRYNSNYWYFDVW	TGTATGAGATATAATAGTAACTACTGGTACTTCGATGTCTGG
TX_Q67_LIVER	719	1.887	CMRYGSSYWYFDVW	TGTATGAGATACGGTAGTAGCTACTGGTACTTCGATGTCTGG
TX_Q67_LIVER	354	0.929	CARKEGNYYGSSWYFDVW	TGTGCAAGAAAGGAGGGAAATTACTACGGTAGTAGCTGGTACTTCGATGTCTGG
TX_Q67_LIVER	64	0.168	CASSRGLRLHYFDYW	TGTGCAAGCTCGAGGGGGCTCAGGCTCCACTACTTTGACTACTGG
TX_Q67_LIVER	55	0.144	CARWGRDSSGYFDYW	TGTGCAAGATGGGGGAGGGACAGCTCAGGCTACTTTGACTACTGG
TX_Q67_LIVER	14	0.037	CARSYGSNWFAYW	TGTGCAAGATCCTACGGTAGTAACTGGTTTGCTTACTGG
TX_Q67_LIVER	12	0.031	CAGDITTVVATWSYWYFDVW	TGTGCAGGAGACATTACTACGGTAGTAGCTACGTGGAGCTACTGGTACTTCGATGTCTGG
TX_Q67_LIVER	10	0.026	CARNWAGDYAMDYW	TGTGCAAGGAACTGGGCCGGGGACTATGCTATGGACTACTGG
TX_Q67_LIVER	2	0.005	CARKEGNYYGSSSLDYW	TGTGCAAGAAAGGAGGGAAATTACTACGGTAGTAGCTCCCTTGACTACTGG
TX_Q67_LIVER	2	0.005	CARSDYYGSSWYFDVW	TGTGCAAGATCAGATTACTACGGTAGTAGCTGGTACTTCGATGTCTGG
TX_Q67_LIVER	2	0.005	CARYYYDYDWYFDVW	TGTGCAAGATACTACTATGATTACGACTGGTACTTCGATGTCTGG
TX_Q67_LIVER	2	0.005	CARKVPHGYFDVW	TGTGCAAGAAAAGTACCTCACGGGTACTTCGATGTCTGG
TX_Q67_LIVER	1	0.003	CARHHPIRDYGYWYFDVW	TGTGCAAGACACCACCCAATACGGGACTACGGCTACTGGTACTTCGATGTCTGG
TX_Q67_LIVER	1	0.003	CARGTSYYDYDGWYFDVW	TGTGCAAGAGGGACCTCCTACTATGATTACGACGGATGGTACTTCGATGTCTGG
TX_Q67_LIVER	1	0.003	CARSSFYGSSYNWYFDVW	TGTGCAAGAAGCTCCTTCTACGGTAGTAGCTACAACTGGTACTTCGATGTCTGG
TX_Q67_LIVER	1	0.003	CASMVTRSYWYFDVW	TGTGCCTCTATGGTTACGAGGAGCTACTGGTACTTCGATGTCTGG
TX_Q67_LIVER	1	0.003	CARRDSNSYWYFDVW	TGTGCAAGACGGGATAGTAACTCCTACTGGTACTTCGATGTCTGG
TX_Q67_LIVER	1	0.003	CARDLTGTRWYFDVW	TGTGCAAGAGATCTAACTGGGACGAGGTGGTACTTCGATGTCTGG
TX_Q67_LIVER	1	0.003	CAGDTDGYFGFAYW	TGTGCAGGAGACACCGATGGTTACTTCGGGTTTGCTTACTGG
TX_Q67_LIVER	1	0.003	CARKEITTGYFDVW	TGTGCAAGAAAGGAGATTACTACGGGGTACTTCGATGTCTGG
TX_Q67_LIVER	1	0.003	CAKAYGSSPLFDYW	TGTGCAAAGGCTTACGGTAGTAGCCCTCTTTTTGACTACTGG
TX_Q67_LIVER	1	0.003	CARSLHGNYFDYW	TGTGCAAGATCCCTACATGGTAACTACTTTGACTACTGG
TX_Q67_LIVER	1	0.003	CAREAIPSYFDYW	TGTGCAAGAGAGGCAATACCGAGCTACTTTGACTACTGG
TX_Q67_LIVER	1	0.003	CVRGYSWYFDVW	TGTGTGAGAGGTTACTCCTGGTACTTCGATGTCTGG
TX_Q67_LIVER	1	0.003	CAGGQGYYFDYW	TGTGCTGGGGGACAGGGCTACTACTTTGACTACTGG
TX_Q67_LIVER	1	0.003	CARATVVAFDYW	TGTGCTAGAGCGACGGTAGTAGCTTTTGACTACTGG
TX_Q67_LIVER	1	0.003	CARAFDGYLDYW	TGTGCAAGAGCGTTTGATGGTTATCTTGACTACTGG
TX_Q67_LIVER	1	0.003	CARDQAYAMDYW	TGTGCAAGAGATCAGGCGTATGCTATGGACTACTGG
TX_Q67_LIVER	1	0.003	CARRSGYFDVW	TGTGCAAGAAGGTCCGGGTACTTCGATGTCTGG
TX_Q67_LIVER	1	0.003	CARAGNFDVW	TGTGCAAGGGCGGGGAACTTCGATGTCTGG
TX_Q67_LIVER	1	0.003	CARGGYFDVW	TGTGCAAGGGGAGGGTACTTCGATGTCTGG
total	38098	100.000		

sample_name	templates	productive_frequency	aaSeqCDR3	clonalSequence
TX_Q67_LNP	8268	99.232	CARSDYYGSSSLDYW	TGTGCAAGATCAGATTACTACGGTAGTAGCTCCCTTGACTACTGG
TX_Q67_LNP	31	0.372	CMRYGNYWYFDVW	TGTATGAGATATGGTAACTACTGGTACTTCGATGTCTGG
TX_Q67_LNP	10	0.120	CAGDITTVVATWSYWYFDVW	TGTGCAGGAGACATTACTACGGTAGTAGCTACGTGGAGCTACTGGTACTTCGATGTCTGG
TX_Q67_LNP	7	0.084	CMRYSNYWYFDVW	TGTATGAGATATAGTAACTACTGGTACTTCGATGTCTGG
TX_Q67_LNP	5	0.060	CARTAVYYDYAWFAYW	TGTGCAAGAACGGCGGTCTACTATGATTACGCCTGGTTTGCTTACTGG
TX_Q67_LNP	2	0.024	CASMVTRSYWYFDVW	TGTGCCTCTATGGTTACGAGGAGCTACTGGTACTTCGATGTCTGG
TX_Q67_LNP	1	0.012	CASFGTTNWYFDVW	TGTGCAAGCTTCGGGACTACGAACTGGTACTTCGATGTCTGG
TX_Q67_LNP	1	0.012	CARDGGYSDYFDYW	TGTGCAAGAGATGGGGGTTACTCGGACTACTTTGACTACTGG
TX_Q67_LNP	1	0.012	CAIHYYGSSLDYW	TGTGCAATTCATTACTACGGTAGTAGCCTTGACTACTGG
TX_Q67_LNP	1	0.012	CAREVGQGGLDYW	TGTGCAAGGGAAGTGGGACAAGGGGGACTGGACTACTGG
TX_Q67_LNP	1	0.012	CARGGLYDGFDYW	TGTGCAAGAGGGGGGCTATATGATGGTTTTGACTACTGG
TX_Q67_LNP	1	0.012	CARRGLLSHFDYW	TGTGCAAGAAGAGGACTACTATCCCACTTTGACTACTGG
TX_Q67_LNP	1	0.012	CARHPSGHFDYW	TGTGCAAGACATCCTTCAGGCCACTTTGACTACTGG
TX_Q67_LNP	1	0.012	CTNYDYDCW	TGCACAAACTATGATTACGACTGCTGG
TX_Q67_LNP	1	0.012	CARHLHYW	TGTGCAAGACATCTTCACTACTGG
total	8332	100		

sample_name	templates	productive_frequency	aaSeqCDR3	clonalSequence
TX_P42	178685	99.868	CMRYSNYWYFDVW	TGTATGAGATATAGTAACTACTGGTACTTCGATGTCTGG
TX_P42	150	0.084	CAGDYDGYWYFDVW	TGTGCAGGAGACTATGATGGTTACTGGTACTTCGATGTCTGG
TX_P42	69	0.039	CARRHYYGSYYAMDYW	TGTGCAAGAAGGCATTACTACGGTAGTTACTATGCTATGGACTACTGG
TX_P42	9	0.005	CMRYGSSYWYFDVW	TGTATGAGATATGGTAGTAGCTACTGGTACTTCGATGTCTGG
TX_P42	8	0.004	CAGDITTVVATWSYWYFDVW	TGTGCAGGAGACATTACTACGGTAGTAGCTACGTGGAGCTACTGGTACTTCGATGTCTGG
total	178921	100		

sample_name	templates	productive_frequency	aaSeqCDR3	clonalSequence
TX_P43	162746	99.572	CMRYSNYWYFDVW	TGTATGAGATATAGTAACTACTGGTACTTCGATGTCTGG
TX_P43	619	0.379	CAGDYDGYWYFDVW	TGTGCAGGAGACTATGATGGTTACTGGTACTTCGATGTCTGG
TX_P43	62	0.038	CARRHYYGSYYAMDYW	TGTGCAAGAAGGCATTACTACGGTAGTTACTATGCTATGGACTACTGG
TX_P43	7	0.004	CAGDITTVVATWSYWYFDV[illegible]	TGTGCAGGAGACATTACTACGGTAGTAGCTACGTGGAGCTACTGGTACTTCGATGTCTGG
TX_P43	7	0.004	CARIYYGNYWYFDVW	TGTGCAAGAATCTACTATGGTAACTACTGGTACTTCGATGTCTGG
TX_P43	3	0.002	CMRYGSSYWYFDVW	TGTATGAGATATGGTAGTAGCTACTGGTACTTCGATGTCTGG
TX_P43	1	0.001	CMSYSNYWYFYVR	TGTATGAGTTATAGTAACTACTGGTACTTCTATGTCCGG
total	163445	100		

sample_name	templates	productive_frequency	aaSeqCDR3	clonalSequence
TX_CD92	167606	99.961	CMRYGNYWYFDVW	TGTATGAGATATGGTAACTACTGGTACTTCGATGTCTGG
TX_CD92	53	0.032	CARRHYYGSYYAMDYW	TGTGCAAGAAGGCATTACTACGGTAGTTACTATGCTATGGACTACTGG
TX_CD92	8	0.005	CAGDITTVVATWSYWYFD[illegible]	TGTGCAGGAGACATTACTACGGTAGTAGCTACGTGGAGCTACTGGTACTTCGATGTCTGG
TX_CD92	3	0.002	CMRYGSSYWYFDVW	TGTATGAGATATGGTAGTAGCTACTGGTACTTCGATGTCTGG
TX_CD92	1	0.001	CVYYYGSSYWYFDVW	TGTGTATATTACTACGGTAGTAGCTACTGGTACTTCGATGTCTGG
total	167671	100		

sample_name	templates	productive_frequency	aaSeqCDR3	clonalSequence
TX_CD95	98126	90.753	CAGDITTVVATWSYWYFDVW	TGTGCAGGAGACATTACTACGGTAGTAGCTACGTGGAGCTACTGGTACTTCGATGTCTGG
TX_CD95	9935	9.189	CMRYGNYWYFDVW	TGTATGAGATATGGTAACTACTGGTACTTCGATGTCTGG
TX_CD95	38	0.035	CARRHYYGSYYAMDYW	TGTGCAAGAAGGCATTACTACGGTAGTTACTATGCTATGGACTACTGG
TX_CD95	22	0.020	CATRGYGSSYEGYFDYW	TGTGCAACGAGGGGCTACGGTAGTAGCTACGAGGGCTACTTTGACTACTGG
TX_CD95	2	0.002	CAGDRWGYWYFDVW	TGTGCAGGAGACAGATGGGGCTACTGGTACTTCGATGTCTGG
TX_CD95	1	0.001	CAGDITVVATWSYWYFDVW	TGTGCAGGAGACATTACGGTAGTAGCTACGTGGAGCTACTGGTACTTCGATGTCTGG
total	108124	100		

sample_name	templates	productive_frequency	aaSeqCDR3	clonalSequence
TX_Q82	129970	99.012	CMRYGNYWYFDVW	TGTATGAGATATGGTAACTACTGGTACTTCGATGTCTGG
TX_Q82	1267	0.965	CVYYYGSSYWYFDVW	TGTGTATATTACTACGGTAGTAGCTACTGGTACTTCGATGTCTGG
TX_Q82	14	0.011	CARIYYGNYWYFDVW	TGTGCAAGAATCTACTATGGTAACTACTGGTACTTCGATGTCTGG
TX_Q82	5	0.004	CAKTGAGYFDVW	TGTGCCAAAACTGGGGCGGGGTACTTCGATGTCTGG
TX_Q82	4	0.003	CAGDRWGYWYFDVW	TGTGCAGGAGACAGATGGGGCTACTGGTACTTCGATGTCTGG
TX_Q82	3	0.002	CARLTIRITTVVDYYAMDYW	TGTGCAAGATTGACGATTCGGATTACTACGGTAGTAGATTACTATGCTATGGACTACTGG
TX_Q82	2	0.002	CARRGDGYYFDYW	TGTGCAAGAAGGGGGGATGGTTACTACTTTGACTACTGG
TX_Q82	1	0.001	TLYLQMSNVRSEDTATYFCMRYG[illegible]	ACCCTGTACCTGCAGATGAGCAATGTGCGATCGGAGGACACAGCCACGTATTTCTGTATGAGATATGGTAACTACTGGAACTTCGATGTCTGG
TX_Q82	1	0.001	LRYGNYWYFDVW	TTGAGATATGGTAACTACTGGTACTTCGATGTCTGG
total	131267	100		

sample_name	templates	productive_frequency	aaSeqCDR3	clonalSequence
TX_Q83	148869	99.774	CMRYGNYWYFDVW	TGTATGAGATATGGTAACTACTGGTACTTCGATGTCTGG
TX_Q83	329	0.221	CVYYYGSSYWYFDVW	TGTGTATATTACTACGGTAGTAGCTACTGGTACTTCGATGTCTGG
TX_Q83	4	0.003	CARSGWDKGFAYW	TGTGCAAGATCGGGCTGGGACAAGGGGTTTGCTTACTGG
TX_Q83	3	0.002	CMRYGSSYWYFDVW	TGTATGAGATACGGTAGTAGCTACTGGTACTTCGATGTCTGG
TX_Q83	1	0.001	CLTGTGDYFDYW	TGTCTAACTGGGACCGGGGACTACTTTGACTACTGG
total	149206	100		

sample_name	templates	productive_frequency	aaSeqCDR3	clonalSequence
TX_O9	16899	55.833	CMRYGNYWYFDVW	TGTATGAGATATGGTAACTACTGGTACTTCGATGTCTGG
TX_O9	9550	31.553	CARSDYYGSSSLDYW	TGTGCAAGATCAGATTACTACGGTAGTAGCTCCCTTGACTACTGG
TX_O9	3131	10.345	CARLYYGSSYWYFDVW	TGTGCCCGCCTTTACTACGGTAGTAGCTACTGGTACTTCGATGTCTGG
TX_O9	599	1.979	CARLANWDWYFDVW	TGTGCAAGACTAGCTAACTGGGACTGGTACTTCGATGTCTGG
TX_O9	47	0.155	CARLYYGSSSLDYW	TGTGCCCGCCTTTACTACGGTAGTAGCTCCCTTGACTACTGG
TX_O9	17	0.056	CARSDYYGSSYWYFDVW	TGTGCAAGATCAGATTACTACGGTAGTAGCTACTGGTACTTCGATGTCTGG
TX_O9	10	0.033	CMRYGSSYWYFDVW	TGTATGAGATATGGTAGTAGCTACTGGTACTTCGATGTCTGG
TX_O9	8	0.026	CMRYGSSYWYFDVW	TGTATGAGATACGGTAGTAGCTACTGGTACTTCGATGTCTGG
TX_O9	3	0.010	CAGDRWGYWYFDVW	TGTGCAGGAGACAGATGGGGCTACTGGTACTTCGATGTCTGG
TX_O9	1	0.003	CTRRVYYYGSSLYYYAMDYW	TGTACCAGGCGGGTTTATTACTACGGTAGTAGCCTATATTACTATGCTATGGACTACTGG
TX_O9	1	0.003	CARSDYYGSSYWYLDVW	TGTGCAAGATCAGATTACTACGGTAGTAGCTACTGGTACCTCGATGTCTGG
TX_O9	1	0.003	CTRSDYYGSSYWYFDVW	TGTACAAGATCAGATTACTACGGTAGTAGCTACTGGTACTTCGATGTCTGG
total	30267	100		

sample_name	templates	productive_frequency	aaSeqCDR3	clonalSequence
TX_O11	13477	98.936	CARSDYYGSSSLDYW	TGTGCAAGATCAGATTACTACGGTAGTAGCTCCCTTGACTACTGG
TX_O11	53	0.389	CMRYSNYWYFDVW	TGTATGAGATATAGTAACTACTGGTACTTCGATGTCTGG
TX_O11	35	0.257	CARLYYGSSYWYFDVW	TGTGCCCGCCTTTACTACGGTAGTAGCTACTGGTACTTCGATGTCTGG
TX_O11	19	0.139	CMRYGSSYWYFDVW	TGTATGAGATACGGTAGTAGCTACTGGTACTTCGATGTCTGG
TX_O11	16	0.117	CMRYGNYWYFDVW	TGTATGAGATATGGTAACTACTGGTACTTCGATGTCTGG
TX_O11	10	0.073	CMRYGSSYWYFDVW	TGTATGAGATATGGTAGTAGCTACTGGTACTTCGATGTCTGG
TX_O11	8	0.059	CASGGKGYAMDYW	TGTGCTAGTGGGGGTAAAGGCTATGCTATGGACTACTGG
TX_O11	4	0.029	CAGDRWGYWYFDVW	TGTGCAGGAGACAGATGGGGCTACTGGTACTTCGATGTCTGG
total	13622	100		

sample_name	templates	productive_frequency	aaSeqCDR3	clonalSequence
TX_642	8196	58.058	CARSDYYGSSSLDYW	TGTGCAAGATCAGATTACTACGGTAGTAGCTCCCTTGACTACTGG
TX_642	5244	37.147	CMRYGNYWYFDVW	TGTATGAGATATGGTAACTACTGGTACTTCGATGTCTGG
TX_642	213	1.509	CARDPYYYYGSSYWYFDVW	TGTGCAAGAGATCCCTATTATTACTACGGTAGTAGCTACTGGTACTTCGATGTCTGG
TX_642	111	0.786	CTELLRYTDYW	TGCACAGAATTACTACGGTACACTGACTACTGG
TX_642	107	0.758	CARHEGGDYDYDSWYFDVW	TGTGCAAGACACGAAGGAGGCGACTATGATTACGACTCTTGGTACTTCGATGTCTGG
TX_642	87	0.616	CARERYYYGSNYFDYW	TGTGCAAGAGAGAGGTATTACTACGGTAGTAACTACTTTGACTACTGG
TX_642	86	0.609	CARSGLIGTGYFDYW	TGTGCAAGATCAGGGCTAATTGGGACGGGGTACTTTGACTACTGG
TX_642	29	0.205	CARGGYYSFDYW	TGTGCAAGAGGTGGTTACTACTCCTTTGACTACTGG
TX_642	18	0.128	CVRAYDGYYVGIYYAMDYW	TGTGTGAGGGCCTATGATGGTTACTACGTAGGGATTTACTATGCTATGGACTACTGG
TX_642	6	0.043	CAGDITTVVATWSYWYFDVW	TGTGCAGGAGACATTACTACGGTAGTAGCTACGTGGAGCTACTGGTACTTCGATGTCTGG
TX_642	2	0.014	CARSGGYYYGSSYGYFDVW	TGTGCAAGATCAGGGGGTTATTACTACGGTAGTAGCTACGGGTACTTCGATGTCTGG
TX_642	2	0.014	CARGGYYDYDGGAWFTYW	TGTGCAAGAGGGGGCTACTATGATTACGACGGGGGGGCCTGGTTTACTTACTGG
TX_642	2	0.014	CARRGTGTGYFDYW	TGTGCAAGACGGGGAACTGGGACGGGGTACTTTGACTACTGG
TX_642	2	0.014	CARGGYGNGAMDYW	TGTGCAAGAGGGGGGTATGGTAATGGGGCTATGGACTACTGG
TX_642	1	0.007	CARSHYYGSSYPLYWYFDVW	TGTGCAAGATCACATTACTACGGTAGTAGCTACCCCCTCTACTGGTACTTCGATGTCTGG
TX_642	1	0.007	CARHVEGSSYVGYFDVW	TGTGCCAGACATGTTGAGGGTAGTAGCTACGTAGGGTACTTCGATGTCTGG
TX_642	1	0.007	CARDPRGSSSGSFDYW	TGTGCAAGAGATCCCCGCGGTAGTAGCTCTGGTTCCTTTGACTACTGG
TX_642	1	0.007	CVRMNYYGSRHFDVW	TGTGTGAGAATGAATTACTACGGTAGTAGGCACTTCGATGTCTGG
TX_642	1	0.007	CARGYDSNWYFDVW	TGTGCAAGAGGCTATGATAGTAACTGGTACTTCGATGTCTGG
TX_642	1	0.007	CARSGNYWYFDVW	TGTGCAAGATCCGGTAACTACTGGTACTTCGATGTCTGG
TX_642	1	0.007	CARGGLLRYFDYW	TGTGCAAGAGGGGGATTACTACGGTACTTTGACTACTGG
TX_642	1	0.007	CVRGYSWYFDVW	TGTGTGAGAGGTTACTCCTGGTACTTCGATGTCTGG
TX_642	1	0.007	CAREGTWYFDVW	TGTGCAAGAGAGGGGACCTGGTACTTCGATGTCTGG
TX_642	1	0.007	CTRRWWYFDVW	TGTACCAGGCGATGGTGGTACTTCGATGTCTGG
TX_642	1	0.007	CARTPYFDYW	TGTGCAAGAACCCCTTACTTTGACTACTGG
TX_642	1	0.007	CAREYFDYW	TGTGCAAGGGAATACTTTGACTACTGG
total	14117	100		

sample_name	templates	productive_frequency	aaSeqCDR3	clonalSequence
TX_702	203247	99.900	CMRYGNYWYFDVW	TGTATGAGATATGGTAACTACTGGTACTTCGATGTCTGG
TX_702	102	0.050	CANFLGGFDVW	TGTGCAAATTTCCTAGGGGGCTTCGATGTCTGG
TX_702	61	0.030	CARRHYYGSYYAMDYW	TGTGCAAGAAGGCATTACTACGGTAGTTACTATGCTATGGACTACTGG
TX_702	10	0.005	CAGDRWGYWYFDVW	TGTGCAGGAGACAGATGGGGCTACTGGTACTTCGATGTCTGG
TX_702	8	0.004	CTGRSYGFAYW	TGTACAGGGCGGTCTTACGGGTTTGCTTACTGG
TX_702	7	0.003	CARGDYYGSSLWYFDVW	TGTGCAAGAGGGGATTACTACGGTAGTAGCCTCTGGTACTTCGATGTCTGG
TX_702	5	0.002	CARRYSNYYAMDYW	TGTGCAAGAAGGTATAGTAACTACTATGCTATGGACTACTGG
TX_702	4	0.002	CAGDITTVVATWSYWYFDVW	TGTGCAGGAGACATTACTACGGTAGTAGCTACGTGGAGCTACTGGTACTTCGATGTCTGG
TX_702	3	0.001	CARLLRYYWYFDVW	TGTGCAAGATTACTACGGTACTACTGGTACTTCGATGTCTGG
TX_702	3	0.001	CRVDTTGGDCW	TGTAGAGTGGATACCACCGGCGGGGACTGCTGG
total	203450	100		

sample_name	templates	productive_frequer	aaSeqCDR3	clonalSequence
TX_703	175291	99.979	CMRYGNYWYFDVW	TGTATGAGATATGGTAACTACTGGTACTTCGATGTCTGG
TX_703	26	0.015	CARRHYYGSYYAMDYW	TGTGCAAGAAGGCATTACTACGGTAGTTACTATGCTATGGACTACTGG
TX_703	5	0.003	CTTWGNW	TGTACCACCTGGGGGAACTGG
TX_703	3	0.002	CARRAYWYFDVW	TGTGCAAGAAGGGCGTACTGGTACTTCGATGTCTGG
TX_703	1	0.001	CAGDRWGYWYFDVW	TGTGCAGGAGACAGATGGGGCTACTGGTACTTCGATGTCTGG
TX_703	1	0.001	CMRYGNWYFDVW	TGTATGAGATATGGTAACTGGTACTTCGATGTCTGG
total	175327	100		

sample_name	productive_frequency	templates	amino_acid
TX6_Q67_2	99.881	53681	CARSDYYGSSSLDYW
TX6_Q67_2	0.007	4	CARSDYYGSSSLDYW
TX6_Q67_2	0.007	4	CARSDYYGSSSLDYW
TX6_Q67_2	0.004	2	CARSDYYGSSSLDYW
TX6_Q67_2	0.004	2	CARSDYYGSSSLDYW
TX6_Q67_2	0.004	2	CARSDYYGSSSLDYW
TX6_Q67_2	0.004	2	CARSDYYGSSSLDYW
TX6_Q67_2	0.004	2	CARSDYYGSSSLDYW
TX6_Q67_2	0.004	2	CARSDYYGSSSLDYW
TX6_Q67_2	0.004	2	CARSDYYGSSSLDYW
TX6_Q67_2	0.004	2	CARSDYYGSSSLDYW
TX6_Q67_2	0.004	2	CARSDYYGSSSLDYW
TX6_Q67_2	0.004	2	SEARLRYFDVW
TX6_Q67_2	0.002	1	CFWTQIRLRYFDVW
TX6_Q67_2	0.002	1	CTARSCRLRYFDVW
TX6_Q67_2	0.002	1	CAREGVYYDYESWYFDVW
TX6_Q67_2	0.002	1	CARSDYYGSSSLDYW
TX6_Q67_2	0.002	1	CARSDYYGSSSLDYW
TX6_Q67_2	0.002	1	CARSDYYGSSSLDYW
TX6_Q67_2	0.002	1	CARSDYYGSSSLDYW
TX6_Q67_2	0.002	1	CARSDYYGSSSLDYW
TX6_Q67_2	0.002	1	CARSDYYGSSSLDYW
TX6_Q67_2	0.002	1	CARSDYYGSSSLDYW
TX6_Q67_2	0.002	1	CARSDYYGSSSLDYW
TX6_Q67_2	0.002	1	CARSDYYGSSSLDYW
TX6_Q67_2	0.002	1	CARSDYYGSSSLDYW
TX6_Q67_2	0.002	1	CARSDYYGSSSLDYW
TX6_Q67_2	0.002	1	CARSDYYGSSSLDYW
TX6_Q67_2	0.002	1	CARSDYYGSSSLDYW
TX6_Q67_2	0.002	1	CARAGYYLAGFAYW
TX6_Q67_2	0.002	1	CMRYSNYWYFDVW
TX6_Q67_2	0.002	1	CARNSYGYDLWFAYW
TX6_Q67_2	0.002	1	CMNRSNVYAMDYW
TX6_Q67_2	0.002	1	CAQGALTRLRYFDVW
TX6_Q67_2	0.002	1	CAERGDRLRYFDVW
TX6_Q67_2	0.002	1	CARSDYYGSSSLDYW
TX6_Q67_2	0.002	1	CASYRTRRLRYFDVW
TX6_Q67_2	0.002	1	CARSDYYGSSSLDYW
TX6_Q67_2	0.002	1	CARSDYYGSSSLDYW
TX6_Q67_2	0.002	1	CARSDYYGSSSLDYW
TX6_Q67_2	0.002	1	CARSDYYGSSSLDYW
TX6_Q67_2	0.002	1	CARSDYYGSSSLDYW
TX6_Q67_2	0.002	1	CARSDYYGSSSLDYW
TX6_Q67_2	0.002	1	CARSDYYGSSSLDYW
TX6_Q67_2	0.002	1	CARSDYYGSSSLDYW
TX6_Q67_2	0.002	1	CARSDYYGSSSLDYW
TX6_Q67_2	0.002	1	CARSDYYGSSSLDYW
TX6_Q67_2	0.002	1	CARSDYYGSSSLDYW
TX6_Q67_2	0.002	1	CARSDYYGSSSLDYW
total	100.000	53745	

rearrangement
CATTGACTGTAGACAAGTCCTCCAGCACAGCCTACATGGAGCTCCGCAGCCTGACATCTGAGGACTCTGCAGTCTATTACTGTGCAAGATCAGATTACTACGGTAGTAGCTCCCTTGACTACTGG
ACATTGACTGTAGACAAGTCCTCAGCACAGCCTACATGGAGCTCCGCAGCCTGACATCTGAGGACTCTGCAGTCTATTACTGTGCAAGATCAGATTACTACGGTAGTAGCTCCCTTGACTACTGG
ACATTGACTGTAGACAAGTCCTCCAGCACAGCCTACATGAGCTCCGCAGCCTGACATCTGAGGACTCTGCAGTCTATTACTGTGCAAGATCAGATTACTACGGTAGTAGCTCCCTTGACTACTGG
ACATTGACTGTAGACAAGTCCCCAGCACAGCCTACATGGAGCTCCGCAGCCTGACATCTGAGGACTCTGCAGTCTATTACTGTGCAAGATCAGATTACTACGGTAGTAGCTCCCTTGACTACTGG
CATTGACTGCAGACAAATCCTCCAGCACAGCCTACATGCAACTGAGCAGCCTGACATCTGAGGACTCTGCAGTCTATTACTGTGCAAGATCAGATTACTACGGTAGTAGCTCCCTTGACTACTGG
ACATTGACTGTAGACAAGTCTCCAGCACAGCCTACATGGAGCTCCGCAGCCTGACATCTGAGGACTCTGCAGTCTATTACTGTGCAAGATCAGATTACTACGGTAGTAGCTCCCTTGACTACTGG
ACATTGACTGTGACAAGTCCTCCAGCACAGCCTACATGGAGCTCCGCAGCCTGACATCTGAGGACTCTGCAGTCTATTACTGTGCAAGATCAGATTACTACGGTAGTAGCTCCCTTGACTACTGG
ACATTGACTGTAGACAGTCCTCCAGCACAGCCTACATGGAGCTCCGCAGCCTGACATCTGAGGACTCTGCAGTCTATTACTGTGCAAGATCAGATTACTACGGTAGTAGCTCCCTTGACTACTGG
ACATGACTGTAGACAAGTCCTCCAGCACAGCCTACATGGAGCTCCGCAGCCTGACATCTGAGGACTCTGCAGTCTATTACTGTGCAAGATCAGATTACTACGGTAGTAGCTCCCTTGACTACTGG
ACATTGACTGTAGACAAGTCCTCCAGCACAGCCTACATGGAGCCCGCAGCCTGACATCTGAGGACTCTGCAGTCTATTACTGTGCAAGATCAGATTACTACGGTAGTAGCTCCCTTGACTACTGG
ACATTGACTGTAGACAATCCTCCAGCACAGCCTACATGGAGCTCCGCAGCCTGACATCTGAGGACTCTGCAGTCTATTACTGTGCAAGATCAGATTACTACGGTAGTAGCTCCCTTGACTACTGG
ACATTGACTGTAGACAAGTCCTCCAGCACAGCCACATGGAGCTCCGCAGCCTGACATCTGAGGACTCTGCAGTCTATTACTGTGCAAGATCAGATTACTACGGTAGTAGCTCCCTTGACTACTGG
AGGGCAAGGCCACATTGACTGTCGACAATACCTCCAGCACAGCCTACATGGAGCTCGGCAGCCTGACTTCTGAGGACTCTGCGGTCTATTACTCTGAGGCCCGTCTTAGGTACTTCGATGTCTGG
TCACCATCTCCAGAGACAATGCCAAGAACACCCTGTTTCTGCAAATGAGCAGTCTAAGGTATGAGGACACGGCCATGTATTACTGTTTCTGGACGCAAATCCGTCTTAGGTACTTCGATGTCTGG
TCACCATCTCCAGAGACAATGCCAAGAACACCCTGTACCTGCAAATGAGCAGTCTGAAGTCTGAGGACACAGCCATGTATTACTGTACAGCACGTAGCTGCCGTCTTAGGTACTTCGATGTCTGG
CAGACACATCCTCCAACACAGCCTACCTGCAGCTCAGCAGCCTGACATCTGAGGACACTGCCATCTATTACTGTGCTAGAGAAGGGGTCTACTATGATTACGAGAGCTGGTACTTCGATGTCTGG
ACATTGACTGTAGAAAGTCCTCCAGCACAGCCTACATGGAGCTCCGCAGCCTGACATCTGAGGACTCTGCAGTCTATTACTGTGCAAGATCAGATTACTACGGTAGTAGCTCCCTTGACTACTGG
CATTGACTGTAGACAAATCCTCCAGCACAGCCTACATGCAGCTCAACAGCCTGACATCTGAGGACTCTGCAGTCTATTACTGTGCAAGATCAGATTACTACGGTAGTAGCTCCCTTGACTACTGG
ACATTACTGTAGACAAGTCCTCCAGCACAGCCTACATGGAGCTCCGCAGCCTGACATCTGAGGACTCTGCAGTCTATTACTGTGCAAGATCAGATTACTACGGTAGTAGCTCCCTTGACTACTGG
CACTGACTGCAGACACATCCTCCAGCACAGCCTACATGCAGCTCAGCAGCCTGACATCTGAGGACTCTGCAGTCTATTTCTGTGCAAGATCAGATTACTACGGTAGTAGCTCCCTTGACTACTGG
CACTGACTTCAGACAAATCCTCCAGCACAGCCTACATGGAGCTCAGCAGCCTGACCTCTGAGGACTCTGCGGTCTATTACTGTGCAAGATCAGATTACTACGGTAGTAGCTCCCTTGACTACTGG
ACATTGACTGTAGACAAGTCCTCCAGCACAGCCTACTGGAGCTCCGCAGCCTGACATCTGAGGACTCTGCAGTCTATTACTGTGCAAGATCAGATTACTACGGTAGTAGCTCCCTTGACTACTGG
ACATTGACTGTAGACAAGTCCTCCAGCACAGCTACATGGAGCTCCGCAGCCTGACATCTGAGGACTCTGCAGTCTATTACTGTGCAAGATCAGATTACTACGGTAGTAGCTCCCTTGACTACTGG
CACATTGACTGTAGACAAGTCCTAGCACAGCCTACATGGAGCTCCGCAGCCTGACATCTGAGGACTCTGCAGTCTATTACTGTGCAAGATCAGATTACTACGGTAGTAGCTCCCTTGACTACTGG
CACATTGACTGTACAAGTCCTCCAGCACAGCCTACATGGAGCTCCGCAGCCTGACATCTGAGGACTCTGCAGTCTATTACTGTGCAAGATCAGATTACTACGGTAGTAGCTCCCTTGACTACTGG
GATTTTCTGTAGACAAGTCCTCCAGCACAGCCTACATGGAGCTCCGCAGCCTGACATCTGAGGACTCTGCAGTCTATTACTGTGCAAGATCAGATTACTACGGTAGTAGCTCCCTTGACTACTGG
CTTTTAATGTAGACAAGTCCTCCAGCACAGCCTACATGGAGCTCCGCAGCCTGACATCTGAGGACTCTGCAGTCTATTACTGTGCAAGATCAGATTACTACGGTAGTAGCTCCCTTGACTACTGG
CATGGCCGGTAGACAAGTCCTCCAGCACAGCCTACATGGAGCTCCGCAGCCTGACATCTGAGGACTCTGCAGTCTATTACTGTGCAAGATCAGATTACTACGGTAGTAGCTCCCTTGACTACTGG
ACATTGACTGTAGACAAGCCTCCAGCACAGCCTACATGGAGCTCCGCAGCCTGACATCTGAGGACTCTGCAGTCTATTACTGTGCAAGATCAGATTACTACGGTAGTAGCTCCCTTGACTACTGG
CCACACTGACTGTAGACAAATCCTCCAGCACAGCCTACATGCAACTCAGCAGCCTGACATCTGAGGACTCTGCGGTCTATTACTGTGCAAGGGCGGGTTACTACCTCGCCGGGTTTGCTTACTGG
GATTCACTATCTTCAGAGACAATGACAAGAGCACCCTGTACCTGCAGATGAGCAATGTGCGATCGGAGGACACAGCCACGTATTTCTGTATGAGATATAGTAACTACTGGTACTTCGATGTCTGG
CAATCTCCAAGGATACCTCCAGAAACCAGGTATTCCTCAAGATCACCAGTGTGGACACTGCAGATACTGCCACATACTACTGTGCTCGAAATTCTTATGGTTACGACCTCTGGTTTGCTTACTGG
AAGCCACATTGACTGCAAACACATCCTCGAGCACAGCCTACATGGAACTCAGCAGCCTCACATCTGAGGACTCTGCGGTCTATTACTGTATGAATAGGTCCAACGTGTATGCTATGGACTACTGG
TCATCTCCAGAGACAATACCAAGAAGACCCTGTACCTGCAAATGAGCAGTCTGAGGTCTGAGGACACAGCCTTGTATTACTGTGCACAGGGTGCCCTGACCCGTCTTAGGTACTTCGATGTCTGG
TCACCATCTCCAGAGACAATGCCAAGAACAACCTGTACCTGCAAATGAGCAGTCTGAGGTCTGAGGACACGGCCTTGTATTACTGTGCAGAAAGAGGCGACCGTCTTAGGTACTTCGATGTCTGG
ACATTGACTGAGACAAGTCCTCCAGCACAGCCTACATGGAGCTCCGCAGCCTGACATCTGAGGACTCTGCAGTCTATTACTGTGCAAGATCAGATTACTACGGTAGTAGCTCCCTTGACTACTGG
GAATCTCCAAGGATACCTCCAACAACCAGGTATTCCTCAAGATCACCACTGTGGACACTGTAGATACTGCCACATACTACTGTGCTTCCTATCGTACTCGCCGTCTTAGGTACTTCGATGTCTGG
ACATTGACTGTAGACAAGTCCTCCAGACAGCCTACATGGAGCTCCGCAGCCTGACATCTGAGGACTCTGCAGTCTATTACTGTGCAAGATCAGATTACTACGGTAGTAGCTCCCTTGACTACTGG
CTGTGTCTGTAGACAAGTCCTCCAGCACAGCCTACATGGAGCTCCGCAGCCTGACATCTGAGGACTCTGCAGTCTATTACTGTGCAAGATCAGATTACTACGGTAGTAGCTCCCTTGACTACTGG
CCTGGCCTGTAGACAAGTCCTCCAGCACAGCCTACATGGAGCTCCGCAGCCTGACATCTGAGGACTCTGCAGTCTATTACTGTGCAAGATCAGATTACTACGGTAGTAGCTCCCTTGACTACTGG
CATTAACTGTAGACAAGTCATCCAGCACAGCCTACATGCAGCTCAAGAGCCTGACATCTGAGGACTCTGCTGTCTATTACTGTGCAAGATCAGATTACTACGGTAGTAGCTCCCTTGACTACTGG
CATTGACTGTAGACACATCCTCCAGCACAGCCTACATGCAGCTCAGCAGCCTGACATCTGAGGACTCTGCGGTCTATTACTGTGCAAGATCAGATTACTACGGTAGTAGCTCCCTTGACTACTGG
ACATTGACTGTAGACAAGTCCTCCAGCACAGCCTCATGGAGCTCCGCAGCCTGACATCTGAGGACTCTGCAGTCTATTACTGTGCAAGATCAGATTACTACGGTAGTAGCTCCCTTGACTACTGG
CACATTGACTGTAGACAAGTCCTCCAGCAGCCTACATGGAGCTCCGCAGCCTGACATCTGAGGACTCTGCAGTCTATTACTGTGCAAGATCAGATTACTACGGTAGTAGCTCCCTTGACTACTGG
CCACATTGACTGTAGACAAGTCCAGCACAGCCTACATGGAGCTCCGCAGCCTGACATCTGAGGACTCTGCAGTCTATTACTGTGCAAGATCAGATTACTACGGTAGTAGCTCCCTTGACTACTGG
AATTTCCTGTAGACAAGTCCTCCAGCACAGCCTACATGGAGCTCCGCAGCCTGACATCTGAGGACTCTGCAGTCTATTACTGTGCAAGATCAGATTACTACGGTAGTAGCTCCCTTGACTACTGG
CAGTGCCGGTAGACAAGTCCTCCAGCACAGCCTACATGGAGCTCCGCAGCCTGACATCTGAGGACTCTGCAGTCTATTACTGTGCAAGATCAGATTACTACGGTAGTAGCTCCCTTGACTACTGG
CACATTGAGTAGACAAGTCCTCCAGCACAGCCTACATGGAGCTCCGCAGCCTGACATCTGAGGACTCTGCAGTCTATTACTGTGCAAGATCAGATTACTACGGTAGTAGCTCCCTTGACTACTGG
AATTGACTGTAGACAATTCCTCCAGCACAGCCTACATGGAGCTCAGCAGCCTGACATCTGAGGACTCTGCAGTCTATTACTGTGCAAGATCAGATTACTACGGTAGTAGCTCCCTTGACTACTGG

sample_name	productive_frequency	templates	amino_acid
TX7_Q76_2	78.714	87584	CMRYSNYWYFDVW
TX7_Q76_2	21.188	23575	CAGDYDGYWYFDVW
TX7_Q76_2	0.004	5	CMRYSNYWYFDVW
TX7_Q76_2	0.004	4	CARHYPWFAYW
TX7_Q76_2	0.002	2	CARRDYSNYEDDYW
TX7_Q76_2	0.002	2	CAGDYDGYWYFDVW
TX7_Q76_2	0.002	2	CAGDYDGYWYFDVW
TX7_Q76_2	0.002	2	CMRYSNYWYFDVW
TX7_Q76_2	0.002	2	CAGDYDGYWYFDVW
TX7_Q76_2	0.002	2	CMRYSNYWYFDVW
TX7_Q76_2	0.002	2	CMRYSNYWYFDVW
TX7_Q76_2	0.002	2	CAGDYDGYWYFDVW
TX7_Q76_2	0.002	2	CMRYSNYWYFDVW
TX7_Q76_2	0.002	2	CMRYSNYWYFDVW
TX7_Q76_2	0.002	2	CAGDYDGYWYFDVW
TX7_Q76_2	0.002	2	CMRYSNYWYFDVW
TX7_Q76_2	0.002	2	CMRYSNYWYFDVW
TX7_Q76_2	0.002	2	CMRYSNYWYFDVW
TX7_Q76_2	0.002	2	CMRYSNYWYFDVW
TX7_Q76_2	0.002	2	CARELANWYYFDYW
TX7_Q76_2	0.001	1	CANNRGYAMDYW
TX7_Q76_2	0.001	1	CELRSEDFAYW
TX7_Q76_2	0.001	1	CARGMDYW
TX7_Q76_2	0.001	1	CVLRLVRLRYFDVW
TX7_Q76_2	0.001	1	CAIMDYW
TX7_Q76_2	0.001	1	CARSYGNYVAWFAYW
TX7_Q76_2	0.001	1	CMRYSNYWYFDVW
TX7_Q76_2	0.001	1	CAGDYDGYWYFDVW
TX7_Q76_2	0.001	1	CVVVEDFAYW
TX7_Q76_2	0.001	1	CAGDYDGYWYFDVW
TX7_Q76_2	0.001	1	CAGDYDGYWYFDVW
TX7_Q76_2	0.001	1	CAGDYDGYWYFDVW
TX7_Q76_2	0.001	1	CAGDYDGYWYFDVW
TX7_Q76_2	0.001	1	CAGDYDGYWYFDVW
TX7_Q76_2	0.001	1	CAGDYDGYWYFDVW
TX7_Q76_2	0.001	1	CAGDYDGYWYFDVW
TX7_Q76_2	0.001	1	CMRYSNYWYFDVW
TX7_Q76_2	0.001	1	CMRYSNYWYFDVW
TX7_Q76_2	0.001	1	CMRYSNYWYFDVW
TX7_Q76_2	0.001	1	CMRYSNYWYFDVW
TX7_Q76_2	0.001	1	CMRYSNYWYFDVW
TX7_Q76_2	0.001	1	CARDYGSSYGYFDVW
TX7_Q76_2	0.001	1	CAGDYDGYWYFDVW
TX7_Q76_2	0.001	1	CMRYSNYWYFDVW
TX7_Q76_2	0.001	1	CMRYSNYWYFDVW
TX7_Q76_2	0.001	1	CMRYSNYWYFDVW
TX7_Q76_2	0.001	1	CAGDYDGYWYFDVW
TX7_Q76_2	0.001	1	CAGDYDGYWYFDVW
TX7_Q76_2	0.001	1	CAGDYDGYWYFDVW
TX7_Q76_2	0.001	1	CAGDYDGYWYFDVW
TX7_Q76_2	0.001	1	CAGDYDGYWYFDVW
TX7_Q76_2	0.001	1	CAGDYDGYWYFDVW
TX7_Q76_2	0.001	1	CAGDYDGYWYFDVW
TX7_Q76_2	0.001	1	CMRYSNYWYFDVW
TX7_Q76_2	0.001	1	CMRYSNYWYFDVW
TX7_Q76_2	0.001	1	CMRYSNYWYFDVW
TX7_Q76_2	0.001	1	CMRYSNYWYFDVW
TX7_Q76_2	0.001	1	CMRYSNYWYFDVW
TX7_Q76_2	0.001	1	CMRYSNYWYFDVW
TX7_Q76_2	0.001	1	CMRYSNYWYFDVW
TX7_Q76_2	0.001	1	CMRYSNYWYFDVW
TX7_Q76_2	0.001	1	CMRYSNYWYFDVW
TX7_Q76_2	0.001	1	CMRYSNYWYFDVW
TX7_Q76_2	0.001	1	CAHGYYVLTGW
TX7_Q76_2	0.001	1	CASTYDTGAMDYW
TX7_Q76_2	0.001	1	CARKGGYYYFDYW
TX7_Q76_2	0.001	1	CARDYDYEFAYW
TX7_Q76_2	0.001	1	CGLHACEDFAYW
TX7_Q76_2	0.001	1	CASLQCEDFAYW
TX7_Q76_2	0.001	1	CAKEPPYYGYDGGVFFDYW
TX7_Q76_2	0.001	1	CARRLPFPFAYW
TX7_Q76_2	0.001	1	CMRYSNYWYFDVW
TX7_Q76_2	0.001	1	CDGESEDFAYW
TX7_Q76_2	0.001	1	CMRYSNYWYFDVW
TX7_Q76_2	0.001	1	CMRYSNYWYFDVW
TX7_Q76_2	0.001	1	CAGDYDGYWYFDVW
TX7_Q76_2	0.001	1	CMRYSNYWYFDVW
TX7_Q76_2	0.001	1	CMRYSNYWYFDVW
TX7_Q76_2	0.001	1	CAGDYDGYWYFDVW
TX7_Q76_2	0.001	1	CAGDYDGYWYFDVW
TX7_Q76_2	0.001	1	CAGDYDGYWYFDVW
TX7_Q76_2	0.001	1	CAGDYDGYWYFDVW
TX7_Q76_2	0.001	1	CAGDYDGYWYFDVW
TX7_Q76_2	0.001	1	CMRYSNYWYFDVW
TX7_Q76_2	0.001	1	CMRYSNYWYFDVW
TX7_Q76_2	0.001	1	CMRYSNFRYFDVW
TX7_Q76_2	0.001	1	CAISCHTGEGFAYW
TX7_Q76_2	0.001	1	CSLHACEDFAYW
total	100	111268	

rearrangement

GATTCACTATCTTCAGAGACAATGACAAGAGCACCCTGTACCTGCAGATGAGCAATGTGCGATCGGAGGACACAGCCACGTATTTCTGTATGAGATATAGTAACTACTGGTACTTCGATGTCTGG
TCTCCATTACTAGAGAAACGTCAAAGAACCAGTTCTTCCTCCAATTGAACTCTGTGACCACAGAGGACACAGCCATGTATTACTGTGCAGGAGACTATGATGGTTACTGGTACTTCGATGTCTGG
CGATTCACTATCTTCAGAGACAATGACAAGAGCACCTGTACCTGCAGATGAGCAATGTGCGATCGGAGGACACAGCCACGTATTTCTGTATGAGATATAGTAACTACTGGTACTTCGATGTCTGG
AGGGGCGATTCACCATCTCCAGAGACAATGCCAAGAACACCCTGTACCTGCAAATGAGCAGTCTGAAGTCTGAGGACACAGCCATGTATTACTGTGCAAGACATTACCCCTGGTTTGCTTACTGG
CCACATTGACTGTAGACAAGTCCTCCAGCACAGCCTACATGGAGCTCCGCAGCCTGACATCTGAGGACACTGCAGTCTATTACTGTGCAAGACGAGACTATAGTAACTACGAGGATGACTACTGG
ATCTCCATACTAGAGAAACGTCAAAGAACCAGTTCTTCCTCCAATTGAACTCTGTGACCACAGAGGACACAGCCATGTATTACTGTGCAGGAGACTATGATGGTTACTGGTACTTCGATGTCTGG
ATCTCCATTACTAGAGAAACGTCAAGAACCAGTTCTTCCTCCAATTGAACTCTGTGACCACAGAGGACACAGCCATGTATTACTGTGCAGGAGACTATGATGGTTACTGGTACTTCGATGTCTGG
CGATTCACTATCTCAGAGACAATGACAAGAGCACCCTGTACCTGCAGATGAGCAATGTGCGATCGGAGGACACAGCCACGTATTTCTGTATGAGATATAGTAACTACTGGTACTTCGATGTCTGG
ATCTCCATTACTAGAGAAACGTCAAAGAACAGTTCTTCCTCCAATTGAACTCTGTGACCACAGAGGACACAGCCATGTATTACTGTGCAGGAGACTATGATGGTTACTGGTACTTCGATGTCTGG
CGATCACTATCTTCAGAGACAATGACAAGAGCACCCTGTACCTGCAGATGAGCAATGTGCGATCGGAGGACACAGCCACGTATTTCTGTATGAGATATAGTAACTACTGGTACTTCGATGTCTGG
CGATTCACTATCTTCAGAGACAATGACAGAGCACCCTGTACCTGCAGATGAGCAATGTGCGATCGGAGGACACAGCCACGTATTTCTGTATGAGATATAGTAACTACTGGTACTTCGATGTCTGG
ATCTCATTACTAGAGAAACGTCAAAGAACCAGTTCTTCCTCCAATTGAACTCTGTGACCACAGAGGACACAGCCATGTATTACTGTGCAGGAGACTATGATGGTTACTGGTACTTCGATGTCTGG
CGATTCACTATCTTCAGAGACATGACAAGAGCACCCTGTACCTGCAGATGAGCAATGTGCGATCGGAGGACACAGCCACGTATTTCTGTATGAGATATAGTAACTACTGGTACTTCGATGTCTGG
CGATTCACTATCTTCAGAGACAATGACAAGAGCACCCTGTACCGCAGATGAGCAATGTGCGATCGGAGGACACAGCCACGTATTTCTGTATGAGATATAGTAACTACTGGTACTTCGATGTCTGG
ATCTCCATTACTAGAGAACGTCAAAGAACCAGTTCTTCCTCCAATTGAACTCTGTGACCACAGAGGACACAGCCATGTATTACTGTGCAGGAGACTATGATGGTTACTGGTACTTCGATGTCTGG
CGATTCACTATCTTCAGAGACAATGACAAGAGACCCTGTACCTGCAGATGAGCAATGTGCGATCGGAGGACACAGCCACGTATTTCTGTATGAGATATAGTAACTACTGGTACTTCGATGTCTGG
CGATTCACTATCTTCAGAGACAATGACAAGAGCACCCTGTACCTCAGATGAGCAATGTGCGATCGGAGGACACAGCCACGTATTTCTGTATGAGATATAGTAACTACTGGTACTTCGATGTCTGG
CGATTCACTATCTTCAGAGACAATGAAAGAGCACCCTGTACCTGCAGATGAGCAATGTGCGATCGGAGGACACAGCCACGTATTTCTGTATGAGATATAGTAACTACTGGTACTTCGATGTCTGG
CGATTCACTATCTTCAGAGACAATGACAAGAGCACCCTGTACTGCAGATGAGCAATGTGCGATCGGAGGACACAGCCACGTATTTCTGTATGAGATATAGTAACTACTGGTACTTCGATGTCTGG
CCACACTGACTGTAGACACATCCTCCAGCACAGCCTACATGCAGCTCAGCAGCCTGACATCTGAGGACTCTGCGGTCTATTACTGTGCAAGAGAGCTGGCTAACTGGTACTACTTTGACTACTGG
GCAAGGCCACACTGACGGCAGACACATCCTCCAGCACTGCCTACATGCAGCTCAGCAGCCTAACATCTGAGGACTCTGCGGTCTATTACTGTGCAAACAATCGGGGATATGCTATGGACTACTGG
AGGGCAAGGCCACATTGACTGTAGACAAGTCCTCCAGCACAGCCTACATGGAGCTCCGCAGCCTGACATCTGAGGACACTGCAGTCTATTACTGTGAGCTGCGAAGTGAAGACTTTGCTTACTGG
AGAAGTTCAAGAGCAAGGCCACACTGACTGTAGACAAACCCTCCAGCACAGCCTACATGCAGCTCAGCAGCCTGACATCTGAGGACTCTGCGGTCTATTATTGTGCAAGGGGGATGGACTACTGG
CCACATTGACTGCAAACACATCCTCGAGCACAGCCTACATGGAACTCAGCAGCCTCACATCTGAGGACTCTGCGGTCTATTACTGTGTACTACGGTTGGTCCGTCTTAGGTACTTCGATGTCTGG
ACCAGAAGTTCAAGGGCAAGGCCACATTGACTGTAAACAAGTCCTCCAGCACAGCCTACATGGAGCTCCGCAGCCTGACATCGGAGGATTCTGCAGTCTATTACTGTGCAATTATGGACTACTGG
CCTTCTCTTTGGAAACCTCTGCCAGCACTGCCTATTTGCAGATCAACAACCTCAAAAATGAGGACACGGCTACATATTTCTGTGCAAGATCCTATGGTAACTACGTTGCCTGGTTTGCTTACTGG
CGATTCACTATCTTCAGAGACAATGACAAGAGCACCCGTACCTGCAGATGAGCAATGTGCGATCGGAGGACACAGCCACGTATTTCTGTATGAGATATAGTAACTACTGGTACTTCGATGTCTGG
ATCTCCATTACTAGAGAAACGTCAAAGAACCAGTTCTTCCTCCATTGAACTCTGTGACCACAGAGGACACAGCCATGTATTACTGTGCAGGAGACTATGATGGTTACTGGTACTTCGATGTCTGG
TGAAAGACAGATTCACCATCTCCAGAGATGATTCAGAAAGCATGCTCTATCTGCAAATGAACAACTTGAAAACTGAGGACACAGCCATGTATTACTGTGTGGTTGTTGAAGACTTTGCTTACTGG
ATCTCCATTACTAGAGAAACGTCAAAGAACCAGTTCTCCTCCAATTGAACTCTGTGACCACAGAGGACACAGCCATGTATTACTGTGCAGGAGACTATGATGGTTACTGGTACTTCGATGTCTGG
ATCTCCATTACTAGAGAAACGTCAAAGAACCAGTTCTTCCTCAATTGAACTCTGTGACCACAGAGGACACAGCCATGTATTACTGTGCAGGAGACTATGATGGTTACTGGTACTTCGATGTCTGG
ATCTCCATTACTAGAGAAACGTCAAAGACCAGTTCTTCCTCCAATTGAACTCTGTGACCACAGAGGACACAGCCATGTATTACTGTGCAGGAGACTATGATGGTTACTGGTACTTCGATGTCTGG
CATCTCCATTACTAGAAACGTCAAAGAACCAGTTCTTCCTCCAATTGAACTCTGTGACCACAGAGGACACAGCCATGTATTACTGTGCAGGAGACTATGATGGTTACTGGTACTTCGATGTCTGG
ATCTCCATTCTAGAGAAACGTCAAAGAACCAGTTCTTCCTCCAATTGAACTCTGTGACCACAGAGGACACAGCCATGTATTACTGTGCAGGAGACTATGATGGTTACTGGTACTTCGATGTCTGG
ATCCCATTACTAGAGAAACGTCAAAGAACCAGTTCTTCCTCCAATTGAACTCTGTGACCACAGAGGACACAGCCATGTATTACTGTGCAGGAGACTATGATGGTTACTGGTACTTCGATGTCTGG
ATCTCCATTACTAGAGAAACGTCAAAGAACCAGTTCTTCCCCAATTGAACTCTGTGACCACAGAGGACACAGCCATGTATTACTGTGCAGGAGACTATGATGGTTACTGGTACTTCGATGTCTGG
CGATTCACTATCTTCAGAGACAATGACAAGAGCACCCTGTACCTGAGATGAGCAATGTGCGATCGGAGGACACAGCCACGTATTTCTGTATGAGATATAGTAACTACTGGTACTTCGATGTCTGG
ATTCACTATCTTCAGAGACAATGACAAGAGCACCCCTGTACCTGCAGATGAGCAATGTGCGATCGGAGGACACAGCCACGTATTTCTGTATGAGATATAGTAACTACTGGTACTTCGATGTCTGG
CGATTCACTATCTTAGAGACAATGACAAGAGCACCCTGTACCTGCAGATGAGCAATGTGCGATCGGAGGACACAGCCACGTATTTCTGTATGAGATATAGTAACTACTGGTACTTCGATGTCTGG
TCGATTCACTATCTTCAGACAATGACAAGAGCACCCTGTACCTGCAGATGAGCAATGTGCGATCGGAGGACACAGCCACGTATTTCTGTATGAGATATAGTAACTACTGGTACTTCGATGTCTGG
CGATTCACTATCTTCAGAGAAATGACAAGAGCACCCTGTACCTGCAGATGAGCAATGTGCGATCGGAGGACACAGCCACGTATTTCTGTATGAGATATAGTAACTACTGGTACTTCGATGTCTGG
CACTGACTGTAGACACATCCTCCAGCACAGCCTACATGCAGCTCAGCAGCCTGACATCTGAGGACTCTGCGGTCTATTACTGTGCAAGAGACTACGGTAGTAGCTACGGGTACTTCGATGTCTGG
CTCCATTACTAGAGAAACGTCAAAGAACCAGTTCTTCCTCCAAATTGAACTCTGTGACCACAGAGGACACAGCCATGTATTACTGTGCAGGAGACTATGATGGTTACTGGTACTTCGATGTCTGG
TCGATTCACTATCTTCAGAGACAATGAAGAGCACCCTGTACCTGCAGATGAGCAATGTGCGATCGGAGGACACAGCCACGTATTTCTGTATGAGATATAGTAACTACTGGTACTTCGATGTCTGG
CGATTACTATCTTCAGAGACAATGACAAGAGCACCCTGTACCTGCAGATGAGCAATGTGCGATCGGAGGACACAGCCACGTATTTCTGTATGAGATATAGTAACTACTGGTACTTCGATGTCTGG
CGATTCACTATCTTCAGAGACAAGACAAGAGCACCCTGTACCTGCAGATGAGCAATGTGCGATCGGAGGACACAGCCACGTATTTCTGTATGAGATATAGTAACTACTGGTACTTCGATGTCTGG
ATCTCCATTACTAGAGAAACGTCAAAGAACCAGTTCTTCCTCCAATTAACTCTGTGACCACAGAGGACACAGCCATGTATTACTGTGCAGGAGACTATGATGGTTACTGGTACTTCGATGTCTGG
ATCTCCATTACTAGAGAAACGTCGAGAACCAGTTCTTCCTCCAATTGAACTCTGTGACCACAGAGGACACAGCCATGTATTACTGTGCAGGAGACTATGATGGTTACTGGTACTTCGATGTCTGG
TCTCGATATCTAGAGAAACGTCAAAGAACCAGTTCTTCCTCCAATTGAACTCTGTGACCACAGAGGACACAGCCATGTATTACTGTGCAGGAGACTATGATGGTTACTGGTACTTCGATGTCTGG
ATCTCCTTACTAGAGAAACGTCAAAGAACCAGTTCTTCCTCCAATTGAACTCTGTGACCACAGAGGACACAGCCATGTATTACTGTGCAGGAGACTATGATGGTTACTGGTACTTCGATGTCTGG
ATCTCCATTACAGAGAAACGTCAAAGAACCAGTTCTTCCTCCAATTGAACTCTGTGACCACAGAGGACACAGCCATGTATTACTGTGCAGGAGACTATGATGGTTACTGGTACTTCGATGTCTGG
CTCCATTACTAGAGAAAACGTCAAAGAACCAGTTCTTCCTCCAATTGAACTCTGTGACCACAGAGGACACAGCCATGTATTACTGTGCAGGAGACTATGATGGTTACTGGTACTTCGATGTCTGG
ATCTCCATTACTAGAGAAACGTCAAAGAACCAGTTCTTCCTCCAATGAACTCTGTGACCACAGAGGACACAGCCATGTATTACTGTGCAGGAGACTATGATGGTTACTGGTACTTCGATGTCTGG
TCGATTCACTATCTTCAGAGACAATGACAAGAGCACCCTGTACCTGGATGAGCAATGTGCGATCGGAGGACACAGCCACGTATTTCTGTATGAGATATAGTAACTACTGGTACTTCGATGTCTGG
CGATTCACTATCTTCAGAGACAATACAAGAGCACCCTGTACCTGCAGATGAGCAATGTGCGATCGGAGGACACAGCCACGTATTTCTGTATGAGATATAGTAACTACTGGTACTTCGATGTCTGG
CGATTCACTATCTTCGAGACAATGACAAGAGCACCCTGTACCTGCAGATGAGCAATGTGCGATCGGAGGACACAGCCACGTATTTCTGTATGAGATATAGTAACTACTGGTACTTCGATGTCTGG
GATTGAGTCTCTTCAGAGACAATGACAAGAGCACCCTGTACCTGCAGATGAGCAATGTGCGATCGGAGGACACAGCCACGTATTTCTGTATGAGATATAGTAACTACTGGTACTTCGATGTCTGG
TTTTATCTATCTTCAGAGACAATGACAAGAGCACCCTGTACCTGCAGATGAGCAATGTGCGATCGGAGGACACAGCCACGTATTTCTGTATGAGATATAGTAACTACTGGTACTTCGATGTCTGG
GCTTCAAGATCTTCAGAGACAATGACAAGAGCACCCTGTACCTGCAGATGAGCAATGTGCGATCGGAGGACACAGCCACGTATTTCTGTATGAGATATAGTAACTACTGGTACTTCGATGTCTGG
GATACAAAATCTTCAGAGACAATGACAAGAGCACCCTGTACCTGCAGATGAGCAATGTGCGATCGGAGGACACAGCCACGTATTTCTGTATGAGATATAGTAACTACTGGTACTTCGATGTCTGG
CGATTCAGTATCTTCAGAGACAATGACAAGAGCACCCGTACCTGCAGATGAGCAATGTGCGATCGGAGGACACAGCCACGTATTTCTGTATGAGATATAGTAACTACTGGTACTTCGATGTCTGG
TCGATTCACTATCTTCAGAGACAATGACAAGAGCACCGTACCTGCAGATGAGCAATGTGCGATCGGAGGACACAGCCACGTATTTCTGTATGAGATATAGTAACTACTGGTACTTCGATGTCTGG
CGATTCACTATCTTCAGAGACAATGACAAGAGCACCCTGACCTGCAGATGAGCAATGTGCGATCGGAGGACACAGCCACGTATTTCTGTATGAGATATAGTAACTACTGGTACTTCGATGTCTGG
AGGGCCGATTCACCATCTCCAGAGACAATGCCAAGAACACCCTGTTCCTGCAAATGACCAGTCTGAGGTCTGAGGACACGGCCATGTATTACTGTGCGCATGGTTACTACGTTTTAACGGGCTGG
AGGCCACATTGACTGTAGACAAATCCTCCAGCACAGCCTACATGCAGCTCAAGAGCCTGACATCTGAGGACTCTGCAGTCTATTACTGTGCAAGCACCTATGATACGGGGGCTATGGACTACTGG
GACTGAGCATCAGCAAAGACAACTCCAAGAGTCAAGTTTTCTTAAAAATGAACAGTCTGCAAACTGATGACACAGCCAGGTACTACTGTGCCAGAAAGGGTGGTTACTACTACTTTGACTACTGG
ATCGAATCTCCATCACTCGTGACACATCTAAGAACCAGTTTTTCCTGAAGTTGAATTCTGTGACTACTGAGGACACAGCCACATATTACTGTGCAAGGGACTATGATTACGAGTTTGCTTACTGG
GCAAGGCCACACTTACTGTAGACAAATCCTCCAGCACAGCCTACATGTTGCTCAGCAGCCTGACCTCTGAGGACTCTGCGGTCTATTTCTGTGGTCTGCATGCCTGTGAAGACTTTGCTTACTGG
ATAAATTCATCATCTCCAGAGACAACGCCAAAAATACGCTGTACCTGCAAATGAGCAAAGTGAGATCTGAGGACACAGCCCTTTATTACTGTGCAAGCCTTCAGTGTGAAGACTTTGCTTACTGG
ACACATCCTCCAGCACTGCCTACATGCAGCTCAGCAGCCTAACATCTGAGGACTCTGCGGTCTATTACTGTGCAAAAGAGCCCCCGTACTATGGTTACGACGGGGGGGTATTTTTTGACTACTGG
GCAAGGCCACATTGACTGTAGACAAGTCCTCCAGCACAGCCTACATGGAGCTCCGCAGCCTGACATCTGAGGACACTGCAGTCTATTACTGTGCAAGAAGGTTACCATTTCCGTTTGCTTACTGG
TATTAAATATCTTCAGAGACAATGACAAGAGCACCCTGTACCTGCAGATGAGCAATGTGCGATCGGAGGACACAGCCACGTATTTCTGTATGAGATATAGTAACTACTGGTACTTCGATGTCTGG
AAGGCAAGGCCACATTTACTGTAGACACATCCTCCAACACAGCCTACATGCAACTCAGAAGCCTGACATCTGAGGACACTGCTGACTATTATTGTGACGGGGAAAGTGAAGACTTTGCTTACTGG
GATTAAGTTTCTTCAGAGACAATGACAAGAGCACCCTGTACCTGCAGATGAGCAATGTGCGATCGGAGGACACAGCCACGTATTTCTGTATGAGATATAGTAACTACTGGTACTTCGATGTCTGG
GATTACATATCTTCAGAGACAATGACAAGAGCACCCTGTACCTGCAGATGAGCAATGTGCGATCGGAGGACACAGCCACGTATTTCTGTATGAGATATAGTAACTACTGGTACTTCGATGTCTGG
ATCTCCATTACTAGAGAAACGTCAAAGAACCAGTTCTTCTCCAATTGAACTCTGTGACCACAGAGGACACAGCCATGTATTACTGTGCAGGAGACTATGATGGTTACTGGTACTTCGATGTCTGG
GATTCTCATTCTTCAGAGACAATGACAAGAGCACCCTGTACCTGCAGATGAGCAATGTGCGATCGGAGGACACAGCCACGTATTTCTGTATGAGATATAGTAACTACTGGTACTTCGATGTCTGG
ATTCACTATCTTCAGAGACAAATGACAAGAGCACCCTGTACCTGCAGATGAGCAATGTGCGATCGGAGGACACAGCCACGTATTTCTGTATGAGATATAGTAACTACTGGTACTTCGATGTCTGG
GCTCATTTACTAGAGAAACGTCAAAGAACCAGTTCTTCCTCCAATTGAACTCTGTGACCACAGAGGACACAGCCATGTATTACTGTGCAGGAGACTATGATGGTTACTGGTACTTCGATGTCTGG
TGAGCATTACTAGAGAAACGTCAAAGAACCAGTTCTTCCTCCAATTGAACTCTGTGACCACAGAGGACACAGCCATGTATTACTGTGCAGGAGACTATGATGGTTACTGGTACTTCGATGTCTGG
TCTACTATACTAGAGAAACGTCAAAGAACCAGTTCTTCCTCCAATTGAACTCTGTGACCACAGAGGACACAGCCATGTATTACTGTGCAGGAGACTATGATGGTTACTGGTACTTCGATGTCTGG
TCTCATATACTAGAGAAACGTCAAAGAACCAGTTCTTCCTCCAATTGAACTCTGTGACCACAGAGGACACAGCCATGTATTACTGTGCAGGAGACTATGATGGTTACTGGTACTTCGATGTCTGG
TCGACAATACTAGAGAAACGTCAAAGAACCAGTTCTTCCTCCAATTGAACTCTGTGACCACAGAGGACACAGCCATGTATTACTGTGCAGGAGACTATGATGGTTACTGGTACTTCGATGTCTGG
CTTTCACTATCTTCAGAGACAATGACAAGAGCACCCTGTACCTGCAGATGAGCAATGTGCGATCGGAGGACACAGCCACGTATTTCTGTATGAGGTATAGTAACTACTGGTACTTCGATGTCTGG
GAGTCAGTTTCTTCAGAGACAATGACAAGAGCACCCTGTACCTGCAGATGAGCAATGTGCGATCGGAGGACACAGCCACGTATTTCTGTATGAGATATAGTAACTACTGGTACTTCGATGTCTGG
TATTCACTATCTTCAGAGACAATGACAAGAGCACCCTGTACCTGCAGATGAGCAATGTGCGATCGGAGGACACAGCCACGTATTTCTGTATGAGATATAGTAACTTCCGGTACTTCGATGTCTGG
GATTCATCTCCAGAGACAATGCCAAGAACACCCTGTACCTGCAAATGAGCAGTCTGAAGTCTGAGGACACAGCCTTGTATTACTGTGCAATATCTTGCCACACAGGTGAAGGCTTTGCTTACTGG
GCAAGGCCACACTTACTGTAGACAAATCCTCCAGCACAGCCTACATGTTGCTCAGCAGCCTGACCTCTGAGGACTCTGCGGTCTATTTCTGTAGTCTGCATGCCTGTGAAGACTTTGCTTACTGG

sample_name	productive_frequency	templates	amino_acid	rearrangement
TX3_R62_2	99.217	80035	CMRYSNYWYFDVW	GATTCACTATCTTCAGAGACAATGACAAGAGCACCCTGTACCTGCAGATGAGCAATGTGCGATCGGAGGACACAGCCACGTATTTCTGTATGAGATATAGTAACTACTGGTACTTCGATGTCTGG
TX3_R62_2	0.699	564	CAGDYDGYWYFDVW	TCTCCATTACTAGAGAAACGTCAAAGAACCAGTTCTTCCTCCAATTGAACTCTGTGACCACAGAGGACACAGCCATGTATTACTGTGCAGGAGACTATGATGGTTACTGGTACTTCGATGTCTGG
TX3_R62_2	0.006	5	CMRYSNYWYFDVW	CGATTCACTATCTTCAGAGACAATGACAAGAGCACCTGTACCTGCAGATGAGCAATGTGCGATCGGAGGACACAGCCACGTATTTCTGTATGAGATATAGTAACTACTGGTACTTCGATGTCTGG
TX3_R62_2	0.002	2	CARGGGYDGFAYW	AGGCCACATTGACTGTAGACAAGTCCTCCAGCACAGCCTACATGGAGCTCCGCAGCCTGACATCTGAGGACTCTGCAGTCTATTACTGTGCAAGAGGGGGAGGTTACGACGGGTTTGCTTACTGG
TX3_R62_2	0.002	2	CMRYSNYWYFDVW	CGATTCACTATCTTCAGAGACATGACAAGAGCACCCTGTACCTGCAGATGAGCAATGTGCGATCGGAGGACACAGCCACGTATTTCTGTATGAGATATAGTAACTACTGGTACTTCGATGTCTGG
TX3_R62_2	0.002	2	CMRYSNYWYFDVW	CGATTCACTATCTTCAGAGACAATGACAGAGCACCCTGTACCTGCAGATGAGCAATGTGCGATCGGAGGACACAGCCACGTATTTCTGTATGAGATATAGTAACTACTGGTACTTCGATGTCTGG
TX3_R62_2	0.002	2	CMRYSNYWYFDVW	CGATCACTATCTTCAGAGACAATGACAAGAGCACCCTGTACCTGCAGATGAGCAATGTGCGATCGGAGGACACAGCCACGTATTTCTGTATGAGATATAGTAACTACTGGTACTTCGATGTCTGG
TX3_R62_2	0.002	2	CMRYSNYWYFDVW	CGATTCACTATCTTCAGAGAAATGACAAGAGCACCCTGTACCTGCAGATGAGCAATGTGCGATCGGAGGACACAGCCACGTATTTCTGTATGAGATATAGTAACTACTGGTACTTCGATGTCTGG
TX3_R62_2	0.002	2	CMRYSNYWYFDVW	CGATTCACATCTTCAGAGACAATGACAAGAGCACCCTGTACCTGCAGATGAGCAATGTGCGATCGGAGGACACAGCCACGTATTTCTGTATGAGATATAGTAACTACTGGTACTTCGATGTCTGG
TX3_R62_2	0.002	2	CMRYSNYWYFDVW	CGATTCACTATCTTCAGAGACAATGACAAGAGCACCCGTACCTGCAGATGAGCAATGTGCGATCGGAGGACACAGCCACGTATTTCTGTATGAGATATAGTAACTACTGGTACTTCGATGTCTGG
TX3_R62_2	0.002	2	CMRYSNYWYFDVW	CGATTCACTATCTTCAGAGACAATGACAAGAGCACCCTGTACCTGAGATGAGCAATGTGCGATCGGAGGACACAGCCACGTATTTCTGTATGAGATATAGTAACTACTGGTACTTCGATGTCTGG
TX3_R62_2	0.002	2	CMRYSNYWYFDVW	CGATTCACTATCTTCAGAGACAATGACAAGAGCACCCTGTACCTCAGATGAGCAATGTGCGATCGGAGGACACAGCCACGTATTTCTGTATGAGATATAGTAACTACTGGTACTTCGATGTCTGG
TX3_R62_2	0.002	2	CMRYSNYWYFDVW	ATTCACTATCTTCAGAGACAATGACAAGAGCACCCCTGTACCTGCAGATGAGCAATGTGCGATCGGAGGACACAGCCACGTATTTCTGTATGAGATATAGTAACTACTGGTACTTCGATGTCTGG
TX3_R62_2	0.002	2	CMRYSNYWYFDVW	CGATTCACTATCTTCAGAGACAATGAAAGAGCACCCTGTACCTGCAGATGAGCAATGTGCGATCGGAGGACACAGCCACGTATTTCTGTATGAGATATAGTAACTACTGGTACTTCGATGTCTGG
TX3_R62_2	0.002	2	CMRYSNYWYFDVW	TCGATTCACTATCTTCAGAGACAATGACAAGAGCACCCTACCTGCAGATGAGCAATGTGCGATCGGAGGACACAGCCACGTATTTCTGTATGAGATATAGTAACTACTGGTACTTCGATGTCTGG
TX3_R62_2	0.002	2	CMRYSNYWYFDVW	CGATTCACTATCTTCAGAGACAATGACAAGAGACCCTGTACCTGCAGATGAGCAATGTGCGATCGGAGGACACAGCCACGTATTTCTGTATGAGATATAGTAACTACTGGTACTTCGATGTCTGG
TX3_R62_2	0.002	2	CMRYSNYWYFDVW	CGATTCACTATCTCAGAGACAATGACAAGAGCACCCTGTACCTGCAGATGAGCAATGTGCGATCGGAGGACACAGCCACGTATTTCTGTATGAGATATAGTAACTACTGGTACTTCGATGTCTGG
TX3_R62_2	0.002	2	CMRYSNYWYFDVW	CGATTCACTATCTTCAGAGACAATGACAAGAGCACCCTGTACTGCAGATGAGCAATGTGCGATCGGAGGACACAGCCACGTATTTCTGTATGAGATATAGTAACTACTGGTACTTCGATGTCTGG
TX3_R62_2	0.002	2	CMRYSNYWYFDVW	CGATTCACTATCTTCAGAGACAATGACAAGAGCACCCTGTACCGCAGATGAGCAATGTGCGATCGGAGGACACAGCCACGTATTTCTGTATGAGATATAGTAACTACTGGTACTTCGATGTCTGG
TX3_R62_2	0.001	1	CARWSVQSVRYFDVW	TCATCTCCAGAGACAACGCCAAAAATACGCTGTACCTGCAAATGAGCAAAGTGAGATCTGAGGACACAGCCCTTTATTACTGTGCACGCTGGAGTGTTCAGTCCGTTAGGTACTTCGATGTCTGG
TX3_R62_2	0.001	1	CTLRGAGEDFAYW	GATTCACCATCTCCAGAGACAATGCCAAGAACACCCTGTACCTGCAAATGAGCAGTCTGAAGTCTGAGGACACAGCCATGTATTACTGTACACTCCGTGGTGCTGGTGAAGACTTTGCTTACTGG
TX3_R62_2	0.001	1	CAGYVPRLRYFDVW	TCGCAGTCTCCAAAGACACCTCCAACAACCAAGCATTCCTGAATATCATCACTGTGGAAACTGCAGATACTGCCATATACTACTGTGCTGGTTACGTACCCCGTCTTAGGTACTTCGATGTCTGG
TX3_R62_2	0.001	1	CMRYSNYWYFDVW	CGATTCACTATCTTAGAGACAATGACAAGAGCACCCTGTACCTGCAGATGAGCAATGTGCGATCGGAGGACACAGCCACGTATTTCTGTATGAGATATAGTAACTACTGGTACTTCGATGTCTGG
TX3_R62_2	0.001	1	CMRYSNYWYFDVW	TCGATTCACTATCTTCAGACAATGACAAGAGCACCCTGTACCTGCAGATGAGCAATGTGCGATCGGAGGACACAGCCACGTATTTCTGTATGAGATATAGTAACTACTGGTACTTCGATGTCTGG
TX3_R62_2	0.001	1	CMRYSNYWYFDVW	GTTTCCATATCTTCAGAGACAATGACAAGAGCACCCTGTACCTGCAGATGAGCAATGTGCGATCGGAGGACACAGCCACGTATTTCTGTATGAGATATAGTAACTACTGGTACTTCGATGTCTGG
TX3_R62_2	0.001	1	CMRYSNYWYFDVW	CGATTCACTATCTTCAGAGACAATGACAAGAGCACCCTGTCCTGCAGATGAGCAATGTGCGATCGGAGGACACAGCCACGTATTTCTGTATGAGATATAGTAACTACTGGTACTTCGATGTCTGG
TX3_R62_2	0.001	1	CMRYSNYWYFDVW	GATTCAGATTCTTCAGAGACAATGACAAGAGCACCCTGTACCTGCAGATGAGCAATGTGCGATCGGAGGACACAGCCACGTATTTCTGTATGAGATATAGTAACTACTGGTACTTCGATGTCTGG
TX3_R62_2	0.001	1	CMRYSNYWYFDVW	ATTCACTATCTTCAGAGACAAATGACAAGAGCACCCTGTACCTGCAGATGAGCAATGTGCGATCGGAGGACACAGCCACGTATTTCTGTATGAGATATAGTAACTACTGGTACTTCGATGTCTGG
TX3_R62_2	0.001	1	CMRYSNYWYFDVW	TAGTCCCTATCTTCAGAGACAATGACAAGAGCACCCTGTACCTGCAGATGAGCAATGTGCGATCGGAGGACACAGCCACGTATTTCTGTATGAGATATAGTAACTACTGGTACTTCGATGTCTGG
TX3_R62_2	0.001	1	CMRYSNYWYFDVW	CGATTCACTATCTTCAGAGACAATGACAAGAGCACCCTGACCTGCAGATGAGCAATGTGCGATCGGAGGACACAGCCACGTATTTCTGTATGAGATATAGTAACTACTGGTACTTCGATGTCTGG
TX3_R62_2	0.001	1	CAGDYDGYWYFDVW	ATCTCCATTACTAGAGAAACGTCAAGAACCAGTTCTTCCTCCAATTGAACTCTGTGACCACAGAGGACACAGCCATGTATTACTGTGCAGGAGACTATGATGGTTACTGGTACTTCGATGTCTGG
TX3_R62_2	0.001	1	CMRYSNYWYFDVW	TCGATTCACTATCTTCAGAGACAATGACAAGAGCACCCTGTACCTGGATGAGCAATGTGCGATCGGAGGACACAGCCACGTATTTCTGTATGAGATATAGTAACTACTGGTACTTCGATGTCTGG
TX3_R62_2	0.001	1	CMRYSNYWYFDVW	CGATTACTATCTTCAGAGACAATGACAAGAGCACCCTGTACCTGCAGATGAGCAATGTGCGATCGGAGGACACAGCCACGTATTTCTGTATGAGATATAGTAACTACTGGTACTTCGATGTCTGG
TX3_R62_2	0.001	1	CMRYSNYWYFDVW	TCGATTCACTATCTTCAGAGACAATGACAAGAGCACCCTGTACCCAGATGAGCAATGTGCGATCGGAGGACACAGCCACGTATTTCTGTATGAGATATAGTAACTACTGGTACTTCGATGTCTGG
TX3_R62_2	0.001	1	CWLKGCEDFAYW	GCAAGGCCACATTGACTGTAGACAAGTCCTCCAGCACAGCCTACATGGAGCTCCGCAGCCTGACATCTGAGGACTCTGCAGTCTATTACTGTTGGTTGAAAGGATGTGAAGACTTTGCTTACTGG
TX3_R62_2	0.001	1	CARPFPDPFRYFDVW	GCATCAGCAAGGACAACTCCAAGAGCCAAATTTTCTTTAAAATGAACAGTCTGCAAGCTGATGACACTGCCATATACTACTGTGCCCGACCCTTTCCAGACCCGTTTAGGTACTTCGATGTCTGG
TX3_R62_2	0.001	1	CTAPNHRLRYFDVW	CCACATTCTCTGTAGACCGGTCCTCCAGCACAGTGTACATGGTGTTGAACAGCCTGACATCTGAGGACCCTGCTGTCTATTACTGTACTGCGCCCAATCACCGTCTTAGGTACTTCGATGTCTGG
TX3_R62_2	0.001	1	SRVRREDFAYW	AGGGCAAGGCCACATTGACTGTCGACAATACCTCCAGCACAGCCTACATGGAGCTCGGCAGCCTGACTTCTGAGGACTCTGCGGTCTATTACTCTAGGGTCAGGCGTGAAGACTTTGCTTACTGG
TX3_R62_2	0.001	1	VFQPHSRLRYFDVW	TTGCCTTCTCTTTGGAAACCTCTGCCAGCACTGCCTATTTGCAGATCAAAACCTCAAAAATGAGGACACGGCTACATATTTCTGTATTCCAACCGCATTCACGTCTTAGGTACTTCGATGTCTGG
TX3_R62_2	0.001	1	CALKPTREDFAYW	GATTCATCGTCTCAAGAGATGATTCCAAAAGCAGTGCATACATGCAGATGAACAGCTTAAGAAAGGAAGACACTGCCGTTTATTACTGTGCATTAAAGCCAACACGTGAAGACTTTGCTTACTGG
TX3_R62_2	0.001	1	CPNSEDFAYW	TCAAGGCCAAGGCCACACTGACTGCAGACAAATCCTCCAGCACAGCCTATATGGAGCTTAGTAGATTGACATCTGAGGACTCTGCAGTCTAATACTGTCCGAATAGTGAAGACTTTGCTTACTGG
TX3_R62_2	0.001	1	CMRYSNYWYFDVW	CGATTCACTATCTTCAGAGACAAGACAAGAGCACCCTGTACCTGCAGATGAGCAATGTGCGATCGGAGGACACAGCCACGTATTTCTGTATGAGATATAGTAACTACTGGTACTTCGATGTCTGG
TX3_R62_2	0.001	1	CMRYSNYWYFDVW	TCGATTCACTATCTTGAGACAATGACAAGAGCACCCTGTACCTGCAGATGAGCAATGTGCGATCGGAGGACACAGCCACGTATTTCTGTATGAGATATAGTAACTACTGGTACTTCGATGTCTGG
TX3_R62_2	0.001	1	CMRYSNYWYFDVW	TCGATTCTATCTTCAGAGACAATGACAAGAGCACCCTGTACCTGCAGATGAGCAATGTGCGATCGGAGGACACAGCCACGTATTTCTGTATGAGATATAGTAACTACTGGTACTTCGATGTCTGG
TX3_R62_2	0.001	1	CMRYSNYWYFDVW	TCGAATCACTATCTTCAGACAATGACAAGAGCACCCTGTACCTGCAGATGAGCAATGTGCGATCGGAGGACACAGCCACGTATTTCTGTATGAGATATAGTAACTACTGGTACTTCGATGTCTGG
TX3_R62_2	0.001	1	SRVRREDFAYW	CGGGCAAGGCCACATTGACTGTCGACAATACCTCCAGCACAGCCTACATGGAGCTCGGCAGCCTGACTTCTGAGGACTCTGCGGTCTATTACTCTAGGGTCAGGCGTGAAGACTTTGCTTACTGG
TX3_R62_2	0.001	1	CMRYSNYWYFDVW	CGATTCACTATCTTCAGAGACAATTACAGAGCACCCTGTACCTGCAGATGAGCAATGTGCGATCGGAGGACACAGCCACGTATTTCTGTATGAGATATAGTAACTACTGGTACTTCGATGTCTGG
TX3_R62_2	0.001	1	CMRYSNYWYFDVW	CGATTCACTATCTTCAGAGACAATACAAGAGCACCCTGTACCTGCAGATGAGCAATGTGCGATCGGAGGACACAGCCACGTATTTCTGTATGAGATATAGTAACTACTGGTACTTCGATGTCTGG
TX3_R62_2	0.001	1	CMRYSNYWYFDVW	TCGATTCACTATCTAGAGACAATGACAAGAGCACCCTGTACCTGCAGATGAGCAATGTGCGATCGGAGGACACAGCCACGTATTTCTGTATGAGATATAGTAACTACTGGTACTTCGATGTCTGG
TX3_R62_2	0.001	1	CMRYSNYWYFDVW	TATTCAGATTCTTCAGAGACAATGACAAGAGCACCCTGTACCTGCAGATGAGCAATGTGCGATCGGAGGACACAGCCACGTATTTCTGTATGAGATATAGTAACTACTGGTACTTCGATGTCTGG
total	100.000	80667		
