# Supplementary material for: Exome sequencing of the TCL1 mouse model for CLL reveals genetic heterogeneity and dynamics during disease development
Source: Leukemia. 2018 Sep 27;33(4):957–68. doi: 10.1038/s41375-018-0260-4 (PMC6477797; doi:10.1038/s41375-018-0260-4)
Supplement: Supplementary file 11 — table S5 [file 41375_2018_260_MOESM11_ESM.pdf]

Mouse ID	Genotype	Survival (days)	Sex	Phenotype
347	TCL1tg	368	m	Splenomegalie, liver infiltration
212	TCL1tg	342	m	Splenomegalie
221	TCL1tg	388	m	Splenomegalie
E31	TCL1tg	388	f	Splenomegalie, liver infiltration
C25	TCL1tg	435	f	Splenomegalie, liver infiltration, enlarged LN mesenterial
D22	TCL1tg	434	m	Splenomegalie, liver infiltration
F3	TCL1tg	245	m	Splenomegalie, liver infiltration, enlarged LN inguinal
R62	3x TX (D22)	49	m	Splenomegalie, enlarged LN inguinal, mucosa infiltration
Q76	7x TX (D22)	34	m	Splenomegalie, liver infiltration, enlarged LN inguinal, subdermal infiltration, mucosa infiltration
Q67	6x TX (C25)	35	f	Splenomegalie, liver infiltration
Q82	2x TX (E31)	70	f	Splenomegalie, liver infiltration
