# Supplementary material for: Exome sequencing of the TCL1 mouse model for CLL reveals genetic heterogeneity and dynamics during disease development
Source: Leukemia. 2018 Sep 27;33(4):957–68. doi: 10.1038/s41375-018-0260-4 (PMC6477797; doi:10.1038/s41375-018-0260-4)
Supplement: Supplementary file 16 — protocol exchange [file 41375_2018_260_MOESM16_ESM.pdf]

NGS-based analysis of the mouse B-cell receptor repertoire

Nadja Zaborsky, Maria Schubert, Franz J. Gassner & Roland Geisberger

SCRI-LIMCR - Salzburg Cancer Research Institute - Laboratory for Immunological and Molecular Cancer Research

Protocol Exchange

Abstract

The B cell receptor (BCR), especially the complementarity-determining region 3 (CDR3) encoded on the variable region of the immunoglobulin heavy chain, is the most important region that determines antigen binding and specificity. Furthermore, sequence analysis of the CDR3 region is important for understanding the clonal distribution and dynamics in the clonal evolution of leukemic cells.
Here we present a sensitive method for fast and precise analysis of the BCR repertoire of mouse chronic lymphocytic leukemia cells by amplification and high-throughput DNA sequencing of the genomic rearranged VDJ locus. Tagging and indexing of the PCR amplified VDJ locus enables multiplexing and Illumina sequencing of up to 3×96 samples (depending on the requested coverage) in parallel on a MiSeq system, with a total coverage of 25 million reads for a high output run. The protocol includes a suggestion for fast bioinformatics analysis using the MiXCR software.

Subject terms:

Genetic analysis
Genomics
Immunological techniques
Nucleic acid based molecular biology

Keywords:

Mouse
PCR
BCR
High-throughput sequencing

<u>Introduction</u>

In the context of cancer, analysis of the BCR repertoire in mice becomes increasingly important to define clones and subclones within a tumor population. There are several approaches for this analysis, e.g. by spectratyping, but high-throughput sequencing is the state-of-the-art nowadays. So far, there are no manufacturers kits for mouse BCR repertoire analysis by NGS available. Therefore, we developed an in-lab protocol with self-designed

degenerated primers for amplification of the rearranged VDJ locus of the immunoglobulin heavy chain using genomic DNA as input material. With this protocol, up to 3×96 mouse samples can be multiplexed and subjected to Illumina sequencing in a fast and cost-saving way.

Reagents

PCR amplification

• Phusion High-Fidelity PCR Master Mix with HF Buffer (Thermo Fisher, cat. no. F531L)
• 2x KAPA HiFi HotStart ReadyMix (Roche, cat. no. 07958927001)
• PCR primer for amplification (untagged, tagged) see Table 1 (Eurofins Genomics; Stock concentration: 100 µmol/L)
• Nextera XT Index 1 Primers (N7XX) and Nextera XT Index 2 Primers (S5XX) from the Nextera XT Index kit (Illumina, cat. no. FC-131-1002) for multiplexing of 96 samples
• Nuclease-Free Water (Qiagen, cat. no. 129114)

Purification of reactions

• AMPure XP beads (Beckman Coulter, cat. no. A63881)
• 1 M Tris-HCl, pH 8.5, sterile (Teknava, cat. no. T1085)
• Ethanol absolute for analysis EMSURE ACS,ISO,Reag. Ph Eur (Merck, cat. no. MC1009831000)
• Nuclease-Free Water (Qiagen, cat. no. 129114)

Determination of DNA quantity and quality

• Qubit dsDNA HS Assay Kit (Thermo Fisher, cat. no. Q32851)
• D1000 ScreenTape (Agilent, cat. no. 5067-5582)
• D1000 Reagents (Agilent, cat. no. 5067-5583)
• D1000 Ladder (Agilent, cat. no. 5067-5586)
• High Sensitivity D1000 ScreenTape (Agilent, cat. no. 5067-5584)
• High Sensitivity D1000 Reagents (Agilent, cat. no. 5067-5585)
• High Sensitivity D1000 Ladder (Agilent, cat. no. 5067-5587)

Illumina Sequencing

• MiSeq Reagent Kit v3 (600-cycle) (Illumina, cat. no. MS-102-3003)
• 0.2 N NaOH (freshly prepared)

REAGENT SETUP

80% ethanol

Prepare an 80% ethanol solution (vol/vol) with nuclease-free water freshly for each purification reaction by mixing 1 part ethanol absolute for analysis and 4 parts nuclease-free water.

10 mM Tris-HCl (pH 8.5)

Prepare a 10 mM Tris-HCl (pH 8.5) solution by adding 100 µl of a 1 M Tris-HCl (pH 8.5) stock solution to 9.9 ml nuclease-free water.

Table 1: Primer sequences for PCR amplification of the VDJ region of the B-cell receptor

Step	Primer	Sequence (5'- 3')	Purpose
7	RG1269	SAGRTBCARCTKMARSAGYCWGGVSCT	Untagged forward primer 1 binding in FR1 of IGHV
7	RG1270	GARGTGMAGCTKGWDGAGWCTGGDGGA	Untagged forward primer 2 binding in FR1 of IGHV
7	RG1271	CTGMRGARACDGTGASHVDRGTBCCTK	Untagged reverse primer binding in IGHJ
22	RG1272	TCGTCGGCAGCGTCAGATGTGTATAAGAGACAG SAGRTBCARCTKMARSAGYCWGGVSCT	Tagged forward primer 1 binding in FR1 of IGHV
22	RG1273	TCGTCGGCAGCGTCAGATGTGTATAAGAGACAG GARGTGMAGCTKGWDGAGWCTGGDGGA	Tagged forward primer 2 binding in FR1 of IGHV
22	RG1274	GTCTCGTGGGCTCGGAGATGTGTATAAGAGACAG CTGMRGARACDGTGASHVDRGTBCCTK	Tagged reverse primer binding in IGHJ

Equipment

- Thermocycler T3000 (Biometra)
- DynaMag-96 Side Magnet (Thermo Fisher; cat. no. 12331D)
- NanoDrop 2000c Spectrophotometers (Thermo Fisher)
- Qubit 2.0 Fluorometer (Invitrogen, cat. no. Q32866)
- 4200 TapeStation system (Agilent, cat. no. G2991AA)
- MiSeqDx (Illumina)
- Eppendorf microcentrifuge 5424 (Eppendorf, cat. no. 022620444)
- MiniStar silverline microcentrifuge (VWR, cat. no. 521-2844)
- Loading Tips for Agilent 4200 TapeStation system (Agilent, cat. no. 5067-5599)
- 1000 µl Filter Tip (Sterile), racked (Starlab, cat. no. S1126-7810)
- 200 µl Graduated Filter Tip (Sterile), Racked (Starlab, cat. no. S1120-8810)
- 100 µl Bevelled Filter Tip (Sterile), Racked (Starlab, cat. no. S1120-1840)
- 10/20 µl XL Graduated Filter Tip (Sterile), Racked (Starlab, cat. no. S1120-3810)
- PCR Tubes, 0.2 ml 8-Strip, natural (Starlab, cat. no. I1402-3500)
- PCR caps, 8-Strip Caps, Domed (Starlab, cat. no. I1400-0800)

• DNA LoBind Tubes, 1.5 mL (Eppendorf, cat. no. 0030108051)
• 0.5ml PCR Tubes (Promega, cat. no. E4941)
• Falcon Centrifuge Tubes, Polypropylene, Sterile, 50 ml (BD Falcon, cat. no. 352070)
• Falcon Centrifuge Tubes, Polypropylene, Sterile, 15 ml (BD Falcon, cat. no. 352097)

• Falcon Centrifuge Tubes, Polypropylene, Sterile, 15 ml (BD Falcon, cat. no. 352097)

<u>**Procedure**</u>

Primer design for amplification of the VDJ region of the B-cell receptor

1| Nucleotide sequences of the mouse IGHV and the IGHJ region can be obtained from the IMTG genome database (http://www.imgt.org/vquest/refseqh.html#VQUEST)
2| Perform a sequences alignment of each gene segment (IGHV and IGHJ) and create a frequency plot with WebLogo (https://weblogo.berkeley.edu/logo.cgi)
3| Primer sequences were obtained based on frequencies of single bases within the aligned sequence using the UIB code for degenerated primers (see Figure 1)
(i) For the IGHV gene segment the sequence alignment of the framework region 1 (FR1) clustered into 2 groups, hence 2 different degenerated forward primers were generated (see Table 1, RG1269 and RG1270)
(ii) For the IGHJ gene segment 1 degenerated reverse primer was generated (see Table 1, RG1271)

Figure 1: Schematic representation of VDJ recombination and primer design for amplification of the VDJ region.

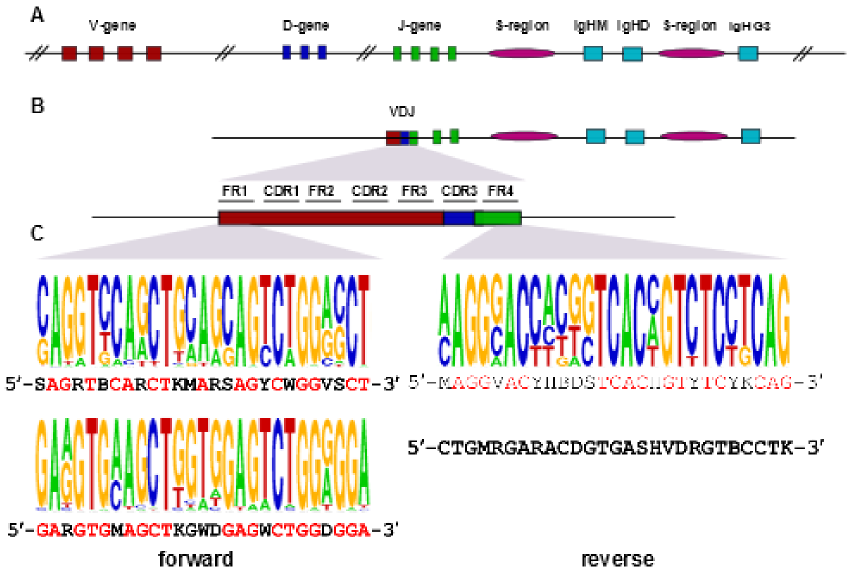

Figure 1: Schematic representation of VDJ recombination and primer design for amplification of the VDJ region. (A) The heavy chain immunoglobulin locus prior and (B) after VDJ recombination, including detailed representation of the rearranged VDJ segments with framework region 1-3 (FR 1-3) and complementarity-determining regions 1-2 (CDR1-2) on the V segment, CDR3 on the rearranged VDJ segments and FR4 on the J segment. (C) Frequency plots of the primer binding sites created in WebLogo and the corresponding primer sequences in bold.

Quantification of genomic DNA (gDNA)
4| Determine gDNA concentration of CD5+/CD19+ sorted CLL cells using the Qubit dsDNA HS Assay Kit according to the manufacturer's instructions.
5| 30 ng of gDNA is used as a template for PCR amplification in Step 7.

Amplification of the VDJ region of the B-cell receptor by PCR
6| Prepare primer mix for 1st PCR reaction (Phusion POL)
(i) Dilute primer stocks (100 µM) of RG1269, RG1270 and RG1271 to a final concentration of 10 µM using nuclease-free water, e.g. mix 5 µl of each 100 µM primer stock with 45 µl of nuclease-free water.
(ii) Mix equal volumes of each primer to generate a primer mix with 10 µM of each primer
7| Set up 1st PCR reaction in 0.2 ml PCR tubes as follows:

Table 2: Components for 1st PCR reaction

Component	Amount [µl or ng]	Final concentration
2X Phusion Master Mix with HF Buffer	10 µl	1X
Primer mix of RG1269/RG1270/RG1271 (10 µM each)	3 µl	1.5 µM each
Template DNA (gDNA, Step 2)	30 ng	1.5 ng/µl
Nuclease-free water	up to 20 µl	
**Total volume**	**20 µl**	

8| Start PCR reaction using the following cycler program:

Table 3: Cycler program for 1st PCR reaction

Cycle number	Temperature [C°]	Time	
1	98	30 s	
2	98	10 s	26 x
3	63	10 s	
4	72	30 s	
5	72	2 min	
6	4	hold	

Purification of the 1st PCR reaction
9| Preparations before start of the purification
(i) The optimal working temperature for the AMPure XP beads is on room temperature. Keep in mind to take them out of the fridge in time. 20 µl AMPure XP beads per sample are needed. Vortex AMPure XP beads for 30 seconds to generate a homogenous solution
(ii) Bring 10 mM Tris-HCl (pH 8.5) solution to room temperature. 52.5 µl is needed per reaction.
(iii) Prepare 80% ethanol solution freshly on room temperature. 400 µl is needed per sample.
10| Add 20 µl AMPure XP beads to each PCR reaction and mix well by pipetting up and down until beads are resuspended
11| Incubate the mixture for 5 min at room temperature

12| Place the samples on a magnetic stand (e.g. DynaMag-96 Side Magnet) and wait until the beads form a pellet and the supernatant is clear
13| Remove the supernatant without destroying the bead pellet
14| Keep samples on the magnetic stand while washing twice with 200 µl 80% ethanol solution. Remove ethanol after 30 seconds incubation without destroying the bead pellet.
15| Make sure to remove all remaining ethanol after each wash step
♣ CRITICAL STEP: Removal of as much ethanol as possible is critical here, to ensure the removal of residual primers of the 1st PCR reaction that could interfere with the 2nd PCR reaction. In addition, it is important to not destroy the bead pellet or lose beads by washing them out which leads to lower yields of the PCR product after elution.
16| Incubate on room temperature for 10 min until residual ethanol is evaporated, but do not over dry the bead pellet. The pellet should appear dry but not broken or cracked.
17| Take samples from magnetic stand and resuspend the dry bead pellet in 52.5 µl 10 mM Tris-HCl (pH 8.5) until the solution is homogenous
18| Incubate the mixture for 5 min at room temperature
19| Place the samples back on the magnetic stand and wait until the beads form a pellet and the supernatant is clear
20| Transfer 50 µl of the supernatant into a new PCR tube without destroying the bead pellet
Troubleshooting!

OPTIONAL: Analyze samples on a D1000 ScreenTape in a TapeStation system to monitor successful amplification and removal of residual primers. The PCR product should have a length of around 370 bp.

♣ PAUSE POINT: Reactions can be stored at -15° to -25°C for up to one week.

2nd PCR amplification for tagging of the VDJ region of the B-cell receptor
21| Prepare primer mix for 2nd PCR reaction (Phusion POL)
(i) Dilute primer stocks (100 µM) of RG1272, RG1273 and RG1274 together in one tube to a final concentration of 1 µM using nuclease-free water, e.g. pipette 10 µl of each 100 µM primer stock together with 970 µl of nuclease-free water.
22| Set up 2nd PCR reaction in 0.2 ml PCR tubes as follows:

Table 4: Components for 2nd PCR reaction

Component	Amount [µl]	Final concentration
2X Phusion Master Mix with HF Buffer	10 µl	1X
Primer mix of RG1272/RG1273/RG1274 (1 µM each)	5 µl	0.25 µM each
Template DNA (AMPure purified PCR product; Step 17)	2.5 µl	
Nuclease-free water	2.5 µl	
**Total volume**	**20 µl**	

23| Start PCR reaction using the following cycler program:

Table 5: Cycler program for 2nd PCR reaction

Cycle number	Temperature [C°]	Time	
1	98	30 s	
2	98	10 s	
3	63	10 s	26 x
4	72	30 s	
5	72	2 min	
6	4	hold	

Purification of the 2nd PCR reaction
24| Repeat step 9 to 20 to purify the PCR product of the 2nd PCR reaction

♣ PAUSE POINT: Reactions can be stored at -15° to -25°C for up to one week.

Determination of DNA quantity and quality
25| Analyze samples on a D1000 ScreenTape in a TapeStation system to monitor successful amplification and removal of residual primers. The PCR product should have a length of around 450 bp. Representative TapeStation electropherograms of a good and a critical run are shown in Figure 2.
Troubleshooting!

Figure 2: Representative TapeStation electropherograms

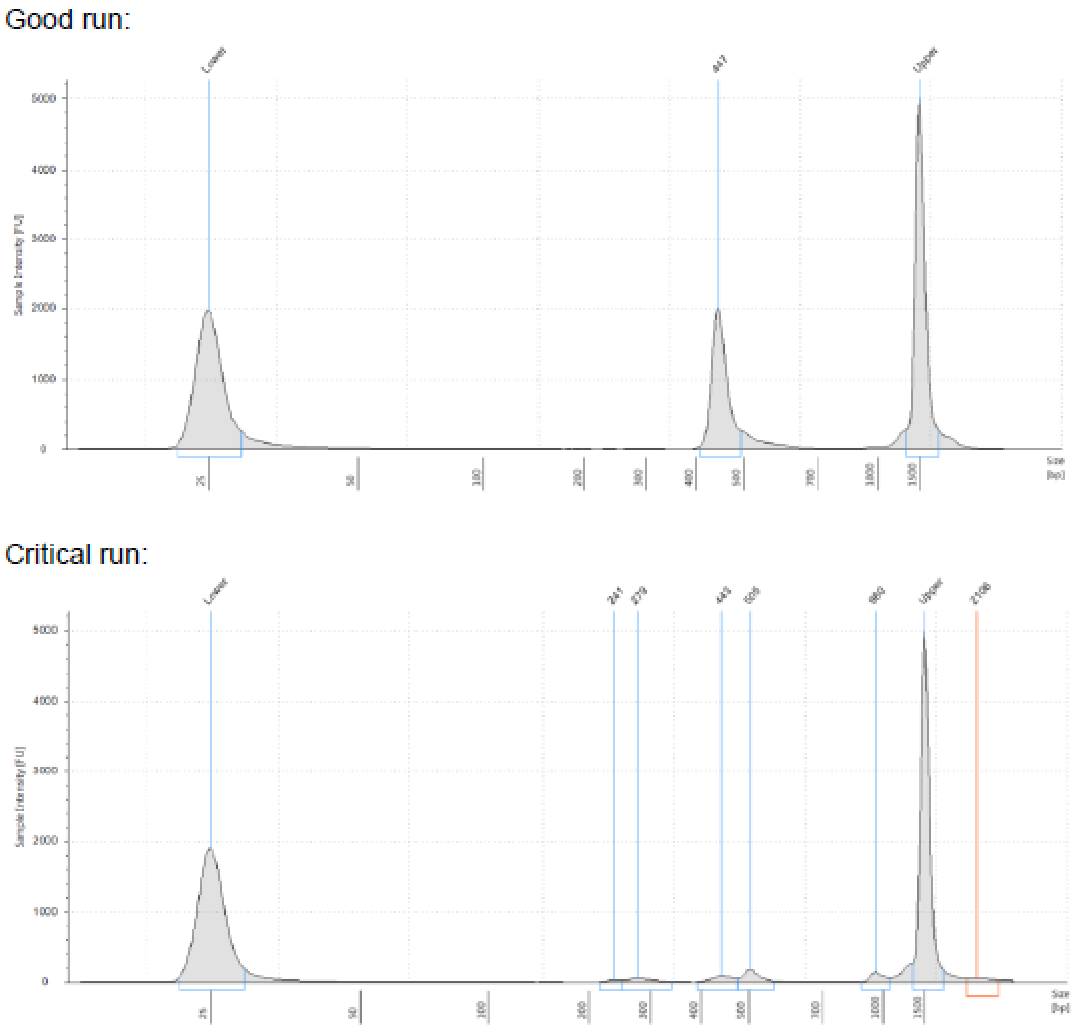

Figure 2: Representative TapeStation electropherogram of a good (top) and a critical (bottom) run of the 2nd PCR reaction and AMPure bead purification.

26| Determine the peak molarity [nmol/l] of the PCR product by integrating under the peak. Calculate the volume inserted for the indexing PCR (Step 27) as follows:

Table 6: Volume for the indexing PCR dependent on peak molarity

Peak molarity [nmol/l]	Volume [µl] for indexing PCR
> 10	1
5 – 10	2
0.5 - 5	3
< 0.5	6

3rd PCR amplification for indexing of samples for Illumina Sequencing

27| Set up indexing PCR reaction in 0.2 ml PCR tubes as follows:

Table 7: Components for indexing PCR reaction

Component	Amount [µl]
2x KAPA HiFi HotStart ReadyMix	10 µl
Nextera XT Index 1 Primers (N7XX)	2 µl
Nextera XT Index 2 Primers (S5XX)	2 µl
Template DNA (AMPure purified PCR product; Step 24)	1-6 µl
Nuclease-free water	0-5 µl
**Total volume**	**20 µl**

♣ CRITICAL STEP: All samples sequenced on one flow cell must have unique Index combinations

28| Start PCR reaction using the following cycler program:

Table 8: Cycler program for indexing PCR reaction

Cycle number	Temperature [C°]	Time	
1	95	3 min	
2	95	30 s	8 x
3	55	30 s	
4	72	30 s	
5	72	5 min	
6	4	hold	

Purification of the indexing PCR reaction

29| Repeat step 9 to 20 to purify the PCR product of the indexing PCR reaction with minor modifications

(i) Use 27.5 µl of 10 mM Tris-HCl (pH 8.5) for resuspension of the beads (Step 17)

(ii) Transfer 25 µl of the supernatant into a new DNA LoBind Tube (1.5 ml) without destroying the bead pellet (Step 20)

♣ PAUSE POINT: Reactions can be stored at -15° to -25°C for up to one week

Quantification of indexed DNA

30| Analyze 1:10 to 1:50 diluted samples (10 mM Tris-HCl, pH 8.5) on a D1000 ScreenTape in a TapeStation system to monitor successful amplification and removal of residual primers. The PCR product should have a length of around 520 bp. Check whether the main peak is above 90 % of the integrated area and no impurity occurs. Representative TapeStation electropherograms of a good and a critical run are shown in Figure 3. Troubleshooting!

CRITICAL STEP: Do not dilute the whole sample, only some µl for analysis in a TapeStation system

Figure 3: Representative TapeStation electropherograms

Good run:

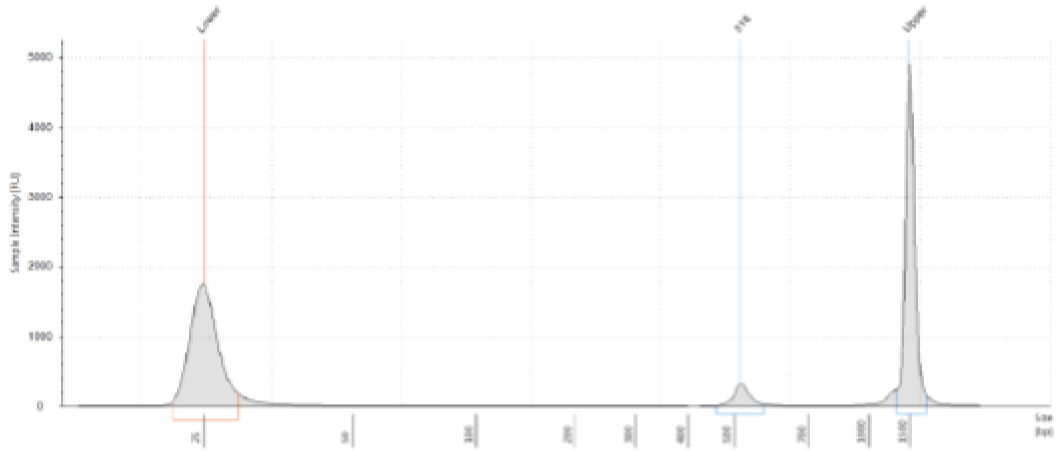

Critical run:

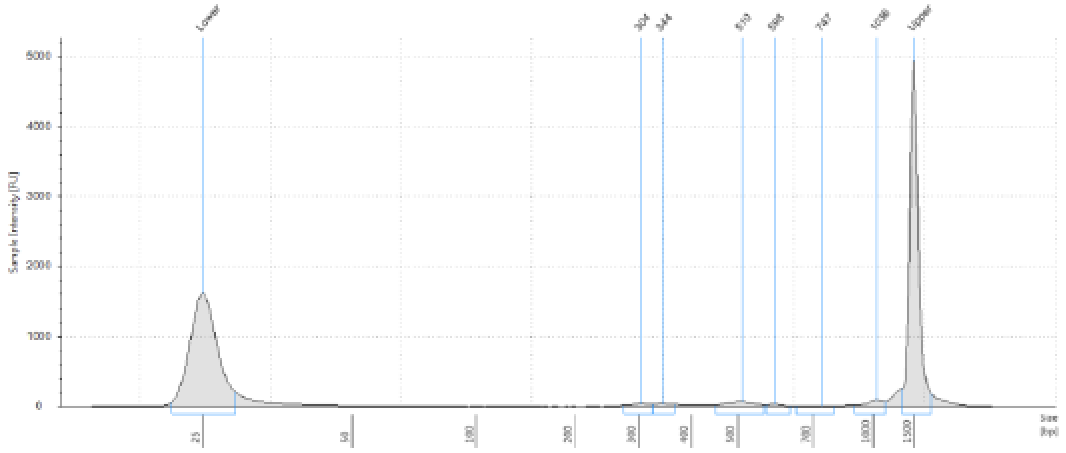

Figure 3: Representative TapeStation electropherogram of a good (top) and a critical (bottom) run of the indexing PCR reaction and AMPure bead purification of 1:20 diluted samples.

31| Determine the DNA concentration [ng/µl] of the purified PCR product (Step 29 (ii)) of the indexing PCR using the Qubit dsDNA HS Assay Kit according to the manufacturer's instructions.

32| Calculate the DNA concentration in nM as follows:

Equation 1: Calculation of DNA concentration in nM

$$conc.\,in\,nM = \frac{conc.\,in\,ng/\mu l}{660\frac{g}{mol} \times average\ library\ size\ (520\ bp)}$$

Pooling and denaturation of the libraries
33| Dilute each sample to a final concentration of 4 nM in 10 mM Tris-HCl (pH 8.5) in DNA LoBind Tubes.
34| Pool equimolar amounts, e.g. 5 µl per sample in a DNA LoBind Tube. Depending on the desired coverage, up to 3×96 samples can be pooled for one run on a MiSeqDx system using MiSeq Reagent Kit v3 (600-cycle) in a 300 bp paired-end run. In this setup with 96 multiplexed samples, a read depth of ~ 250.000 reads per sample is achieved.
35| Determine DNA concentration [ng/µl] of the library pool (Step 34) using the Qubit dsDNA HS Assay Kit according to the manufacturer's instructions.
CRITICAL STEP: The concentration of the pool should be 4 nM.

36| For denaturation of the library pool combine 5 µl of the 4 nM library pool with 5 µl of 0.2 N NaOH (freshly prepared) in a DNA LoBind Tube, mix by vortexing, spin down and incubate at room temperature for 5 min.
37| Add 990 µl pre-chilled HT1 buffer to obtain a 20 pM denatured library, keep the library on ice.
38| Dilute the denatured library to 8 pM by combining 240 µl of the 20 pM denatured library (Step 37) with 360 µl pre-chilled HT1 buffer.
39| Keep the diluted and denatured library on ice until sequencing.

Sequencing on a MiSeqDx system
40| Prepare the MiSeqDx system for a sequencing run according to the manufacturer's instructions.
41| Use the Illumina Experiment Manager Software in FASTQ only Run Settings to generate the sample sheet using the following parameters:

Table 9: MiSeqDx system run parameter

Parameter	Selection
Library Preparation Kit	Nextera XT
Index Reads	2
Read type	Paired End
Cycle read 1	301
Cycle read 2	301
**additional**	use Adapter Trimming

42| Sequence with the MiSeq Reagent Kit v3 (600-cycle).

Analysis of the B cell receptor repertoire
42| For bioinformatic analysis the MiXCR software v2.1.8 and the reference library repseqio.v1.4 was used. Change the species parameter setting to *Mus musculus*; the default setting is *Homo sapiens*.

<u>Timing</u>

Total time: ~ 5 days
Step 1-3 Primer design for amplification of the VDJ region of the B-cell receptor – 3 h
Step 4-5: Quantification of genomic DNA (gDNA) – 1h
Step 6-8: Amplification of the VDJ region of the B-cell receptor by PCR – 1 h

Step 9-20: Purification of the 1st PCR reaction – 1.5 h
21-23: 2nd PCR amplification for tagging of the VDJ region of the B-cell receptor – 1 h
24: Purification of the 2nd PCR reaction – 1.5 h
25-26: Determination of DNA quantity and quality – 1 h
27-28: 3rd PCR amplification for indexing of samples for Illumina Sequencing – 1 h
29: Purification of the indexing PCR reaction – 1.5 h
30-32: Quantification of indexed DNA – 1.5 h
33-39: Pooling and denaturation of the libraries – 1.5 h
40-41: Sequencing on a MiSeqDx system – 2 d
42: Analysis of the B cell receptor repertoire – 1 d

The timing information is based on the preparation of 24 samples, for more samples consider more time.

Troubleshooting

Table 10: Troubleshooting

Step	Problem	Possible reason	Solution
20	No amplification of the PCR product	• Incorrect/not enough template or primer<br>• Lose of beads during AMPure purification	increase amount of template DNA, ensure good DNA integrity
20	Low-molecular impurities	Insufficient washing during AMPure purification	Improve purification procedure
25	No or low yields of the PCR product	• Incorrect/not enough template or primer<br>• Lose of beads during AMPure purification	Adapt volume inserted for the 2nd PCR
25	Low-molecular impurities	Insufficient washing during AMPure purification	Improve purification procedure
30	No or low yields of the PCR product	• Incorrect/not enough template DNA<br>• Lose of beads during AMPure purification	Adapt volume inserted for the indexing PCR
30	Low-molecular impurities	Insufficient washing during AMPure purification	Improve purification procedure

Anticipated Results

Fast and precise analysis of the amino acid sequence of the BCR CDR3 region in mice by high-throughput sequencing on an Illumina MiSeqDx system.

References

Bolotin, C.A. et al. MiXCR: software for comprehensive adaptive immunity profiling. Nat. Methods. 12, 380-381 (2015).

Lefranc, M.-P. and Lefranc, G. The Immunoglobulin FactsBook. Academic Press, London, UK (458 pages), (2001).

Crooks G.E. et al. WebLogo: A sequence logo generator. Genome Research 14, 1188-1190 (2004).

Schneider T.D., Stephens R.M. Sequence Logos: A New Way to Display Consensus Sequences. Nucleic Acids Res. 18, 6097-6100 (1990).

Associated Publications

This protocol is related to the following articles:

- **Exome sequencing of the TCL1 mouse model for CLL reveals genetic heterogeneity and dynamics during disease development**

  doi:

Author information

Affiliations

Salzburg Cancer Research Institute - Laboratory for Immunological and Molecular Cancer Research (SCRI-LIMCR); Cancer Cluster Salzburg, Austria

Nadja Zaborsky, Maria Schubert, Franz J. Gassner & Roland Geisberger

Unaffiliated

Competing financial interests

The authors declare no conflicting financial interests.

Corresponding author

Correspondence to:

- **Nadja Zaborsky** (n.zaborsky@salk.at)
